# Characterization of the immunophenotypes and antigenomes of colorectal cancers reveals distinct tumor escape mechanisms and novel targets for immunotherapy

**DOI:** 10.1186/s13059-015-0620-6

**Published:** 2015-03-31

**Authors:** Mihaela Angelova, Pornpimol Charoentong, Hubert Hackl, Maria L Fischer, Rene Snajder, Anne M Krogsdam, Maximilian J Waldner, Gabriela Bindea, Bernhard Mlecnik, Jerome Galon, Zlatko Trajanoski

**Affiliations:** Biocenter, Division of Bioinformatics, Medical University of Innsbruck, Innrain 80, 6020 Innsbruck, Austria; Department of Medicine 1, University of Erlangen-Nuremberg, 91054 Erlangen, Germany; INSERM U872, Integrative Cancer Immunology Laboratory, 75006 Paris, France; Cordeliers Research Centre, Université Pierre et Marie Curie Paris 6, 75006 Paris, France

## Abstract

**Background:**

While large-scale cancer genomic projects are comprehensively characterizing the mutational spectrum of various cancers, so far little attention has been devoted to either define the antigenicity of these mutations or to characterize the immune responses they elicit. Here we present a strategy to characterize the immunophenotypes and the antigen-ome of human colorectal cancer.

**Results:**

We apply our strategy to a large colorectal cancer cohort (n = 598) and show that subpopulations of tumor-infiltrating lymphocytes are associated with distinct molecular phenotypes. The characterization of the antigenome shows that a large number of cancer-germline antigens are expressed in all patients. In contrast, neo-antigens are rarely shared between patients, indicating that cancer vaccination requires individualized strategy. Analysis of the genetic basis of the tumors reveals distinct tumor escape mechanisms for the patient subgroups. Hypermutated tumors are depleted of immunosuppressive cells and show upregulation of immunoinhibitory molecules. Non-hypermutated tumors are enriched with immunosuppressive cells, and the expression of immunoinhibitors and MHC molecules is downregulated. Reconstruction of the interaction network of tumor-infiltrating lymphocytes and immunomodulatory molecules followed by a validation with 11 independent cohorts (n = 1,945) identifies BCMA as a novel druggable target. Finally, linear regression modeling identifies major determinants of tumor immunogenicity, which include well-characterized modulators as well as a novel candidate, CCR8, which is then tested in an orthologous immunodeficient mouse model.

**Conclusions:**

The immunophenotypes of the tumors and the cancer antigenome remain widely unexplored, and our findings represent a step toward the development of personalized cancer immunotherapies.

**Electronic supplementary material:**

The online version of this article (doi:10.1186/s13059-015-0620-6) contains supplementary material, which is available to authorized users.

## Background

Recent studies using large cohorts and next-generation sequencing (NGS) technologies are providing a wealth of information and have revealed the genomic landscapes of common human cancers [1]. But so far little attention has been devoted to either define the cancer antigen-ome (that is, the repertoire of the tumor antigens) or to elucidate the immune responses they elicit. This knowledge could be exploited for gaining mechanistic insights into tumor progression and for the development of cancer immunotherapies.

Several types of immunotherapies have been shown to have great clinical impact, including adoptive T-cell transfer therapy, cellular vaccines, and checkpoint blockade inhibitors, such as the FDA-approved anti-CTLA4 monoclonal antibody (ipilimumab) and antibodies that block signaling through PD-1 and PD-L1 [2,3]. However, in cancer patients responding to immunotherapy it is not known which antigens are responsible for tumor regression and the elucidation of the cancer antigenome is thus an important requirement for identifying antigens which induce an adaptive immune response [4]. For example, a recent study described a screening platform to detect neo-antigen-specific CD4+ T cells [5] based on exome and RNA sequencing of the tumor followed by peptide synthesis and co-culture of neo-antigen-loaded B cells and CD4+ T cells. Furthermore, the identification of highly immunogenic tumor antigens is a prerequisite for developing personalized cancer vaccines as shown in a proof-of-concept study that demonstrated in a mouse model that a therapeutic pipeline based on NGS analysis, neo-antigen prediction and selection, and peptide synthesis followed by vaccination is feasible [6]. Relevance of this type of whole exome-based analysis in human cancer has been subsequently shown in melanoma [7].

Without doubt, given the exciting development of these immunotherapeutic strategies, the importance and clinical relevance of intratumoral immune landscapes and cancer antigenomes is becoming increasingly appreciated. In a seminal paper, epitope prediction algorithms were used to identify candidate tumor antigens [8]. Only recently, the first attempt to explore genomic data was performed by carrying out meta-analysis for several cancers and it could be shown that neo-antigens were associated with increased patient survival [9]. However, the number of subjects for individual cancer types was relatively small (515 patients for 6 different tumor sites) and did not allow analysis for specific cancer subtypes (for example, microsatellite instable (MSI) or microsatellite stable (MSS) tumors in colorectal cancer (CRC) patients). Moreover, since the expression of only three immune genes was assessed, tumor-infiltrating lymphocytes (TILs) were completely undefined. Therefore, the adaptive immune response remained elusive.

In order to comprehensively characterize the antigenicity and immunogenicity of human CRC, we developed an analytical strategy and examined genomic data sets from The Cancer Genome Atlas (TCGA; n = 598) [10]. We first defined a compendium of immune genes using expression data from purified immune cells and used RNA sequencing data to identify subpopulations of TILs. Specific TILs were associated with distinct molecular phenotypes (hypermutated and non-hypermutated phenotype; MSS and high levels of microsatellite instable (MSI-H) phenotype; and CpG island methylation (CIMP) phenotype). Next we used RNA- and whole-exome NGS data to chart the antigenome comprising two major classes: cancer-germline antigens and neo-antigens, and could show that neo-antigens were rarely shared between patients. We then analyzed the genetic basis of the tumors and revealed distinct tumor escape mechanisms for the patient subgroups. Finally, we used two modeling approaches, namely reconstruction of the interaction network of TILs and immunomodulatory molecules, and linear regression analyses of the determinants of immunogenicity, and identified novel candidates for immunotherapy. These targets were subsequently validated using large independent cohorts and an orthologous immunodeficient mouse model.

## Results

### Tumor-infiltrating lymphocyte subpopulations are associated with distinct molecular phenotypes in colorectal cancer

An overview of the analytical strategy and the methods used is shown in Figure S1 in Additional file 1. We first built a compendium of genes (1,980) related to specific immune cells using gene expression profiles assessed in purified immune cells and extracted from 36 studies comprising 813 microarrays (Figure S2 in Additional file 1; Table S1 in Additional file 2). A subset of these genes which are representative for specific immune cell subpopulations were then selected based on correlation analysis (Table S2 in Additional file 2). In the following we call these genes immune metagenes (812 in total). The expression of the immune metagenes as determined by RNA sequencing was used to estimate 28 subpopulations of TILs, including major types related to adaptive immunity (central and effector memory CD4+/CD8+ cells, gamma delta T cells (Tgd), T-helper 1 (Th1), Th2, Th17, regulatory T cells (Treg), follicular helper T cells (Tfh), immature and memory B cells) as well as innate immunity (macrophages, monocytes, mast cells, eosinophils, neutrophils, dendritic cells (DCs), natural killer (NK) cells, and natural killer T cells (NKT).

**Figure 1:**
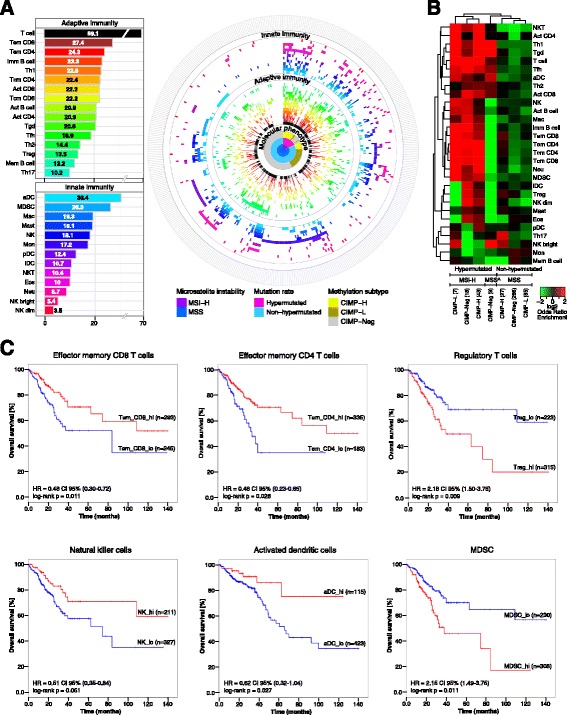
**Molecular phenotypes and immunophenotypes of CRC tumors. (A)** Subpopulations of TILs enriched in the tumors and spin chart for the TCGA cohort . The numbers for the subpopulations represent percentages of tumors with enriched immune cell subpopulations. T-cell compartment includes all T-cell subpopulations. Tem, effector memory T cell; Tcm, central memory T cell; Act, activated; aDC, activated dendritic cell; pDC, plasmacytoid dendritic cell; iDC, immature dendritic cell; Mac, macrophages; Eos, eosinophils; Neu, neutrophils; NK, natural killer cells. The spin chart gives an overview of the cohort (n = 460) with each line representing one patient. The molecular phenotypes are shown in the inner circle. The subpopulations of TILs significantly (q-value ≤0.1) enriched in individual patients based on single sample gene set enrichment analysis (ssGSEA) are shown in the outer circles. Within each molecular phenotype, the patients are sorted according to the enriched immune cell types: the immune cell types are ordered by their odds ratio for the corresponding molecular phenotype in a descending order. CIMP-H, CIMP-high; CIMP-L, CIMP-low; CIMP-Neg, CIMP-negative. **(B)** Heat map of log-transformed odds ratios of the TIL subpopulations for three different molecular phenotypes and different combinations thereof (for example, hypermutated, MSI-H, and CIMP-L). **(C)** Kaplan-Meier (KM) curves for overall survival for patients using the relative number of immune cells (Tem CD8, Tem CD4, Treg, NK, aDC and MDSC). Shown are groups with high relative numbers of cells (hi, red) versus the low relative number of cells (lo, blue) at the optimum value cutoff. CI, confidence interval; HR, hazards ratio.

The enrichment analysis of TIL subpopulations using metagene expression profiles indicated that TILs are present in 96.6% of the tumors and that 70% of the tumors show a T-cell phenotype (Figure 1A), previously defined as inflamed phenotype [11]. These results are consistent with our previous studies [12,13]. The higher resolution of the analysis in the present study revealed a comprehensive picture of the intratumoral immune landscape and showed high enrichment of many subpopulations of both adaptive and innate immunity. The adaptive immunity subpopulations with highest enrichments included effector memory CD4+ and CD8+ cells, immature B cells, and Th1 cells, whereas the innate immunity subpopulations included activated DCs, myeloid-derived suppressor cells (MDSCs) and macrophages (Figure 1A; see also interactive version at [14]). The metagene expression signatures were then used to associate TIL subpopulations with distinct molecular phenotypes from the TCGA cohort related to the mutational status (hypermutated and non-hypermutatated), the microsatellite status (MSS and MSI-H), and the methylation status (CIMP-high, CIMP-low, and CIMP-negative [15]) (Figure 1A). As can be seen, there is a partial overlap between the different phenotypes (for example, all MSI-H tumors are hypermutated and most of them belong to the CIMP-high group).

The quantification of TIL subpopulations showed that TILs were associated with distinct molecular phenotypes related to three features (mutational status, microsatellite status, and methylation status) or combinations thereof (Figure 1B). It should be noted that from 12 possible combinations (2 mutation rate statuses × 2 MSI statuses × 3 methylation subtypes) 5 groups were excluded due to a small number of samples. As expected, MSI-H tumors were characterized by enrichment of TILs mostly related to adaptive immunity. A small group of the hypermutated tumors with MSS phenotype (denoted as MSS^) were characterized by lower enrichment of effector memory and central memory CD4+ and CD8+ cells. Interestingly, in the CIMP-high groups the only cell types that were enriched in MSS tumors but not in MSI-H tumors were Th17 cells and NKbright cells. Thus, combining RNA sequencing data and immune metagenes enables precise molecular taxonomy for any CRC phenotype characterized by genomic instability, mutational load, or epigenetic instability.

### Immunophenotypes evolve during tumor progression

We have previously shown that individual TIL subpopulations are associated with overall survival (OS) and disease-free survival (DFS), including CD8+ T cells [12] and B cells [13]. The present analyses of high-resolution data (RNA-seq) enabled us for the first time to comprehensively chart the immune landscape and identify the associations of major TIL subpopulations with OS (Figure 1C; Figure S3 in Additional file 1). Results of this univariate analysis were comparable with those of the multivariate analysis using a Cox regression model for correcting for the molecular phenotypes (Table S3 in Additional file 2). This is in concordance with our previous study, which showed that immune signatures have the highest predictive accuracy [16]. TILs associated with good prognosis included effector memory and central memory CD8+ cells, effector memory CD4+ cells, NK cells, and activated DCs, whereas MDSCs, Tregs, Th17, and mast cells were associated with bad prognosis.

Tumor progression is characterized by changes in the genomic landscape and clonal architecture. In order to analyze the immune phenotypes during tumor progression, the examination of early and advanced lesions from the same patient or matching primary and metastases would be required. While comparisons across patients are less robust due to intratumor variations, we attempted to identify immune subsets during tumor progression using the TCGA data. We focused on non-hypermutated tumors (MSS patients) since there were only two MSI-H and MSS^ patients with stage IV tumors. In the MSS patients the number of mutations increased during progression from stage I to stage IV (Figure S4 in Additional file 1). Intriguingly, the number of subclonal mutations slightly decreased (*P* = 0.002, r = -0.16). The progression of the tumor was characterized by distinct immune patterns. For example, activated CD8+ cells were significantly enriched from stage I to stage III tumors and depleted in stage IV tumors (Figure S4 in Additional file 1). In contrast, Tregs were depleted in stage I and stage II and enriched in stage III and stage IV tumors. In general, the enrichment of the TIL subpopulations related to adaptive immunity decreased whereas enrichment of TILs related to innate immunity increased. This was also evident at the level of individual markers (Figure S5 in Additional file 1).

### Colorectal cancer antigenome is diverse and sparse

Comprehensive characterization of the cancer antigenome is a prerequisite for developing personalized cancer immunotherapies, that is, for selecting antigens for cancer vaccination. For cancer vaccination two types of antigens with high tumor specificity can be used: neo-antigens arising from somatic mutations which are unique to each tumor and non-mutated cancer-germline antigens which are shared between many tumors [17]. We therefore characterized both antigen classes in three patient groups: MSI-H, MSS^, and MSS. RNA-sequencing data were used to predict at four-digit resolution the human leukocyte antigen (HLA) haplotype of each patient and to identify cancer-germline antigens. In order to identify neo-antigens we used 222,169 somatic mutations from the TCGA cohort, selected expressed mutations (207,679) and evaluated binding of all possible 8- to 11-mer mutant peptides (4,618,035) (Materials and methods).

**Figure 2:**
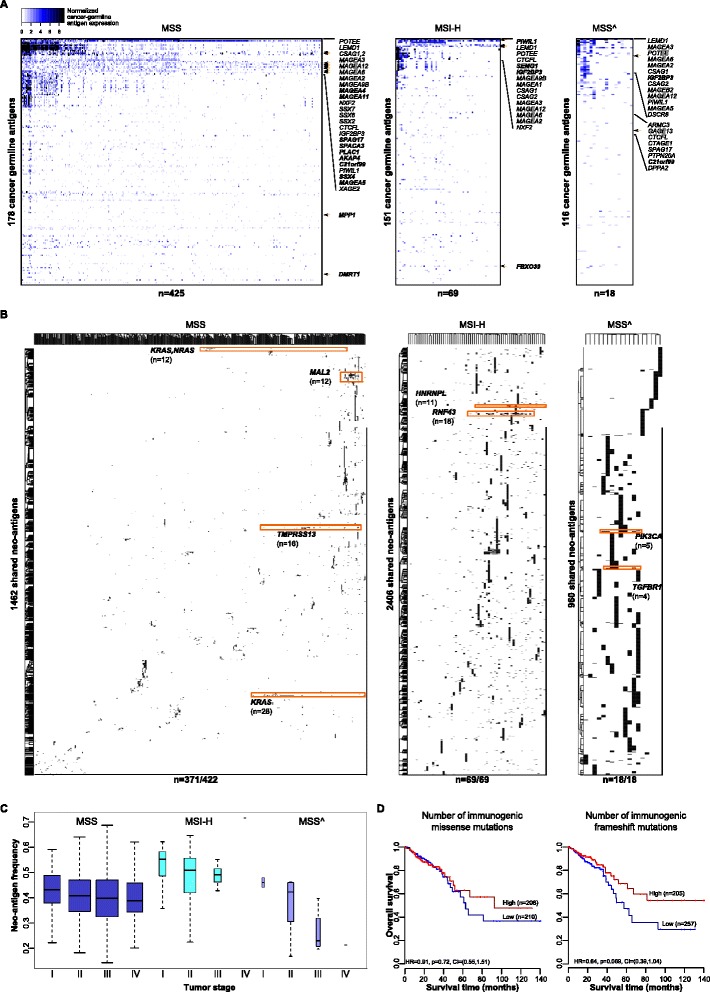
**CRC antigenome comprising two antigen classes: cancer-germline antigens and neo-antigens. (A)** Two-dimensional hierarchical clustering of the expression of cancer-germline antigens calculated from the RNA sequencing data for the three molecular phenotypes (MSS, MSI-H and MSS^). All displayed matrix elements met the threshold as described in Methods. Genes marked in bold were significantly higher expressed in a specific patient group. **(B)** Two-dimensional hierarchical clustering of neo-antigens, that is, identical peptides shared in more than two patients in the three groups. Highlighted are most frequent neo-antigens in the specific group and the corresponding mutated gene. **(C)** Neo-antigen frequencies from stage I to stage IV in MSS, MSI-H and MSS^ patients. **(D)** Survival analysis for the number of immunogenic missense and frameshift mutations in all CRC patients.

A large number of cancer-germline antigens (178) were detectable in all patients (Figure 2A). About 62% (110) of the expressed cancer-germline antigens were shared and only 8% (14) were specific for MSS, MSI-H, and MSS^ tumors. This intriguing result suggests that the expression of cancer-germline antigens is independent of both the molecular phenotype and the immunophenotype of the tumors.

In strong contrast, the neo-antigens were infrequently shared between patients (Figure 2B). From the total of 92,028 neo-antigens (19,241 for MSS, 42,562 for MSI-H and 29,717 for MSS^) only 4% (3,714) were shared between two or more patients (Figure 2B; Table S4 in Additional file 2). These shared neo-antigens represent identical peptides originating from one or more genes. The most frequent neo-antigens were induced by mutations in KRAS, RNF43, and PIK3CA in MSS, MSI-H, and MSS^ patients and were shared in 7%, 26% and 28% of the patients, respectively. Both, KRAS and PIK3CA are known to be frequently mutated in CRC. It was only recently shown that RNF43 is frequently mutated in CRC and endometrial cancers [18]. Peptides for the neo-antigens that are shared in at least seven patients and the corresponding genes are given in Figure S6 in Additional file 1 (see interactive version at [14]). The number of neo-antigens and the neo-antigen frequencies were 45 ± 22, 635 ± 308, and 1,651 ± 1,455, and 0.38, 0.48, and 0.39 for the MSS, MSI-H, and MSS^ patients, respectively. In MSS tumors the neo-antigen frequencies decreased from 0.43 in stage I to 0.39 in stage IV (*P* = 0.05; Figure 2C). Hence, with increasing tumor stage, tumor antigenicity appears to decrease, which might explain why prognosis is inversely related to tumor stage at diagnosis. It can be envisaged that in stage I there is a greater presence of antigenic tumor cells and/or that the immune cells are less anergic or exhausted [19]. Finally, survival analysis showed an association of the number of immunogenic missense and frameshift mutations with survival (Figure 2D), albeit not significant.

### Genetic basis of the tumors determines tumor escape mechanisms

**Figure 3:**
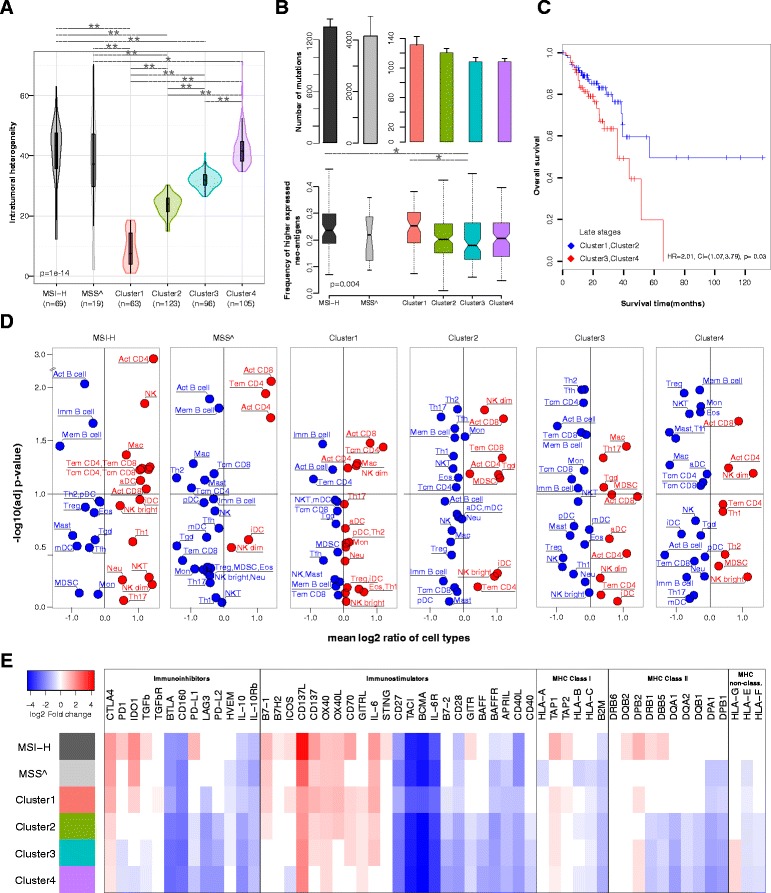
**Tumor heterogeneity and intratumoral immune landscapes in hypermutated and non-hypermutated tumors. (A)** Violin plots (that is, box plots with a rotated probability density plot) of intratumoral heterogeneity represented by the calculated cancer cell fractions in MSI-H and MSS patients. MSS tumors (colored plots) were grouped in four clusters based on their cancer cell fraction distributions. The Kolmogorov-Smirnov D statistic was used as a similarity measure to cluster the tumors into the four groups (Figure S7 in Additional file 1). **(B)** Number of mutations and neo-antigen frequencies in the hypermutated tumors (MSI-H and MSS^) and in the four clusters of non-hypermutated tumors (MSS) colored as in panel **(A)**. * *P >* 0.01, ** *P* < 0.01. Error bars represent standard error of the mean. **(C)** Survival analysis of the homogeneous (cluster1 and cluster2) and heterogeneous (cluster3 and cluster4) MSS tumors in late stages. **(D)** Volcano plots for enriched (red) and depleted (blue) TIL subpopulations in the distinct patient groups compared with normal samples (n = 50). **(E)** Log2 fold change of the expression (RNA sequencing data) of selected genes relative to normal tissue for the MSI-H and MSS patients colored as in **(A)**.

Our analysis of the antigenome landscape of CRC demonstrated that the majority of the patients, including patients with lower mutational load (MSS), express neo-antigens and, hence, cancer vaccination may be effective also in these patients. However, tumor mutational heterogeneity adds another layer of complexity and may hinder personalized strategies [20]. Within a tumor, clones may be present which do not elicit T-cell responses against a given neo-antigen used in the vaccine and these cells may thus have selective advantage and outgrow other clones. Using SNP-array data and whole-exome NGS data we calculated the cancer cell fractions and estimated the tumor heterogeneity for MSS and MSI-H patients. Two different algorithms were applied for this analysis, ABSOLUTE [21] and PyClone [22], which led to similar results (data not shown). MSS patients were then clustered into four groups based on the cancer cell fractions (Figure S7 in Additional file 1). As expected, MSI-H tumors were the most heterogeneous ones and had the highest neo-antigen frequencies (Figure 3A,B). In the MSS phenotype the most homogeneous tumors (cluster 1) had the largest neo-antigen frequencies (Figure 3C). Survival analyses showed that MSS patients with homogeneous tumors (clusters 1 and 2) had better prognosis (hazard ratio 0.58, confidence interval 0.3 to 1.12) than the patients with more heterogeneous tumors (clusters 3 and 4) (Figure 3C).

We then characterized the immunophenotypes for the genetically different tumors and visualized the immune subpopulations, which were enriched and depleted compared with normal tissue, using volcano plots (Figure 3D). Major differences in the enrichment of TIL subpopulations could be observed in the MSI-H, MSS^, and the four MSS subtypes. MSI-H tumors were enriched with all CD4 and CD8 subpopulations as well as with activated DCs and NK cells. The immune response was less pronounced in MSS^ tumors, and was further dampened in the MSS tumors, albeit all groups were enriched with cytotoxic cells. Interestingly, in the MSS tumors the number of significantly depleted subpopulations was proportional to the tumor heterogeneity and increased from cluster 1 to cluster 4 (Figure 3D).

The genetic and immunophenotypic variability of the CRC tumors shown above impose the question of whether different tumors use different mechanisms of tumor escape. This knowledge is of utmost importance for making decisions about the most appropriate immunotherapy regimen. We carried out systematic analysis of the escape mechanisms and examined the enrichment of immunosuppressive cell types (MDSCs and Tregs), and the expression of five classes of molecules: key immunoinhibitory genes which may be upregulated to produce tumor escape (for example, CTLA-4), key immunostimulatory genes (for example, OX40), which may be downregulated to avoid immune destruction, as well as major histocompatibility (MHC) class I, MHC class II, and non-classical MHC molecules. We included all previously reported immunomodulators [23,24] and the recently identified candidate STING (stimulator of interferon genes complex) [25].

In the hypermutated tumors (MSI-H and MSS^) Tregs and MDSCs were depleted, whereas in the non-hypermutated tumors (MSS clusters) either Tregs (cluster 1) or MDSCs (clusters 2 to 4) were enriched (Figure 3D). The expression profiles of the immunomodulatory genes showed consistent upregulation of CD137L and down regulation of BTLA, CD160, CD27, TACI, BCMA and IL-6R in all tumors (Figure 3E). In the hypermutated tumors the immunoinhibitors CTLA4 and IDO1 were highly upregulated whereas PD1 and PD-L1 were upregulated only in the MSI-H group (Figure 3E; Figure S8 in Additional file 1). In the non-hypermutated tumors a number of immunoinhibitors were significantly downregulated (Figure S8 in Additional file 1). Strikingly, in these samples, the tumor heterogeneity was proportional to the decreasing expression of many immunomodulators, as well as MHC class I and class II molecules. Notably, clusters 3 and 4 showed increased expression of HLA-G molecules, which is associated with worse OS and DFS in CRC [26]. Thus, it appears that the genetic basis of the tumors determines the tumor escape mechanisms. In hypermutated tumors immunoinhibitors are upregulated and immunosuppressive cells are depleted. In non-hypermutated tumors (MSS) immunosuppressive cells are enriched and the immunoinhibitors are downregulated. It is noteworthy that within the group of non-hypermutated tumors, the tumor heterogeneity might represent an additional parameter, which can be used to identify the patients that would benefit from targeted immunotherapy. However, further validation and functional studies are required to investigate the predictive power of this parameter.

### Reconstruction of the interaction network of TILs and T-cell immunomodulatory molecules reveals candidates for immunotherapy

**Figure 4:**
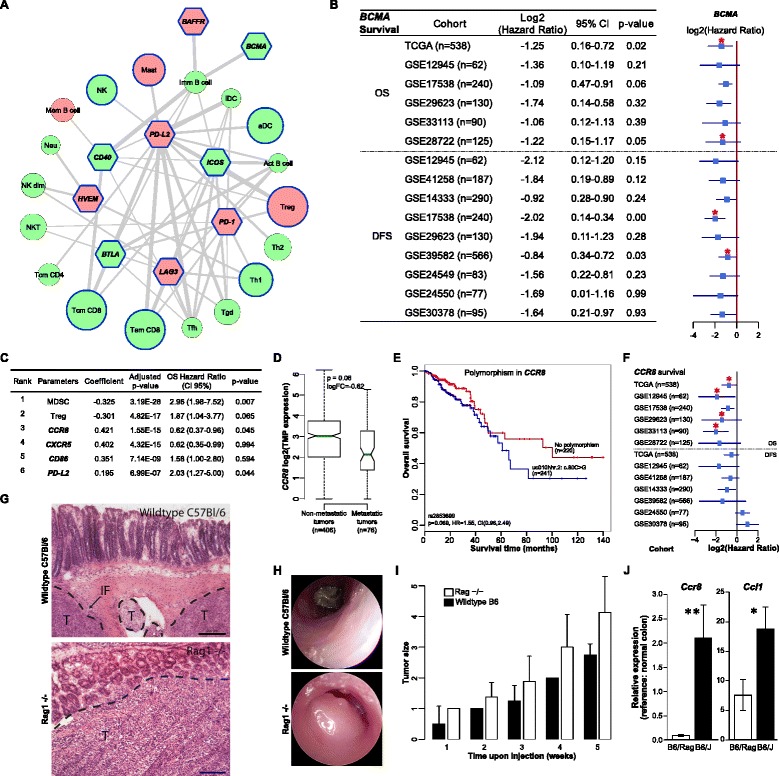
**Determinants of tumor immunogenicity in human CRC. (A)** Network of TILs and immunomodulatory molecules. Shown are only candidate genes (hexagons) which were significantly associated with survival in the TCGA cohort. The thickness of the edges is proportional to the strength of the pairwise gene correlation. The size of the circles (TILs) is inversely proportional to the log-rank *P*-value, with large circles representing lower *P*-values. Significant association with OS is indicated by a blue node border. Bad prognosis is represented by red node color, good prognosis by green node color. **(B)** Survival analysis for BCMA using RNA sequencing data (TCGA cohort) as well as microarray data from 11 independent cohorts. OS, overall survival; DFS, disease-free survival. A forest plot with the corresponding hazard ratios is shown on the right. Red asterisks mark the cohorts for which the results are significant. CI, confidence interval. **(C)** Table with the major determinants of tumor immunogenicity identified using linear regression modeling, the corresponding coefficients and the adjusted *P*-values. Shown are also the results of the survival analysis for the TCGA cohort, including OS hazard ratio and the *P*-values. **(D)** Expression of the chemokine CCR8 in patients with and without metastasis in the TCGA cohort calculated from the RNA sequencing data. **(E)** Kaplan-Meier curves for germline mutations in CCR8 for the TCGA cohort. **(F)** Survival analysis for CCR8 in the TCGA cohort (RNA sequencing data) and 10 independent cohorts (microarray data). Red asterisks mark the cohorts for which the results are significant. **(G)** Hematoxylin and eosin staining of tissue sections from an orthotopic mouse model using murine MC38 cell line in wild-type C57Bl/6 and immunodeficient RAG1-/- mice. IF, immune cell infiltration; T, tumor. Black bar: 200 μm. **(H,I)** Endoscopic scoring **(H)** and tumor growth **(I)** after injection of MC38 cells (10^4^) into the submucosa of wild-type C57Bl/6 and RAG1-/- mice. Error bars represent standard error of the mean. **(J)** Expression of CCR8 and its ligand CCL1 in the orthotopic mouse model of CRC. * *P =* 0.038, ** *P* = 0.017. Error bars represent standard error of the mean.

A number of immunoinhibitory and immunostimulatory molecules are currently under investigation for immunotherapy in various cancers [27]. In an attempt to identify promising candidates for CRC immunotherapy, we reconstructed a network of TILs and immunomodulatory molecules for which agonists/antagonists are available. The interaction network between TILs and these druggable targets was then filtered for molecules which were significantly associated with good or bad prognosis in the TCGA data set. The highly interconnected subpopulations of TILs were Tfh, B cells, immature DCs, Tgd, Tregs, and CD8+ cells (Figure 4A). The molecules significantly associated with survival included PD-1 and PD-L2. To validate the candidates we then collated public expression data sets from 11 microarray studies comprising a total of 1,945 patients (Table S5 in Additional file 2). Survival analyses carried out for the nine candidates showed that BCMA (TNFRSF17) was consistently associated with good prognosis in all cohorts (Figure 4B; Figure S9 in Additional file 1). BCMA is a member of the tumor necrosis factor receptor superfamily and was first described as B-cell maturation factor [28]. The receptor specifically binds to BAFF (TNFRSF13B) and leads to the activation of NF-kappa B and MAPK8/JNK [29]. An antagonist is currently in phase II/III trials for other indications and in phase I for non-Hodgkin lymphoma and multiple myeloma [27].

### Linear regression modeling identifies major determinants of tumor immunogenicity in colorectal cancer

Antigenicity of the tumor is necessary but not sufficient for it to be immunogenic, that is, to elicit adaptive immune responses *in vivo*. The microenvironment of the tumor can be immunosuppressive and prevent anti-tumor immunity. Our previous study [13] and the results of this work show not only highly heterogeneous TILs but also varying ratios of different T-cell subsets, including suppressive ones. These observations raise the question of the underlying molecular mechanisms that explain the differences in immunogenicity of the tumors. The question can be reduced to the notion of sources of immunogenic differences, which can be divided into two categories: tumor intrinsic factors and tumor extrinsic factors. Tumor intrinsic factors include the cancer antigenome, the expression of immunoinhibitors and immunostimulators (for example, PD-L1 [17]), and HLA class I molecule alterations [30]. Tumor extrinsic factors include chemokines which regulate T-cell trafficking [31], immunosuppressive TILs, soluble immunomodulatory factors (cytokines) [31], and germline polymorphisms in immune regulatory genes [32]. We examined major tumor intrinsic and extrinsic factors in order to comprehensively characterize the tumor immunogenicity. A linear regression model including all determinants (221 parameters; Table S6 in Additional file 2) was applied to predict the presence of cytotoxic cells (approximated as the average of the expression levels of the CD8+, NK, and Tgd cell metagenes). The number of features in the linear model was reduced by lasso-regularization [33] and only variables with significant coefficients (*P* < 0.001) were included in the final model. The model was evaluated by a 10-fold cross-validation procedure. The identified features disclosed the major determinants, which were ranked as follows: MDSCs, Tregs, CD86/B7-2, CCR8, CXCR5, and PD-L2) (Figure 4C).

All but one of these determinants have been previously implicated as modulators of the tumor microenvironment. MDCSs and Tregs are well-known immunosuppressors [31]. CD86/B7-2 is ligand for CTLA4 whereas PD-L2 is ligand for PD-1. Clinical trials of blocking monoclonal antibodies against CTLA4 and PD-1 are currently underway for a variety of epithelial malignancies, including CRC [34]. CXCR5 is a receptor for the CXCL13 chemokine, for which we recently showed that genomic instability is a mechanism associated with intratumoral Tfh and B-cell infiltration in CRC [13]. A novel candidate identified in the present study was the chemokine receptor CCR8.

We then analyzed the available data and provide here several lines of evidence for the involvement of CCR8 in tumor progression in human CRC: 1) CCR8 is downregulated in metastatic tumors compared with non-metastatic tumors in the TCGA cohort (Figure 4D); 2) patients with germline mutations in CCR8 have lower survival rates, albeit not significant (*P* = 0.069; Figure 4E); 3) expression of CCR8 in the TCGA cohort was significantly associated with good prognosis. We also analyzed the expression of CCR8 and its ligand CCL1 using RNA sequencing data from the TCGA cohort as well as using unpublished quantitative real-time PCR data from our previous study [35] comprising 125 samples and show that CCR8, and to lesser extent CCL1, are expressed in human CRC tissue (Figure S10 in Additional file 1). We then validated the prognostic power of CCR8 in the TCGA cohort and 10 additional cohorts (n = 1,862; in one cohort the probes for CCR8 were not identifiable; Figure 4F). These results indicate that CCR8 is a novel candidate for modulating immunogenicity in CRC.

There are conflicting results in the literature on the expression of CCR8 in various cell types. Originally it was shown that CCR8 is expressed in Th2 cells and is involved in eosinophil recruitment [36]. In a recent study it was reported that CCR8 is expressed in Tregs [37] whereas another study using a melanoma model reported that CCR8 is expressed on tumor cells [38]. This study showed that blocking CCR8 or CCL1 inhibits tumor cell migration to lymphatic endothelial cells and that CCR8 is also expressed in human metastatic melanoma. In order to further elucidate the role of CCR8 in CRC we used an orthotopic CRC mouse model. Mouse CRC cell line MC38 was injected endoscopically into the colonic submucosa of syngenic C57Bl/6 wild-type and RAG1-/- immunodeficient mice and the tumor growth was monitored weekly using endoscopy. The morphology of these tumors is comparable to human CRC, showing infiltrative growth through the mucosa and an immune response within the tumor and at the tumor margin (Figure 4G). Tumor growth was monitored by endoscopy weekly (Figure 4H) and developed faster in RAG1-/- mice (Figure 4I). The expression of CCR8, and to lesser extent its ligand CCL1, were very low in immunodeficient RAG1-/- mice (Figure 4J), indicating that CCR8 is primarily expressed in TILs in CRC.

## Discussion

We have developed an integrated strategy and for the first time characterized the immunophenotypes and the antigenome of human CRC. Our approach to deeply mine large datasets enabled us not only to disentangle tumor-immune cell interactions, but also to devise strategies for cancer immunotherapy in CRC. The dissection of the complex tumor microenvironment suggests several important biological conclusions.

First, the results support the notion that the subpopulations of TILs mirror the underlying molecular phenotypes. Moreover, based on the enrichment of TIL subpopulations, specific characterization of all three studied molecular phenotypes (mutational status, microsatellite status, methylation status) or the combinations thereof is possible. Hence, a single assay (RNA sequencing) enables refined molecular taxonomy of the tumors. One possible explanation for this observation is that the number of features (expression of several hundred mRNAs) with cell type-related expression patterns is two orders of magnitude larger than the features used for traditional molecular phenotyping. Notably, the use of well-characterized expression of gene signatures in pure cell types reduces the noise inherent in high-throughput expression studies. With the advent of single cell sequencing techniques [39] and the development of deconvolution algorithms [40], we expect that future studies will allow even more precise characterization of the intratumoral immune landscape. We advocate that RNA sequencing should be routinely performed on tumor specimens and might lead to the identification of additional molecular subtypes.

Second, the antigenicity of the tumors and the intratumoral immune landscape evolve in parallel during tumor progression, with decreasing antigen frequencies, alleviated adaptive immunity response, and intensified innate immunity. The data also indicate that the mutational load has a major impact on the number of TILs as observed in the MSI-H and MSS^ groups. In the patient group with lower mutational load (MSS), it appears that tumor antigenicity decreases to a larger extent, as shown by an increasing number of mutations and decreasing neo-antigen frequencies during tumor progression. It should be noted that although longitudinal genomic data are currently not available, the large number of patients enabled the use of the concept of a statistical ensemble, that is, averaging large numbers of patients per stage represents a snap shot of the underlying process.

Third, the results of the quantification of the antigenome in CRC showed that the number and expression levels of cancer-germline antigens were similar across tumors with different genomic instabilities, mutational loads, epigenetic instabilities, or TIL enrichments, whereas the neo-antigen landscape was highly diverse. It is intriguing to speculate that the T-cell responses might be primarily directed against neo-antigens rather than against cancer-germline antigens. It is noteworthy that the quality of the T-cell responses directed against neo-antigens and cancer-germline antigens has not been compared so far and future experimental studies are needed in order to validate our observation. Such studies would require isolation of neo-antigen and cancer-germline antigen-specific T cells from patient material and experiments with autologous tumors.

Finally, one striking observation was how the genetic basis of the tumors determines the tumor escape mechanisms. As expected, hypermutated tumors (MSI-H and MSS^ tumors) showed high intratumor heterogeneity, suggesting that the greater mutational load in these tumors results in a higher load of neo-antigens, and promotes T-cell activation and infiltration [41]. These tumors were characterized by depleted subpopulations of immunosuppressive cells and upregulation of immunoinhibitory molecules. Interestingly, similar effective immune responses were observed also in non-hypermutated tumors, but only in the group of homogeneous tumors. The inverse association of the tumor heterogeneity and the immune responses (and hence, distinct tumor escape mechanisms) was evident at several levels: enrichment of immunosuppressive cells, expression of immunostimulators, and expression of MHC I and MHC II molecules. One possible explanation could be that the immune system is able to efficiently sculpt the clonal architecture (immunoediting [42]) only of developing non-hypermutated tumors. However, the available data do not allow deciphering the cause and the consequence of this complex tumor-immune cell interaction, and further studies will be necessary to identify the mechanisms driving this cancer immunoediting in tumors with different mutational phenotypes.

Beyond these biological insights, the results from this study also have important implications for clinical translations.

### Implications for cancer immunotherapies

The results of our systematic analyses of genomic data have several critical implications for cancer immunotherapies in CRC. Most importantly, we propose the use of two genetic features, that is, mutational load and tumor heterogeneity, to stratify the patients for targeted immunotherapy. In our analyses we used whole-exome NGS and SNP array data to estimate the mutational load and the tumor heterogeneity. A new algorithm [43] allows the quantification of tumor heterogeneity solely from whole-exome NGS data and might pave the way for clinical application. Since many clinical centers are routinely collecting whole-exome NGS data, our approach can be used to analyze retrospective cohorts and initiate prospective studies on cancer immunotherapy of CRC and other solid tumors.

The set of major determinants of tumor immunogenicity in CRC we identified included not only immunosuppressive cells and chemokine receptors but also molecules which are currently tested in clinical trials: B7-2 (ligand of CTLA4) and PD-L2 (ligand of PD-1). It will be important to identify predictive markers for these therapies in order to select the patients most likely to respond to therapy. For example, preliminary evidence for a correlation between PD-L1 expression on tumor cells and the likelihood of response to anti-PD-1 therapy was reported in a patient with CRC [44]. Thus, the use of the analytical strategy presented here has the potential to pinpoint predictive markers in patients on immunotherapy.

In addition, our integrative analyses led to the identification of two novel targets for therapy: BCMA and CCR8. BCMA was identified using a network reconstruction approach and validated in 11 independent cohorts. The function of this receptor is well characterized and both agonists and antagonists are available to study the effects on tumor progression. CCR8 was identified using a feature reduction method and the prognostic power was validated using independent cohorts. The function of the CCR8-CCL1 axis is less clear. In melanoma CCR8-CCL1 controls the egress of tumor cells from the afferent lymphatics into the lymph node [38]. Here we show in an orthotopic mouse model of CRC that CCR8 is not expressed in tumors from RAG1-/- mice, indicating that the expression is limited to immune cells. Hence, it appears that the function of CCR8-CCL1 is different in endothelial tumors. Further experimental studies are required to identify the role of CCR8 in CRC.

### Implications for cancer vaccination

The quantification of the antigenome and both antigen classes, cancer-germline antigens and neo-antigens, of CRC has major clinical consequences. The sparsity of the neo-antigen space suggests that neo-antigens are rarely shared among patients and advocates against the development of off-the-shelf vaccines. Based on the results from our analyses, the size of the peptide library which could be required for vaccination of 50% of the MSS patients would be around 70. Paradoxically, it seems that a patient group with a high mutation rate (MSI-H) is amenable for off-the shelf vaccination: a vaccine with five peptides would cover 50% of these patients. Thus, personalized cancer vaccination strategy is required in which whole-exome NGS is performed to identify somatic mutations, followed by bioinformatics analyses to identify neo-antigens, and synthesis of peptide- or DNA/RNA-based vaccines. Viability of such personalized cancer vaccination strategy was recently demonstrated in clinical studies in melanoma patients [7,45]. These proof-of-principle studies provided evidence that T cells reactive to neo-antigens may play a critical role in tumor regression.

However, patients should be stratified for cancer vaccination based on the evaluation of the intratumoral immune landscape rather than on the molecular phenotype (hypermutated versus non-hypermutated, or MSS versus MSI-H). As shown in this study, comprehensive characterization of both the genomic and intratumoral immune landscapes is possible using whole-exome NGS and RNA sequencing data, and can pave the way towards personalized cancer immunotherapy. Moreover, in order to increase the likelihood that a neo-antigen vaccine will induce T-cell responses and prevent other clones from outgrowing, multi-epitope vaccines seem obligatory. It was recently shown that using this type of multi-epitope vaccine regimen up to 12 peptides can be used [46]. However, the exact number of candidate antigens will require further experimental studies with patient-derived xenograft models. Finally, additional studies will be needed in order to prioritize antigens from a large number of candidates and select the optimal set of antigens.

## Conclusions

The approach we show here to characterize the immunophenotypes and the antigenomes of tumors using NGS data can help to identify mechanisms of tumor progression and disentangle the complex tumor-immune cell interactions not only in CRC but also in other solid cancers. Over and above, it can help to improve therapeutic efficacy, even in the absence of immunotherapy. Understanding the molecular basis of the interactions between cytotoxic chemotherapeutics or targeted anticancer agents and the immune system is essential for the development of optimal therapeutic schemes [47] and in the long run will enable precision oncoimmunology and result in clinical benefit for the patients.

## Materials and methods

### Identification of immune-related genes

In order to build a compendium of immune genes related to specific immune cell subpopulations, we collected expression profiles for different immune cell types from 36 studies from the Gene Expression Omnibus [48] and Array Express [49]. The list of datasets is provided in Table S1 in Additional file 2. Additionally, we included expression profiles from normal mucosa [50] and CRC cell lines [13,51] as out-groups. A total of 813 Affymetrix HG-U133A microarrays were then normalized using the frozen robust multiarray analysis (fRMA) [52]. Transcripts with significant differential expression across cell subtypes were identified using ANOVA (*P* < 0.05). For the remaining genes, we ranked median mRNA expression levels within each immune cell type from highest to lowest. A two-fold change cutoff from the highest expression group to the next highest expression cell type was considered as a criterion for cell type-specific expression. For each gene in each cell type, we fixed the median expression value of the highest group and permuted group data and then calculated differential expression in the real data set compared with the permutation sets (10,000 permutations, *P* < 0.05). The specificity of gene expression relied on the Jensen-Shannon divergence, which is an entropy-based measure that quantifies the similarity between a gene expression pattern and another predefined pattern that represented an extreme case in which a gene is expressed in only one tissue [53]. Due to the lack of expression data, candidate genes for MDSCs were selected from the literature. The so-defined immune-related gene expression signatures comprised 1,980 genes. For the identification of subsets of genes representative for specific immune cell types, we applied a strategy used in our previous study [54] and in another published work [55]: we selected genes with an average correlation r ≥ 0.6 (*P* < 0.01) between all specific immune genes in the same cell type [56]. This threshold was chosen to satisfy two goals: selection of genes with relatively high correlation such that their correlation could not be considered a chance event; and selection of a reasonable number of genes suitable for gene set enrichment analysis (GSEA) [57] (at least 11 genes per subpopulation). Furthermore, using a set of genes instead of individual markers for specific TILs ensures robust estimation and is less susceptible to noise arising from the expression of the metagenes in tumor or stromal cells. The immune metagenes (812) are listed in Table S2 in Additional file 2.

### Genomic and clinical data

TCGA Data Portal was queried for clinical information (n = 610), somatic mutations (n = 522), germline mutations (n = 468), methylation data (n = 582), SNP arrays (n = 573), and RNA-seq expression profiles (n = 598) of colon and rectum adenocarcinoma patients (last update July 2014). Raw sequencing data and alignments were downloaded from the Cancer Genomics Hub (cgHub) repository (September 2013). Details on data generation are available in the original study [10]. A total of 222,169 somatic mutations were screened for potential mutated epitopes in 522 patients. The mutations were collected from TCGA portal (n = 386 in the original publication) and additional experimentally validated somatic mutations (n = 136) were used. The latter were lifted over from the hg18 to hg19 genome version prior to merging. To select for germline mutations, all protected mutations were downloaded from TCGA portal. There were hg19-mapped mutations for 385 tumors, 86 of which were excluded, because only the somatic mutations were analyzed. Additional mutations located on the hg18 genome (n = 169) were lifted over to hg19 prior to merging, summing up to 468 patients. The normalized gene expression results analyzed according to the RNASeqV2 protocol were obtained from TCGA portal. Clinical data and microarray expression profiles for validation were collated from 11 studies (Gene Expression Omnibus accession numbers GSE12945, GSE41258, GSE14333, GSE17538, GSE29623, GSE33113, GSE39582, GSE24549, GSE24550, GSE30378, GSE28722).

### Identification of tumor-infiltrating lymphocyte subpopulations

We used GSEA [57] to identify immune cell types that are over-represented in the tumor microenvironment. The expression levels of each gene were z-score normalized across all patients. For each patient (or group of patients) genes were then ranked in descending order according to their z-scores (mean of z-scores). The association was represented by a normalized enrichment score (NES). An immune cell type was considered enriched in a patient or group of patients when the false discovery rate (q-value) was ≤10%. The tumors were grouped into seven classes of molecular phenotypes based on their mutation rates, and CIMP and MSI status. Each phenotype class of patients was compared with the remaining patients to test if there is a nonrandom association between the phenotype classes and enrichment of an immune cell type (Fisher exact test). The resulting log-transformed odds ratio was used to cluster the phenotype classes and the immune cell types (two-dimensional hierarchical clustering, Euclidean distance, average linkage). Clustering and visualization were done with the software Genesis [58]. The relative number of immune cells, *I*_*c*_, which is proportional to the absolute number of cells of a specific immune cell type *c* in a heterogeneous tumor sample, was estimated by calculating in each cell type:

$$ {I}_c={\displaystyle \sum_{i=1}^{n_c}}\frac{lo{g}_{10}\left({x}_i^c+1\right)}{w_i^c}, $$

where *n*_*c*_ is the number of immune metagenes per cell type *c*, ${x}_i^c$ are the normalized counts per gene *i* (TPM), and ${w}_i^c$ is the weight defined as median log2-intensity of gene *i* in cell type c from microarray expression data, which was used to identify immune related genes.

### Characterization of colorectal cancer cancer-germline antigens

The CTDatabase [59] was queried for tumor-specific antigens: 92 localized on chromosome X and 124 in the remaining chromosomes. The genes that showed downregulation in the tumor samples compared with normal tissue (n = 50) were removed (both from TCGA and Genentech cohort, statistical significance by at least one of the EBSeq and DESeq analyses). Using the normal tissue samples, a baseline for cancer-germline antigen expression was set as three standard deviations from the median normal expression [60]. Positive expression is considered above the baseline. Additionally, zero standard deviation was excluded, resulting with 178 cancer-germline antigens. K-means clustering was applied to the RNA-sequencing data for each patient group to identify clusters of highly expressed cancer-germline antigens.

### Characterization of the molecular phenotypes from TCGA data

Molecular phenotypes were defined using three features: MSI status, mutation rates, and methylation subtypes. For all three molecular phenotypes we used the most recent update of the raw sequencing TCGA data set. The MSI status was experimentally determined and provided with the clinical dataset (n = 590). The tumors assigned with MSI-low status (n = 93) were subsumed to the MSS group (n = 427). In the original publication, mutation rates were provided for 224 tumors [10]. Adopting the same approach, mutation rates were then calculated for a total of 470 patients using raw exome-sequencing data and MSI status as available by the time of query. To define the hypermutability of the tumors, we calculated the non-silent mutation rate and the number of nonsynonymous coding mutations per 1 million bases and normalized by the sequencing coverage of the experiment. The mutation rate threshold used to separate the hypermutated and non-hypermutated tumors was 8.24 (as reported in [10]). One tumor was excluded, since it was classified as non-hypermutated with high microsatellite instability, which could be explained by potential low purity of the sample. Among the 18 MSS^, 3 were MSI-low, and 15 were MSS. Methylation subtypes were calculated according to the originally reported workflow: unsupervised hierarchical clustering (recursively partitioned mixture model) of the highest deviating DNA methylation measurements [10]. A total of 582 tumors were classified into three groups: CIMP-high, CIMP-low and CIMP-negative.

### Characterization of colorectal cancer neo-antigens

From the 222,169 somatic mutations, we selected mutations that reside within exons and do not span exon borders. The DNA changes were mapped on the protein level. Start codon changes and splicing mutations were excluded. Along the missense, nonstop, frameshift, and non-frameshift mutations, we slid windows with lengths 8 of 11 nucleotides. To estimate the HLA haplotype of each patient, the raw RNA-seq data were analyzed using HLAminer’s method for prediction of HLA class I alleles [61]. The HLA sequences were updated according to the IMGT/HLA public repository [62]. To reduce the ambiguity, we considered the matching normal samples, the Allele Frequency Net Database [63] and the prediction scores. The predicted four-digit HLA class I alleles and the mutated peptides were used as input for netMHCpan [64] to estimate their binding affinities. Both strong and weak binding peptides were analyzed for the selection of neo-antigens (prediction rank ≤2). The selected neo-antigens were then compared with human protein sequences and the matching peptides were distinguished from the novel peptides.

Epitope databases were screened in order to evaluate the predicted neo-antigens (last update Dec 2014). Deposited peptidic epitopes, T-cell assays and MHC ligand assays were downloaded from the Immune Epitope Database [65], Dana Farber repository [66], and CIG-DB [67]. Automatized HTTP-mediated download and HTML processing retrieved all records from the following databases: MHCBN [68], PeptideDatabase [69], SYFPEITHI [70], and TANTIGEN [71]. The entries were filtered to select for HLA class I-binding epitopes. The total number of reported peptide-MHC affinities (55,000) was five times smaller than the predicted CRC antigenome, and therefore only a small fraction of the MHC-peptide combinations were experimentally covered. Only 15 peptide-HLA allele or peptide-HLA supertype combinations matched the databases and 13 of them were experimentally confirmed (exact peptide-MHC allele matches: VVGAVGVGK and HLA-A*11:01, CLLDILDTAGL and HLA-A*02:01, and LLGRNSFEVCV and HLA-A*02:01).

### Estimation of tumor heterogeneity

The ABSOLUTE algorithm [21] was used to integrate the copy number data together with the somatic mutations in order to estimate the purity and ploidy, and measure the fraction of cancer cells per mutation. The SNP data were downloaded from TCGA portal and analyzed with HAPSEG [72]. The cancer cell fractions were also estimated using another analysis workflow based on PyClone [22]. Matched tumor and normal arrays were analyzed together to determine the parent specific DNA copy numbers [73]. Finally, PyClone v0.12.7 was run with default parameters. The tumor heterogeneity was then estimated as the area under the curve of the cumulative density function from all cancer cell fractions per tumor. The clonal architecture of the tumors was compared in a pairwise manner. A similarity measure was defined as the Kolmogorov-Smirnov D statistic of the cancer cell fraction distributions. Based on this measure, four clonal groups were defined by hierarchical clustering with Euclidean measure and the Ward agglomeration method.

### Reconstruction of the tumor-infiltrating lymphocyte interaction network

Based on the gene expression data we reconstructed an immune cell-gene (immunostimulatory/immunoinhibitory molecules) network. The edge weights of the network are based on the Pearson correlation coefficient between the relative number of immune cells and the expression of the immunomodulatory molecules (r ≥ 0.6, *P* < 0.05). Patients were dichotomized based on the median relative number of immune cells and the gene expression of immunomodulator molecules based on optimal cutoff method, respectively. The nodes of the network were colored according to the hazard ratio for OS and marked with a blue border if genes or cell types were significantly (log-rank) associated with OS. The size of the immune cell nodes was encoded by -log_10_(log rank *P*-value). The network was visualized using Cytoscape [74].

### Identification of determinants of tumor immunogenicity

A linear regression model with 221 parameters including the neo-antigen frequencies, immunomodulatory factors, classical and non-classical HLA class I molecule, chemokines, cytokines, immunosuppressive TILs, and cancer-germline antigens was applied to predict the presence of cytotoxic cells (as an approximation the average of gene expression levels of the CD8, NK, and Tgd metagenes were used). The number of features included in the linear model was reduced by lasso regularization [33]. Only variables with highly significant coefficients (adjusted *P* < 0.005) were included in the final model. The model performance was then evaluated by a 10-fold cross-validation procedure.

### Statistical analyses

To test for differential expression across two (tumor and normal) or more groups (adaptive clusters), we used the EBSeq [75] and DESeq [76] R packages. The number of iterations for EBSeq was adjusted to meet the convergence criteria. The number of subclonal mutations was compared between two or more patient groups using the Wilcoxon, Mann-Whitney and the Kruskal-Wallis tests. For multiple comparisons of expression values, neo-antigen frequencies, and tumor heterogeneity among patient groups, ANOVA and Tukey *post hoc* tests were used. Normality of the distributions was tested with Shapiro-Wilk test. The information on all follow-ups for each patient was extracted from the clinical XML files in the complete clinical set. The overall survival time was defined using the latest information. For survival analysis, the patients were dichotomized based on gene expression levels. The optimal cutpoints were searched within the inner 80% selection interval and chosen based on a minimal corrected *P*-value as proposed by Altman *et al*. [77] and based on a maximum Harrell's C indices [78]. Kaplan-Meier estimator of survival was used to visualize the survival curves. To test whether the number of immunogenic mutations (frameshift or missense) correlates with patient survival, we defined the patients based on the median value after excluding the outer 20%. Hazard ratio and the logrank test were used to compare disease-free and OS between patients in different groups. OS of the patient groups with different immune cell infiltrations were additionally analyzed using univariate and multivariate cox regression including MSI status, hypermutation, methylation subtype, and tumor site (left and right sided) as binary covariates. All analyses were performed using the statistical software environment R (package *survival*).

### Mouse model

All animal experiments were performed according to national guidelines and approved by the government of Middle Franconia, Germany. C57Bl/6 wild-type and Rag1-/- mice were kept on a 12-h light/12-h dark cycle and housed according to institutional guidelines. Endoscopic injection of murine MC38 colon tumor cells in the submucosa of the colon of the wild-type and RAG1-/- mice: the needle was positioned inside the working channel of the endoscope so that there would be no damage to the colonic mucosa. After insertion of the endoscope, the tip of the needle was carefully inserted through the mucosa into the submucosa, and a low number of cells (10^4^ to 10^5^) were injected into the submucosa. During subsequent weeks, tumor growth was analyzed via colon endoscopy. After 2 to 4 weeks, the mice were sacrificed and the tumors excised.

### Quantitative real-time PCR

Total RNA was isolated from excised tumors (see mouse model above) using Trizol™ (Life Technologies, Carlsbad, CA, USA) and reverse transcribed using SuperScript III and Random Priming (Life Technologies). SYBR green-based quantitative PCR was performed on a Viia 7 Lightcycler (Life Technologies) with the following primers: *TfIIb*_forward, 5’-GTCACATGTCCGAATCATCCA; *TfIIb*_reverse, 5’-TCAATAACTCGGTCCCCTACAA; *Uxt*_forward, 5’-CTCACAGAGCTCAGCGACAGC; *Uxt*_reverse, 5’-AAATTCTGCAGGCCTTGTAGTTCTC; *Ccr8*_forward, 5’-CTTGTTTGTGCTGGGCCTTC; *Ccr8*_reverse, 5’-GGCCAGAGACCACCTTACAC; *Ccl1*_forward, 5’-GGCTGCCGTGTGGATACAG; *Ccl1*_reverse, 5’-AGGTGATTTTGAACCCACGTTT. *Ccl1* and *Ccr8* values were normalized against *TfIIb* and *Uxt*.

## Additional files

Additional file 1: Figure S1. Analytical pipelines used for the analyses of heterogeneous genomic data sets. **Figure S2.** Heatmap of the expression of genes related to specific immune cell subpopulations from microarray studies. **Figure S3.** Survival analysis for major TIL subpopulations. **Figure S4.** Number of mutations (a) and enrichment of TILs in MSS tumors from stage I to stage IV (b). **Figure S5.** Expression of specific markers for selected immune cell subpopulations from stage I to stage IV. **Figure S6.** Neo-antigens shared in more than seven patients and the corresponding mutated genes. **Figure S7.** Hierarchical clustering of the cancer cell fraction in MSS patients. **Figure S8.** Expression of immunomodulatory molecules in hypermutated and non-hypermutated tumors. **Figure S9.** Forrest plots for the expression of significant T-cell immunomodulatory molecules in the TCGA cohort and in the validation cohorts. **Figure S10.** Expression of CCR8 and CCL1 in human CRC samples. Left panel: TCGA data. Right panel: quantitative real-time PCR data from [35] normalized to 18S rRNA. Additional file 2: Table S1. Accession numbers of the microarray profiles used to derive the compendium of immune genes. **Table S2.** List of immune metagenes. **Table S3.** Survival analysis using univariate and multivariate Cox regression for patient groups with different immune cell infiltrations including molecular phenotypes as covariates. **Table S4.** List of predicted neo-antigens shared in at least two patients. **Table S5.** Accession numbers of the microarray profiles of the validation datasets. **Table S6.** List of features used for the regression model.

## Abbreviations

CIMP: CpG island methylation
CRC: colorectal cancer
DC: dendritic cell
DFS: disease-free survival
GSEA: gene set enrichment analysis
HLA: human leukocyte antigen
MDSC: myeloid-derived suppressor cell
MHC: major histocompatibility
MSI: microsatellite instable
MSS: microsatellite stable
NGS: next-generation sequencing
NK: natural killer
NKT: natural killer T cell
OS: overall survival
SNP: single-nucleotide polymorphism
TCGA: The Cancer Genome Atlas
Tfh: follicular helper T cell
Tgd: gamma delta T cell
Th: T-helper
TIL: tumor-infiltrating lymphocyte
Treg: regulatory T cell
