# Supplementary material for: Characterization of the immunophenotypes and antigenomes of colorectal cancers reveals distinct tumor escape mechanisms and novel targets for immunotherapy
Source: Genome Biol. 2015 Mar 31;16(1):64. doi: 10.1186/s13059-015-0620-6 (PMC4377852; doi:10.1186/s13059-015-0620-6)
Supplement: Additional file 1: Figure S1. — Analytical pipelines used for the analyses of heterogeneous genomic data sets. Figure S2. Heatmap of the expression of genes related to specific immune cell subpopulations from microarray studies. Figure S3. Survival analysis for major TIL subpopulations. Figure S4. Number of mutations (a) and enrichment of TILs in MSS tumors from stage I to stage IV (b). Figure S5. Expression of specific markers for selected immune cell subpopulations from stage I to stage IV. Figure S6. Neo-antigens shared in more than seven patients and the corresponding mutated genes. Figure S7. Hierarchical clustering of the cancer cell fraction in MSS patients. Figure S8. Expression of immunomodulatory molecules in hypermutated and non-hypermutated tumors. Figure S9. Forrest plots for the expression of significant T-cell immunomodulatory molecules in the TCGA cohort and in the validation cohorts. Figure S10. Expression of CCR8 and CCL1 in human CRC samples. Left panel: TCGA data. Right panel: quantitative real-time PCR data from [35] normalized to 18S rRNA. [file 13059_2015_620_MOESM1_ESM.pdf]

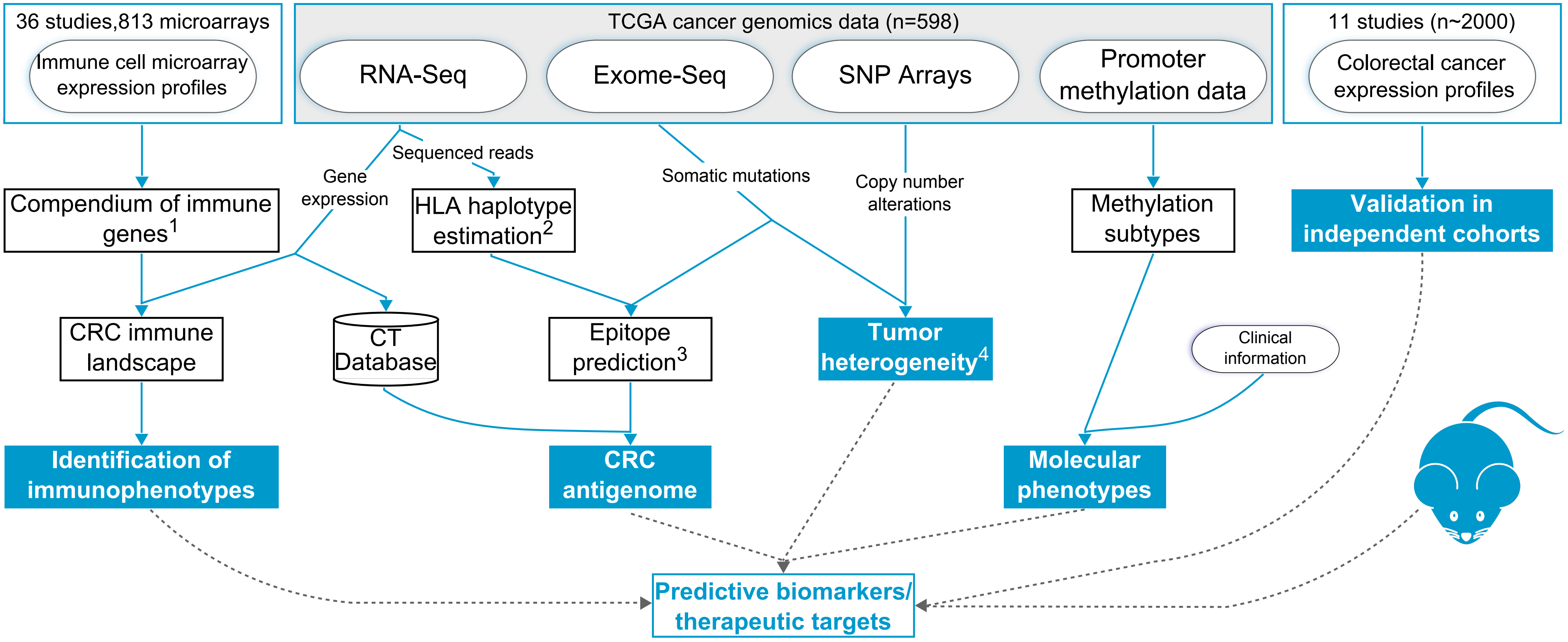

Tools/Methods

1. Bindea G et al. *Immunity* 2013; 39(4):782-95
2. Warren RL et al. *Genome Medicine* 2012; 4:95
3. Nielsen M et al. *PLoS ONE* 2007; 2: e796
4. Carter SL et al. *Nat Biotech* 2012; 30: 413-421

Figure S1

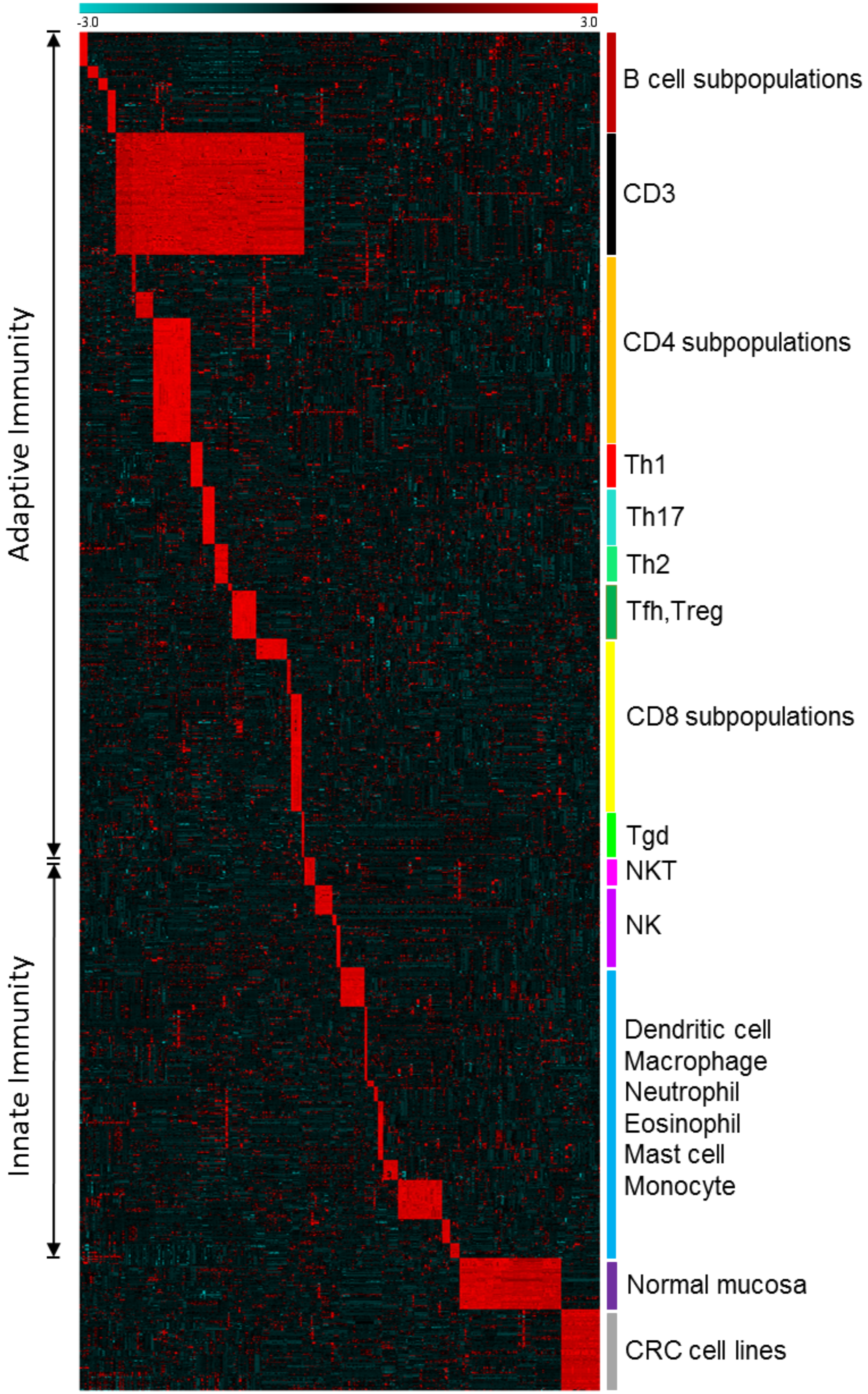

-3.0
3.0
Adaptive Immunity
Innate Immunity
B cell subpopulations
CD3
CD4 subpopulations
Th1
Th17
Th2
Tfh,Treg
CD8 subpopulations
Tgd
NKT
NK
Dendritic cell
Macrophage
Neutrophil
Eosinophil
Mast cell
Monocyte
Normal mucosa
CRC cell lines

Figure S2

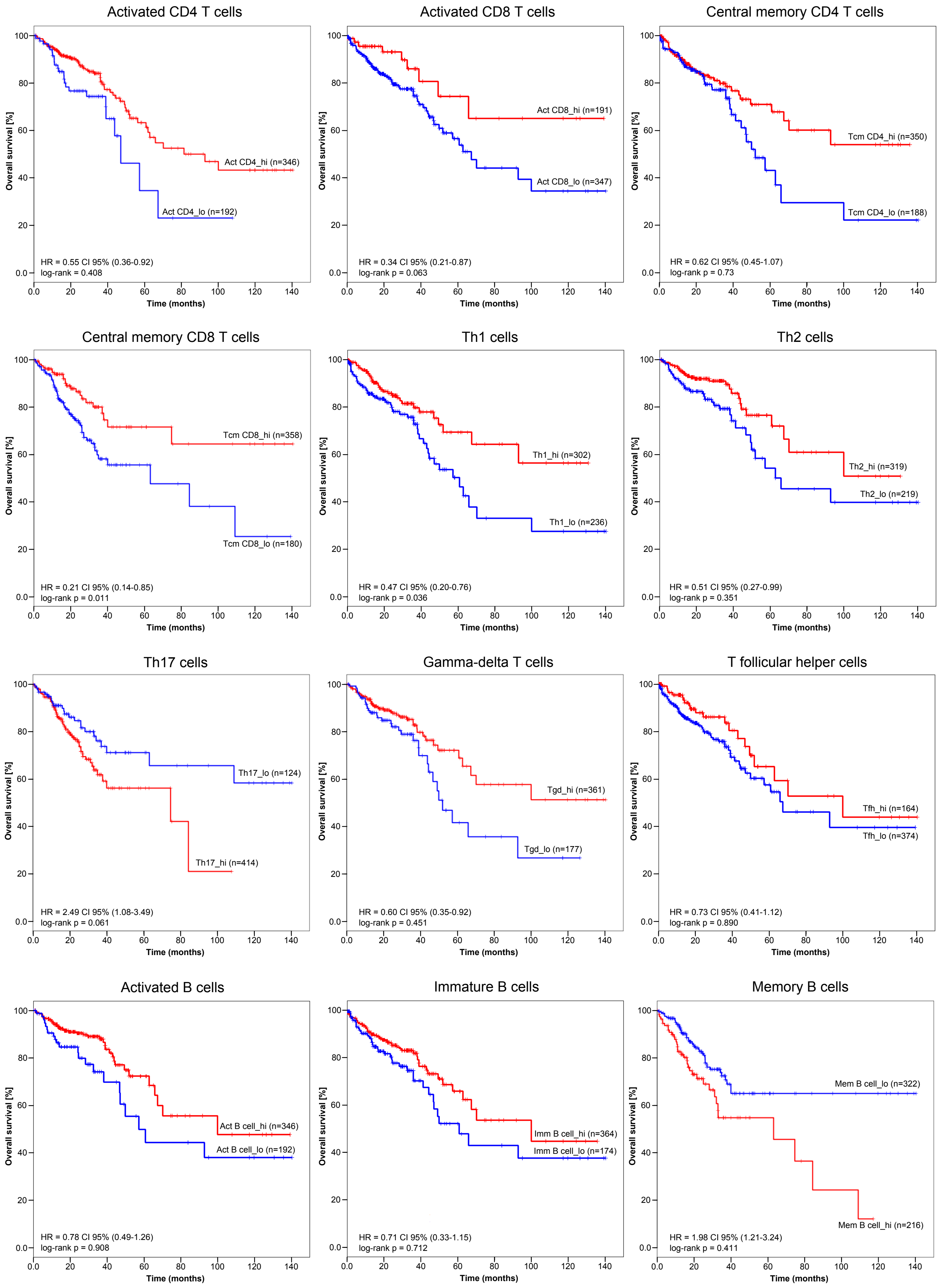

Activated CD4 T cells
Act CD4_hi (n=346)
Act CD4_lo (n=192)
HR = 0.55 CI 95% (0.36-0.92)
log-rank = 0.408
Activated CD8 T cells
Act CD8_hi (n=191)
Act CD8_lo (n=347)
HR = 0.34 CI 95% (0.21-0.87)
log-rank p = 0.063
Central memory CD4 T cells
Tcm CD4_hi (n=350)
Tcm CD4_lo (n=188)
HR = 0.62 CI 95% (0.45-1.07)
log-rank p = 0.73
Central memory CD8 T cells
Tcm CD8_hi (n=358)
Tcm CD8_lo (n=180)
HR = 0.21 CI 95% (0.14-0.85)
log-rank p = 0.011
Th1 cells
Th1_hi (n=302)
Th1_lo (n=236)
HR = 0.47 CI 95% (0.20-0.76)
log-rank p = 0.036
Th2 cells
Th2_hi (n=319)
Th2_lo (n=219)
HR = 0.51 CI 95% (0.27-0.99)
log-rank p = 0.351
Th17 cells
Th17_lo (n=124)
Th17_hi (n=414)
HR = 2.49 CI 95% (1.08-3.49)
log-rank p = 0.061
Gamma-delta T cells
Tgd_hi (n=361)
Tgd_lo (n=177)
HR = 0.60 CI 95% (0.35-0.92)
log-rank p = 0.451
T follicular helper cells
Tfh_hi (n=164)
Tfh_lo (n=374)
HR = 0.73 CI 95% (0.41-1.12)
log-rank p = 0.890
Activated B cells
Act B cell_hi (n=346)
Act B cell_lo (n=192)
HR = 0.78 CI 95% (0.49-1.26)
log-rank p = 0.908
Immature B cells
Imm B cell_hi (n=364)
Imm B cell_lo (n=174)
HR = 0.71 CI 95% (0.33-1.15)
log-rank p = 0.712
Memory B cells
Mem B cell_lo (n=322)
Mem B cell_hi (n=216)
HR = 1.98 CI 95% (1.21-3.24)
log-rank p = 0.411
Overall survival [%]
Time (months)

Figure S3a

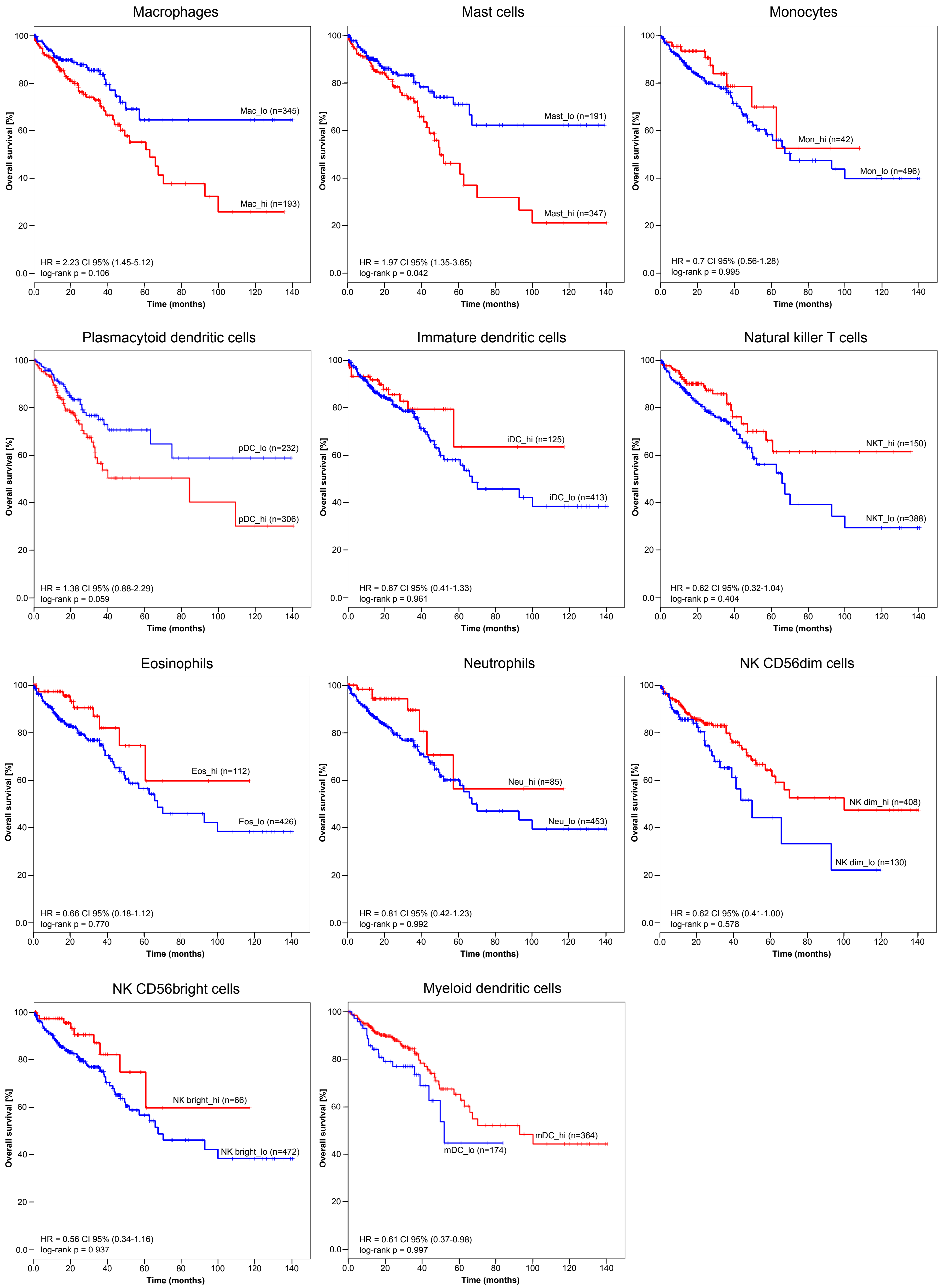

Macrophages
Mac_lo (n=345)
Mac_hi (n=193)
HR = 2.23 CI 95% (1.45-5.12)
log-rank p = 0.106
Mast cells
Mast_lo (n=191)
Mast_hi (n=347)
HR = 1.97 CI 95% (1.35-3.65)
log-rank p = 0.042
Monocytes
Mon_hi (n=42)
Mon_lo (n=496)
HR = 0.7 CI 95% (0.56-1.28)
log-rank p = 0.995
Plasmacytoid dendritic cells
pDC_lo (n=232)
pDC_hi (n=306)
HR = 1.38 CI 95% (0.88-2.29)
log-rank p = 0.059
Immature dendritic cells
iDC_hi (n=125)
iDC_lo (n=413)
HR = 0.87 CI 95% (0.41-1.33)
log-rank p = 0.961
Natural killer T cells
NKT_hi (n=150)
NKT_lo (n=388)
HR = 0.62 CI 95% (0.32-1.04)
log-rank p = 0.404
Eosinophils
Eos_hi (n=112)
Eos_lo (n=426)
HR = 0.66 CI 95% (0.18-1.12)
log-rank p = 0.770
Neutrophils
Neu_hi (n=85)
Neu_lo (n=453)
HR = 0.81 CI 95% (0.42-1.23)
log-rank p = 0.992
NK CD56dim cells
NK dim_hi (n=408)
NK dim_lo (n=130)
HR = 0.62 CI 95% (0.41-1.00)
log-rank p = 0.578
NK CD56bright cells
NK bright_hi (n=66)
NK bright_lo (n=472)
HR = 0.56 CI 95% (0.34-1.16)
log-rank p = 0.937
Myeloid dendritic cells
mDC_hi (n=364)
mDC_lo (n=174)
HR = 0.61 CI 95% (0.37-0.98)
log-rank p = 0.997
Overall survival [%]
Time (months)

Figure S3b

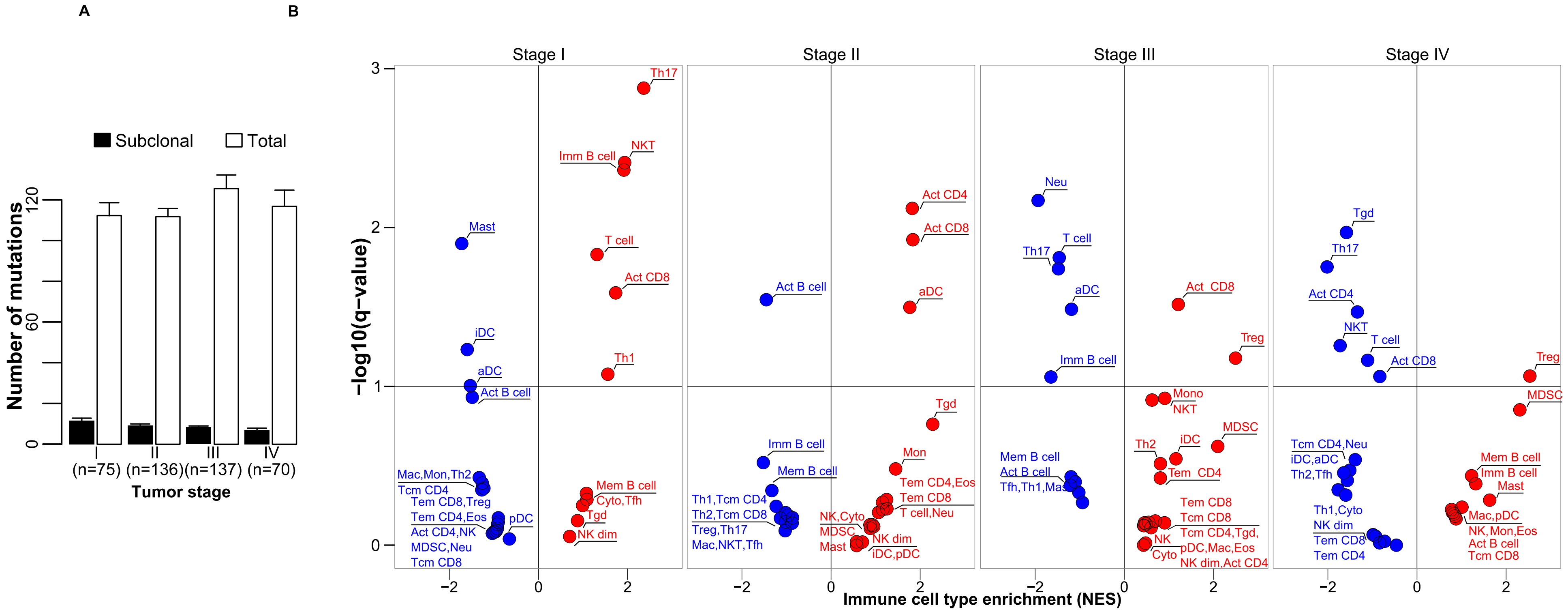

A
B
Subclonal
Total
Number of mutations
Tumor stage
(n=75) (n=136)(n=137) (n=70)
Stage I
Stage II
Stage III
Stage IV
−log10(q−value)
Immune cell type enrichment (NES)

Figure S4

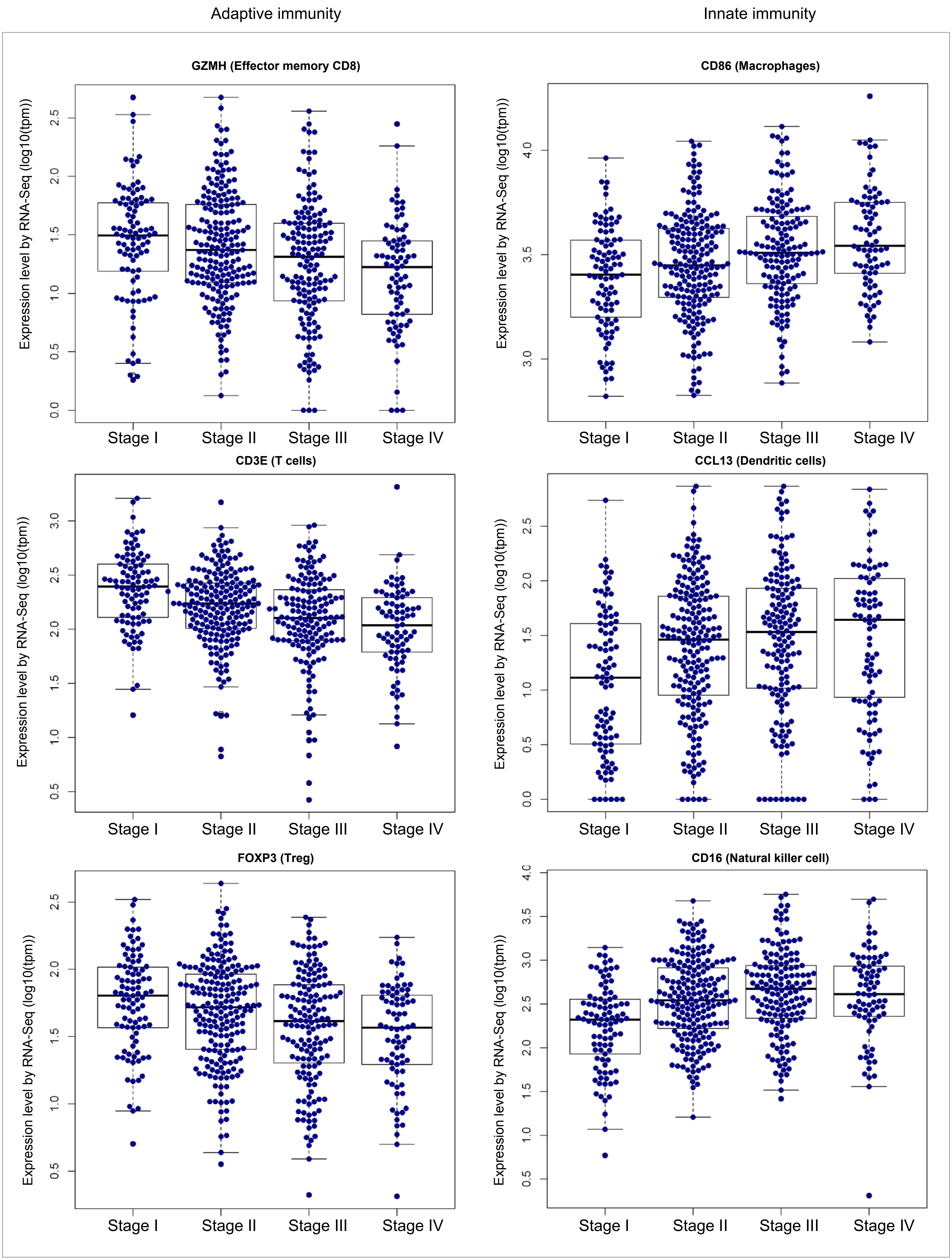

Adaptive immunity
Innate immunity
GZMH (Effector memory CD8)
CD86 (Macrophages)
CD3E (T cells)
CCL13 (Dendritic cells)
FOXP3 (Treg)
CD16 (Natural killer cell)
Expression level by RNA-Seq (log10(tpm))
Stage I
Stage II
Stage III
Stage IV

Figure S5

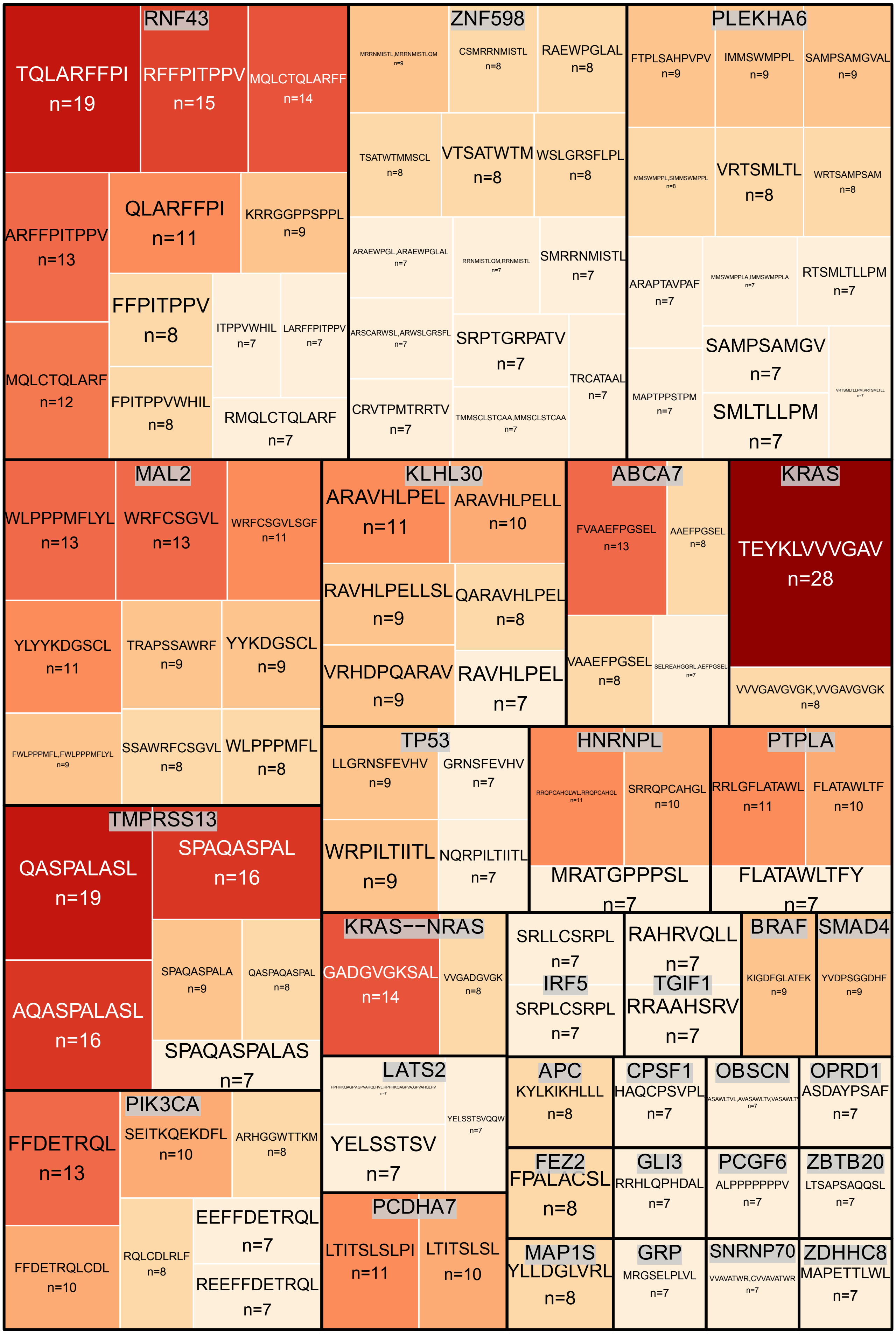

RNF43
TQLARFFPI n=19
RFFPITPPV n=15
MQLCTQLARFF n=14
ARFFPITPPV n=13
QLARFFPI n=11
KRRGGPPSPPL n=9
FFPITPPV n=8
ITPPVWHIL n=7
LARFFPITPPV n=7
MQLCTQLARF n=12
FPITPPVWHIL n=8
RMQLCTQLARF n=7
ZNF598
MRRNMISTL,MRRNMISTLQM n=9
CSMRRNMISTL n=8
RAEWPGLAL n=8
TSATWTMMSCL n=8
VTSATWTM n=8
WSLGRSFLPL n=8
ARAEWPGL,ARAEWPGLAL n=7
RRNMISTLQM,RRNMISTL n=7
SMRRNMISTL n=7
ARSCARWSL,ARWSLGRSFL n=7
SRPTGRPATV n=7
TRCATAAL n=7
CRVTPMTRRTV n=7
TMMSCLSTCAA,MMSCLSTCAA n=7
PLEKHA6
FTPLSAHPVPV n=9
IMMSWMPPL n=9
SAMPSAMGVAL n=9
MMSWMPPL,SIMMSWMPPL n=8
VRTSMLTL n=8
WRTSAMPSAM n=8
ARAPTAVPAF n=7
MMSWMPPLA,IMMSWMPPLA n=7
RTSMLTLLPM n=7
SAMPSAMGV n=7
MAPTPPSTPM n=7
SMLTLLPM n=7
VRTSMLTLLPM,VRTSMLTL n=7
MAL2
WLPPPMFLYL n=13
WRFCSGVL n=13
WRFCSGVLSGF n=11
YLYYKDGSCL n=11
TRAPSSAWRF n=9
YYKDGSCL n=9
FWLPPPMFL,FWLPPPMFLYL n=9
SSAWRFCSGVL n=8
WLPPPMFL n=8
KLHL30
ARAVHLPEL n=11
ARAVHLPELL n=10
RAVHLPELLSL n=9
QARAVHLPEL n=8
VRHDPQARAV n=9
RAVHLPEL n=7
ABCA7
FVAAEFPGSEL n=13
AAEFPGSEL n=8
VAAEFPGSEL n=8
SELREAHGGRL,AEFPGSEL n=7
KRAS
TEYKLVVVGAV n=28
VVVGAVGVGK,VVGAVGVGK n=8
TP53
LLGRNSFEVHV n=9
GRNSFEVHV n=7
WRPILTIITL n=9
NQRPILTIITL n=7
HNRNPL
RRQPCAHGLWL,RRQPCAHGL n=11
SRRQPCAHGL n=10
MRATGPPPSL n=7
PTPLA
RRLGFLATAWL n=11
FLATAWLTF n=10
FLATAWLTFY n=7
TMPRSS13
QASPALASL n=19
SPAQASPAL n=16
AQASPALASL n=16
SPAQASPALA n=9
QASPAQASPAL n=8
SPAQASPALAS n=7
KRAS--NRAS
GADGVGKSAL n=14
VVGADGVGK n=8
SRLLCSRPL n=7
IRF5
SRPLCSRPL n=7
RAHRVQLL n=7
TGIF1
RRAAHSRV n=7
BRAF
KIGDFGLATEK n=9
SMAD4
YVDPSGGDHF n=9
LATS2
YELSSTSVQQW n=7
YELSSTSV n=7
APC
KYLKIKHLLL n=8
CPSF1
HAQCPSVPL n=7
OBSCN
ASAWLTVL,AVASAWLTV,VASAWLTV n=7
OPRD1
ASDYPSAF n=7
FEZ2
FPALACSL n=8
GLI3
RRHLQPHDAL n=7
PCGF6
ALPPPPPPPV n=7
ZBTB20
LTSAPSAQQSL n=7
PIK3CA
FFDETRQL n=13
SEITKQEKDFL n=10
ARHGGWTTKM n=8
FFDETRQLCDL n=10
RQLCDLRLF n=8
EEFFDETRQL n=7
REEFFDETRQL n=7
PCDHA7
LTITSLSLPI n=11
LTITSLSL n=10
MAP1S
YLLDGLVRL n=8
GRP
MRGSELPLVL n=7
SNRNP70
VVAVATWR,CVVAVATWR n=7
ZDHHC8
MAPETTLWL n=7

Figure S6

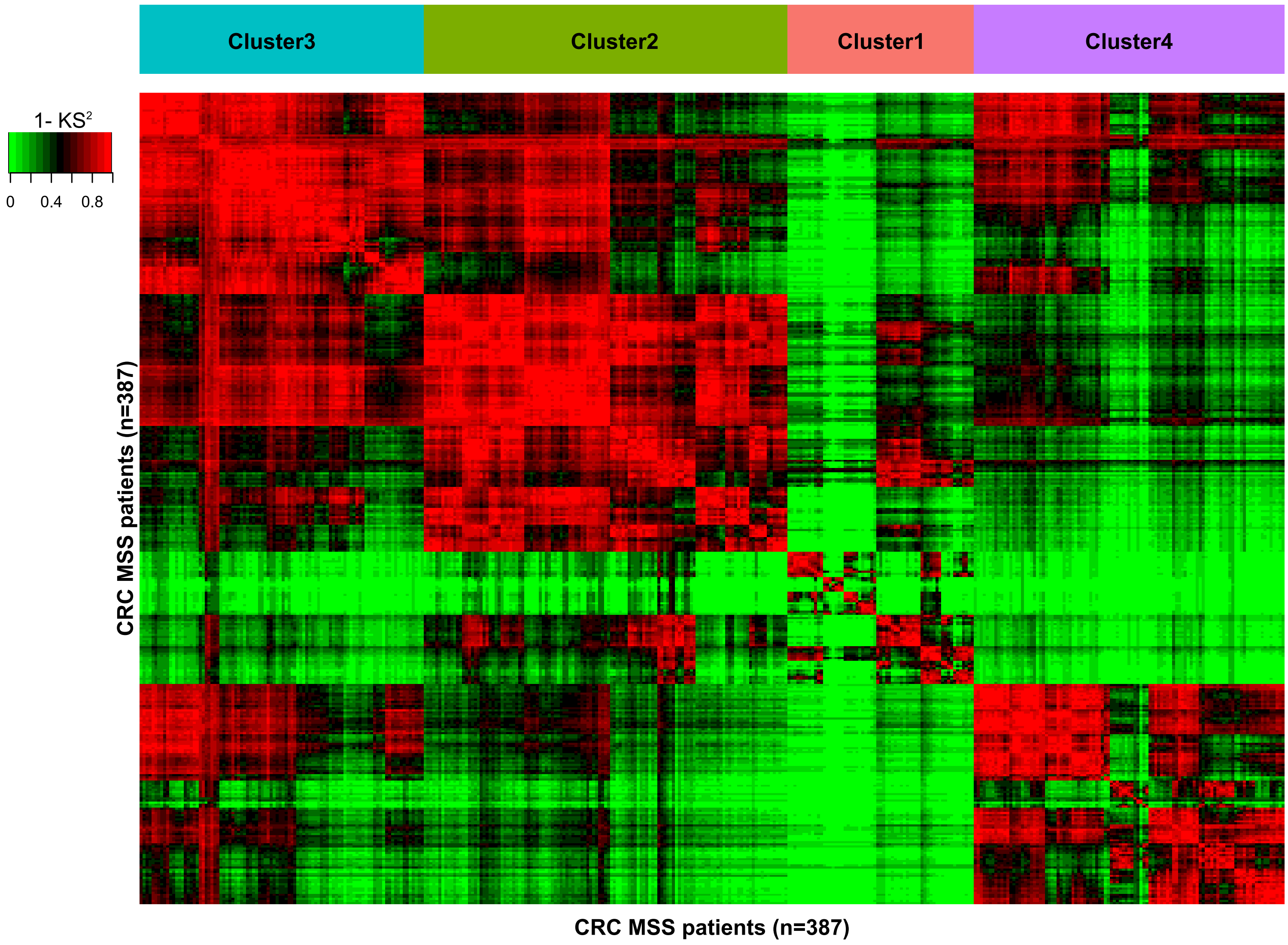
Cluster3
Cluster2
Cluster1
Cluster4
1- KS2
0
0.4
0.8
CRC MSS patients (n=387)
CRC MSS patients (n=387)

Figure S7

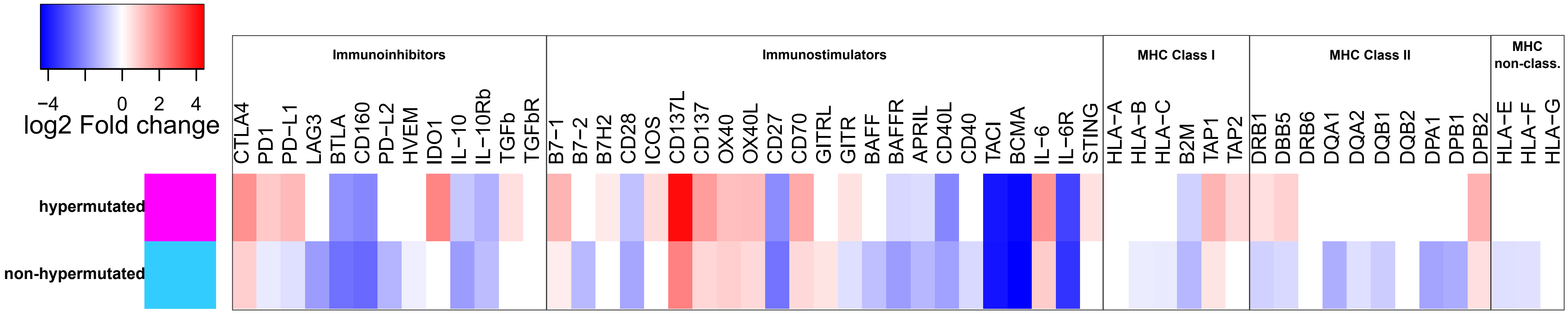
−4
0
2
4
log2 Fold change
Immunoinhibitors
Immunostimulators
MHC Class I
MHC Class II
MHC non-class.
CTLA4
PD1
PD−L1
LAG3
BTLA
CD160
PD−L2
HVEM
IDO1
IL−10
IL−10Rb
TGFb
TGFbR
B7−1
B7−2
B7H2
CD28
ICOS
CD137L
CD137
OX40
OX40L
CD27
CD70
GITRL
GITR
BAFF
BAFFR
APRIL
CD40L
CD40
TACI
BCMA
IL−6
IL−6R
STING
HLA−A
HLA−B
HLA−C
B2M
TAP1
TAP2
DRB1
DBB5
DRB6
DQA1
DQA2
DQB1
DQB2
DPA1
DPB1
DPB2
HLA−E
HLA−F
HLA−G
hypermutated
non-hypermutated

Figure S8

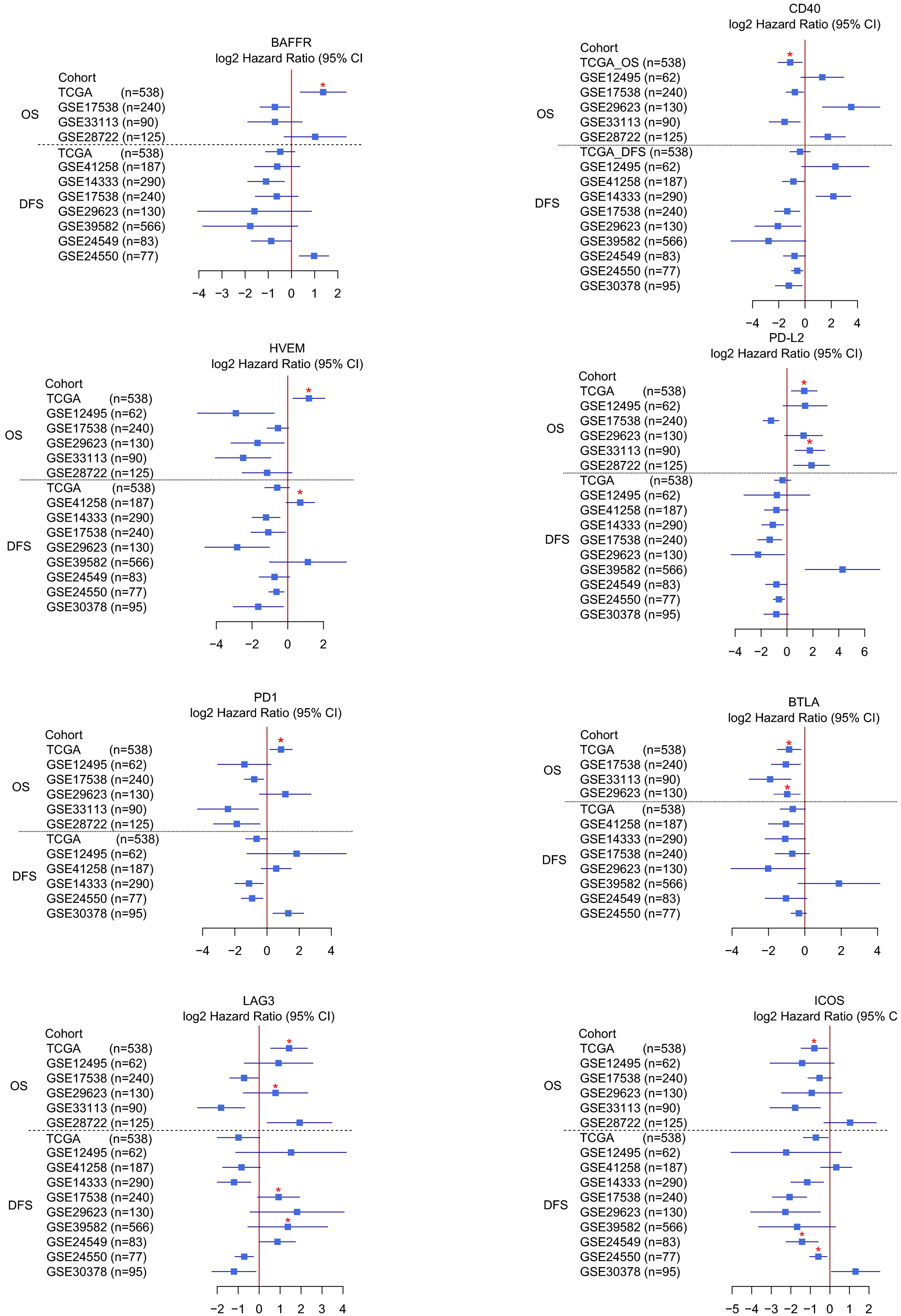

BAFFR
log2 Hazard Ratio (95% CI
Cohort
OS
TCGA (n=538)
GSE17538 (n=240)
GSE33113 (n=90)
GSE28722 (n=125)
DFS
TCGA (n=538)
GSE41258 (n=187)
GSE14333 (n=290)
GSE17538 (n=240)
GSE29623 (n=130)
GSE39582 (n=566)
GSE24549 (n=83)
GSE24550 (n=77)
−4 −3 −2 −1 0 1 2
CD40
log2 Hazard Ratio (95% CI)
Cohort
OS
TCGA_OS (n=538)
GSE12495 (n=62)
GSE17538 (n=240)
GSE29623 (n=130)
GSE33113 (n=90)
GSE28722 (n=125)
DFS
TCGA_DFS (n=538)
GSE12495 (n=62)
GSE41258 (n=187)
GSE14333 (n=290)
GSE17538 (n=240)
GSE29623 (n=130)
GSE39582 (n=566)
GSE24549 (n=83)
GSE24550 (n=77)
GSE30378 (n=95)
−4 −2 0 2 4
HVEM
log2 Hazard Ratio (95% CI)
Cohort
OS
TCGA (n=538)
GSE12495 (n=62)
GSE17538 (n=240)
GSE29623 (n=130)
GSE33113 (n=90)
GSE28722 (n=125)
DFS
TCGA (n=538)
GSE41258 (n=187)
GSE14333 (n=290)
GSE17538 (n=240)
GSE29623 (n=130)
GSE39582 (n=566)
GSE24549 (n=83)
GSE24550 (n=77)
GSE30378 (n=95)
−4 −2 0 2
PD-L2
log2 Hazard Ratio (95% CI)
Cohort
OS
TCGA (n=538)
GSE12495 (n=62)
GSE17538 (n=240)
GSE29623 (n=130)
GSE33113 (n=90)
GSE28722 (n=125)
DFS
TCGA (n=538)
GSE12495 (n=62)
GSE41258 (n=187)
GSE14333 (n=290)
GSE17538 (n=240)
GSE29623 (n=130)
GSE39582 (n=566)
GSE24549 (n=83)
GSE24550 (n=77)
GSE30378 (n=95)
−4 −2 0 2 4 6
PD1
log2 Hazard Ratio (95% CI)
Cohort
OS
TCGA (n=538)
GSE12495 (n=62)
GSE17538 (n=240)
GSE29623 (n=130)
GSE33113 (n=90)
GSE28722 (n=125)
DFS
TCGA (n=538)
GSE12495 (n=62)
GSE41258 (n=187)
GSE14333 (n=290)
GSE24550 (n=77)
GSE30378 (n=95)
−4 −2 0 2 4
BTLA
log2 Hazard Ratio (95% CI)
Cohort
OS
TCGA (n=538)
GSE17538 (n=240)
GSE33113 (n=90)
GSE29623 (n=130)
DFS
TCGA (n=538)
GSE41258 (n=187)
GSE14333 (n=290)
GSE17538 (n=240)
GSE29623 (n=130)
GSE39582 (n=566)
GSE24549 (n=83)
GSE24550 (n=77)
−4 −2 0 2 4
LAG3
log2 Hazard Ratio (95% CI)
Cohort
OS
TCGA (n=538)
GSE12495 (n=62)
GSE17538 (n=240)
GSE29623 (n=130)
GSE33113 (n=90)
GSE28722 (n=125)
DFS
TCGA (n=538)
GSE12495 (n=62)
GSE41258 (n=187)
GSE14333 (n=290)
GSE17538 (n=240)
GSE29623 (n=130)
GSE39582 (n=566)
GSE24549 (n=83)
GSE24550 (n=77)
GSE30378 (n=95)
−2 −1 0 1 2 3 4
ICOS
log2 Hazard Ratio (95% C
Cohort
OS
TCGA (n=538)
GSE12495 (n=62)
GSE17538 (n=240)
GSE29623 (n=130)
GSE33113 (n=90)
GSE28722 (n=125)
DFS
TCGA (n=538)
GSE12495 (n=62)
GSE41258 (n=187)
GSE14333 (n=290)
GSE17538 (n=240)
GSE29623 (n=130)
GSE39582 (n=566)
GSE24549 (n=83)
GSE24550 (n=77)
GSE30378 (n=95)
−5 −4 −3 −2 −1 0 1 2

Figure S9

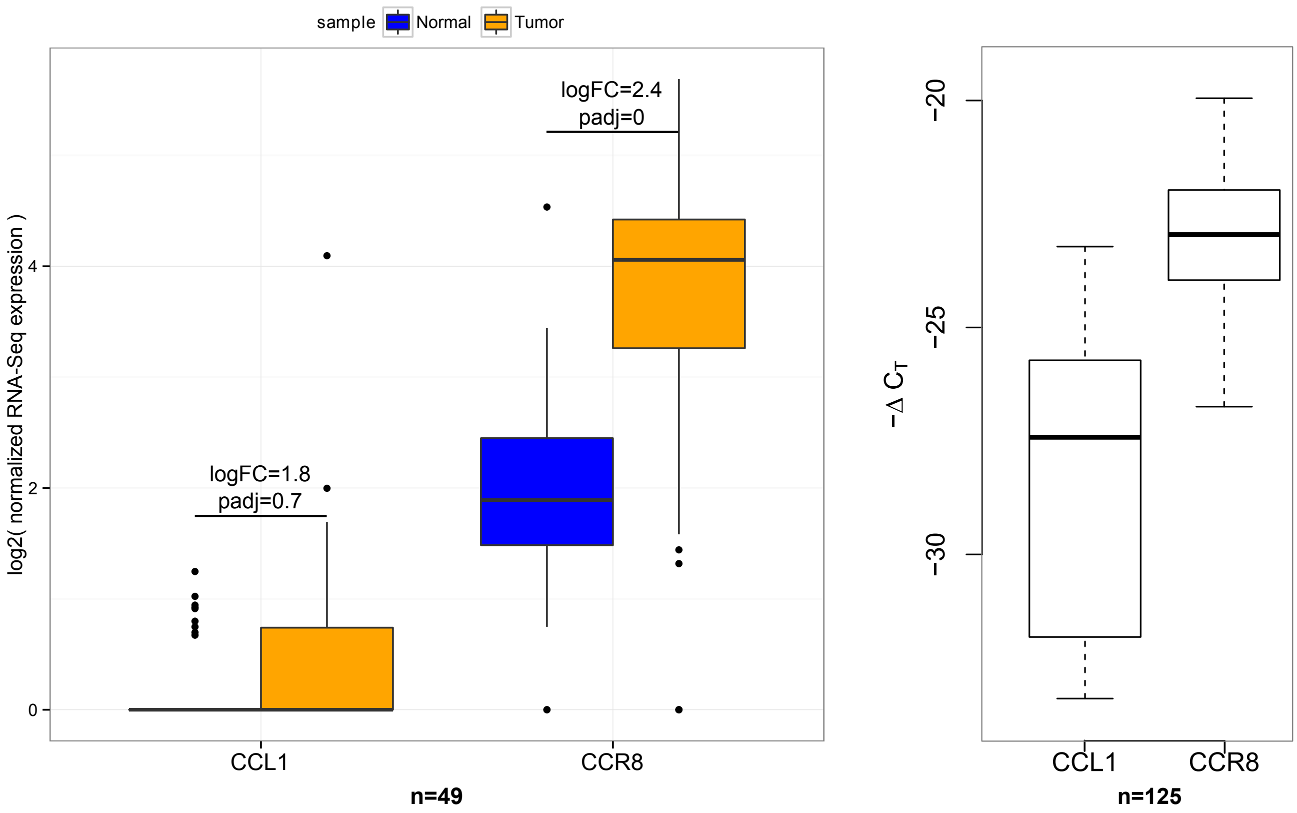
sample
Normal
Tumor
log2(normalized RNA-Seq expression)
logFC=2.4
padj=0
logFC=1.8
padj=0.7
4
2
0
CCL1
CCR8
n=49
−Δ C_T
−20
−25
−30
CCL1
CCR8
n=125

Figure S10
