# Supplementary material for: Characterization of the immunophenotypes and antigenomes of colorectal cancers reveals distinct tumor escape mechanisms and novel targets for immunotherapy
Source: Genome Biol. 2015 Mar 31;16(1):64. doi: 10.1186/s13059-015-0620-6 (PMC4377852; doi:10.1186/s13059-015-0620-6)
Supplement: Additional file 2: Table S1. — Accession numbers of the microarray profiles used to derive the compendium of immune genes. Table S2. List of immune metagenes. Table S3. Survival analysis using univariate and multivariate Cox regression for patient groups with different immune cell infiltrations including molecular phenotypes as covariates. Table S4. List of predicted neo-antigens shared in at least two patients. Table S5. Accession numbers of the microarray profiles of the validation datasets. Table S6. List of features used for the regression model. [file 13059_2015_620_MOESM2_ESM.pdf]

Table S1: Accession numbers of the microarray profiles used to derive the compendium of immune genes

DB source	DATASET ID	Publication (PMID)	Platform (s)	Study description
ArrayExpress	E-MEXP-380	16966385	HG-U133A	Human NK cells were sorted into CD56dim and CD56bright NK cell subpopulations. Gene profiling was performed, in order to define characteristics of both populations.
ArrayExpress	E-MTAB-62	20379172	HG-U133A	The experiment contains systematically annotated and consistently normalized human gene expression data matrix of 5372 samples integrated from 206 public experiments of a HG-U133A array platform, representing 369 different cell and tissue types, disease states and cell lines
ArrayExpress	E-MEXP-384	21680796	HG-U133A	Transcription profiling of human precursor-B-cell differentiation: five subsets representing the main stages of human precursor-B-cell differentiation and CD34+lin- cord blood cells were purified.
ArrayExpress	E-MEXP-243	15746248	HG-U133A	Transcription profiling of human memory T cells stimulated with PhI p-extract in healthy and alergic individuals
ArrayExpress	E-TABM-145	19274049	HG-U133A	The data contains 79 different human tissues and cell types with 2 replicates per tissue/cell type.
ArrayExpress	E-MTAB-57	16919171	HG-U133A	Expression profiling studies on colon cancer comparing tumoral and normal biopsies from a series of patients in order to identify molecular biomarkers.
GEO	GSE22886	15789058	HG-U133A	Expression profiles of twelve different types of human leukocytes from peripherial blood and bone marrow. The gene expression profiles are performed before and after treatment to induce activation and/or differentiation.
GEO	GSE11188	19435912	HG-U133A	Gene-expression profiles of non-tumor-reactive CD8+ T cells
GEO	GSE3982	16339519	HG-U133A	Gene transcription profiles for the major leukocyte types in humans: Different leukocyte subsets were either isolated or differentiated from human blood to obtain consistently >95% pure populations. Gene profiles for activated effector cells such as macrophages, neutrophils and mast cells were also generated.
GEO	GSE22601	15928199	HG-U133A	Gene expression is measured in nine stages of T cell development.

GEO	GSE13017	19453521	HG-U133A	Homo sapiens CD4+CD25- and CD4+CD25hi T cells were sorted from healthy individual donors and analyzed ex vivo (non-activated) or 1 day following T-cell receptor activation using anti-CD3/IL2 and anti-CD3/-CD28/IL2 (activated), respectively.
GEO	GSE1460	15210650	HG-U133A	Subpopulations of human fetal thymocyte and circulating naïve T cells were obtained through FACS sorting.
GEO	GSE4571	16836768	HG-U133A	Exrpression profiles of human Treg cells obtained from peripheral blood by cell sorting and their naive controls.
GEO	GSE24634	21347372	HG-U133A	Expression data from developing regulatory T cells
GEO	GSE36476	22434910	HG-U133A	Gene expression signatures in elderly CD4 T cell responses
GEO	GSE17354	19652199	HG-U133A	Gene expression profiling of CD4+ and CD8+ T-cells from gene therapy treated ADA patients and from healthy controls
GEO	GSE1133	15075390	HG-U133A	Tissue-specific pattern of mRNA expression in 79 human and mouse tissues
GEO	GSE3996	17573725	HG-U133A	Differential gene expression in regulatory T-cells after activation by temporary contact with dendritic cells
GEO	GSE1323	16531451	HG-U133A	Expression profiling of the SW480 and SW620 colon cancer cells lines derived from a primary tumor and a corresponding metastasis from the same individual.
GEO	GSE24514	21544814	HG-U133A	Expression data from human MSI colorectal cancer and normal colonic mucosa
GEO	GSE6740	17251300	HG-U133A	Comparison of transcriptional profiles of CD4+ and CD8+ T cells from HIV-infected pateints and uninfected control group-
GEO	GSE8835	15965501	HG-U133A	CD4 T cells and CD8 T cells were obtained from peripheral blood mononuclear cells of previously untreated patients with CLL and healthy individuals.
GEO	GSE13411	19124732	HG-U133A	Four subsets of human splenic B cells (naïve, IgM-memory, Ig isotype switched memory and plasma cells); sort-purified and analysed immediately ex vivo; performed in duplicate.
GEO	GSE5580	17032758	HG-U133A	Gene expression profiling of circulating total blood leukocytes, T-Cells, and Monocytes in severe trauma patients and healthy subjects.
GEO	GSE6477	17409404	HG-U133A	Expression data from different stages of plasma cell neoplasm.
GEO	GSE4045	16819509	HG-U133A	Expression profile of serrated and conventional colorectal tumors
GEO	GSE2328	15781863	HG-U133A	Application of genome-wide expression analysis to human health and disease

GEO	GSE11430	18578872	HG-U133_Plus_2	Gene expression profiles of human monocytes and monocyte-derived-macrophages.
GEO	GSE2509	16531451	HG-U133A	Two colon cancer cell lines are profiled: SW480 and SW620.
GEO	GSE24759	21241896	U133AAofAv2	38 distinct hematopoietic cell states based on cell surface marker expression, representing hematopoietic stem and progenitor cells, terminally differentiated cells, and intermediate states are defined.
GEO	GSE41258	19359472	HG-U133A	390 expression arrays from colorectal cancer patients
GEO	GSE28726	21632718	HG-U133_Plus_2	Microarray analysis was performed to determine the transcriptional profiles of NKT, CD1d-aGC+ Va24-, and CD4 T cells.
GEO	GSE30687	21985787	HG-U133A_2	Gene profiling of metastatic colorectal cancer cells (L1 and L2) and non-mestastatic colorectal cancer cells (sw620 and sw480)
GEO	GSE35340	23074278	HG-U133_Plus_2	Transcriptome analysis of highly purified CD207+/CD1a+ Langerhans cell histiocytosis (LCH) cells derived from different locations and disease courses and three major human dendritic cell lineages: epidermal Langerhans cells, myeloid dendritic cells (mDC1) and plasmacytoid dendritic cells (pDC).
GEO	GSE49910	24053356	HG-U133_Plus_2	Meta-analysis of publically available human primary cell data
GEO	GSE51540	24492460	HG-U133_Plus_2	Cassical Th17 cells (Th17) or those generated in the presence of the inhibitor (iTh17) are sorted and analyzed by full transcriptome microarray analysis.

Table S2: List of immune metagenes.

**Name**	**CellType**
AKNA	Activated B cells
ARHGAP25	Activated B cells
CCL21	Activated B cells
CD180	Activated B cells
CD2	Activated B cells
CD27	Activated B cells
CD37	Activated B cells
CD38	Activated B cells
CLEC17A	Activated B cells
CLEC9A	Activated B cells
CLECL1	Activated B cells
FAIM3	Activated B cells
FAM65B	Activated B cells
GIMAP4	Activated B cells
MAP4K1	Activated B cells
PAX5	Activated B cells
TNFRSF17	Activated B cells
TRAF3IP3	Activated B cells
ANLN	Activated CD4
BRIP1	Activated CD4
BUB1B	Activated CD4
CASC5	Activated CD4
CCNB1	Activated CD4
CCNB2	Activated CD4
CCNE2	Activated CD4
CEP55	Activated CD4
CKAP2L	Activated CD4
DLGAP5	Activated CD4
DTL	Activated CD4
E2F8	Activated CD4
ECT2	Activated CD4
ESCO2	Activated CD4
EXO1	Activated CD4
EXOC6	Activated CD4
FBXO5	Activated CD4
FIGNL1	Activated CD4
HELLS	Activated CD4
HMMR	Activated CD4
IARS	Activated CD4
KIF11	Activated CD4
KIF18A	Activated CD4
KIF20A	Activated CD4
KNTC1	Activated CD4
MAD2L1	Activated CD4
MASTL	Activated CD4
MTHFD2	Activated CD4
NCAPG2	Activated CD4

NCAPH	Activated CD4
NEIL3	Activated CD4
NUF2	Activated CD4
PRC1	Activated CD4
PSAT1	Activated CD4
RTKN2	Activated CD4
ADRM1	Activated CD8
AHSA1	Activated CD8
C1GALT1C1	Activated CD8
CCT5	Activated CD8
CCT6B	Activated CD8
CETN3	Activated CD8
CSE1L	Activated CD8
EIF2S1	Activated CD8
GAL	Activated CD8
GEMIN6	Activated CD8
GPT2	Activated CD8
KIAA0101	Activated CD8
MND1	Activated CD8
MPZL1	Activated CD8
MRPS16	Activated CD8
PCNA	Activated CD8
PTRH2	Activated CD8
RFC5	Activated CD8
SPC25	Activated CD8
TIMM13	Activated CD8
TIMM8B	Activated CD8
TK1	Activated CD8
TUBB	Activated CD8
TXNDC17	Activated CD8
ADAM19	Central memory CD4
AHNAK	Central memory CD4
AIM2	Central memory CD4
COL4A1	Central memory CD4
CYLD	Central memory CD4
DPEP2	Central memory CD4
EPHA4	Central memory CD4
FYN	Central memory CD4
IL6R	Central memory CD4
IPCEF1	Central memory CD4
KLF10	Central memory CD4
MAL	Central memory CD4
MALT1	Central memory CD4
NXN	Central memory CD4
OPTN	Central memory CD4
PLCL2	Central memory CD4
PNRC1	Central memory CD4
SMAD4	Central memory CD4
SPOCK2	Central memory CD4
TRIB2	Central memory CD4

TXK	Central memory CD4
ADAM23	Central memory CD8
ATP2B4	Central memory CD8
BCL2L11	Central memory CD8
CD300A	Central memory CD8
CD80	Central memory CD8
CHN1	Central memory CD8
F2R	Central memory CD8
F8	Central memory CD8
GABARAPL1	Central memory CD8
GPR183	Central memory CD8
KLRK1	Central memory CD8
MSC	Central memory CD8
PYHIN1	Central memory CD8
RCAN2	Central memory CD8
RGS1	Central memory CD8
SLAMF1	Central memory CD8
SLFN11	Central memory CD8
ZEB2	Central memory CD8
BST2	Cytotoxic cells
BTN3A2	Cytotoxic cells
IL10RA	Cytotoxic cells
KLRB1	Cytotoxic cells
LAT2	Cytotoxic cells
LILRA2	Cytotoxic cells
SELL	Cytotoxic cells
SLAMF6	Cytotoxic cells
SLAMF7	Cytotoxic cells
C1QC	DC
CCDC88A	DC
CCL13	DC
CCL3L1	DC
CCL3L3	DC
CD300E	DC
CD86	DC
CLEC1A	DC
CLEC4C	DC
CLEC5A	DC
CLIC2	DC
CSF2RA	DC
FAM49A	DC
FCGR1A	DC
FN1	DC
FSTL1	DC
GPR109B	DC
GPX3	DC
HLA-DQA2	DC
INHBA	DC
LGMN	DC
NGFR	DC

PDGFRL	DC
PDPN	DC
PRKAR2B	DC
SIGLEC1	DC
SIGLEC5	DC
SLAMF9	DC
STAB1	DC
THBD	DC
TNFAIP2	DC
TTYH2	DC
UBD	DC
VCAM1	DC
BMI1	Effector memory CD4
CASP3	Effector memory CD4
CNOT10	Effector memory CD4
COPB2	Effector memory CD4
DARS	Effector memory CD4
EXOSC9	Effector memory CD4
EZH2	Effector memory CD4
GDE1	Effector memory CD4
IFT74	Effector memory CD4
KLF5	Effector memory CD4
NDUFB9	Effector memory CD4
NUP205	Effector memory CD4
SHCBP1	Effector memory CD4
XRCC6	Effector memory CD4
APOBEC3H	Effector memory CD8
ARHGAP10	Effector memory CD8
ATP10D	Effector memory CD8
BIRC3	Effector memory CD8
C3AR1	Effector memory CD8
CCL4	Effector memory CD8
CD244	Effector memory CD8
CD55	Effector memory CD8
CFLAR	Effector memory CD8
CMKLR1	Effector memory CD8
DAPP1	Effector memory CD8
DFNB31	Effector memory CD8
DRAM1	Effector memory CD8
EFNA5	Effector memory CD8
FCGR2C	Effector memory CD8
FCGR3A	Effector memory CD8
FCRL6	Effector memory CD8
FGR	Effector memory CD8
GPR114	Effector memory CD8
GZMH	Effector memory CD8
HAPLN3	Effector memory CD8
HLA-DMB	Effector memory CD8
HLA-DPA1	Effector memory CD8
HLA-DPB1	Effector memory CD8

IFI16	Effector memory CD8
IKZF2	Effector memory CD8
IL15	Effector memory CD8
IL2RB	Effector memory CD8
JAKMIP1	Effector memory CD8
KLRD1	Effector memory CD8
LY96	Effector memory CD8
NFKBIA	Effector memory CD8
PLXNC1	Effector memory CD8
PPP4R1	Effector memory CD8
PTPN22	Effector memory CD8
RGS18	Effector memory CD8
SETD7	Effector memory CD8
SH2D1B	Effector memory CD8
SLA2	Effector memory CD8
STXBP1	Effector memory CD8
TLR5	Effector memory CD8
TYROBP	Effector memory CD8
CCL24	Eosinophil
CFD	Eosinophil
CREB3	Eosinophil
EGR1	Eosinophil
EPX	Eosinophil
FBXO16	Eosinophil
HFE	Eosinophil
HIVEP1	Eosinophil
HIVEP2	Eosinophil
MBP	Eosinophil
PRNP	Eosinophil
RNASE2	Eosinophil
RNASE3	Eosinophil
S100A4	Eosinophil
SIGLEC8	Eosinophil
ATP6V1A	iDC
C1QB	iDC
CHST11	iDC
CSF1	iDC
DPYD	iDC
FNDC3B	iDC
GRP	iDC
IL1RN	iDC
INPP5F	iDC
LPL	iDC
LPXN	iDC
MMD	iDC
PLAU	iDC
PLCB2	iDC
RAB38	iDC
RDX	iDC
RRAGD	iDC

TACSTD2	iDC
TGM2	iDC
TIMD4	iDC
TM7SF4	iDC
BANK1	Immature B cells
CD22	Immature B cells
CYBB	Immature B cells
ETS1	Immature B cells
FAM129C	Immature B cells
FCRL1	Immature B cells
FCRL2	Immature B cells
FCRL3	Immature B cells
FCRL5	Immature B cells
FCRLA	Immature B cells
HDAC9	Immature B cells
HLA-DOB	Immature B cells
HLA-DQA1	Immature B cells
HVCN1	Immature B cells
KIAA0226	Immature B cells
NCF1	Immature B cells
NCF1B	Immature B cells
P2RY10	Immature B cells
PNOC	Immature B cells
SP100	Immature B cells
STAP1	Immature B cells
TAGAP	Immature B cells
TXNIP	Immature B cells
ZCCHC2	Immature B cells
AIF1	Macrophages
CCL1	Macrophages
CCL14	Macrophages
CCL26	Macrophages
CD163	Macrophages
CD300LB	Macrophages
CNR1	Macrophages
CNR2	Macrophages
CPM	Macrophages
CSF3R	Macrophages
ENG	Macrophages
FCAR	Macrophages
IGF1	Macrophages
IL34	Macrophages
L1CAM	Macrophages
LILRA1	Macrophages
LILRA5	Macrophages
LRP1	Macrophages
MS4A7	Macrophages
MS4A8B	Macrophages
TREM1	Macrophages
TREML1	Macrophages

ACSL4	Mast cells
ADAMTS3	Mast cells
ADCYAP1	Mast cells
ARHGAP15	Mast cells
ATP8B4	Mast cells
C19orf59	Mast cells
CASQ1	Mast cells
CLC	Mast cells
CMA1	Mast cells
CPA3	Mast cells
CTSG	Mast cells
DUSP14	Mast cells
EGR3	Mast cells
EMR1	Mast cells
FST	Mast cells
HDC	Mast cells
HEY1	Mast cells
KRT1	Mast cells
LXN	Mast cells
MEIS2	Mast cells
MS4A2	Mast cells
MS4A3	Mast cells
NLRP3	Mast cells
NTRK1	Mast cells
PLAT	Mast cells
PTGS1	Mast cells
PTGS2	Mast cells
RGS16	Mast cells
SCG2	Mast cells
SDPR	Mast cells
SERPINB2	Mast cells
SIGLEC14	Mast cells
SIGLEC6	Mast cells
SLC18A2	Mast cells
SLC24A3	Mast cells
TAL1	Mast cells
TARP	Mast cells
TIE1	Mast cells
TIMP1	Mast cells
TNFAIP6	Mast cells
TSPAN4	Mast cells
VAT1	Mast cells
ATP5O	mDC
CBX1	mDC
DNAJC15	mDC
ENSA	mDC
FOXN3	mDC
PRCP	mDC
SSB	mDC
TPMT	mDC

AICDA	Memory B cells
CCNA2	Memory B cells
CDKN3	Memory B cells
CLCN5	Memory B cells
ENPP1	Memory B cells
FCER1A	Memory B cells
FCRL4	Memory B cells
MYC	Memory B cells
RUNX2	Memory B cells
SORL1	Memory B cells
SOX5	Memory B cells
STAT5A	Memory B cells
STAT5B	Memory B cells
TLR9	Memory B cells
ACTR3	Monocytes
ANXA5	Monocytes
ARPC2	Monocytes
ATP6V1B2	Monocytes
BASP1	Monocytes
CD300LF	Monocytes
DAZAP2	Monocytes
EIF4A1	Monocytes
EIF4G2	Monocytes
EMP3	Monocytes
FCN1	Monocytes
FTL	Monocytes
GABARAP	Monocytes
HIF1A	Monocytes
LITAF	Monocytes
NCOA4	Monocytes
OLR1	Monocytes
RAB1A	Monocytes
RHOA	Monocytes
SAT1	Monocytes
SDCBP	Monocytes
SRGN	Monocytes
TEK	Monocytes
TMBIM6	Monocytes
UBE2D3	Monocytes
ABTB1	Neutrophils
AMPD2	Neutrophils
CAMP	Neutrophils
EMR4P	Neutrophils
FPR1	Neutrophils
FPR2	Neutrophils
GPR77	Neutrophils
MAEA	Neutrophils
PROK2	Neutrophils
SEC14L1	Neutrophils
SEPX1	Neutrophils

SLC25A37	Neutrophils
TNFSF14	Neutrophils
TREML4	Neutrophils
VNN2	Neutrophils
XPO6	Neutrophils
AKT3	NK
AXL	NK
CDH2	NK
CRTAM	NK
CYTH1	NK
FASLG	NK
GRB2	NK
KLRG1	NK
LILRB5	NK
LST1	NK
MAPK4	NK
NOTCH3	NK
PIK3CG	NK
PILRA	NK
PLCG2	NK
SIGLEC7	NK
SIGLEC9	NK
C11orf75	NK56 bright
DYNLL1	NK56 bright
HCP5	NK56 bright
HDGFRP2	NK56 bright
KRT86	NK56 bright
MLST8	NK56 bright
MYL6B	NK56 bright
TAX1BP3	NK56 bright
AKR7A3	NK56 dim
CLTB	NK56 dim
FAM27A	NK56 dim
GLS2	NK56 dim
GPRC5C	NK56 dim
GRIN1	NK56 dim
HLA-E	NK56 dim
KIR2DS4	NK56 dim
KLHL21	NK56 dim
KRT80	NK56 dim
MPL	NK56 dim
PHRF1	NK56 dim
PORCN	NK56 dim
PQBP1	NK56 dim
PSMC4	NK56 dim
TEX264	NK56 dim
UPP1	NK56 dim
BTN2A2	NKT
CD101	NKT
GNLY	NKT

KIR2DL1	NKT
KIR2DL3	NKT
KIR3DL1	NKT
KIR3DL2	NKT
KLRC1	NKT
MICB	NKT
NCR1	NKT
NFATC2IP	NKT
TNFRSF11A	NKT
CBX6	pDC
DAB2	pDC
DDX17	pDC
HIGD1A	pDC
IDH3A	pDC
IL3RA	pDC
MAGED1	pDC
NUCB2	pDC
OFD1	pDC
OGT	pDC
PDIA4	pDC
SERTAD2	pDC
SIRPA	pDC
SPCS3	pDC
TMED2	pDC
TMX1	pDC
UGCG	pDC
ZDHHC17	pDC
BATF	T cells
BTLA	T cells
CCL11	T cells
CCL17	T cells
CCL22	T cells
CCL3	T cells
CCL5	T cells
CCL7	T cells
CCR1	T cells
CCR3	T cells
CCR4	T cells
CCR8	T cells
CD1A	T cells
CD1B	T cells
CD1C	T cells
CD1D	T cells
CD1E	T cells
CD247	T cells
CD28	T cells
CD3D	T cells
CD3E	T cells
CD3G	T cells
CD69	T cells

CD8A	T cells
CXCR1	T cells
CXCR4	T cells
CXCR5	T cells
CXCR6	T cells
FGFBP2	T cells
FLT3	T cells
FOSL1	T cells
GRAP2	T cells
HLA-DRA	T cells
HLA-DRB1	T cells
HLA-DRB5	T cells
HRH2	T cells
IFNG	T cells
IL10	T cells
IL12B	T cells
IL12RB1	T cells
IL12RB2	T cells
IL18R1	T cells
IL18RAP	T cells
IL1B	T cells
IL21	T cells
IL21R	T cells
IL24	T cells
IL26	T cells
IL27	T cells
IL2RA	T cells
IL6	T cells
IL7	T cells
IRF4	T cells
IRF8	T cells
ITGAL	T cells
ITK	T cells
JAK2	T cells
JAK3	T cells
LAIR1	T cells
LAT	T cells
LAX1	T cells
LCK	T cells
LCP2	T cells
LILRA3	T cells
LILRA4	T cells
LILRB1	T cells
LILRB2	T cells
LILRB3	T cells
LILRB4	T cells
MS4A4A	T cells
NAPSB	T cells
NFATC1	T cells
NFKB2	T cells

P2RY14	T cells
PIK3CD	T cells
POU2F2	T cells
PRF1	T cells
RUNX3	T cells
SPI1	T cells
STAT1	T cells
STAT4	T cells
TNF	T cells
TPRG1	T cells
TSLP	T cells
UBASH3B	T cells
ZAP70	T cells
B3GAT1	TFH
BCL6	TFH
CCR7	TFH
CD200	TFH
CD83	TFH
CD84	TFH
CDK5R1	TFH
FGF2	TFH
GPR18	TFH
PDCD1	TFH
ACP5	TGD
C1orf54	TGD
CARD8	TGD
CCL18	TGD
CD209	TGD
CD33	TGD
CECR1	TGD
CLEC10A	TGD
CLEC4A	TGD
CSF1R	TGD
FCER1G	TGD
FCGR2A	TGD
FCGR2B	TGD
FCGR3B	TGD
FGF7	TGD
FGL2	TGD
FPR3	TGD
GM2A	TGD
GPNMB	TGD
HCK	TGD
HK3	TGD
LIPA	TGD
MARCO	TGD
MMP12	TGD
MNDA	TGD
MRC1	TGD
MS4A6A	TGD

NPL	TGD
PLA2G7	TGD
PLEK	TGD
RAB23	TGD
RNASE6	TGD
SDC2	TGD
SLAMF8	TGD
SLC15A3	TGD
SLC38A6	TGD
SLC7A7	TGD
SPP1	TGD
TFEC	TGD
TM6SF1	TGD
TNFSF8	TGD
TREM2	TGD
TRPV2	TGD
ADAM8	Th1
CCR5	Th1
CD151	Th1
CD48	Th1
CD52	Th1
CD53	Th1
CD6	Th1
CD68	Th1
CD7	Th1
CD96	Th1
CD97	Th1
EBI3	Th1
GPR84	Th1
HAVCR2	Th1
ICAM1	Th1
ICAM3	Th1
IGSF6	Th1
IL12A	Th1
IL7R	Th1
IRF1	Th1
ITGA4	Th1
ITGAM	Th1
ITGAX	Th1
ITGB2	Th1
ITGB4	Th1
ITGB7	Th1
LTA	Th1
LTB	Th1
METRNL	Th1
P2RX5	Th1
PTPRC	Th1
SPN	Th1
TBX21	Th1
TLR6	Th1

TNFRSF1A	Th1
TNFRSF9	Th1
TRAT1	Th1
ABP1	Th17
B4GALNT4	Th17
C2CD4A	Th17
C2CD4B	Th17
CA2	Th17
CCDC65	Th17
CEACAM3	Th17
CHRM3	Th17
DOC2B	Th17
F12	Th17
FURIN	Th17
GPR25	Th17
IL17A	Th17
IL17C	Th17
IL17F	Th17
IL17RC	Th17
IL17RE	Th17
IL23A	Th17
ILDR1	Th17
LONRF3	Th17
LTK	Th17
MOCOS	Th17
SH2D6	Th17
TNIP2	Th17
TRAF3IP2	Th17
YBX2	Th17
ASB2	Th2
CALD1	Th2
CCR2	Th2
CSRP2	Th2
DAPK1	Th2
DLC1	Th2
DNAJC12	Th2
DUSP6	Th2
GATA3	Th2
GNAI1	Th2
HTR2B	Th2
LAMP3	Th2
NRP2	Th2
OSBPL1A	Th2
PDE4B	Th2
PHLDA1	Th2
PLA2G4A	Th2
RAB27B	Th2
RBMS3	Th2
RNF125	Th2
SIGLEC10	Th2

SKAP1	Th2
SMAD2	Th2
TMPRSS3	Th2
UBASH3A	Th2
CCL19	Treg
CD34	Treg
CD72	Treg
CTLA4	Treg
FOXP3	Treg
GADD45B	Treg
GEM	Treg
IL1RL1	Treg
IL9R	Treg
MADCAM1	Treg
MYH10	Treg
NCF2	Treg
RCSD1	Treg
RYR1	Treg
SELE	Treg
SELP	Treg
SFRP1	Treg
SIT1	Treg
TIGIT	Treg
TLR10	Treg
TLR2	Treg
TLR7	Treg
TLR8	Treg
TRAF1	Treg
WIPF1	Treg
TGFB1	Treg
ADORA3	MDSC
AG2	MDSC
ARG1	MDSC
BIN2	MDSC
C1orf162	MDSC
CAPS	MDSC
CD117	MDSC
CD11B	MDSC
CD11C	MDSC
CD124	MDSC
CD14	MDSC
CD15	MDSC
CD163L1	MDSC
CD1D1	MDSC
CD21	MDSC
CD23	MDSC
CD274	MDSC
CD31	MDSC
CD35	MDSC
CD40	MDSC

CD43	MDSC
CD44	MDSC
CD66B	MDSC
COX2	MDSC
CR2	MDSC
CTR9	MDSC
EBP	MDSC
FAM48A	MDSC
FAM70B	MDSC
FCER2	MDSC
FCGRT	MDSC
FERMT3	MDSC
FLOT1	MDSC
FLT1	MDSC
GIMAP7	MDSC
GLI4	MDSC
GNA15	MDSC
GPR34	MDSC
GPSM3	MDSC
HLA-DR	MDSC
IDO	MDSC
IKZF1	MDSC
IL12	MDSC
IL13	MDSC
IL18BP	MDSC
IL1R	MDSC
IL4RA	MDSC
INPP5D	MDSC
ITGA3	MDSC
KDR	MDSC
KRIT1	MDSC
LGALS3	MDSC
MGAT4A	MDSC
NAIP	MDSC
NEK3	MDSC
NFSF13	MDSC
NOG	MDSC
PARVG	MDSC
PDRG1	MDSC
PECAM1	MDSC
PIK3R5	MDSC
PPP1R2P4	MDSC
PSAP	MDSC
PTGES2	MDSC
PTPRE	MDSC
RNASE1	MDSC
RP11	MDSC
S100A8	MDSC
S100A9	MDSC
SELPLG	MDSC

SLA	MDSC
SLC36A1	MDSC
SLC44A1	MDSC
ST8SIA4	MDSC
STAT3	MDSC
STAT6	MDSC
TBXAS1	MDSC
TFGFB1	MDSC
TFGFB2	MDSC
TFGFB3	MDSC
TFGFB5	MDSC
TFRC	MDSC
TGFB2	MDSC
TPP1	MDSC
VTCN1	MDSC

Table S3. Univariate and multivariate Cox Hazards Analysis of Patients with molecular phenotypes

Variables	Univariate Cox model		Multivariate Cox model*		
	HR (95% CI)	p	HR (95% CI)	p	sign†
Tem CD8	0.46 (0.16-0.85)	0.026	0.61 (0.12-0.88)	0.044	*
Tem CD4	0.54 (0.43-0.98)	0.038	0.59 (0.35-1.01)	0.051	·
Treg	2.25 (1.57-3.69)	0.015	2.11 (1.24-3.76)	0.038	*
NK	0.64 (0.42-0.88)	0.057	0.69 (0.35-0.93)	0.065	·
Act DC	0.71 (0.43-1.12)	0.031	0.71 (0.33-1.19)	0.042	*
MDSC	2.39 (1.62-4.06)	0.023	2.15 (1.33-4.21)	0.037	*
Act CD4	0.42 (0.33-0.87)	0.402	0.52 (0.12-1.05)	0.621	
Act CD8	0.36 (0.12-0.93)	0.052	0.45 (0.23-0.78)	0.059	·
Tcm CD4	0.57 (0.32-1.12)	0.701	0.45 (0.11-1.32)	0.881	
Tcm CD8	0.31 (0.09-0.82)	0.023	0.52 (0.24-0.96)	0.036	*
Th1	0.65 (0.12-0.78)	0.041	0.66 (0.32-0.87)	0.068	·
Th2	0.62 (0.31-1.15)	0.458	0.69 (0.23-1.48)	0.651	
Th17	2.35 (1.16-3.66)	0.057	2.64 (1.02-3.69)	0.066	·
Tgd	0.56 (0.33-1.05)	0.552	0.44 (0.15-1.26)	0.651	
Tfh	0.61 (0.12-1.45)	0.991	0.75 (0.41-1.69)	0.875	
Act B	0.66 (0.28-1.15)	0.899	0.67 (0.24-1.56)	0.901	
Imm B	0.65 (0.23-1.31)	0.657	0.46 (0.30-1.67)	0.664	
Mem B	1.47 (1.01-3.99)	0.356	1.12 (0.93-3.68)	0.401	
Mac	2.57 (1.17-5.58)	0.097	2.01 (1.68-4.69)	0.125	
Mast	1.97 (1.05-3.59)	0.039	2.14 (0.89-3.70)	0.114	
Mon	0.64 (0.43-1.35)	0.879	0.45 (0.10-1.24)	0.993	
pDC	1.15 (0.67-2.01)	0.705	1.41 (0.45-2.96)	0.781	
iDC	0.77 (0.36-1.09)	0.845	0.46 (0.12-1.15)	0.658	
mDC	0.57 (0.24-1.06)	0.997	0.47 (0.22-1.01)	0.778	
NKT	0.54 (0.12-0.93)	0.652	0.61 (0.30-1.16)	0.933	
Eos	0.51 (0.23-1.28)	0.678	0.64 (0.11-1.34)	0.752	
Neu	0.78 (0.52-1.16)	0.999	0.98 (0.23-1.36)	0.999	
NK CD56dim	0.66 (0.35-0.98)	0.773	0.46 (0.22-1.19)	0.801	
NK CD56bright	0.55 (0.41-1.25)	0.871	0.75 (0.43-1.39)	0.923	
MSI status (MSI-H vs. MSS)	0.85 (0.49-1.98)	0.821			
Mutation rate (Hypermutated vs. Non-hypermutated)	0.94 (0.46-1.86)	0.835			
Methylation subtype (CIMP-H vs. CIMP-L/CIMPneg)	1.28 (0.94-3.68)	0.635			
Tumor site (Right vs. Left colon)	0.58 (0.32-1.06)	0.547			

* including molecular phenotypes (MSI, mutation Rate, methylation subtype, tumor site) as binary covaria

† $p<0.001$ … ***, $0.001\leq p<0.01$ … **, $0.01\leq p<0.05$ … *, $0.05\leq p<0.1$ … ·

Table S4: List of predicted neo-antigens

Peptide sequence(s)	# Patients	Chromosome:StartPosition	Genes
TEYKLVVVGAV	28	12:25398284	KRAS
QASPALASL	19	11:117789313	TMPRSS13
TQLARFFPI	19	17:56435161--17:56435252	RNF43
SPAQASPAL	16	11:117789313--11:117789312	TMPRSS13
AQASPALASL	16	11:117789313	TMPRSS13
RFFPITPPV	15	17:56435161--17:56435252	RNF43
GADGVGKSAL	14	1:115258747--12:25398284	KRAS--NRAS
MQLCTQLARFF	14	17:56435252--17:56435161	RNF43
FVAAEFPGSEL	13	19:1065018	ABCA7
ARFFPITPPV	13	17:56435161--17:56435252	RNF43
FFDETRQL	13	3:178916876	PIK3CA
WRFCSGVL	13	8:120220776	MAL2
WLPPPMFLYL	13	8:120220776	MAL2
MQLCTQLARF	12	17:56435252--17:56435161	RNF43
RRQPCAHGLWL, RRQPCAHGL	11	19:39330958--19:39330959	HNRNPL
WRFCSGVLSGF	11	8:120220776	MAL2
ARAVHLPEL	11	2:239050033	KLHL30
YLYYKDGSCL	11	8:120220776	MAL2
QLARFFPI	11	17:56435161	RNF43
RRLGFLATAWL	11	10:17659131	PTPLA
LTITSLSLPI	11	5:140215022	PCDHA7
FLATAWLTF	10	10:17659131	PTPLA
LTITSLSL	10	5:140215022	PCDHA7
ARAVHLPELL	10	2:239050033	KLHL30
FFDETRQLCDL	10	3:178916876	PIK3CA
SEITKQEKDFL	10	3:178936091	PIK3CA
SRRQPCAHGL	10	19:39330959--19:39330958	HNRNPL
MRRNMISTL, MRRNMISTLQM	9	16:2059622	ZNF598
IMMSWMPPL	9	1:204228411	PLEKHA6
YVDPSGGDHF	9	18:48591919	SMAD4
YYKDGSCL	9	8:120220776	MAL2
WRPILTIITL	9	17:7577539	TP53
KIGDFGLATEK	9	7:140453136	BRAF
FTPLSAHPVPV	9	1:204228411	PLEKHA6
SPAQASPALA	9	11:117789313	TMPRSS13
TRAPSSAWRF	9	8:120220776	MAL2
LLGRNSFEVHV	9	17:7577120	TP53
FWLPPPMFL, FWLPPPMFLYL	9	8:120220776	MAL2
SAMPSAMGVAL	9	1:204228411	PLEKHA6
KRRGGPPSPPL	9	17:56435161	RNF43
VRHDPQARAV	9	2:239050033	KLHL30
RAVHLPELLSL	9	2:239050033	KLHL30
VVGADGVGK	8	1:115258747--12:25398284	KRAS--NRAS
WSLGRSFLPL	8	16:2059622	ZNF598
VTSATWTM	8	16:2059622	ZNF598
YLLDGLVRL	8	19:17837512	MAP1S
CSMRRNMISTL	8	16:2059622	ZNF598
TSATWTMMSCL	8	16:2059622	ZNF598
AAEFPGSEL	8	19:1065018	ABCA7
VRTSMLTL	8	1:204228411	PLEKHA6
WLPPPMFL	8	8:120220776	MAL2
KYLKIKHLLL	8	5:112175626--5:112175596-- 5:112175594--5:112175605-- 5:112175572--5:112175606	APC
RQLCDLRLF	8	3:178916876	PIK3CA
ARHGGWTTKM	8	3:178952085	PIK3CA
VAAEFPGSEL	8	19:1065018	ABCA7
FPALACSL	8	2:36825137	FEZ2
MMSWMPPL, SIMMSWMPPL	8	1:204228411	PLEKHA6
FPITPPVWHIL	8	17:56435161	RNF43
QARAVHLPEL	8	2:239050033	KLHL30
RAEWPGLAL	8	16:2059622	ZNF598
SSAWRFCSGVL	8	8:120220776	MAL2
WRTSAMPSAM	8	1:204228411	PLEKHA6
QASPAQASPAL	8	11:117789313	TMPRSS13
VVVGAVGVGK, VVGAVGVGK	8	12:25398284	KRAS
FFPITPPV	8	17:56435161	RNF43
RMQLCTQLARF	7	17:56435161	RNF43
VRTSMLTLLPM, VRTSMLTLL	7	1:204228411	PLEKHA6
HPHHKQAGPV, GPVAHQLHVL, HPHHKQAGPVA, GPVAHQLHV	7	13:21562948	LATS2
GRNSFEVHV	7	17:7577120	TP53
EEFFDETRQL	7	3:178916876	PIK3CA
RAHRVQLL	7	18:3452223	TGIF1
ALPPPPPPPV	7	10:105110740	PCGF6
RAVHLPEL	7	2:239050033	KLHL30
MRGSELPLVL	7	18:56887507	GRP
YELSSTSV	7	13:21562832	LATS2
LTSAPSAQQSL	7	3:114058003	ZBTB20
SMRRNMISTL	7	16:2059622	ZNF598
SELREAHGGRL, AEFPGSEL	7	19:1065018	ABCA7
HAQCPSVPL	7	8:145625525	CPSF1
RTSMLTLLPM	7	1:204228411	PLEKHA6
ASDAYPSAF	7	1:29138975	OPRD1
CRVTPMTRRTV	7	16:2059622	ZNF598
RRNMISTLQM, RRNMISTL	7	16:2059622	ZNF598
ITPPVWHIL	7	17:56435252--17:56435161	RNF43
MMSWMPPLA, IMMSWMPPLA	7	1:204228411	PLEKHA6
SRLLCSRPL	7	7:128587374	IRF5
SRPLCSRPL	7	7:128587381--7:128587374-- 7:128587374--7:128587381	IRF5
TRCATAAL	7	16:2059622	ZNF598
MRATGPPPSL	7	19:39330959	HNRNPL
YELSSTSVQQW	7	13:21562832	LATS2
SMLTLLPM	7	1:204228411	PLEKHA6
TMMSCLSTCAA, MMSCLSTCAA	7	16:2059622	ZNF598
ARSCARWSL, ARWSLGRSFL	7	16:2059622	ZNF598
ARAPTAVPAF	7	1:204228411	PLEKHA6
NQRPILTIITL	7	17:7577538	TP53
MAPTPPSSTPM	7	1:204228411	PLEKHA6
VASAWLTVL, AVASAWLTV, VASAWLTV	7	1:228504670	OBSCN
SPAQASPALAS	7	11:117789313	TMPRSS13
MAPETTLWL	7	22:20130522--22:20130515	ZDHHC8
ARAEWPGL, ARAEWPGLAL	7	16:2059622	ZNF598
LARFFPITPPV	7	17:56435161	RNF43
VVAVATWR, CVVAVATWR	7	19:49611319	SNRNP70
FLATAWLTFY	7	10:17659131	PTPLA
RRHLQPHDAL	7	7:42005678	GLI3
SAMPSAMGV	7	1:204228411	PLEKHA6
RRAAHSRV	7	18:3452223	TGIF1
REEFFDETRQL	7	3:178916876	PIK3CA
SRPTGRPATV	7	16:2059622	ZNF598
SALARPVSAAL	6	7:75677504	MDH2
HRRAAHSRV	6	18:3452223	TGIF1
WVRHDPQARAV	6	2:239050033	KLHL30
MAPTPPSSTPMI	6	1:204228411	PLEKHA6
SQSIMMSW	6	1:204228411	PLEKHA6
RRRRRCPGGRV	6	17:3627473	GSG2
LLAPATEHSSL	6	17:72350412	KIF19
KRRRLGFL	6	10:17659149--10:17659131-- 10:17659131--10:17659149	PTPLA
LQMDFLVHPA	6	5:112175766--5:112175756-- 5:112175752--5:112175772-- 5:112175770--5:112175761	APC
SAHPVPVL	6	1:204228411	PLEKHA6
TTRCATAAL	6	16:2059622	ZNF598
CLLDILDTAGK	6	1:115256530	NRAS
SELPLVLLAL	6	18:56887507	GRP
TRFLTVPV	6	7:139167934	KLRG2
SATQASMTL	6	22:20130515--22:20130522	ZDHHC8
CTQLARFFPI	6	17:56435161	RNF43
ARWSLGRSF	6	16:2059622	ZNF598
MRAGGCCHAL	6	10:46999601	GPRIN2
LRPGHLHLL	6	8:145625525	CPSF1
RELYDRGPPL	6	19:49850473	TEAD2
QRPILTIITL	6	17:7577538	TP53
FAVLGTMVM	6	1:150530506--1:150529443	ADAMTSL4
RRGDPRLKTF, LRRGDPRLKTF	6	1:204228411	PLEKHA6
MENSHPPTTTT, MENSHPPT	6	12:122242658	SETD1B
TSMLTLLPM	6	1:204228411	PLEKHA6
AAAGAVFL	6	10:71906150	TYSND1
HVAHAAAAL, VAHAAAAL	6	2:43452464--2:43452491-- 2:43452244--2:43452439-- 2:43452342	ZFP36L2
APATEHSSLSL	6	17:72350412	KIF19
TEHSSLSL, TEHSSLSLHSL	6	17:72350412	KIF19
LFHPWTTDM	6	2:171572940	SP5
AALPPPPPPPV	6	10:105110740	PCGF6
RAPEVPRCAM	6	22:20130515--22:20130522	ZDHHC8
FTPLSAHPV	6	1:204228411	PLEKHA6
SSYSRVALV	6	21:37507501	CBR3
SEITKQEKDF	6	3:178936091	PIK3CA
STQPHVPPSTL	6	17:17119709	FLCN
ALARPVSAAL	6	7:75677504	MDH2
ARPGPWRTSAM	6	1:204228411	PLEKHA6
RRHAQCPSVPL	6	8:145625525	CPSF1
GSMSSSAAV	6	16:2059622	ZNF598
FAALANAL	6	19:1004896	GRIN3B
RTSAMPSAM	6	1:204228411	PLEKHA6
VLSGCHLAV	6	1:204226546--1:204228411-- 1:204228411	PLEKHA6
AMPSAMGV	6	1:204228411	PLEKHA6
SRLFHPWTTDM	6	2:171572940	SP5
AEFPGSELREA	6	19:1065018	ABCA7
GRRASVPCRPM, RRASVPCRPM, TRQECGRRASV	6	1:204228411	PLEKHA6
TRHHHPAHHF	6	X:54209387	FAM120C
RTLACVCAL	6	19:17837425	MAP1S
SSAWRFCSGV	6	8:120220776	MAL2
RSCVVAVATWR	6	19:49611319	SNRNP70
DTAGKEEY	5	1:115256530--12:25380277	KRAS--NRAS
GRSAVGDPEL	5	17:40706906	HSD17B1
CRRKVLRL	5	19:47878858	DHX34

HAAAALLL, VAHAAAALL, HAAAALLLFGL, VAHAAAALLL	5	2:43452464--2:43452244--2:43452439--2:43452342	ZFP36L2
NQRPILTI	5	17:7577538	TP53
KIKHLLLK	5	5:112175596--5:112175606--5:112175553--5:112175605--5:112175594	APC
ATAGAPPALM	5	7:100285170	GIGYF1
SQAPPGSQSI	5	1:204228411	PLEKHA6
MTSLSASTSL	5	17:17119709	FLCN
LQLPAAAPPV	5	2:43452532--2:43452244--2:43452464--2:43452342--2:43452439	ZFP36L2
KPSEKYLKI	5	5:112175605--5:112175572--5:112175606--5:112175626	APC
VLFPGCTPPAY	5	19:17837512	MAP1S
VRLWRLRGGAL	5	16:705360	WDR90
AMAPETTLWL	5	22:20130515--22:20130522	ZDHHC8
CEICVKNTAI, CEICVKNTAIL	5	18:34205516	FHOD3
RTTTATMPTCV	5	16:2059622	ZNF598
KTFGVPLGSIL	5	1:204228411	PLEKHA6
YMCNSSCMGSM	5	17:7577548	TP53
MTACSILARL	5	22:20130522--22:20130515	ZDHHC8
QLCDLRLFQPF	5	3:178916876	PIK3CA
SPVRRRRRC	5	17:3627473	GSG2
LSAHPVPVL, LSAHPVPV	5	1:204228411	PLEKHA6
LMAPVGSV	5	17:48433967	XYLT2
CAMDSLRPV	5	12:121432118--12:121432115	HNF1A
QMDFLVHPA	5	5:112175766--5:112175761--5:112175772--5:112175752	APC
SAWLTVLGL	5	1:228504670	OBSCN
LPAPFRVNHAV	5	5:112175351--5:112175484--5:112175346--5:112175350--5:112175424	APC
TAFWRSLL	5	19:51015404	ASPDH
VERCPWKGICW	5	10:29760116	SVIL
MRGSELPLVLL	5	18:56887507	GRP
CRDAVASAWL	5	1:228504670	OBSCN
VLAAAGAV, VLAAAGAVFL	5	10:71906150	TYSND1
TMMSCLSTCA	5	16:2059622	ZNF598
STSGRSTF	5	16:2059622	ZNF598
TRQLCDLRL, TRQLCDLRLF	5	3:178916876	PIK3CA
PRAHRVQLL, FRGPHPRAHRV, FRGPHPRAH	5	18:3452223	TGIF1
AATTRCATAAL	5	16:2059622	ZNF598

LSAPCPAQVL, SAPCPAQVLL, LSAPCPAQVLL, LSAPCPAQV, FAALANALL	5	19:1004896	GRIN3B
SRPLCSRLL, SRPLCSRL	5	7:128587374	IRF5
LVVVGASGVGK	5	12:25398285	KRAS
SMSSSAAV	5	16:2059622	ZNF598
RRGRRTSLAAL, RRGRRTSL	5	2:43452464--2:43452244--2:43452439--2:43452342	ZFP36L2
SRMAHPRL	5	22:31485922	SMTN
LARPVSAAL	5	7:75677504	MDH2
LAAAGAVFL	5	10:71906150	TYSND1
RVTPMTRRTV	5	16:2059622	ZNF598
RVLLSCARGPL, LLSCARGPL	5	10:135186806	ECHS1
LPPPMFLYL	5	8:120220776	MAL2
KRRRLGVL	5	10:17659149	PTPLA
FAVLGTMV	5	1:150530506--1:150529443	ADAMTSL4
TSATWTMM	5	16:2059622	ZNF598
LRALREMVSNM	5	13:46288145	SPERT
YPSAFPSA, YPSAFPSAGA	5	1:29138975	OPRD1
TPMTRRTV	5	16:2059622	ZNF598
RARTARSAL	5	18:34205516	FHOD3
GMWGGGCWV	5	8:145689659	CYHR1
AEDEAGRSAV	5	17:40706906	HSD17B1
VTSATWTMM	5	16:2059622	ZNF598
LLMNLATADL, LLMNLATADLL	5	19:17000632	F2RL3
IRSEGDQLL	5	19:51843808	VSIG10L
TRLMAPVGSVM	5	17:48433967	XYLT2
KQAGPVAHQL	5	13:21562948	LATS2
CRRGRRRL, CRRGRRRLL	5	2:43452342--2:43452439--2:43452244--2:43452464	ZFP36L2
ARWSTSFCL, LRARWSTSFCL	5	22:31485922	SMTN
STRDPLSEITK	5	3:178936091	PIK3CA
LANPVLPNEV	5	19:45867259	ERCC2
MNWRPILTI	5	17:7577539	TP53
RESRFWLA, RESRFWLAL	5	18:34205516	FHOD3
RLMAPVGSVM, LMAPVGSVM	5	17:48433967	XYLT2
RAPSSAWRF	5	8:120220776	MAL2
HVAHAAAALL, HAAAALLLF, HVAHAAAALLL	5	2:43452342--2:43452439--2:43452491--2:43452464	ZFP36L2
HSSATQASMTL	5	22:20130522--22:20130515	ZDHHC8
HLANPVLPNEV	5	19:45867259	ERCC2
ALLALLLAA, ALLALLLA, ALLALLALLL, ALLALLALLLA	5	1:85039999	CTBS
RAPQPPGLAV	5	2:43452439--2:43452342--2:43452464--2:43452244	ZFP36L2

HPRAHRVQL, HPRAHRVQLL	5	18:3452223	TGIF1
CAMAPETTLWL	5	22:20130515--22:20130522	ZDHHC8
SPRRAGPTAL	5	1:155178782	MTX1
APATEHSSL	5	17:72350412	KIF19
FLATAWLT	5	10:17659131	PTPLA
RAPCRPPPV	5	18:34205516	FHOD3
LAAAGAVF	5	10:71906150	TYSND1
SPRLESPRAPA	5	1:41976328	HIVEP3
MRMKKWAPL, MRMKKWAPLM	5	7:100283942--7:100285170	GIGYF1
RRESRFWL, RRESRFWLAL	5	18:34205516	FHOD3
FEVHVCACPG	5	17:7577120	TP53
SSYSRVAL	5	21:37507501	CBR3
FSIRRWSTI	5	7:150698398	NOS3
HRAAPSALGAL, HRAAPSAL	5	6:18122506	NHLRC1
TMAPTPPSTPM	5	1:204228411	PLEKHA6
MLSALARPV	5	7:75677504	MDH2
YITEADSKEAY	5	14:32561296	ARHGAP5
GPRMQLCTQL	5	17:56435161	RNF43
KEEYSAMRDQY	4	12:25380277--1:115256530	KRAS--NRAS
LLDILDTAGL	4	12:25380276--1:115256529	KRAS--NRAS
ILDTAGKEEY	4	12:25380277--1:115256530	KRAS--NRAS
SVYSGNLL	4	2:97749725--2:96078466	FAHD2B--FAHD2A
DILDTAGKEEY, DTAGKEEYSAM	4	12:25380277--1:115256530	NRAS--KRAS
AELQARLAAL	4	19:7747293	TRAPPC5
FRVNHAVEW, LRVVRLPAPF	4	5:112175346--5:112175424--5:112175351	APC
SRAASLLF	4	7:150698398	NOS3
ALAPQAWWPA	4	3:114058003	ZBTB20
LSPEPPAYSL	4	X:3631167	PRKX
FEVCVCACPG	4	17:7577121	TP53
GEWELCRAA	4	1:200880978	C1orf106
RQPCAHGLWL	4	19:39330958--19:39330959	HNRNPL
SRSARTGRSV	4	22:20130522--22:20130515	ZDHHC8
STSGRSTFCV	4	16:2059622	ZNF598
WEALGAPEAL, WEALGAPEA	4	19:622336	POLRMT
ARRSCVVAV, RRSCVVAV	4	19:49611319	SNRNP70
VRRAPQPPGL, RRAPQPPGLAV, AAAPPVRLARV, RRAPQPPGL	4	2:43452244--2:43452464--2:43452342--2:43452439	ZFP36L2
RWPPRTPL, RWPPRTPLFPL, RRWPPRTPL, RWPPRTPLF, RRWPPRTPLF	4	5:137803132	EGR1
QSIMMSWMPPL	4	1:204228411	PLEKHA6

WVRPRRRLL	4	22:38477342	SLC16A8
MFLYLYYK, PMFLYLYYK	4	8:120220776	MAL2
MQDPLLSLWTL	4	3:168833257	MECOM
SYLEDVRLI, VRLIHRDL	4	17:37881332	ERBB2
TRPRLLPL, RLLPLTRPRLL, TRPRLLPLI	4	5:137803132	EGR1
RRWATWLSLAL, SRRWATWL, SRRWATWLSL, RRWATWLSL, SRSRRWATWL	4	10:124895627	HMX3
RAAQPDSPSEV	4	2:43452491--2:43452464--2:43452439	ZFP36L2
LTIQNAAFL, YQDTSLMMIVL	4	13:20638677	ZMYM2
RTWPGIACRV	4	16:2059622	ZNF598
RPSTRFLTV, SPRPSTRFLTV	4	7:139167934	KLRG2
RRQPPPTTSF	4	16:30736370	SRCAP
CWAPAPGSPRL	4	22:20130522--22:20130515	ZDHHC8
RAAHSRVPSPR	4	18:3452223	TGIF1
MENSHPPTT, MENSHPPTTT	4	12:122242658	SETD1B
LPTPPPPPLLL	4	1:109792735	CELSR2
IPEMDFEPSPA	4	2:39187190	ARHGEF33
AEWPGLAL	4	16:2059622	ZNF598
SVHLIVLR, RVVRLPAPFR	4	5:112175350--5:112175351--5:112175346	APC
KYPQAAAV, LRPVRLQKY	4	12:121432115--12:121432118	HNF1A
YTVSPRKVDPV	4	5:176026129--5:176026138--5:176026120--5:176026138--5:176026129	GPRIN1
WRSLLACCQL	4	19:51015404	ASPDH
RRERTAAWQL	4	16:50745399	NOD2
FSSPAWPQF	4	15:40544617	C15orf56
ATADLLLALAL, ATADLLLAL	4	19:17000632	F2RL3
SGAWTMRMKK, GAWTMRMKK	4	7:100283942--7:100285170	GIGYF1
YPVQRLPF	4	2:242674803	D2HGDH
GTDPNLVL	4	19:17392894	ANKLE1
EVVRHCPH	4	17:7578406	TP53
FFPITPPVWHI	4	17:56435161	RNF43
ALACSLEEKL	4	2:36825137	FEZ2
APQNGGSSE	4	16:88599701--16:88599696--16:88599703--16:88599703--16:88599696--16:88599701	ZFPM1
EPSPAEPL	4	2:39187190	ARHGEF33
FFDETRQLC	4	3:178916876	PIK3CA
GSELPLVLLAL, GSELPLVL	4	18:56887507	GRP
WLWAGWTV	4	14:20846338	TEP1
MSSYSRVALV, MSSYSRVAL	4	21:37507501	CBR3

RPLLHLHL	4	17:70119909--17:70120314--17:70120194--17:70120345	SOX9
LRCPLTAPL, ARDLRCPL	4	12:121432115--12:121432118	HNF1A
TRKQTKVLWPL, LRPPPLSL, TRKQTKVL	4	5:137803132	EGR1
PRAHRVQL	4	18:3452223	TGIF1
RPTGRPATV	4	16:2059622	ZNF598
HPHRRHLWGPF	4	17:70120345--17:70120194--17:70120314--17:70119909	SOX9
SRPRSAASL	4	5:137803132	EGR1
GRIHRLAL	4	19:6737481	GPR108
MRPPRAGPLGL	4	22:19968971	ARVCF
ARLVATPAL	4	22:20130515--22:20130522	ZDHHC8
SMQDPLLSL	4	3:168833257	MECOM
LTSPRPLTL	4	12:58025103	B4GALNT1
HTSTVHCM	4	4:153249384	FBXW7
RSLELGAAFPL, MRRSLELGAAF	4	19:33167455	RGS9BP
APFRVNHAV	4	5:112175350--5:112175346--5:112175484--5:112175351	APC
HRVRPRACM	4	16:50745399	NOD2
CARWSLGRSF	4	16:2059622	ZNF598
RRPWNSFTSL, VRRPWNSFTSL	4	16:50745399	NOD2
MTACSILARLV	4	22:20130515--22:20130522	ZDHHC8
CAMAPETTL	4	22:20130515--22:20130522	ZDHHC8
MNWRPILTII	4	17:7577539	TP53
RSRSWTSK	4	7:100283942--7:100285170	GIGYF1
ATFSSVPLV, FSSVPLVASI	4	1:22191454	HSPG2
RRPHSCLADV, RRPHSCLADVL	4	5:112175351--5:112175346--5:112175424	APC
QRASRMRRWWM, MRAQRASRM, SRMRRWWMNAA, SRMRRWWM	4	19:871222	MED16
SELPLVLLALV, SELPLVLL, SELPLVLLA	4	18:56887507	GRP
RAAPSALGAL	4	6:18122506	NHLRC1
VVRHCPHHER	4	17:7578406	TP53
FPSAGANA	4	1:29138975	OPRD1
SAYLGRTL, SAYLGRTLLV, SAYLGRTLL	4	10:890939	LARP4B
FANNSGFV, WAFANNSGF, WAFANNSGFV, FANNSGFVAEL	4	9:140008750	DPP7
WRLRGGAL	4	16:705360	WDR90

RMTSLSASTSL	4	17:17119709	FLCN
TSSPRRSPALR, TTTTSSPR	4	12:122242658	SETD1B
EPAPAPATY	4	16:84224967	ADAD2
SRPPVTQRL	4	2:70315174	PCBP1
ERYPVQRLPF, TRERYPVQRL, QRLPFSTV, ERYPVQRL	4	2:242674803	D2HGDH
LARLVATPAL	4	22:20130515--22:20130522	ZDHHC8
ARRESRFWLAL, SRMPARRESRF	4	18:34205516	FHOD3
NPVLPNEV, LPNEVLQEAV	4	19:45867259	ERCC2
APANPEPSA	4	1:41976328	HIVEP3
YLWPSRTL	4	14:89878534	FOXN3
LMVEAVAMM	4	X:135956575--X:135956571	RBMX
KVHLNTMFR, KVHLNTMFRR, KVVLRHPK	4	5:112175346--5:112175351--5:112175350	APC
RELLLVGRY, GERELLLVGRY	4	17:18918510--17:18918511	SLC5A10
LRGPPPRKL	4	16:15802687	MYH11
SSRQHRSALM, HRSALMTF, SRQHRSALMTF, HRSALMTFLL, SRQHRSALM, HRSALMTFL, SRQHRSAL	4	16:50745399	NOD2
LRARGGTTI	4	12:122242658	SETD1B
ARTARSAL, VRARTARSAL	4	18:34205516	FHOD3
IRSEGDQLLI, IRSEGDQLLIV	4	19:51843808	VSIG10L
VRQQRLRL	4	2:43452439--2:43452342--2:43452464--2:43452244	ZFP36L2
SLAALQLPA, QLPAAAPPV, ALQLPAAAPPV, SLAALQLPAA	4	2:43452244--2:43452532--2:43452464--2:43452342	ZFP36L2
CTACVERCPWK	4	10:29760116	SVIL
QAWWPARRGPL	4	3:114058003	ZBTB20
AAAPPVRL	4	2:43452464--2:43452439--2:43452342	ZFP36L2
NQRPILTII	4	17:7577538	TP53
LLFLGLRGL, LLLRLLLFL, LLLRLLLFLGL, FLGLRGLHAL	4	2:43452244--2:43452532--2:43452464--2:43452342	ZFP36L2
RESRFWLALQA	4	18:34205516	FHOD3
APAPAQPSAT, APAQPSAT	4	9:124855211	TTLL11
SPVRRRRRCPG	4	17:3627473	GSG2
RRGRRRLL	4	2:43452464--2:43452244--2:43452439--2:43452342	ZFP36L2
AEATNGEARAA	4	16:88599696	ZFPM1
FEPSPAEPL, MDFEPSPAEPL	4	2:39187190	ARHGEF33
SLASCASVAI, SLASCASV	4	1:150529443--1:150530506	ADAMTSL4
VLQMDFLV	4	5:112175752--5:112175772--5:112175761--5:112175766	APC

RLMAPVGSV	4	17:48433967	XYLT2
FVVADLLL	4	1:154304153	ATP8B2
RLLLLLLPLLW	4	19:41858921	TGFB1
LMNLATADLLL	4	19:17000632	F2RL3
MRPLPGRIEV, MRPLPGRI	4	18:658064	C18orf56
SPRRAGPT	4	1:155178782	MTX1
MTEVVRHCPH	4	17:7578406	TP53
AARAEWPGLAL	4	16:2059622	ZNF598
KEAYGRHQREI	4	14:32561296	ARHGAP5
GMNQRPILTI	4	17:7577538	TP53
FWLPPPMF	4	8:120220776	MAL2
TAVPAFTPL, RAPTAVPAF	4	1:204228411	PLEKHA6
LLPRALPSLL	4	18:34205516	FHOD3
APFRVNHAVEW	4	5:112175493--5:112175424--5:112175351--5:112175484	APC
RTSMLTLL	4	1:204228411	PLEKHA6
REMVSNMSGPS, REMVSNMS, REMVSNMSG, KELRALREM	4	13:46288145	SPERT
GLGVEVPGV	4	1:227922918	JMJD4
LSYHRLLPL, LPLWAALPL	4	2:43452491--2:43452464--2:43452439	ZFP36L2
SSATQASMTL, HSSATQASM	4	22:20130522--22:20130515	ZDHHC8
GAVGVGKSAL	4	12:25398284	KRAS
LEQMLHSCRI	4	X:56591874	UBQLN2
LWDVLLSRELF	4	1:15850613	CASP9
VRSTVCAMDSL, VRSTVCAM, LRPVRLQKY	4	12:121432118--12:121432115	HNF1A
FPPPRSPC, FPPPRSPCPPA	4	8:120220776	MAL2
KLVVVGAAGV	4	12:25398284	KRAS
RSFPAASPSGL	4	20:23066469	CD93
TPMTRRTVGT, WPGIACRVTPM, MTRRTVGTRSA, TPMTRRTVG	4	16:2059622	ZNF598
ATEHSSLSL	4	17:72350412	KIF19
KRNTGTLGSL	4	11:8751889	ST5
WATWLSLAL, RSRRWATWLSL, RSRRWATWL	4	10:124895627	HMX3
CMGGMNWR	4	17:7577539	TP53
SLPTRPRLL, LRPPPLSL, RLLPLTRPRLL	4	5:137803132	EGR1
ALRARGGTTI, SPRRSPAL	4	12:122242658	SETD1B
GPHPRAHRVQL	4	18:3452223	TGIF1
RLLPLWAAL, RLLPLWAALPL	4	2:43452464--2:43452491--2:43452532--2:43452342	ZFP36L2
LGFLATAWLTF	4	10:17659131	PTPLA

MSRPQLRRWL, MSRPQLRRWLL	4	1:85040070	CTBS
GRRTSLAALQL, RRTSLAAL, RRTSLAALQL, GRRTSLAAL	4	2:43452342--2:43452439--2:43452244--2:43452464	ZFP36L2
RQLCDLRL	4	3:178916876	PIK3CA
WSLGRSFL	4	16:2059622	ZNF598
LRRPCSGARPV, RRPCSGARPV	4	16:50745399	NOD2
VPLPTPPPPPL	4	1:109792735	CELSR2
RRHAQCPSV	4	8:145625525	CPSF1
TPPPPPLLL	4	1:109792735	CELSR2
LASCASVAI	4	1:150529443--1:150530506	ADAMTSL4
HTSTVHCMHLH	4	4:153249384	FBXW7
CRPACTCISM	4	17:48433967	XYLT2
HLYGSYPPA	4	17:48433967	XYLT2
TEHSSLSLH, TEHSSLSLHS	4	17:72350412	KIF19
LLSPLDSSPL	4	3:77657038	ROBO2
LRPGHLHL	4	8:145625525	CPSF1
GRPDMLRL	4	2:43452439--2:43452342--2:43452464--2:43452244	ZFP36L2
LRWLFPTGGSV	4	20:60887581	LAMA5
KYLKIKHLL, KYLKIKHL	4	5:112175594--5:112175606--5:112175605--5:112175572	APC
CSMRRNMI	4	16:2059622	ZNF598
AEASTGAW, AEASTGAWTA	4	18:34205516	FHOD3
KYSRWIFL	4	5:112175760--5:112175752--5:112175756	APC
GRTPTTRLM	4	17:48433967	XYLT2
GLPAGAAAQV	4	2:43452244--2:43452532--2:43452464--2:43452342	ZFP36L2
GIACRVTPM	4	16:2059622	ZNF598
CEICVKSHHRV	4	18:34205516	FHOD3
LPRCGCSASSA, LPRCGCSA	4	16:2059622	ZNF598
SEKYLKIKHLL	4	5:112175594--5:112175553--5:112175596--5:112175626	APC
CTPPAYLL	4	19:17837512	MAP1S
RASVPCRPM	4	1:204228411	PLEKHA6
MASAPPGERL	4	1:248020556	TRIM58
VRSVEALTPL	4	19:17393015	ANKLE1
YSATHRIHEI	4	19:11978669	ZNF439
FPGCTPPAYL	4	19:17837512	MAP1S
WTMRMKKWAPL	4	7:100285170	GIGYF1
CRRPSRTTF, RRPSRTTF	4	16:50745399	NOD2
ARHPDPQL	4	2:43452464--2:43452244--2:43452439--2:43452342	ZFP36L2
FPVNGPPRDSL	4	1:213031948	FLVCR1
ARALPPDVHHL	4	22:24121378	MMP11

LMMWRTPM, LLMMWRTPM, RAWRRFPLLM, MMWRTPMTTRL	4	22:18300932	MICAL3
RAPPVVLRL	4	1:151318741	RFX5
GRYGGGGRLV, GRYGGGGRLVI	4	17:18918511--17:18918510	SLC5A10
RAAARAVGGRL	4	2:43452439--2:43452491--2:43452464	ZFP36L2
RLWFREAEIY	4	9:101900288	TGFBR1
FRGLGVEV	4	1:227922918	JMJD4
QPPTLQPPTL	4	7:128587374	IRF5
YQDTSLMM, YQDTSLMMIV, RLYQDTSLM, YQDTSLMMI, LYQDTSLMM, LYQDTSLM	4	13:20638677	ZMYM2
APGKRRRL	4	10:17659149	PTPLA
GRFAVLGTMVM, AREAGRFAVL	4	1:150530506	ADAMTSL4
SRVPSPRSW	4	18:3452223	TGIF1
SAPCPAQVL	4	19:1004896	GRIN3B
VATPALPTQQL	4	22:20130522--22:20130515	ZDHHC8
RATGPPPSL	4	19:39330959	HNRNPL
EAAAVVTAAF	4	7:100285170	GIGYF1
GTFPVNGPPR	4	1:213031948	FLVCR1
SYSRVALV	4	21:37507501	CBR3
FLLLLLLLLV	4	20:44520238	CTSA
LLLFGLVLLL, LLFGLVLLLL, LLLRLLLFL, LLLRLLLFLGL, LLFGLVLL, LLLFGLVLL, LLLFGLVLLLL, LLFGLVLLL	4	2:43452464--2:43452244--2:43452532--2:43452342	ZFP36L2
GEKTEIQLTM	4	2:203420130	BMPR2
TMMSCLST, MMSCLSTCA	4	16:2059622	ZNF598
CTQLARFF	4	17:56435161	RNF43
MMIGSQHL, KMMIGSQHLL	4	3:77657038	ROBO2
SARRSCVVAV	4	19:49611319	SNRNP70
AARSCARWSL	4	16:2059622	ZNF598
FSLNLTPEAVL, FSLNLTPEAV	4	6:166721224	PRR18
HVPPSTLWGV	4	17:17119709	FLCN
FVCENNSPAL	3	5:140604458--5:140531213--5:140595076	PCDHB13--PCDHB6--PCDHB14
FRMGLYTGEI	3	5:140209546--5:140237500--5:140188642	PCDHAC1, PCDHAC2, PCDHA2, PCDHA12, PCDHA5, PCDHA11, PCDHA1, PCDHA7, PCDHA6, PCDHA8, PCDHA13, PCDHA10, PCDHA9, PCDHA3, PCDHA4--PCDHA6--PCDHA10
CLLDILDTAGL	3	12:25380276--1:115256529	KRAS--NRAS
ILDTAGREEY	3	1:115256529--12:25380276	NRAS--KRAS
SRRFKEPWFL	3	19:30935604--18:74154167	ZNF516--ZNF536
YIFSDKMGTL	3	1:154313476--5:160061438	ATP8B2--ATP10B
WRFCPQACL	3	19:54745497--19:54725745	LILRA6, LILRB3--LILRB3
VVGAAGVGK, VVVGAAGVGK	3	12:25398284--1:115258747	KRAS--NRAS
LIHEITHTGEK	3	19:12542084--19:12059564	ZNF443--ZNF700
KAFFCPVYY	3	19:12384448--19:12502590	ZNF44--ZNF799
GREEYSAM	3	12:25380276--1:115256529	KRAS--NRAS
GEDMVVAL, GEDMVVALV, GEDMVVALVL, EDMVVALVL	3	17:39742887--17:39768744	KRT16--KRT14
EVVRHCPHH	3	17:7578406	TP53
LATEKSRW, FGLATEKSRW	3	7:140453136	BRAF
QEVVVHKKRGL	3	2:148683686	ACVR2A
SAWRFCSGVL	3	8:120220776	MAL2
LLGPHSLMV, YHLLGPHSL, LLGPHSLMVTI, MKNPYHLL, YHLLGPHSLM, VSMKNPYHLL, SMKNPYHLL, HLLGPHSLMV	3	10:63958149	RTKN2
HRPGTPPPL	3	2:74687410	WBP1
LPNEVLQEA	3	19:45867259	ERCC2
LALGPRMQL	3	17:56435161	RNF43
RLHTAHSTL	3	17:70119909--17:70120194--17:70120345	SOX9
RSALMTFLL, ALMTFLLASW	3	16:50745399	NOD2
IRQLSSKSL	3	14:75230947	YLPM1
FLFTTRGPSA	3	19:50713713	MYH14
LRLPLCPPQM	3	9:135947054--9:135947065	CEL
RPTGRPATVAA	3	16:2059622	ZNF598
AAPPHRLL	3	19:48197891	GLTSCR1
MALARTSV	3	15:74219546	LOXL1
ITPPVWHI, RFFPITPPVW, FFPITPPVW	3	17:56435161	RNF43
VLHKQINK, TTVLHKQINK, TVLHKQINK	3	6:64421713	PHF3
LPTPPPPPLL, LPTPPPPPL	3	1:109792735	CELSR2
SSATQASM	3	22:20130522--22:20130515	ZDHHC8
GLDDRSPQAPL	3	X:129190011	BCORL1
RPQLRRWLL, RPQLRRWLLV	3	1:85040070	CTBS
MMIGSQHLL, LLFEAWLLLLL, LLFEAWLLLL, LLFEAWLLL, LLFEAWLL	3	3:77657038	ROBO2
GLMAADRAWGL, MAADRAWGLF, MAADRAWGL, LMAADRAWGL, AADRAWGLF	3	7:128852004	SMO
FRSSHPFECY	3	1:223177026	DISP1
WLFPTGGSV	3	20:60887581	LAMA5
SSTPAAGSSL	3	18:3456550--18:3457371--18:3456572	TGIF1
WMRPPRSLL	3	22:18300932	MICAL3
HRLLPLWAAL, SYHRLLPL	3	2:43452439--2:43452342--2:43452464	ZFP36L2
SLLFSIRR, RAASLLFSIR, RAASLLFSIRR, AASLLFSIRR	3	7:150698398	NOS3
SFHPLVATQAL	3	1:228559450	OBSCN
RVMVPLPV	3	3:3067806	CNTN4
KQTKVLWPL	3	5:137803132	EGR1
CLAHTQFLR, LAHTQFLRAL	3	17:80400208--17:80400298	HEXDC
YLQDLDFIF, FINKGLHL, ILVPFFHLYL, YLQDLDFI, YLQDLDFIFI	3	8:113241029	CSMD3
RLLPLWAA	3	2:43452342--2:43452532--2:43452464	ZFP36L2
FWLPPPMFLY	3	8:120220776	MAL2
NEVLQEAV	3	19:45867259	ERCC2
EAFFAFSSLL, EAFFAFSSL	3	12:51392999	SLC11A2
CFLQCFQGGW, CFLQCFQGGWW	3	10:29760116	SVIL
KTPPTNALTL	3	5:137803132	EGR1
SPRAPANPE, SPRAPANPEP, SPRAPANPEPS, APANPEPSAT	3	1:41976328	HIVEP3
QQLAAVRGVAL	3	12:111800827	FAM109A
STWTRRRDSPR	3	1:155178782	MTX1
FQMMEGKAF	3	11:124845049	CCDC15
TFSNVNNY	3	16:88599701--16:88599696--16:88599696--16:88599701	ZFPM1
LAVRTSML, LAVRTSMLTLL, LAVRTSMLTL	3	1:204228411	PLEKHA6
LQMDFLVH	3	5:112175756--5:112175772--5:112175752	APC
FASVVNYF, YFIEKENYHYV	3	13:48615145	NUDT15
WLLLLLSPL, LLFEAWLL, LLFEAWLLL, LLFEAWLLLLL, LLFEAWLLLL	3	3:77657038	ROBO2
LYELSSTSV	3	13:21562832	LATS2
LEADMRRSLEL	3	19:33167455	RGS9BP
MQQDSTRTV	3	2:95537572	TEKT4
REEYSAMRDQY	3	1:115256529	NRAS
SVTSATWTM, SSVTSATWTM	3	16:2059622	ZNF598
KTYGARGV	3	18:56246440	ALPK2
TAMPQMQLQAL	3	17:40937417--17:40939870	WNK4
LQDTHGWKSEL	3	2:95537622	TEKT4
LSAQTTAL	3	2:71206267	ANKRD53
LAFNVPGGVWL	3	14:20846338	TEP1
YHRWIVTSM, LYHRWIVTSM, HRWIVTSM	3	10:890939	LARP4B
CMGGMNQR	3	17:7577538	TP53
YPVQRLPFSTV	3	2:242674803	D2HGDH
WRSTQPHV	3	17:17119709	FLCN
ILVPFFHL, ILVPFFHLYL	3	8:113241029	CSMD3
RRLGFLATAW	3	10:17659131	PTPLA
MALLSEGLDEL	3	20:37555116	FAM83D
TTTTSSPRR, TTTSSPRR	3	12:122242658	SETD1B
ITDFGHSEIL, ITDFGHSEI	3	22:29091840	CHEK2
HRIHEITHI, SYSATHRIHEI	3	19:11978669	ZNF439
FLRSRSWTSK, KGWRRRRNLPK	3	7:100285170--7:100283942	GIGYF1
RDAVASAWL	3	1:228504670	OBSCN
WTAATLRCPAV	3	3:51417604	DOCK3
RTLACVCALL	3	19:17837425	MAP1S
LEIRQLSSKSL	3	14:75230947	YLPM1
FPGSELREA	3	19:1065018	ABCA7

RPACTCISM	3	17:48433967	XYLT2
RERTAAWQL	3	16:50745399	NOD2
LVASISAVSL, SSVPLVASI	3	1:22191454	HSPG2
ILARLVATPAL, ILARLVATPA	3	22:20130522--22:20130515	ZDHHC8
GRSSRQPRL	3	10:124895627	HMX3
LPHALLAPSL	3	1:228559450	OBSCN
TSSPRRSPAL	3	12:122242658	SETD1B
MPSAMGVAL	3	1:204228411	PLEKHA6
APSGHEGILAL	3	11:76751542	B3GNT6
RTSLAALQL	3	2:43452439--2:43452464	ZFP36L2
NWRPILTI	3	17:7577539	TP53
GRTPTTRL, TRLMAPVGSV	3	17:48433967	XYLT2
LQMIWAFY	3	1:35972480	KIAA0319L
LRQARRGPPGL	3	3:183951135	VWA5B2
APPSTRMMAT, APPSTRMM, APPSTRMMA	3	2:227661664	IRS1
SPFRSVGV, SPFRSVGVSA	3	17:7577036--17:7577065--17:7576881	TP53
FPISFIPG, TPPGGAAFPI, STPPGGAAF, FPISFIPGA	3	1:154942910	SHC1
CPLCSPRWL	3	3:183951135	VWA5B2
LCMFFEPLLTI	3	2:46987060	SOCS5
YVDPSGGDH	3	18:48591919	SMAD4
RHWWLWWWIW, RHWWLWWW	3	12:53207602	KRT4
AEWPGLALA	3	16:2059622	ZNF598
HPADPQPPA, HPADPQPPAL	3	17:70120194--17:70120345--17:70119909	SOX9
LRSRRRGL	3	10:124895627	HMX3
KLMHTKIDKLR	3	10:91497569	KIF20B
RASPSAVCL	3	20:23066469	CD93
NVFVLNLFHV, FVLNLFHVPV, YVYIIETNVFV, LLYVYIIETNV, VLNLFHVPV, YIIETNVFVL, FVLNLFHV, YIIETNVFV	3	18:12699829	CEP76
TVHCMHLHEKR	3	4:153249384	FBXW7
MAVLPALL	3	11:107992346	ACAT1
KTSTIALF	3	8:25154087	DOCK5
CSRLLCSR	3	7:128587374	IRF5
RRETEPCMLL, ARRETEPCMLL	3	1:156814343--1:156814053	INSRR
RRRRCPGGRV	3	17:3627473	GSG2
ALFAALANAL	3	19:1004896	GRIN3B
MTFLLASWCV	3	16:50745399	NOD2
SQSGAWTM	3	7:100285170	GIGYF1
YGMWGGGCWVW	3	8:145689659	CYHR1
KLFNEMAERH, KLFNEMAER	3	22:25315953	SGSM1

TMNDSKHK, LTMNDSKHK, KLESPALK	3	2:203420130	BMPR2
YAARGRGQWAV	3	10:1094906	IDI1
TSFPFLSGL	3	16:30736370	SRCAP
SSHRFCFHQY	3	7:91732038	AKAP9
WRLLQAQAAGV	3	1:6614391	NOL9
LAGSLSTM, FGVPLGSILCL	3	1:204228411	PLEKHA6
YMMKKGKRDKL, KLPFLLLI, KRDKLPFLL, KRDKLPFLLL	3	10:63958149	RTKN2
LEGGTPPWMPF, LEGGTPPWM, LEGGTPPW	3	10:100186987	HPS1
WRVSPRPLSPL, WRVSPRPL	3	13:111368164	ING1
MTSMTSMTSM, TSMTSMTSM	3	22:38483198--22:38483204--22:38483174	BAIAP2L2
HVHPSRLL	3	10:114900984	TCF7L2
QQTGKSPPV	3	19:58879976	ZNF837
VAFPRPRPF	3	19:1231142	C19orf26
FRRLVSTL, RRLVSTLNF, FFFRRLVSTL, KFFFRRLVSTL, KFFFRRLV, FRRLVSTLNF	3	10:94243045	IDE
RPLCSRLL	3	7:128587374	IRF5
AVASAWLTVL	3	1:228504670	OBSCN
RRDDDGTL	3	4:24801315	SOD3
TRWKPLQTVRL, TRWKPLQTV	3	6:151673000	AKAP12
FLPPMHCPTA	3	22:20130515--22:20130522	ZDHHC8
GEWELCRA	3	1:200880978	C1orf106
RPIAQWHPL	3	11:1858572	SYT8
MAAMRLMPKF, MAAMRLMPKFL	3	2:27248517	MAPRE3
FRFRGRLV, VRFFRFRGRLV, FFRFRGRLV, FRFRGRLVW, RFFRFRGRLV	3	19:54974318	LENG9
RMWNDTVQK	3	4:62910205	LPHN3
MSPMTPMTPM, SMSPMTPMTPM	3	22:38483155	BAIAP2L2
STNPFNFPF, ISTNPFNFPF, STNPFNFPFV	3	17:10258071	MYH13
KSSCLQIHQR	3	19:44778796--19:44778797	ZNF233
FTFCDVFWL, KLIRFLCF, FTFCDVFWLL, KLIRFLCFTF, FYKLIRFLCF	3	11:111742146	ALG9
LYYKDGSCL	3	8:120220776	MAL2
RAPSSFHPL, RAPSSFHPLV	3	1:228559450	OBSCN
RVWPSTTTL, VWPSTTTL	3	6:13206135	PHACTR1
KLAACARECV	3	17:2266799	SGSM2
SALPQGRAPL	3	18:34205516	FHOD3

LPWHIQIGILI, LPWHIQIGI	3	12:66622063	IRAK3
FSNVNNYYVH	3	16:88599701--16:88599696--16:88599696--16:88599701	ZFPM1
WLGPASVAPA, TLWLGPASV	3	22:20130515--22:20130522	ZDHHC8
LRLQRSLLEI, IRKQRQHL, LRLQRSLL	3	10:61828580	ANK3
SRRWATWLSLA	3	10:124895627	HMX3
HLWGPFHPA	3	17:70120314--17:70120194--17:70120345	SOX9
RVLLSCAR	3	10:135186806	ECHS1
HRPLCQRFPTL, SRHAAHRPL	3	16:14346300	MKL2
SWTPALRTM	3	3:52551362--3:52550119	STAB1
ETRQLCDLR	3	3:178916876	PIK3CA
SRCSSFLLPL, SRCSSFLL	3	1:156814343--1:156814053	INSRR
VAPPTPPPLLL, LLLSLHPSL	3	2:204305619	RAPH1
HVWSHHTL	3	19:990281	WDR18
ASVAPATL	3	22:20130522--22:20130515	ZDHHC8
VRAGNPAVAAL	3	3:139258508	RBP1
NAPQLTITSL	3	5:140215022	PCDHA7
MAHPPHYL, IMAHPPHYL	3	1:55081756	FAM151A
FANNSGFVA	3	9:140008750	DPP7
TLWGVRMTSL	3	17:17119709	FLCN
LPPPMFLYLY, LPPPMFLY	3	8:120220776	MAL2
FQEEPMTGFYV	3	16:28847350--16:28847356	ATXN2L
SRKRIIVI, SRKRIIVINYI	3	10:63958149	RTKN2
KSHHRVVF	3	18:34205516	FHOD3
AALANALL, FAALANALLSA	3	19:1004896	GRIN3B
LPAPFRVNHA, VVRLPAPFR, VVRLPAPF	3	5:112175351--5:112175346--5:112175350	APC
KRTKCFTF	3	10:89717770	PTEN
SEGAETPRI, AESEGAETPRI	3	19:51843808	VSIG10L
FMQEFYKLIRF, FYKLIRFLCF, KLIRFLCFTF, KLIRFLCF, FYKLIRFL	3	11:111742146	ALG9
KQMNDARHGGW	3	3:178952085	PIK3CA
RMMRRQWSL	3	6:116836954	FAM26E
SREERLWF	3	9:101900288	TGFBR1
RTGPRTTTATM	3	16:2059622	ZNF598
GRNSFEVCV	3	17:7577121	TP53
YGPPPTSL	3	19:39330958	HNRNPL
YPSAFPSAG, FPSAGANAS	3	1:29138975	OPRD1
FTLGAGPRFL	3	8:144810138	FAM83H
RQPCAHGLW	3	19:39330959--19:39330958	HNRNPL
RLWRAWRRFPL, RAWRRFPL, RAWRRFPLL, RAWRRFPLLMM	3	22:18300932	MICAL3
MSPSLLDYL, YEMSPSLLDY	3	9:134183379	PPAPDC3

GEAVAPAL, GEAVAPALL	3	8:8860620	ERI1
RAGTRGSL	3	16:2059622	ZNF598
LLAPPPPSAPL	3	14:75230947	YLPM1
SPTCLGHPV	3	19:10202224	C19orf66
SQNDFQSLL	3	21:30701851	BACH1
REMVSNMSGP, KELRALREMV, ALREMVSNM	3	13:46288145	SPERT
QSGAWTMRMKK, QSGAWTMRMK, SQSGAWTMRMK, GAWTMRMK	3	7:100283942--7:100285170	GIGYF1
GSELPLVLL	3	18:56887507	GRP
TPRIRSEGDQL	3	19:51843808	VSIG10L
SELREAHG, SELREAHGG	3	19:1065018	ABCA7
FARRECPPEPA	3	17:79899272	MYADML2
TADLLLAL, TADLLLALAL	3	19:17000632	F2RL3
FLHPHCKIALF, FLHPHCKIAL	3	10:85978958	CDHR1
MAPVGSVM	3	17:48433967	XYLT2
GEKTEIQL	3	2:203420130	BMPR2
LRELYDRGPPL	3	19:49850473	TEAD2
RMPARRESRF	3	18:34205516	FHOD3
SRMRRWWMNA, MRRWWMNAA	3	19:871222	MED16
ATGSSPGK	3	20:23618427	CST3
IQDFIFFVMPL	3	5:72199545	TNPO1
WLVLVRLEPV, LLLWLVLV	3	3:66550762--3:66550756--3:66550756--3:66550762	LRIG1
CTYPTQKPF	3	8:39091459	ADAM32
RTLACVCALLV	3	19:17837425	MAP1S
ASAPTSPL	3	22:46449891	C22orf26
FTRAERLTWL, TRAERLTWL	3	11:72408055	ARAP1
KRSSQSRSPV	3	18:34205516	FHOD3
ASRPPVTQRL	3	2:70315174	PCBP1
YMLLLWVENL	3	14:103593950	TNFAIP2
LLLRFSCMRV, WLPSLPTMALL	3	14:20846338	TEP1
RRRRHPRAAL, RRHPRAAL, RRRHPRAAL, RRRRRHPRAAL	3	8:145106939	OPLAH
WPDTTHPTCIL	3	22:20130522	ZDHHC8
STVHCMHLHEK, STVHCMHLH, TVHCMHLHEK	3	4:153249384	FBXW7
WTRRRDSPRRA	3	1:155178782	MTX1
LLSAPCPAQVL	3	19:1004896	GRIN3B
VLSSLTSLR	3	17:56448298	RNF43
CRRIWSTV	3	17:11998898	MAP2K4
RMQLCTQL	3	17:56435161--17:56435252	RNF43

FFTSVPPGF, TFFTSVPPGF	3	20:30789952	PLAGL2
HLHHLLKQLK	3	5:112175596--5:112175594--5:112175553	APC
LWRLRGGAL, RLWRLRGGAL	3	16:705360	WDR90
VREQLQQEQAL	3	8:145001588	PLEC
GRYGGGGRL	3	17:18918510--17:18918511	SLC5A10
YLSELTMEL, ISAKRNYL, ISAKRNYLSEL	3	1:39759288	MACF1
CTMPPLLH	3	17:48433967	XYLT2
HMDCLRWLK	3	12:133250289	POLE
YQTEFQAL	3	7:94539496	PPP1R9A
RQACLTFRGSW	3	10:64564892	ADO
AVAPALLESPR	3	8:8860620	ERI1
YVAEAGPPPAL	3	14:74967607	LTBP2
SQIEIEAVAI	3	8:99115125	HRSP12
SLPTRPRLLPL	3	5:137803132	EGR1
FSVAELQARL	3	19:7747293	TRAPPC5
RAVHLPELL	3	2:239050033	KLHL30
LRMKRLPVAV, LRCRPPTL, CRPPTLRM, LRMKRLPV, CRPPTLRMKRL	3	20:3215425--20:3215484--20:3218646	SLC4A11
RLLPYPFHVL, YPFHVLAL, WLSPISACPRL	3	17:40937417--17:40939870	WNK4
RQLTHVDQASF	3	7:87564497	ADAM22
LLSEGLDELPA, LLSEGLDEL	3	20:37555116	FAM83D
TRPGGWNRM	3	1:36935323	CSF3R
KRERMMLPV	3	18:34205516	FHOD3
AEGRAGARGL	3	10:100186986	HPS1
STAKSASSRM	3	2:43452512--2:43452623--2:43452682	ZFP36L2
ARPVSAAL	3	7:75677504	MDH2
RLPAPFRV	3	5:112175484--5:112175346--5:112175424	APC
RLKTFGVPL	3	1:204228411	PLEKHA6
LRARWSTSF, LRSLQRSPAL	3	22:31485922	SMTN
RQNGYRAL, RQNGYRALM	3	9:5897631	MLANA
SLPGPCASLL	3	3:51417604	DOCK3
MLLMNLATA, LLMNLATA	3	19:17000632	F2RL3
YLPGSDPDPTL	3	16:29908433	SEZ6L2
RQPCAHGL	3	19:39330959--19:39330958	HNRNPL
QPHVAHAAAAL	3	2:43452244--2:43452491--2:43452464	ZFP36L2
SRAASLLFSI	3	7:150698398	NOS3
LLARALPPDV	3	22:24121378	MMP11
ILPRKVLQMDF	3	5:112175756--5:112175752	APC
MEHDVALRREG	3	7:100285170	GIGYF1

ARSCIQAYF	3	5:64905248	TRIM23
RLRDEQLNSPY	3	15:102264476	TARSL2
AERTLACVCAL	3	19:17837425	MAP1S
RAPPPACHWL	3	17:56448298	RNF43
YSKDSGLL	3	15:45003781	B2M
LQMVPCSM	3	3:168833257	MECOM
LANPVLPNEVL	3	19:45867259	ERCC2
HQLLTSGMELK	3	4:153245446	FBXW7
SPRRGSSPA	3	1:17265560	CROCC
TPPPPPLLLLL, TPPPPPLLLL	3	1:109792735	CELSR2
ASAWLTVL, ASAWLTVLGL, VASAWLTVLGL	3	1:228504670	OBSCN
HEVFSAPAV	3	4:1165130	SPON2
SRAECGSTRSL	3	1:16462199	EPHA2
CVCACPGRDRR, CVCACPGR	3	17:7577121	TP53
WPQCPVAERA, CPVAERAVETI, CPVAERAV, CPQGWPQCPV, WPQCPVAERAV	3	2:227661664	IRS1
SAVGDPEL	3	17:40706906	HSD17B1
TAACPMTACSI	3	22:20130515--22:20130522	ZDHHC8
SYLWPSRTL	3	14:89878534	FOXN3
APQQRASF	3	1:204120952	ETNK2
FEVHVCAC	3	17:7577120	TP53
SQAPPGSQSIM, SQSIMMSWM	3	1:204228411	PLEKHA6
CTMPPLLHPQW	3	17:48433967	XYLT2
TAILAITL	3	18:34205516	FHOD3
SPRKVDPV	3	5:176026120--5:176026138--5:176026129--5:176026124	GPRIN1
AELQARLA, AELQARLAA	3	19:7747293	TRAPPC5
SLHPSLPKL, LLLSLHPSL	3	2:204305619	RAPH1
CARGPLRPPV	3	10:135186806	ECHS1
DEAGRSAV	3	17:40706906	HSD17B1
FLPGSTPSL	3	16:84213651	TAF1C
WLPSLPTMAL, WLPSLPTMALL	3	14:20846338	TEP1
IERSSLTVHQI	3	5:178358763	ZFP2
YTYRRPLQTHY, QTHYGYINVK	3	1:76779647	ST6GALNAC3
FTLRKNLSSLL	3	2:202151290	CASP8
KACAGLWPW	3	20:23066469	CD93
LETMLHGQVM	3	15:42371752	PLA2G4D
WAPAPGSPRL	3	22:20130522--22:20130515	ZDHHC8
FTKEASKKAM	3	7:100285170	GIGYF1
SLASCASVA	3	1:150530506--1:150529443	ADAMTSL4
FQNEIFGSTV	3	7:91732038	AKAP9

QAHPQVPAL	3	3:114058003	ZBTB20
REQLQQEQAL, REQLQQEQALL	3	8:145001588	PLEC
FGVPLGSIL, FGVPLGSILCL	3	1:204228411	PLEKHA6
TRVLNSSKPPL, WRVGPKTRVL	3	14:75230947	YLPM1
QRMQQDSTRTV	3	2:95537572	TEKT4
GEFLLCLPG	3	10:100186986	HPS1
SQFTLALPMWL, FTLALPMWL	3	6:10557410	GCNT2
RRAPRSPFL	3	7:100283942--7:100285170	GIGYF1
MMDPHENILL, YMMDPHENI, RTTNYDFSLYM, YMMDPHENIL, YMMDPHENILL	3	5:160753376	GABRB2
EVVRHCPHHER	3	17:7578406	TP53
SYDTYTPPI	3	11:31812317	PAX6
REAPGKRRRL	3	10:17659149	PTPLA
RMMLPVRAR	3	18:34205516	FHOD3
RRCPRLPGV	3	10:61828580	ANK3
NYIISRNCQQL, SRKRIIVINYI, YIISRNCQQL	3	10:63958149	RTKN2
SRFWLALQA, ARRESRFWL	3	18:34205516	FHOD3
LVASISAV	3	1:22191454	HSPG2
IAHFFRISLF, HFFRISLF, VQIAHFFRI	3	3:40529660	ZNF619
SPRPRLLTM	3	7:97933623	BAIAP2L1
GRADLIRLLL, GRADLIRL, GRADLIRLL	3	19:33098632	ANKRD27
MMRATGPPPSL	3	19:39330959	HNRNPL
SLLSRMPA, LLPRALPSL, MLLPRALPSLL, MLLPRALPSL	3	18:34205516	FHOD3
ASYLWPSRTL	3	14:89878534	FOXN3
VRGPASPM, CRRVRGPASPM, RRVRGPASPM, VRGPASPMGM	3	12:6657974	IFFO1
FLHVFFFLHFL, FLHVFFFL, HVFFFLHFL	3	2:230661315	TRIP12
HVAAVCCVQY	3	4:153247289	FBXW7
YLDKFVQRLL, LLYVYIIET, LLYVYIIETNV, RLLYVYII, YLDKFVQRL, FVQRLLYV, SLYLDKFV	3	18:12699829	CEP76
RDAVASAWLTV	3	1:228504670	OBSCN
TSAPSAQQSL	3	3:114058003	ZBTB20
SGFWLPPPM	3	8:120220776	MAL2
SRNDFEWVY	3	14:100625902	DEGS2

AEAAAVVTAAF	3	7:100285170	GIGYF1
GRRHLQPHDAL	3	7:42005678	GLI3
RTRSPGRAEV	3	22:38122462	TRIOBP
SREWKVKFPEL, SREWKVKF	3	15:45003781	B2M
SRMASTSCAA, SRMASTSCA	3	2:43452623--2:43452512	ZFP36L2
CIYRLTWGRRR, CIYRLTWGRR, CIYRLTWGR	3	7:102715804	ARMC10
FLAVGGPWAT, FLAVGGPWA	3	X:153688565	PLXNA3
LRHPKVHL, LRHPKVHLNTM	3	5:112175351--5:112175346	APC
RAVPHLSY, RAVPHLSYHRL	3	2:43452491--2:43452464--2:43452439	ZFP36L2
SYKQDRDITL, SYKQDRDITLL	3	3:136287655	STAG1
VRRPWNSF	3	16:50745399	NOD2
SQLAGIPL	3	3:195453018	MUC20
NEPWYKLFSEL	3	10:97135757	SORBS1
FSAEKTPLPL	3	7:97933623	BAIAP2L1
MALLLLRFSCM	3	14:20846338	TEP1
KQLKPSEKYLK	3	5:112175596--5:112175552--5:112175594	APC
MAHPPLSTV	3	1:55081757	FAM151A
SLLERVLFY, MSLLERVLFY	3	3:3067806	CNTN4
IIMANMYQWI, NMYQWIRV, SRQKFPFDLII	3	14:96761299	ATG2B
TTSSTSLSF	3	19:46269076	SIX5
LTAPLACLHL, LTAPLACL	3	12:121432118--12:121432115	HNF1A
AQDGGAGQGAL	3	8:146033347	ZNF517
TEADSKEAY	3	14:32561296	ARHGAP5
ISMTTISRATW	3	17:48433967	XYLT2
MPPPPRRPL	3	11:45955607	PHF21A
LLPPPTTTAGL	3	11:118770651	BCL9L
YWKEDLPM	3	X:84526620	ZNF711
STLWGVRMTSL	3	17:17119709	FLCN
RQMGSSLLV, KRQMGSSL, QMGSSLLV, VSMKNPYHLL, KRQMGSSLL	3	10:63958149	RTKN2
DRFVARKL	3	12:113376388	OAS3
WPWRGRRPP, WRGRRPPPPL, GRRPPPPL, WPWRGRRPPP, WPWRGRRPPPP	3	19:4491824	HDGFRP2
AEASTGAWTAE, AEASTGAWT	3	18:34205516	FHOD3
MSLSWGTALM, SLSWGTAL	3	17:25910016	KSR1
VAHAAAALLLF	3	2:43452342--2:43452439--2:43452464	ZFP36L2
SEAPGAQY	3	19:12996740	KLF1
RRNLQRNL, RRNLQRNLKVL, KRRNLQRNL, RRNLQRNLKV	3	15:64967247	ZNF609

VAHQLHVL	3	13:21562948	LATS2
CPAQVLLRL, CPAQVLLRLL	3	19:1004896	GRIN3B
TEVPSLTAL	3	2:1481231	TPO
RSKTLHHLL, SRSKTLHHL, MPPSRSKTL, SRSKTLHHLL	3	5:112175606--5:112175605	APC
STWHVAHTFL, STWHVAHTFLL, HVAHTFLLM, TWHVAHTFLL	3	11:57185284	SLC43A3
RPIRTGPS, TPRPIRTGPSL, QPPTPTPRPI, RPIRTGPSL, TPRPIRTGPS	3	19:4491824	HDGFRP2
YHRLLPLWAAL	3	2:43452342--2:43452464	ZFP36L2
LYDRGPPL	3	19:49850473	TEAD2
MQLELASL	3	9:139694569	KIAA1984
WSPTLPRTPLL	3	1:36636587--1:36636626	MAP7D1
FSTLPLPNLRM	3	12:56478854	ERBB3
YEGAATATDIV	3	6:166571895	T
MPPSGLRLLLL	3	19:41858921	TGFB1
SLGRSFLPL	3	16:2059622	ZNF598
RQYWEALGA	3	19:622336	POLRMT
RRAAAPTQQL	3	1:228559450	OBSCN
MEGPAPVSSF	3	14:23549379	ACIN1
GRAPLEGMLL	3	18:34205516	FHOD3
GVWLWAGWTV	3	14:20846338	TEP1
TTSFPFLSGL	3	16:30736370	SRCAP
LRFSGGPPGL	3	17:18181545	TOP3A
RRCPGGRV	3	17:3627473	GSG2
TAVPAFTPLSA	3	1:204228411	PLEKHA6
FRDQPVFFENL	3	14:96761299	ATG2B
HPKVHLNTM, HLNTMFRR	3	5:112175350--5:112175346--5:112175351	APC
HRCRGWWL	3	19:55815035	BRSK1
APGKRRRLGF, APGKRRRLGFL	3	10:17659131--10:17659149--10:17659149--10:17659131	PTPLA
RSWTSKGWRR	3	7:100283942--7:100285170	GIGYF1
SPRRAGPTALS, SPRRAGPTA	3	1:155178782	MTX1
SRRAARASTA, RRAARASTA	3	2:43452623--2:43452512	ZFP36L2
RRPGWKGHHRV	3	18:34205516	FHOD3
SSCMGGMNWR	3	17:7577539	TP53
SVATLGPL, WVSVATLGPL, RAQWVSVATL	3	9:137688741--9:137693829--9:137677855	COL5A1
GRFAVLGTM	3	1:150530506	ADAMTSL4
KTFGVPLGSI	3	1:204228411	PLEKHA6
WELCRAAPG	3	1:200880978	C1orf106
FLARSWNTM, LARSWNTM	3	3:77657038	ROBO2
GIFTFRFWV, FTFRFWVEKL	3	18:12699829	CEP76

RAFMQGQLGV	3	8:145738768	RECQL4
VAMMITAAHV	3	X:135956571--X:135956575	RBMX
SAMAATTTTTI, MAATTTTTI, MAATTTTTIF	3	1:176525981	PAPPA2
SMNRRPILTI	3	17:7577548	TP53
RERMMLPV	3	18:34205516	FHOD3
SARRSCVV	3	19:49611319	SNRNP70
VAAARGLLL	3	16:28847356--16:28847350	ATXN2L
ASVPCRPM	3	1:204228411	PLEKHA6
AASPSGLRAPV	3	20:23066469	CD93
RALPSLLSR, RALPSLLSRM	3	18:34205516	FHOD3
LLINFHSA, FLLLINFHSA, KRDKLPFLL, KLPFLLLI, KRDKLPFLLL	3	10:63958149	RTKN2
FLATPPPL, FLATPPPLL	3	2:60688969	BCL11A
RRKKKCVCYI, CRRKKKCVCYI	3	10:114925333	TCF7L2
WRVGATAAL	3	15:41099899	ZFYVE19
MMAFYLKYT, QIFMMAFYL, FMMAFYLKYT	3	18:12699829	CEP76
LEHDGGCVPI	3	7:149517978	SSPO
SWSPPCQSL	3	1:67390426	WDR78
GRAPPVVL, GRAPPVVLRL	3	1:151318741	RFX5
ATTRCATAAL	3	16:2059622	ZNF598
TEVVRHCPH	3	17:7578406	TP53
RPSTRFLTVPV	3	7:139167934	KLRG2
MLLMNLATADL	3	19:17000632	F2RL3
RSLARASL	3	6:30863251--6:30864517--6:30864547	DDR1
FLRAGSEVLAA	3	10:71906150	TYSND1
LPASAPTSPL	3	22:46449891	C22orf26
HPLERRGGTTA, HPLERRGGTT, HPLERRGGT	3	2:227661664	IRS1
FFTSVPPGFKM	3	20:30789952	PLAGL2
TTHPTCIL	3	22:20130515--22:20130522	ZDHHC8
VLLSCARGPL	3	10:135186806	ECHS1
FPGCTPPAY, FPGCTPPAYLL	3	19:17837512	MAP1S
SAMPSAMGVA, TSAMPSAM, SAMGVALPT	3	1:204228411	PLEKHA6
GRLTAIKLF, GRLTAIKL	3	4:115544340	UGT8
EMRAGGCCHAL	3	10:46999601	GPRIN2
SRAPCRPPPV	3	18:34205516	FHOD3
LRRVGGQRPL	3	8:145106939	OPLAH
ARFFPITPPVW	3	17:56435161--17:56435252	RNF43
LPPPPPPPV	3	10:105110740	PCGF6
SYLLLIQML, SYLLLIQM	3	14:23549379	ACIN1

RARGGTTI	3	12:122242658	SETD1B
VRWAAASKL	3	19:52715982	PPP2R1A
RYPVQRLPF	3	2:242674803	D2HGDH
WVMELEWLI, WVMELEWL	3	18:12699829	CEP76
CRPACTCI	3	17:48433967	XYLT2
FRRYKVLREI, FRRYKVLREIL, RRYKVLREIL, RRYKVLREILT, SNFRRYKV, RRYKVLREI	3	21:38302646	HLCS
SVMSCSLL	3	17:48433967	XYLT2
GRTLLVTCIL, LYHRWIVTSM, AYLGRTLLV, AYLGRTLL	3	10:890939	LARP4B
RTDCLLAVVI	3	5:57753012	PLK2
RMKKWAPLM	3	7:100285170--7:100283942	GIGYF1
LGDPFHPL	3	19:10116508	COL5A3
FRARILCL	3	3:40529660	ZNF619
NFSAHLLM	3	10:94243045	IDE
ALMVVPAL, ATAGAPPAL	3	7:100285170	GIGYF1
LELIQTINNIV	3	8:68116980	ARFGEF1
FASLRPRSVV, FASLRPRSV, FASLRPRSVVI	3	16:28847356--16:28847350	ATXN2L
CPPAPTSCGPT	3	8:120220776	MAL2
RQSAILYVHRI	3	4:436610--4:435686	ZNF721
MERCTHCTGSC	3	16:2059622	ZNF598
RRGGPPSPPL	3	17:56435161	RNF43
CSGVLSGFW, LSGFWLPPPMF	3	8:120220776	MAL2
KRDQCFQMM	3	11:124845049	CCDC15
WLPPPMFLYLY, WLPPPMFLY	3	8:120220776	MAL2
FSWVGGGGTRL	3	20:23066469	CD93
FTHSQTQI, FTHSQTQIKGV	3	15:34064243	RYR3
VRLARVRRA	3	2:43452439--2:43452244--2:43452464	ZFP36L2
YTDPSSLQKHV	3	12:57861838	GLI1
LRAQGCNPSL	3	19:4816465	TICAM1
TQLARFFPIT, MQLCTQLA	3	17:56435161	RNF43
KRYCILQV, SRKRYCIL, SRKRYCILQV	3	10:63958149	RTKN2
RADLIRLLL, RADLIRLLLK, RADLIRLL	3	19:33098632	ANKRD27
KRRRLGFLATA, RRRLGFLA, KRRRLGFLA, KRRRLGFLAT, RRLGFLAT, RRLGFLATA, RRRLGFLAT, RRRLGFLATA	3	10:17659131--10:17659149--10:17659149--10:17659131	PTPLA
SRPPVTQRLV, SRPPVTQRLVV	3	2:70315174	PCBP1
SARGAPSCHL	3	16:2059622	ZNF598
IRSEGDQL	3	19:51843808	VSIG10L
YFADMERHHIL	3	3:25668727	TOP2B

FSHSGSLQI	3	19:12256527	ZNF625
SGHEGILAL	3	11:76751542	B3GNT6
QRRPGPSPEAL	3	19:17439320	ANO8
PWKGICWKW, CWKWPVTV	3	10:29760116	SVIL
FPITPPVWHI, TPPVWHIL	3	17:56435161	RNF43
SVHLIVLRVVR, VVRLPAPFR, LIVLRVVR, VLLSVHLIVLR, VVRLPAPF, LSVHLIVLR, LLSVHLIVLR, HLIVLRVVR	3	5:112175346--5:112175351--5:112175350	APC
FHPWTTDM	3	2:171572940	SP5
KMMELEWLI, KMMELEWL	3	18:12699829	CEP76
KYSRWIFLF	3	5:112175756--5:112175752--5:112175760	APC
TEYKLVVVGAC	3	12:25398285	KRAS
MAVKRIWSTV	3	17:11998898	MAP2K4
LPSAPSGGPL	3	1:228559450	OBSCN
LAMKIGGVSL	3	5:72199545	TNPO1
RASPARASPAL	3	11:117789327--11:117789342--11:117789327	TMPRSS13
STALKIWLV, KIWLVLISPL	3	6:30863251--6:30864517--6:30864547	DDR1
ARMGGAASF	3	7:91732038	AKAP9
CPKCSTPA, CPKCSTPATL, TPATLTLAV	3	19:10202224	C19orf66
LAWARAHLAV	3	14:104642560--14:104642401--14:104642555	KIF26A
MMITAAHVTDM, LMVEAVAM	3	X:135956575	RBMX
VRLPAPFRV	3	5:112175424--5:112175351	APC
STVHCMHL	3	4:153249384	FBXW7
RVTQALQASL	3	9:137693829--9:137688741--9:137677855	COL5A1
TEYKLVVVGAS	3	12:25398285	KRAS
RTPTTRLM	3	17:48433967	XYLT2
YVDPSGGDHFC	3	18:48591919	SMAD4
LRFSCMRVASY, LRFSCMRV	3	14:20846338	TEP1
YTVSPRKV	3	5:176026120--5:176026138	GPRIN1
GPRTTTATM	3	16:2059622	ZNF598
KASSPGHPL	3	15:79750586	KIAA1024
SRQKFPFDL, SRQKFPFDLII	3	14:96761299	ATG2B
CEICVKSH	3	18:34205516	FHOD3
RRIPPPHPRCL	3	12:123351892	VPS37B
LPRAGTGTRI, YPTRILPRA, RIAAAPTL, TRIAAAPTL	3	19:10202224	C19orf66
LTIQNAAF	3	13:20638677	ZMYM2
FSHKLYLKNM	3	19:52888438	ZNF880
WMVPWGMEA	3	14:104642560--14:104642401	KIF26A

RNLPPKVVLR	3	5:112175350--5:112175346--5:112175351	APC
STPAAGSSL	3	18:3456550--18:3456403--18:3457371	TGIF1
RERLYQDTSLM	3	13:20638677	ZMYM2
PPLPPPPPTPM, LPPPPPTPM, GRRPPPPL, WRGRRPPPPL, WPWRGRRPPPP, LPPPPPTPMCL, WPWRGRRPPP	3	19:4491824	HDGFRP2
LTHPAHQPL	3	1:27105931	ARID1A
NQFVVADLLL, FVVADLLLL, NQFVVADLL, NQFVVADLLLL	3	1:154304153	ATP8B2
YVSRTLVVFV, SRTLVVFVSI, YVSRTLVV	3	4:141888889	RNF150
TPSPTTRV	3	19:39330959--19:39330958	HNRNPL
RTPLFPLL	3	5:137803132	EGR1
SYPSSLSV, SYPSSLSVFSV, SVFSVPGYHLY, KYSYPSSLSVF, KYSYPSSL, SYPSSLSVF, YSYPSSLSV, YSYPSSLSVF	3	3:169802469	GPR160
SCSMRRNMI	3	16:2059622	ZNF598
GRVGKSAL	3	1:115258745	NRAS
RRPAAGGDREL	3	19:17932138	INSL3
RRMHVCGGSLL, RRMHVCGGSL	3	16:1273490	TPSG1
FSVPGYHLY, SVPGYHLYY, FSVPGYHLYYF, YSYPSSLSVF, SVFSVPGYHLY, SYPSSLSVFSV, KYSYPSSLSVF, SYPSSLSVF, FSVPGYHLYY, SVPGYHLYYF	3	3:169802469	GPR160
RERVDLSKL	3	5:112175596--5:112175594--5:112175553	APC
VLDINDNPPL	3	4:134071648--4:134071649	PCDH10
RRTRKQTKVL, GRRTRKQTKVL, RRTRKQTKV	3	5:137803132	EGR1
LSFWPGGY, LSFWPGGYPAY	3	15:45003781	B2M
CASLLSTL	3	3:51417604	DOCK3
WRAWRRFPL, WRAWRRFPLL, WRAWRRFPLLM	3	22:18300932	MICAL3
WMTSLISSL, MTSLISSL, SRWMTSLISSL	3	16:14346300	MKL2
LPGEGGDGTPA	3	9:25677953	TUSC1
REMRAGGCCHA	3	10:46999601	GPRIN2

LRAPSSFHPLV, LRAPSSFHPL	3	1:228559450	OBSCN
YSSGFCNIAV	3	11:108117798	ATM
FRRPHSCLA, FRRPHSCLADV, FRRPHSCL	3	5:112175346--5:112175351	APC
MPPHVVLTF	3	16:89167094	ACSF3
LQIAEIKDW	3	5:112175212	APC
FVMPLHHGL, FIFFVMPL	3	5:72199545	TNPO1
AESCSSAPA	3	2:56411817	CCDC85A
WPSRRQPC, WPSRRQPCA	3	19:39330958--19:39330959	HNRNPL
LYQDTSLMMIV, LYQDTSLMMI, RLYQDTSLMM, LMMIVLTI	3	13:20638677	ZMYM2
SEALLAQP, SEALLAQPPC, SEALLAQPP, SEALLAQPPCC	3	19:36258938	C19orf55
LPRQYWEALGA, LPRQYWEAL, LPRQYWEA	3	19:622336	POLRMT
PRSTWHSVLM	3	16:50745399	NOD2
SPSLASCASV	3	1:150530506--1:150529443	ADAMTSL4
AYPSAFPSA	3	1:29138975	OPRD1
SSRQHRSAL	3	16:50745399	NOD2
FTDIIKLFNEM, FTDIIKLF	3	22:25315953	SGSM1
LEATALGA	3	16:2059622	ZNF598
CAMDSLRPVRL, LQKYPQAAA, LQKYPQAAAV, AMDSLRPV	3	12:121432118--12:121432115	HNF1A
KQIHTGEK	2	16:31926853--19:37643428	ZNF267--ZNF585A
ISITFFCLF	2	X:49069216--3:53804490	CACNA1F--CACNA1D
SRVRTSFF, YPSSRVRTSFF	2	X:101912485--X:101971767	GPRASP1--ARMCX5-GPRASP2, GPRASP2
YALRSFNYEQL	2	5:140745458--4:134072889	PCDH10--PCDHGA5
ARIGRILCL	2	2:166848843--2:166245228	SCN2A--SCN1A
LLDPSQNNL	2	19:12246636--1:247202765	ZNF670--ZNF20
LEDLLMGM	2	6:150342081--6:150267749	ULBP2--RAET1L
KAFFCPVY	2	19:12384448--19:12502590	ZNF44--ZNF799
VPPPPHLL	2	16:24942180--7:29874359	WIPF3--ARHGAP17

LEAQEYHL	2	19:54823313--19:54783825	LILRB2--LILRA5
DTAGREEY	2	1:115256529--12:25380276	NRAS--KRAS
YSGNLLSF, LSFSRRGM, SVYSGNLLSF	2	2:97749725--2:96078466	FAHD2A--FAHD2B
CSVGNTLVL	2	2:178482721--2:178416783	TTC30A--TTC30B
HRDLAAQNILL, HRDLAAQNIL	2	4:55956220--5:180043464	FLT4--KDR
FTQPEYTVFV, TQPEYTVFV, FTQPEYTVF	2	5:140175915--5:140250051	PCDHA11--PCDHA2
FILVRNPMNV	2	19:37130434--19:37618972	ZNF420--ZNF461
FRPGKVLCF, FRPGKVLCFL	2	X:70597459--9:32634800	TAF1--TAF1L
YELVVTAW	2	5:140175831--5:140236912	PCDHA10--PCDHA2
TRRRHIEI, RYLTRRRHI, NRYLTRRRHI, RYLTRRRHIEI	2	7:27185425--7:27194742	HOXA7--HOXA6
EQTPLTTLY, AEQTPLTTLY	2	12:112228337--15:101434258	ALDH1A3--ALDH2
HQRLHTGKSL	2	19:58018106--1:38304394	ZNF773--MTF1
CSATATLHV	2	5:140564088--5:140559596	PCDHB16--PCDHB8
RIPFRMGLY	2	5:140237500--5:140188642	PCDHAC1, PCDHAC2, PCDHA2, PCDHA12, PCDHA5, PCDHA11, PCDHA1, PCDHA7, PCDHA6, PCDHA8, PCDHA13, PCDHA10, PCDHA9, PCDHA3, PCDHA4--PCDHA10
SSLFLFLVLLF, FLVLLFVAV, FLFLVLLFVAV, SLFLFLVLLFV, SVSSLFLFLV, FLFLVLLFV	2	5:140531943--5:140595806	PCDHB6--PCDHB13
NPMNVRNV	2	19:37440746--19:37618972	ZNF568--ZNF420

RQASKALLPV	2	2:241631398--2:241622187	AQP12B--AQP12A
LSSPTRPL	2	19:40392496--12:122618185	MLXIP--FCGBP
FVLDKNDNAPA	2	5:140209348--5:140188444	PCDHA6--PCDHAC1, PCDHAC2, PCDHA2, PCDHA12, PCDHA5, PCDHA11, PCDHA1, PCDHA7, PCDHA6, PCDHA8, PCDHA13, PCDHA10, PCDHA9, PCDHA3, PCDHA4
KAFSHSSF	2	19:12383358--3:44762890	ZNF502--ZNF44
TQHQIIHI	2	19:37619881--10:38344518	ZNF33A--ZNF420
KIIHNREK	2	19:22271382--4:437702	ZNF721--ZNF257
LSFSRRGMK	2	2:96078466--2:97749725	FAHD2A--FAHD2B
FIDKVWFL, SFIDKVWFL	2	12:52777579--12:52992893	KRT84--KRT72
SELARINYL	2	5:118469808--15:51828428	DMXL1--DMXL2
VLLSSSFI, ALAVLLSSSFI	2	1:89618400--1:89659035	GBP4--GBP7
YRDMMLENY	2	7:55990945--19:53652530	ZNF347--ZNF713
SGQNTWLSY, GQNTWLSY, DSGQNTWLSY	2	5:140802601--5:140503375	PCDHB4--PCDHGA11
CNKCGKAFR	2	19:52869505--19:40520762	ZNF610--ZNF546
EIIHTGERPY	2	19:11891955--22:24086597	ZNF70--ZNF441
AQHWIIHTGEK	2	19:52825675--19:52794000	ZNF766--ZNF480
REIQTAVCLLL, IQTAVCLLL	2	6:26043588--6:27775387	HIST1H2BL--HIST1H2BB

FFYTTTTLII, FFYTTTTL, FYTTTTLII, IFFYTTTTLI, FFYTTTTLI, IFFYTTTTL, FYTTTTLIIAF, FYTTTTLI	2	11:57191485	SLC43A3--SLC43A3, PRG2
HEITHTGEKPY	2	19:12461545--19:12637432	ZNF442--ZNF564
RVYTGEKPY	2	19:38189537--12:133587135	ZNF607--ZNF26
QEMSWIADT, GEQEMSWIADT, GEQEMSWI, GEQEMSWIA, QEMSWIADTYA	2	X:120182230--10:88827869	GLUD1--GLUD2
TLHQIIHTGEK	2	19:38126321--19:57089719	ZNF470--ZFP30
ILHNFNLK	2	10:96612560--10:96748674	CYP2C9--CYP2C19
ALQAFEFHV, YEALQAFEFHV	2	5:140475969--5:140481822	PCDHB2--PCDHB3
SGHNTWLSY, ADSGHNTWLSY, ADSGHNTW, VDADSGHNTW	2	5:140731619--5:140779486	PCDHGB5--PCDHGB1
SRRGMKSSV, SRRGMKSSVRL	2	2:97749725--2:96078466	FAHD2B--FAHD2A
MYQVPAQL, MYQVPAQLGHL	2	20:23667825--20:23729753	CST4--CST1
FQDEEGKEYV	2	X:70617214--9:32632006	TAF1--TAF1L
RSHTGEKPYK	2	19:20229196--20:45130930	ZNF334--ZNF90
ATYVAIQAV	2	1:229568356--7:5568319	ACTA1--ACTB
TAPPLLLL	2	22:39427885--17:7751828	APOBEC3D--KDM6B
HQHIHTGER	2	7:150094966--16:30795140	ZNF775--ZNF629
KLHRDVMLENF	2	19:44585249--19:44844613	ZNF284--ZFP112
LEEKVCAL	2	12:14591091--20:25434140	NINL--ATF7IP
NADKTWYL	2	1:158152835--1:158325784	CD1E--CD1D
IVHQQTHTGEK	2	19:52496818--19:52519847	ZNF615--ZNF614
GLHCKHAK	2	4:69811043--4:69964387	UGT2B7--UGT2A3
TVHQITHTGEK	2	X:47835717--19:37118082	ZNF182--ZNF382
MMMCYLFH, MMMCYLFHMY, MMMCYLFHM, DMMMCYLF, MMMCYLFHMYV	2	15:34150114--19:39071143	RYR3--RYR1

YLIITICA, YLIITICAV, LIITICAV	2	5:140203464--5:140167982	PCDHA1--PCDHA5
FMYTIAGLCML, YTIAGLCML, FMYTIAGL, YTIAGLCMLKL, YTIAGLCM, FQFDTSFMYTI, FMYTIAGLCM	2	3:113329873--11:117062612	SIDT2--SIDT1
FTLEKGLMSA	2	19:57910688--19:58290302	ZNF548--ZNF586
YRKFTSASNV, FTSASNVWSY, TSASNVWSY, FTSASNVW, YRKFTSASNVW, SASNVWSY	2	3:134960059--1:23235584	EPHB1--EPHB2
SRRFKEPWF	2	19:30935604--18:74154167	ZNF516--ZNF536
YKFEECGKAF	2	19:19905752--19:21991304	ZNF506--ZNF43
TRIPFRVGLY, TRIPFRVGL	2	5:140188624--5:140263705	PCDHAC1, PCDHAC2, PCDHA2, PCDHA12, PCDHA5, PCDHA11, PCDHA1, PCDHA7, PCDHA6, PCDHA8, PCDHA13, PCDHA10, PCDHA9, PCDHA3, PCDHA4--PCDHA13
ALSSKALV	2	5:140590109--5:140531462	PCDHB6--PCDHB12
SPTNVMSV	2	8:145999587--6:28358666	ZSCAN12--ZNF34
SFFPKSSPLF, SFFPKSSPL, RSFFPKSSPL	2	8:26221272	DOCK5, PPP2R2A--PPP2R2A
HTCKICGK	2	19:11917583--19:11833351	ZNF491--ZNF823
HQLHREIEI	2	19:57743562--17:8110524	AURKC--AURKB
YMGEISTT, FRVGLYMGEI	2	5:140176432--5:140209559	PCDHA2--PCDHA6
MMKNQRVQLMH, VQLMHQMK, RVQLMHQMK	2	X:69595981--5:154395374	KIF4B--KIF4A

CEKSCCSCCPV	2	16:56667311--16:56660437	MT1M--MT1E
MQTHTGEKPY	2	19:9868344--19:14829800	ZNF333--ZNF846
RRRGKNKVAV, RRGKNKVAV	2	2:178095799--7:26225093	NFE2L3--NFE2L2
FSRRGMKSSVR	2	2:96078466--2:97749725	FAHD2A--FAHD2B
IRLVRVFHIF, VRVFHIFKL, IRLVRVFHI	2	12:5154539--12:4920259	KCNA6--KCNA5
FSAGKWICV	2	10:96612495--10:96748609	CYP2C19--CYP2C9
ATVTLTVAM, LSATVTLTVAM, SATVTLTVAM	2	5:140794744--5:140712241	PCDHGA1--PCDHGA10
DTAGLEEY, ILDTAGLEEY	2	1:115256529--12:25380276	NRAS--KRAS
VVVGACGVGK, VVGACGVGK	2	12:25398285--1:115258748	NRAS--KRAS
FPGPKGPL	2	9:137688741--6:33141280	COL11A2--COL5A1
FTTNSMNIPAL, MGFTTNSM	2	20:13071817--14:78036765	SPTLC3--SPTLC2
SSAPCTELV	2	5:140516715--5:140559320	PCDHB8--PCDHB5
VVYPWTQRLF, PWTQRLFESF, VVYPWTQRL, VYPWTQRLF, WTQRLFESF, VYPWTQRL	2	11:5255410--11:5247996	HBB--HBD
LQFDGALNVDL, LQFDGALNV	2	2:132237994--2:130951687	TUBA3E--TUBA3D
ITYVSLIY, ITYVSLIYDAF	2	1:39749132	MACF1
RPPGSPGQST, SPGQSTQLPL, SPGQSTQL	2	17:8296383	RNF222
LPGRLLQSCI, LPGRLLQSCIL, FPCLRLLPGRL	2	6:116836954	FAM26E
HLMVEAVAM	2	X:135956575	RBMX
YRLQARIAHQI, ARIAHQIQEL, LQARIAHQI	2	19:11100016	SMARCA4
FFYRCICL	2	7:20371449	ITGB8
ARAGACWV	2	19:7592423--19:7592444	MCOLN1
QRRPHLNL	2	1:203144870	MYBPH
RVFSVAELQAR	2	19:7747293	TRAPPC5
FSPPPAACSPA	2	6:45390355	RUNX2
TRIMKTDF, TRIMKTDFAGL	2	4:20258327	SLIT2
GELELLLVAA, RVRAGELEL, RVRAGELELL, RVRAGELELLL, GELELLLV	2	15:41803754	LTK
FQIHTPPA	2	14:58698938	ACTR10
FASDNFLGI	2	16:11862325	ZC3H7A
VALWQQQICAI, VALWQQQI	2	17:66899514	ABCA8

GRRGLCVLRRL, GRRGLCVL, RRGLCVLRRL	2	10:3214942	PITRM1
YKKERICKLSM, YKKERICKL, ERICKLSM	2	3:52729561	GLT8D1
YLNSSGSY, SYLNSSGSYWV, YLNSSGSYWV	2	4:113353573	ALPK1
RPRPPLPPRL, LGRPRPRPPL, RPRPRPPL	2	16:88599701	ZFPM1
FSNLVRLM, VRLMGPWHLCM, RLMGPWHL, VRLMGPWHL, RLMGPWHLCM, GFSNLVRLM	2	16:30736371	SRCAP
YLSFQEVREYM, FLDILNYLSF, ILNYLSFQEVR, FLDILNYL	2	1:91977351	CDC7
KMISTISPW	2	12:14019044	GRIN2B
LFYKRNSTNLF, LFYKRNSTNL, FYKRNSTNL, KRNSTNLF, FYKRNSTNLF	2	3:15467851	METTL6
ELAGDQPY	2	5:76249497	CRHBP
MSLSNILMKF, SLSNILMKFRL, MSLSNILM	2	12:88524079	CEP290
RQFWFLMYPFR, SVRQFWFLMY, RQFWFLMY, ISVRQFWFLMY, RQFWFLMYPF	2	X:78426851	GPR174
HLLFEAWLL, KMMIGSQHL	2	3:77657038	ROBO2
WIYMSDNYTNV, YMSDNYTNV	2	2:71797059	DYSF
FVCEAIQRF, LEFVCEAI	2	11:14501200	COPB1
LRQNQKTPTSL	2	1:93575840	MTF2
ISREAGGGLY	2	5:178418554	GRM6
LSSLTSLR, SEEPVLSSL	2	17:56448298	RNF43
SQINLIDLA, HRITSQINL, HRITSQINLI	2	1:200573037	KIF14
CVERCPWK	2	10:29760116	SVIL
FTNQKENIPL	2	16:55860154	CES1
RSAKYIQIHGR	2	19:11917796	ZNF491
RLLPTYRPTL, FPLYSGRLL	2	11:46702223--11:46702224	ARHGAP1
FFLLITITL	2	17:34313637	CCL14
TAPPSTRMM	2	2:227661664	IRS1
NMDFTDIIKLF	2	22:25315953	SGSM1
QRTIVRTIVL, RTIVRTIVL	2	9:101900171	TGFBR1
MRHKQVKENCL	2	3:113753983	KIAA1407
SEIPPPAPA, SEIPPPAPAT	2	17:73491063	KIAA0195
LAFPPAGAPWV, GHQRSSSL, SRGHQRSSSL	2	19:17039884	CPAMD8
RQSSSLIAHQI	2	5:178311162	ZNF354B

NYFFVFSVIFF, YFFVFSVIFF	2	3:194140668--3:194140669	ATP13A3
HTSIWLLQTDF, QTDFVLDYPK, LQTDFVLDY, WLLQTDFVLDY, QTDFVLDY, LLQTDFVLDY	2	2:234581341	UGT1A8, UGT1A7, UGT1A3, UGT1A1, UGT1A5, UGT1A10, UGT1A6, UGT1A9, UGT1A4
KGFFKHTVQK, GFFKHTVQK	2	2:157185044	NR4A2
MQLDTVLTL, FLMQLDTVLTL	2	2:164466486	FIGN
FLVVFILLSSV, FLVVFILL	2	7:92733781	SAMD9
GPPARAIAT	2	1:36935323	CSF3R
MRRQQNEV, RRQQNEVVV, MRRQQNEVVV, RRQQNEVVVEL, RRQQNEVV, MRRQQNEVV	2	3:160254612	KPNA4
MSPPASRWNPL, RWNPLRRTRAL	2	16:1270496	CACNA1H
RLRQQLVCHQR	2	19:36831575	ZFP14
GRRSRAYM	2	19:35758275	LSR
LENEDQEVCML, QEVCMLFKDL	2	5:148427439	SH3TC2
RTMAGPSASL, TMAGPSASL, MAGPSASL	2	11:71948748	INPPL1
CMSSSLPPLW	2	1:156814053	INSRR
QHFAHFTELAI	2	11:47283147	NR1H3
MICHGCIVLK, GMICHGCIVLK	2	5:112176027	APC
GRHSPPTL	2	7:99818416--7:99817848	PVRIG
SQAGLGVW	2	3:48612871	COL7A1
RLLLLLLPLL, LLLLPLLWLL, LLLLLPLLWLL, RLLLLLLPL	2	19:41858921	TGFB1
RVVRLPAPF	2	5:112175351--5:112175346	APC
WLPCTHPTGL	2	19:55815035	BRSK1
IMRPSTAAA	2	12:7249400	C1RL
TPEVQGRVPT, TPEVQGRVPTL	2	11:66472274	SPTBN2
KPEEPAEV	2	4:7044380	CCDC96
TQDTTVSL, RTQDTTVSL	2	6:32811743	PSMB8
APPTPSPTTRV, APPTPSPT, RPPVWAPPT, RVWPSRRQPC	2	19:39330958--19:39330959	HNRNPL
ARPPRLLF, ARPPRLLFTSL	2	7:99817848--7:99818416	PVRIG
SQVWTAATL	2	3:51417604	DOCK3
EFQPVSLLM	2	12:24048776	SOX5
EPQESGDGSL	2	21:36042579	CLIC6
LPRKVLQMDF, LPRKVLQM	2	5:112175752--5:112175761	APC

FLLHATPQTQL, TQLPKVWDPV, FLLHATPQT	2	16:50745399	NOD2
HVQVRRKVPV	2	2:43452464--2:43452491	ZFP36L2
SYNPHTGMESL, SYNPHTGM	2	6:30624769	DHX16
LAASSSAW	2	17:8296440	RNF222
ATWMRPPRSLL	2	22:18300932	MICAL3
RRPLQPLL	2	1:36643581--1:36643530	MAP7D1
LTHPLLVSLL, LTHPLLVSL, LTHPLLVSLLL, FSTASLAKEL, LAKELTHPL	2	16:68835781	CDH1
QRWKWVSQNRL, SQNRLFNLGTM, SQNRLFNL	2	17:60742035	MRC2
QARDLRCPL, CPLTAPLACL, DLRCPLTAPL	2	12:121432118--12:121432115	HNF1A
SVVLRSPR, MASSVVLRSPR, MASSVVLR, CMASSVVLR	2	8:22054260--8:22054259	BMP1
GELTVTGM, LTVTGMTPDSL	2	6:32049373	TNXB
VRLDSMVL, VRLDSMVLL	2	8:13259103	DLC1
SWPWTRCL, RMGGRHPSWPW, RMGGRHPSW, SWPWTRCLRM	2	19:49458971	BAX
QTSTVNTF	2	3:169587528	LRRC31
WLYQESLPTL, TLFCMTHGL, TLFCMTHGLLI	2	6:10557410	GCNT2
WQDIIYGMAVL, WQDIIYGMAV, QDIIYGMAVL	2	11:22294500	ANO5
TRYWPASSI	2	19:17838753--19:17838385	MAP1S
ALLAAIWFQTY, LLAAIWFQTY	2	2:9098719	MBOAT2
AQDLAGVISAL	2	15:41099899	ZFYVE19
FQSMSWPI, SMSWPILTIFL, FQSMSWPIL	2	11:33117910	CSTF3
DPFKRLISAF	2	2:101010201	CHST10
IRRNHSSL	2	11:108032695--11:108032701	NPAT
FSSREERLWF	2	9:101900288	TGFBR1
ARPTSCSTCRM	2	9:136918529	BRD3
QRPPRSILSL, QRPPRSIL	2	6:36867371	C6orf89
LQSVHEVF	2	4:1165130	SPON2
VFPEGLGDVW	2	21:38081480	SIM2
FFTMVPYEL, NSSDGFFTM, FTMVPYEL, FTMVPYELQEI	2	1:46120890	GPBP1L1
LRMNQGKI	2	1:20231475	OTUD3
HPGACHFSGRV	2	9:18680349	ADAMTSL1
HFLEKSPLEEW, VFFFLHFL, RFLHVFFFLHF	2	2:230661315	TRIP12

KYFYKNGTV, YFYKNGTVM, YFYKNGTV, KYFYKNGTVM	2	2:210559274	MAP2
STIPVHIL, STIPVHILF, STIPVHILFF	2	17:37829317	PGAP3
WPGIACRV, WPGIACRVT	2	16:2059622	ZNF598
KDQVWAAMTCI, QQFMKDQVWAA	2	14:68221958	ZFYVE26
YQFWKNFHTL, YQFWKNFHT	2	3:46414975	CCR5
RSASPGPK	2	11:67176565	TBC1D10C
SQKRGQLCSPL	2	8:22054260--8:22054259	BMP1
CACPGRDWR	2	17:7577094	TP53
VRRSLCLKFNL, RRSLCLKF, VRRSLCLKF	2	15:32926204	ARHGAP11A
TLRMKRLPVAV, RPPTLRMKRL, LRMKRLPVA, LRCRPPTLRM, TLRMKRLPV	2	20:3218646--20:3215484	SLC4A11
RVGGQRPLPR	2	8:145106939	OPLAH
WTYCCLPSLL, WTYCCLPSLLL, WTYCCLPSL	2	2:73302665--2:73302655	RAB11FIP5
CPGRNAWWL	2	7:128852004	SMO
YMYNSSCM, YNYMYNSSCM, YMYNSSCMGGM, NYMYNSSCM, TTIHYNYMY	2	17:7577568	TP53
SPMSLSWGTAL, SPHSPGASP, SPMSLSWGT, CPASPHSPGAS, SPMSLSWGTA, SPGASPMSL, SPHSPGAS, SPHSPGASPM, MSLSWGTAL, SPHSPGASPMS	2	17:25910016	KSR1
YRFHKFHSPAL, YRFHKFHSPA, EEHYRFHKF, LEEHYRFHKF	2	2:31457617	EHD3
YSLGEFSSGR	2	11:77649828	INTS4
REGAQLKKW	2	7:123332485	WASL
ASEESLPF	2	7:149544824	ZNF862
ITSCPVTWM	2	1:147092680	BCL9
FASTRDKLNY, YSIQYVLEV	2	8:110452953	PKHD1L1
IASLSCFTCMM, MMTMAISL, FTCMMTMAI, MMTMAISLTAL, CMMTMAISL, MTMAISLTAL, CMMTMAISLTA, MMTMAISLTA, MTMAISLTA, FTCMMTMAISL, IASLSCFTCM	2	20:31384651	DNMT3B
CTNFYHHK, CLCTNFYHHK	2	5:112176027	APC
YYKDYLHCEWI	2	8:3256976	CSMD1
APSAAAAAAQV	2	X:112022477	AMOT

FISSVPSSL	2	16:84214763--16:84214674	TAF1C
CLSSLHLHER	2	1:247263870	ZNF669
RMAFEGSLAL, MAFEGSLAL	2	9:137708907--9:137707451	COL5A1
ATRSRHRTRFV, ATRSRHRTRF, RSRHRTRFV, RHRTRFVF	2	11:65628334	MUS81
LRCPAVPAA, LRCPAVPA, LRCPAVPAAA	2	3:51417604	DOCK3
KRPARSRLVPL, KRPARSRL, ARSRLVPLGSL, ARSRLVPL, KRPARSRLV, SRLVPLGSL	2	7:20371449	ITGB8
KRNHGHPQL	2	12:77449827--12:77449858	E2F7
ARSKFTGL, LRTSARSKF	2	5:133473786--5:133451703	TCF7
TYFQIHINK, GTYFQIHINK, YFQIHINKYKM	2	20:20278871	C20orf26
TTPCTSSIL	2	17:6406883	PITPNM3
LPRRSGRAAAT, LPRRSGRAAA, LPRRSGRAA	2	6:43251912	TTBK1
VPGAAAPA, APAAAALM, VPGAAAPAA, AAAPAAAAL, VPGAAAPAAA, VPGAAAPAAAA	2	17:7496122	FXR2
HSFVQLCADY, FVQLCADY	2	14:57675371	EXOC5
EQLQNLII	2	6:109787329	ZBTB24
SQNWGSLPL	2	19:13882968	MRI1
EISNNVLAGI	2	2:48059591	FBXO11
IPYMAKFVV, REIICIPYMA, QVYREIICIPY, IPYMAKFVVF, VYREIICIPYM, REIICIPYM, YREIICIPYM, REIICIPY	2	4:114262920	ANK2
KYMKHTRL, KYMKHTRLF	2	17:18149086	FLII
ETDNVDLVSEL, ETDNVDLV	2	22:37906309	CARD10
KVWDPVFFGA, TQLPKVWDPV	2	16:50745399	NOD2
FRLSRKIY	2	15:41865543	TYRO3
MKMACGKRSL	2	16:15131990	NTAN1
AVRHRGRRGPA	2	2:43452491--2:43452464	ZFP36L2
GDFWYVTCAI, AQDLGGDFWYV, DLGGDFWYV	2	4:7666085	SORCS2
RRQPCAHGLW	2	19:39330958--19:39330959	HNRNPL
RPLSLQCQSLA, LRPLSLQCQSL, RPLSLQCQSL, ERNLRPLSL	2	9:35043650	C9orf131
YTLQPLDHEEL, AESGKVYTL	2	5:140167430	PCDHA1
MPKLHFASRI	2	7:93516175	TFPI2
GRAAQLLDVRL, VRLFELSEL	2	19:19040312	HOMER3

LEIALTISPF	2	1:100889837	CDC14A
FLFSFQNHIRI, FSFQNHIRI	2	19:12692176	ZNF490
TLAQWSPVIAA, AQWSPVIAA, TLAQWSPVI, ALTLAQWSPV, TLAQWSPV	2	5:137803132	EGR1
VVDYGTRLFHL	2	5:109124646	MAN2A1
FQEEPMTGFF	2	16:28847350--16:28847356	ATXN2L
KTSTIALFK	2	8:25154087	DOCK5
LARVSVFLSM, TPPSCLARVSV, LPTPPSCLARV, ARVSVFLSM, RVSVFLSMSTL, LPTPPSCL, RVSVFLSM	2	20:3215484--20:3218646	SLC4A11
HADRGAFAL	2	7:100465746	TRIP6
FFLWRSFRLV, FFLWRSFRL, RSFRLVRSRLY, FRLVRSRL, FRLVRSRLY, WRSFRLVRSRL, FRLVRSRLYV	2	14:39746268	CTAGE5
LPSPPGLQL	2	6:149772190	ZC3H12D
VAASPRRM, LVAASPRRMCL, VAASPRRMCL, LVAASPRRM	2	15:41146715--15:41146120	SPINT1
VAELQARLAAL	2	19:7747293	TRAPPC5
FEARHLGSL	2	11:76371564	LRRC32
HREQRAAAPL	2	X:17710476	NHS
MSRAVCLPV, RAVCLPVPCPV	2	17:56729347	TEX14
TRRRSRRSTSF, RRRSRRSTSF, RRSRRSTSF	2	16:89598371	SPG7
HTHPHQPL	2	11:67812475	TCIRG1
PEWGRVSL	2	17:7462459	TNFSF13
AAALDRFVARK, KLQPRKEFVEK, ALDRFVARK, AALDRFVARKL, AALDRFVARK, FVARKLQPRK	2	12:113376388	OAS3
VRQFQLKI, VRQFQLKIAEL	2	4:6086661	JAKMIP1
QMIWAFYLK, MIWAFYLK	2	1:35972480	KIAA0319L
KLYLKNMR	2	19:52888438	ZNF880
QPLQPPTL, QPPTLQPL, APTDRLQPL, QPLQPPTLQPL, ALAPTDRLQPL	2	17:70120194--17:70119909	SOX9
ELQARLAAL	2	19:7747293	TRAPPC5
KYLLTSMYV	2	X:154088801	F8
HRPPWHGLM, LMAADRAW	2	7:128852004	SMO
AEVHGSPEH	2	19:55815035	BRSK1
HVTPKTPLPM	2	10:75407290	SYNPO2L
QENNSKLECL, QENNSKLECLL	2	1:214814828	CENPF

TYREKLSGF, YREKLSGF, WRAKISTMSTY	2	12:122018740--12:122018773	KDM2B
GLALALALA, GLALALALALA, GLALALALAL, LLWMGLALA, LLWMGLAL, GLLWMGLALA, LLWMGLALALA, GLLWMGLALAL, LLWMGLALAL	2	11:6411931--11:6411935	SMPD1
KQICIPRSLSV	2	3:170013719	PRKCI
GRALRALMAM, LRALMAMAL, GRALRALM, ALMAMALA	2	17:73501069	CASKIN2
KTQKVQMKF	2	3:178938934	PIK3CA
TEFPEILWHL	2	17:41610700	ETV4
HRRRVQCSL, RRRVQCSL	2	11:64572093	MEN1
RRMGWPPA, RRMGWPPAWM	2	14:24532703	LRRC16B
KYMSQFPKVWI, YMSQFPKVWIL, SQFPKVWI, SQFPKVWILRL, SQFPKVWIL	2	2:158142577	GALNT5
RSPCLLLLWLV	2	3:66550762	LRIG1
SEILGETSLM, SEILGETSL	2	22:29091840	CHEK2
FIFNQNFFQDL	2	11:12234929	MICAL2
CRRGLEQLL	2	19:17393530	ANKLE1
SLRKQLLLFLV	2	5:57753012	PLK2
RIDQICSL, RIDQICSLM, RIDQICSLMAV	2	17:33482349	UNC45B
SRILSPYCI	2	19:56244405	NLRP9
RIRSEGDQL, RIRSEGDQLL	2	19:51843808	VSIG10L
NEQKYADVV	2	13:114774873	RASA3
VALTSSSASI, SSASISPV, RVALTSSSASI, LTSSSASI, LTSSSASISPV, SSSASISPV, TSSSASISPV	2	15:41146715--15:41146120	SPINT1
GADGFPALA	2	2:36825137	FEZ2
LQRHVCNI, HVCNIHNK	2	3:168812978	MECOM
FPAASPSGL	2	20:23066469	CD93
LRPLHVPRV, WRTAVLLRPL	2	11:76751604--11:76751585--11:76751585--11:76751542	B3GNT6
SLDPPAQV	2	10:104175781	PSD
LLQMVPCSM	2	3:168833257	MECOM
TVIAASWRV, WRVGGPLGSL, VIAASWRV	2	1:35370731--1:35370639	DLGAP3
FHTGNTSF, HQRFHTGNTSF	2	19:52496382	ZNF615
IFMENIML	2	16:5105344	C16orf89
IINMALSEY, NIINMALSEY	2	14:91110426	TTC7B
SMQLELASL	2	9:139694569	KIAA1984
ARYCADACGEL	2	14:74058832	ACOT4

RQSIWDVI	2	2:215846946	ABCA12
CEDTRAGLW	2	8:145641328	SLC39A4
LSHLYLGCNK, ALSHLYLGCNK, YLGCNKLASF, HLYLGCNK	2	3:49756397	AMIGO3
AYIDRDRKIL, AYIDRDRKI	2	11:72301503	PDE2A
LSHQVPILF, YLSHQVPIL, YLSHQVPILF	2	13:31903690	B3GALTL
SHLILLWLL, SHLILLWL	2	22:31485922	SMTN
DEAGTPHEGW	2	1:153906129	DENND4B
CQLVQNLSLQV	2	2:223423432	SGPP2
GMNWRPILTI	2	17:7577539	TP53
RASCASSASK, ASCASSASK	2	20:23066469	CD93
ELLLVGRY	2	17:18918510--17:18918511	SLC5A10
GRDPKTPL, GRDPKTPLTL	2	19:7592423--19:7592444	MCOLN1
REFRGLSDCLV	2	X:118603984	SLC25A5
SARLLRAEM	2	15:83680287	C15orf40
FYVGWAEATV	2	16:28847356--16:28847350	ATXN2L
NRVPRAATSRM, SRMERIWPGGV	2	4:166915629	TLL1
SQRGHPRTAL	2	1:228559450	OBSCN
FLKNTPNDASY	2	12:120591163	GCN1L1
RAFAESHNQI	2	4:88106626	KLHL8
LEKQICIPRSL	2	3:170013719	PRKCI
SSSSAGLLK	2	22:28389367	TTC28
NSMLVFLKKSK	2	1:35972480	KIAA0319L
WAWPGPCSF	2	16:88599701	ZFPM1
YAAYSGRKL	2	17:19729452	ULK2
RYGGGGRLVI, RYGGGGRLVIV	2	17:18918510	SLC5A10
RLWTIREFTL, FTLERSLMNVM, LERSLMNVM, FTLERSLMNV, REFTLERSL, FTLERSLM, REFTLERSLM, RLWTIREF, LERSLMNV, LERSLMNVMSV	2	3:44636243	ZNF660
PYPGLQCL, PYPGLQCLPL	2	12:123351799	VPS37B
TSMDSYRRV, RVIPICALYV	2	5:138643806	MATR3
RPLCGRPL	2	7:128587381	IRF5
RIHEITHIGEK	2	19:11978669	ZNF439
SPYSCQAEGPC	2	15:102264476	TARSL2
RAAAALRAV, LTRERAAAAL, TRERAAAAL, AAAALRAV	2	19:50158042	SCAF1
NEQSLPHL, NEQSLPHLI	2	3:24231684	THRB
YTSTLMLGGAM	2	9:37442272	ZBTB5

RRRVMLIV, RRWRRRVMLI, RRWRRRVM, RRWRRRVMLIV, WRRRVMLI, RWRRRVML, RWRRRVMLI, RRRVMLIVM, RRVMLIVM, WRRRVMLIVM, RRWRRRVML, WRRRVMLIV, KRRWRRRVML	2	20:3765819	CENPB
IAIKNITASY	2	18:21134924	NPC1
TEYSEDNLLFW, SEDNLLFWLA	2	6:153345514	RGS17
HLRDDIKEYK	2	9:95485059	BICD2
REAATRAEV	2	19:55815035	BRSK1
ISNEFNSQNLV	2	15:25616254	UBE3A
QPWDPKGPPKV	2	19:55869880	FAM71E2
AYLLDGLVRL	2	19:17837512	MAP1S
APVGSVMSCSL	2	17:48433967	XYLT2
NRHFKTLQI, QIDGTVTHLL, LQIDGTVTHLL	2	X:71427103	ERCC6L
CMYQACNY, APFYKRCM	2	11:1027811	MUC6
ALGGGAWEL	2	11:69063724	MYEOV
ALAPQAWWPAR	2	3:114058003	ZBTB20
KMLSVHNI	2	17:57952010	TUBD1
QRPSPRPRLL, QRPSPRPRL, RLLTMPTGL	2	7:97933623	BAIAP2L1
RVLGKDCNK, KTSFFFEARSK, KTSFFFEAR	2	5:111576501	EPB41L4A
SYRVITSKYL	2	X:39913253	BCOR
MPGAYIYA, YVTENNMPGAY, MPGAYIYAVSA, MPGAYIYAV	2	4:134072724	PCDH10
RLFNVTPR, RLFNVTPRTSR, FSLRLFNVTPR, SLRLFNVTPR	2	21:45656840	ICOSLG
AAMWNRCADL	2	12:52283257	ANKRD33
WRRENTARRGM, RRENTARRGM	2	6:33179125--6:33179214	RING1
RNLKLKLHTFK, KLHTFKSHK, KLKLHTFKSHK, KLKLHTFK	2	X:16870700	RBBP7
FHKDPNVNML	2	11:61615706	FADS2
RANPYSIVSL	2	17:21318698	KCNJ12
TRDAPGLRV, TRDAPGLRVPL	2	12:58025813	B4GALNT1
KSSSASSL, LVAASPRRMCL, LVAASPRRM	2	15:41146120--15:41146715	SPINT1
MALRPILTL	2	8:28210809	ZNF395
WLRDELANM	2	14:104124001	KLC1, APOPT1
VVDPPPSSLTM, FVVDPPPSSL	2	7:126173278	GRM8
FVKTTQLIPL	2	11:123513183	SCN3B

SPFRKHFRPGT, SPFRKHFRPG, MENISPFRKHF, NISPFRKHFR, FRKHFRPGTV, ENISPFRKHFR	2	17:7577065--17:7576881	TP53
LLPTASFL, SLLPKNPRTL, TLLPTASFL, FLGWASAQTA, FLGWASAQT, LLPKNPRTL	2	17:73501069	CASKIN2
ETATAGAPPA, ETATAGAPPAL	2	7:100285170	GIGYF1
VFSPSLQNHYL, FSPSLQNHYL, GVFSPSLQNHY	2	17:42232679	C17orf53
RQMGSSLLVSM, VSMKNPYHL, RQMGSSLL, SMKNPYHL, KRQMGSSLLV	2	10:63958149	RTKN2
AMAPETTLW, MAPETTLW	2	22:20130522--22:20130515	ZDHHC8
SLRTDCLLAVV, SLRTDCLLAV	2	5:57753012	PLK2
QMIWAFYLKMM, LQMIWAFYLKM, YLKMMYHIF, QMIWAFYLKM	2	1:35972480	KIAA0319L
AAAALLLF, AHAAAALLLF	2	2:43452439--2:43452464	ZFP36L2
ILFSSVVYF, LFSSVVYF	2	1:111216188	KCNA3
MSPPGRTQL, SMSPPGRTQL, KSMSPPGRTQL	2	12:124848228	NCOR2
MQWNNKKMAM, TMQWNNKKMAM	2	3:77657038	ROBO2
VRRKVPVRAWL, RRKVPVRAWL	2	2:43452464--2:43452439	ZFP36L2
NRKPHTPF	2	5:174156219	MSX2
TEYARVTF, TEYARVTFSTV	2	7:139056179	LUC7L2, C7orf55-LUC7L2
YRPAGRRDVTH, RRDVTHGIL, GRRDVTHGIL	2	18:908273	ADCYAP1
RTTCHSIL	2	12:40713870	LRRK2
AAFFNFVM, LAAFFNFVM	2	6:90415853	MDN1
KTAGNQISQR	2	2:152390743	NEB
QFYSPNIMAL	2	1:77627340	PIGK
ILDNYLQADTV	2	6:90578192	CASP8AP2
WQWGGAETTL	2	1:228111954	WNT9A
YQMDDVYKEPL, HIIYQMDDVY, IIYQMDDVY	2	8:38874818	ADAM9
HYQLRNVWERL, LRNVWERL, YQLRNVWERL	2	5:32126695	GOLPH3
LATADLLL, NLATADLLLAL	2	19:17000632	F2RL3
GFLATAWLTF	2	10:17659131	PTPLA
FPVDQFFLKM	2	2:39074185	DHX57
MRWGLRPL, LRPLGPGAAAL	2	1:220267581	IARS2

RQYMDKIILTI, YMDKIILTIL, YMDKIILTI	2	17:29858706	RAB11FIP4
QWVMHLCW, NQWVMHLCW	2	2:230661315	TRIP12
LRLLLLLLPL, MPPSGLRLLL	2	19:41858921	TGFB1
RELEKKILVF, LEKKILVF	2	1:37947226	ZC3H12A
QEQAARQAA, QEQAARQA	2	17:70117710--17:70117715	SOX9
VSADSGLLV, SADSGLLV, VSADSGLL	2	16:624114	PIGQ
SWAPKTSSL	2	1:156715104	HDGF
LEYINLVC	2	6:56915605	KIAA1586
AVAALQSPL, RAGNPAVAAL	2	3:139258508	RBP1
KMKKAHFLKTW, FLPSAAQKM	2	16:68835781	CDH1
FPERSRVRRM, RSRVRRML, RVRRMLAGSV, FPERSRVRRML, RSRVRRMLA	2	2:242149010	ANO7
MGNGGASLNLI	2	7:100285170	GIGYF1
TMSKMKMGRY, MSKMKMGRY	2	12:41967381	PDZRN4
YAALLTKTNQI	2	3:7620629	GRM7
TRMPGIPAGL	2	17:61781804	STRADA
KRNYLSELTM, KRNYLSEL	2	1:39759288	MACF1
MSISLVMAI, YMSISLVM	2	2:241569692	GPR35
KAAKARDREL	2	19:2226772	DOT1L
SRDGPARPYRL	2	X:153039502	PLXNB3
SRDKLPRPPL	2	1:207195389	C1orf116
DPQARAVHL	2	2:239050033	KLHL30
LLSSSWAL, ALALLSSSWAL, ALLSSSWAL	2	12:14019044	GRIN2B
IPASLNDTAL	2	10:15255561	FAM171A1
FYTLVSEGVL	2	5:140481456	PCDHB3
WLPSRQKTLL, LLLASGAPRY, WLPSRQKTL, LPSRQKTLLL, WLPSRQKTLLL	2	16:30505572	ITGAL
KYEQLALDLF, KYEQLALDL, QLALDLFSECY, YEQLALDL, REAKYEQLAL, REAKYEQLA, REAKYEQL	2	11:2435956	TRPM5
FAAARVDVF	2	2:179472135	TTN
TFCDVFWL, CFTFCDVFWLL, CFTFCDVFW, RFLCFTFCDVF, TFCDVFWLL, CFTFCDVF	2	11:111742146	ALG9
SAPCLHGASCL	2	9:126128253	CRB2
RRPIAQWHPL, ARRPIAQWHPL	2	11:1858572	SYT8

SEVLAAAGA, SEVLAAAGAVF, SEVLAAAGAV	2	10:71906150	TYSND1
FQMTGSTL, YSFLTTFQM, TTFQMTGSTL	2	6:71004213	COL9A1
EADSKEAY, ITEADSKEAY	2	14:32561296	ARHGAP5
MFFVAISF, IYMFFVAI, IYMFFVAISF, YMFFVAISFKT	2	12:63541343	AVPR1A
TFQRAPLKF	2	3:58089788	FLNB
RAVPHLSYHR	2	2:43452439--2:43452491	ZFP36L2
FFSRSHLDF, DFFSRSHLDF, FSRSHLDFSPY	2	13:109661386	MYO16
RQQGPHIPIW, CRQQGPHIPI	2	22:19839750	C22orf29
SENQIGHVLCL, SENQIGHVL	2	5:112174117	APC
APCLHGASCL	2	9:126128253	CRB2
VEECVQKFIKI	2	11:31436427	DNAJC24
SGYWGRRRAL	2	9:214706	C9orf66
WPHSSEDLLTL, WPHSSEDL, WPHSSEDLL	2	10:104176197--10:104176203	PSD
RTEHTTSSM	2	11:111742146	ALG9
FNHNTLALI, FNHNTLALIL, ALDENFNHNTL, FNHNTLAL	2	1:203037706	PPFIA4
FLARSWNT	2	3:77657038	ROBO2
ILFQEAFQI, FQIERPVL	2	1:32800539	MARCKSL1
SRSWKLIL, KYASRSWKL, KYASRSWKLIL	2	14:55429791	WDHD1
RRELRVPCTL	2	16:1250389	CACNA1H
RPVRLQKYPQA	2	12:121432118--12:121432115	HNF1A
MARPRTGK, GMARPRTGK	2	9:2824764	KIAA0020
VRGLLVGLV	2	2:43452439--2:43452244	ZFP36L2
GRNQEKLNKFM	2	8:104337567	FZD6
KLCDSGELVTI	2	3:119720928	GSK3B
RASEGFRYI	2	10:119134615	PDZD8
TTRGRMCSR	2	22:50297991	ALG12
ILSEHSRTTLF, FLLERQQM, SRTTLFLL	2	1:225211543	DNAH14
WGDAWQSLNCK, AWQSLNCKWLW, WQSLNCKWLW, AWQSLNCKW, VWGDAWQSL	2	12:58157486	CYP27B1
KEATSNQDLL, KEATSNQDL	2	3:168833257	MECOM
QRWTPAPTAM	2	3:52550119	STAB1
HPAEQCGM, HPAEQCGMF	2	21:47754665	PCNT
HWWLWWWIWGL, RHWWLWWWI, WWWIWGLLQW, HWWLWWWI	2	12:53207602	KRT4

VRATQLAF	2	10:12136141	DHTKD1
MVEDKLAL, VEDKLALF	2	2:169746017	SPC25
EEYIIQTLSRV, WEEYIIQT, WEEYIIQTLS, EEYIIQTLS, EEYIIQTL, WEEYIIQTLSR, WEEYIIQTL, EEYIIQTLSR	2	6:118596709	SLC35F1
EIRQLSSKSL	2	14:75230947	YLPM1
YIIREGAM, YIIREGAMGK	2	5:45303797	HCN1
KTDPAMEPQFK	2	16:74773946	FA2H
SQNFALTKHHW, FALTKHHWLIL, FALTKHHWL, ALTKHHWLI	2	8:32453511	NRG1
HSRVPSPR, AAHSRVPSPR	2	18:3452223	TGIF1
WTEPECGF	2	2:202344861	STRADB
NSVIIKCDGFY, VIIKCDGFY	2	1:173155919	TNFSF4
KENTLILL	2	10:100995386	HPSE2
FWSQFPFL, SYLKTMYSSLF, FLQCCHHHHL, MYSSLFWSQF, YSSLFWSQF, KTMYSSLFW, YSSLFWSQFPF, YLKTMYSSLF, KTMYSSLF	2	6:102503432	GRIK2
IPTEKLREM	2	11:61290608	SYT7
REHRREWEI, REWEIQLVN, REWEIQLV, REWEIQLVNES, REHRREWEIQL	2	7:139056179	LUC7L2, C7orf55-LUC7L2
WPDTTHPTCI	2	22:20130522	ZDHHC8
LGKQYWPI, QYWPITAF, ELGKQYWPI	2	3:30713643	TGFBR2
RRVEPQTPWV	2	6:26091204	HFE
EEAALCPA	2	17:7462459	TNFSF13
KAHAFVVQQK, HAFVVQQK	2	8:144997927	PLEC
LPEALHEAL, LPEALHEA	2	11:64083221	ESRRA
YSELSGDV	2	1:115267948	CSDE1
WWPARRGPL	2	3:114058003	ZBTB20
MGNGGASL	2	7:100285170	GIGYF1
PEWKCSPLSL, PEWKCSPL	2	11:10328153	ADM
AQRPRGHPAL, QRPRGHPAL	2	11:76751585	B3GNT6
RPPARPARPPA, RAGPRRPPA, LPRAGPRRPPA	2	16:88599701	ZFPM1
VPLRIHFCPV, CPVHRPPW, SPVPLRIHF	2	7:128852004	SMO
KLAELFTS, KLAELFTSWI	2	5:138643806	MATR3
KSYWEDYHQF	2	1:75708605	SLC44A5
HVAAVHCVQY	2	4:153247288	FBXW7
GQRCAITL, TRGQRCAITL, GQRCAITLWF	2	1:43212981	LEPRE1

WPAPTHAL, THALPPPKPL	2	19:17838385--19:17838753	MAP1S
CSSWGTLTGRM, LTGRMPLLF, GRMPLLFTCM	2	16:81904511	PLCG2
MVLNYSCSL	2	11:118925720	HYOU1
FLDNSLNTV, SLNTVLNR, FLDNSLNTVL	2	13:36909193	SPG20
YGMWGGGCWV	2	8:145689659	CYHR1
MAWAQHGSPAI, WAQHGSPAI, MAWAQHGSPA, RSSWPRWTM, RRSSWPRWTM	2	14:105174013--14:105173873	INF2
LEATALGAAT, LEATALGAA	2	16:2059622	ZNF598
AQVLLRLL	2	19:1004896	GRIN3B
YVLSGHPTQIF, FSSSQLLRQYV, YVLSGHPTQI	2	9:97062172	ZNF169
ALWCASSV	2	1:27106804	ARID1A
MAAAVADEAV	2	17:33469279	NLE1
SSRKRYCILQV, LQVSHYMM, KRYCILQVSHY, SSRKRYCIL	2	10:63958149	RTKN2
NRSSHITQHKI	2	19:22271382	ZNF257
LTSSSREWK, YPAYSKDSGLL, LLTSSSREWK, GLLTSSSREWK	2	15:45003781	B2M
YKLTYTKVWKM	2	3:77657038	ROBO2
SRKIQIGHVL	2	5:112174027--5:112174093	APC
GPHPRAHRV	2	18:3452223	TGIF1
HIQEIQTLRK	2	9:123177407	CDK5RAP2
TYDFKADIWSL	2	2:242438686	STK25
RAIQMASQTGK, IQMASQTGK, AIQMASQTGK, QMASQTGK	2	2:68622935	PLEK
IELHPLLI, IELHPLLIRII, IELHPLLIRI	2	20:35563544	SAMHD1
TTRLRTQACR, TTRLRTQACRR	2	22:18300932	MICAL3
KFFDETRRL	2	3:178916854	PIK3CA
CARWSLGRSFL	2	16:2059622	ZNF598
ARFSPGPGL	2	1:161696782	FCRLB
TSDTPKLPF	2	16:28847356--16:28847350	ATXN2L
LLYEKLLVQHL	2	2:107423243	ST6GAL2
SYSSNPHLLSY, YPSEPLEL, SYPSEPLEL, YSSNPHLLSY	2	19:54744182	LILRA6, LILRB3
ISPSTAHLY, CISPSTAHLY	2	6:135360902	HBS1L
YTLVIPIQYYV	2	7:87911999	STEAP4
YQNQHTMNK	2	1:93680314	CCDC18
RRRCPGGRVPK	2	17:3627473	GSG2
KRKPQSGLYV	2	6:84895045	KIAA1009
SAVNAGNRL, LVRSHTSM	2	19:38028415	ZNF793
SLNQCTHFLI, SLNQCTHFL	2	3:168833560	MECOM

METQFRSPL	2	12:42706970	ZCRB1
MEAQVQEL	2	16:15820795	MYH11
GQLGVVVA, MQGQLGVVV, GQLGVVVATV	2	8:145738768	RECQL4
CSDKNISIF	2	15:42110446	MAPKBP1
GRKSLLPQLL, MRLGAGRKSLL, MRLGAGRKSL, GRKSLLPQL	2	17:43482356	ARHGAP27
YFMKQMNDAY, AYHGGWTTKM	2	3:178952084	PIK3CA
KRNFKRRESL, FAMNKDKKRNF	2	18:65178966	DSEL
RPATRTAGL, CRPATRTAGLI, CRPATRTAGL	2	9:98211549	PTCH1
WRSCHHPV, HPVPLCPLI, VPLCPLICQQA, WRSCHHPVPL, HPVPLCPL	2	1:207139176	FCAMR
QQLRGLKW, LKWENGLSL	2	19:55525586	GP6
KEATLHDCL	2	18:6093469	L3MBTL4
CWGSPPRTTGF	2	19:10433397--19:10433828	RAVER1
CPAVPAAA	2	3:51417604	DOCK3
SYSQNTLIAL, YSQNTLIA, YSQNTLIAL, YSQNTLIALV	2	1:208062132	CD34
HQYSKGIFFI, HQYSKGIFFIF	2	7:47955186	PKD1L1
SPFLARSWNTM	2	3:77657038	ROBO2
FNFPFVSQGEV	2	17:10258071	MYH13
LTRPRLLPL	2	5:137803132	EGR1
KSAFFKHVAAM, AFFKHVAAM, FFKHVAAM, SAFFKHVAAM	2	8:141381188	TRAPPC9
LMMWRTPMT	2	22:18300932	MICAL3
AAFPISFI	2	1:154942910	SHC1
CAARSCARWSL	2	16:2059622	ZNF598
HPSGGGSG	2	7:1586653	TMEM184A
WELLTGEVPYH, GEVPYHGIDCL, GEVPYHGI	2	11:65375494	MAP3K11
LPRRRRRHPRA, RVGGQRPL, ELRRVGGQRPL, RPLPRRRRRH	2	8:145106939	OPLAH
FPQSLYRGTV, FPQSLYRGTVA, GSPPRFPQSLY	2	11:619789	CDHR5
SLAAAPGA	2	12:124848228	NCOR2
WRHQVLCTTCV	2	2:73302655--2:73302665	RAB11FIP5
YRRSVWFLQL, YRRSVWFL, RRSVWFLQL	2	5:141046035	ARAP3
ITIINLTVSM, ITIINLTV	2	5:138643806	MATR3

VVDIPLFFSFL	2	17:67260957	ABCA5
SRWSASGWASL	2	17:73501069	CASKIN2
FTSVPPGF, FTSVPPGFKM	2	20:30789952	PLAGL2
HLAVRTSMLTL	2	1:204228411	PLEKHA6
ASIFNNFVI, KMASIFNNF, MASIFNNFVI, KMASIFNNFV, KMASIFNNFVI, VKMASIFNNFV	2	11:66334760	CTSF
TLIDDDNPPCL	2	9:137019598	WDR5
FCSGVLSGFW, SGVLSGFW	2	8:120220776	MAL2
FWKLDTLQTF	2	3:62464048	CADPS
KAASFPCFL, FPCFLHSTL, ASFPCFLHSTL	2	2:204305618	RAPH1
GEARAAPQNG	2	16:88599703--16:88599701--16:88599696--16:88599703--16:88599696--16:88599701	ZFPM1
HPAWLSPI, ISACPRLLPY, ISACPRLL	2	17:40937417--17:40939870	WNK4
APTHQQFPF	2	2:108998302	SULT1C4
YLKIKHLLLK, KIKHLLLKR	2	5:112175594--5:112175596	APC
DEAPPLSGSW	2	9:35091693	PIGO
VYIEVVNV	2	5:140558318	PCDHB8
SRQSIWDVI, SRQSIWDV	2	2:215846946	ABCA12
ERIHIYKL, AEKERIHI, AEKERIHIYKL	2	17:263194	C17orf97
FSVMIPPM	2	2:1652960	PXDN
HLLERVNMWL	2	3:154862150	MME
CQEVFGCVERY	2	6:152470650	SYNE1
KISPITWTTY	2	14:74060514	ACOT4
TMLRNIWRA, FIWPSFMVMTM, SFMVMTML, FMVMTMLRNI, MTMLRNIWRAY	2	7:21942715	CDCA7L
FLSELGVTF, FLSELGVTFTI	2	17:59857686	BRIP1
SVRSPLLMHK, RSVRSPLLMHK, LLMHKTQASTV, SVRSPLLMH, RSPLLMHK	2	2:179659911	TTN
SPAPSPLATPL	2	1:54605318--1:54605319	CDCP2
FRYLLAVLIL, FRYLLAVLILF, FRYLLAVLI, FRYLLAVL	2	5:74324983	GCNT4
FAICFVCGI, FAICFVCGIF, FAICFVCGIFF	2	6:166743666	SFT2D1
FEPSIRGSVL, FEPSIRGSVLL	2	17:61560495--17:61560426	ACE
SQFGGVLHL, FSQFGGVL, RRFSQFGGVL, FSQFGGVLHL	2	6:24437388	GPLD1

PTAPHMEY, IPTAPHMEYI, MIPTAPHMEY, RVMIPTAPHM	2	11:75298129	MAP6
HPWGPSCLL, LPHQLHPW, HPWGPSCLLCL, HPWGPSCL	2	1:154942911	SHC1
ALAGPVLTTL	2	9:98209593--9:98209617	PTCH1
MGSQSGAWTM, TRMGSQSGAW	2	7:100285170	GIGYF1
LYGHTSTVHCM	2	4:153249384	FBXW7
SASCSSSFSL, SSSFSLVM, CSSSFSLVM, SFSLVMTL, SASCSSSFSLV, SFSLVMTLTL, KSTSASCSSSF, SSFSLVMTLTL, TSASCSSSF, SSFSLVMTL, SASCSSSF, STSASCSSSF, FSLVMTLTL	2	11:970195	AP2A2
LEAWRVSPRPL	2	13:111368164	ING1
EIYFLGPK	2	17:42818751	DBF4B
FMHSAHLAL	2	20:62421840	ZBTB46
SLLSRMPAR	2	18:34205516	FHOD3
KLEEGWGV	2	1:28920547	RAB42
RRFELRRV, RRVGGQRPL	2	8:145106939	OPLAH
AQAQRAWWVL	2	20:61870727	BIRC7
TRDERTHKI	2	6:159636168	FNDC1
TWMRPPRSLL, TWMRPPRSL, WMRPPRSL	2	22:18300932	MICAL3
EEDPLWPSTAM	2	2:136566201	LCT
STAKRLEAAFR, SLSASTAKR	2	8:65493429	BHLHE22
SVAPATLPCR, SVAPATLPCRR	2	22:20130522	ZDHHC8
ITIQGSIAF, SANANVTI, SANANVTITI	2	10:7608128	ITIH5
AVRGCSSRGPV	2	1:161696782	FCRLB
MDFTDIIKLF	2	22:25315953	SGSM1
VQDCSFVV	2	3:33255459	SUSD5
QRSVLTKHQII	2	10:38120580	ZNF248
YYWDASACVI, YYWDASACV, LYYWDASACVI, YYWDASACVIM	2	1:205740743	RAB7L1
SRDVVVVKVTM	2	5:52214576	ITGA1
PMFLYLYY	2	8:120220776	MAL2
SRHHSRDV	2	10:18828493	CACNB2
SFLSEAHF	2	3:175345104	NAALADL2
FTKDNQVHI	2	5:64893000	TRIM23
QEEEAAAAAAA, EEAAAAAAAYL	2	1:11710779	FBXO2
EEWGGEAH	2	14:24040436	JPH4

VPREKRAQW, VPREKRAQWV, QWVSVATL	2	9:137677855--9:137688741	COL5A1
LLHSWISL, CLLHSWISL	2	1:35370639--1:35370731	DLGAP3
KTSNHLIF	2	10:25861759	GPR158
GVFEYHNIWVY, WVYDEEATDLL	2	17:27977745	SSH2
YADAGILY	2	1:197073231	ASPM
RLVSTLNFSA	2	10:94243045	IDE
YQNEEEEEAIY	2	4:27019558	STIM2
STPAGFHPPV	2	12:123351891	VPS37B
KTKDVPFFK	2	6:151673370	AKAP12
IRAKTASI	2	14:27066542	NOVA1
FFRFRGRL, VRFFRFRGRL	2	19:54974318	LENG9
FATWSALV, FFFATWSALV, FFFATWSAL, FFATWSALVRL, FFATWSAL, FFATWSALV, FATWSALVRLF	2	14:55818554	FBXO34
VRHHPPCWSPL	2	5:14487781	TRIO
HVVDITHTSPA	2	2:74590132	DCTN1
ARLARTAI	2	2:43452623	ZFP36L2
VTYHHHLY	2	19:33202775	NUDT19
ATEHSSLSLH	2	17:72350412	KIF19
KQLKPSEKYL	2	5:112175594--5:112175596	APC
FQISTYQA, LLAVVIQFQI, STYQAQLRI, LAVVIQFQI, STYQAQLRISL, CLLAVVIQFQI, FQISTYQAQL	2	5:57753012	PLK2
KITTANVVYY	2	14:30100143	PRKD1
ALMRSHWPSA	2	11:64457919	NRXN2
YQAQLRISL	2	5:57753012	PLK2
FATANRGNGL	2	1:246670481	SMYD3
LDFDVTDNQI, NQIYWTDI	2	12:12317235	LRP6
VRGCSSRGPVL	2	1:161696782	FCRLB
MLDMAECEGIV	2	1:29641954	PTPRU
MVRWAAASKL, MVRWAAASK	2	19:52715982	PPP2R1A
FLNPRKATQSF	2	6:80383444	SH3BGRL2
MEHRGPPQNI	2	X:112065639	AMOT
YMQPPPRHYL, MQPPPRHYL, YMQPPPRHY, LYMQPPPRHYL, HYLCSSSM	2	11:6590002	DNHD1
HSHKRNCQLK	2	15:51860704	DMXL2
MLHSCRIQK, EQMLHSCRI	2	X:56591874	UBQLN2
ALSPEPPAYSL	2	X:3631167	PRKX
RQNRFASSK	2	8:109796543	TMEM74
VEGQIHKLCL	2	13:31232879	USPL1

VIDKMNIKL	2	15:62233987	VPS13C
KIIQNEGIK, IIQNEGIK	2	7:87479241	SLC25A40
RRLGHNWSI, LRRLGHNWSI	2	1:17266586	CROCC
LLMQEITAM	2	6:152644655	SYNE1
TTMECVPV	2	20:57767560	ZNF831
ILNGISAL, YILNGISALR, YILNGISAL, IRYILNGI, KQIRYILNGI	2	7:22767227	IL6
IIDNLHHV	2	12:78591108	NAV3
RAVARMGQGMM, RAVARMGQGM	2	17:79803764	P4HB
LRREKSNL	2	1:17266586	CROCC
AASSRAQVLAL	2	6:5260936	LYRM4
SLKSHLLKY	2	2:179529600--2:179528765	TTN
LPYPFHVL, YPFHVLALEV, ISACPRLL, LPYPFHVLAL, ISACPRLLPY	2	17:40939870--17:40937417	WNK4
FSINSDIWSY	2	9:94486762	ROR2
FVVCTTGWL	2	3:33255459	SUSD5
MAISLTAL, MMTMAISLTAL, FTCMMTMAISL, MMTMAISLTA, MTMAISLTA, MTMAISLTAL, CMMTMAISLTA, FTCMMTMAI, MMTMAISL, CMMTMAISL, MAISLTALCAA	2	20:31384651	DNMT3B
VASDVTKQL	2	2:179454575	TTN
HRLALTGEKRA, GRIHRLALT	2	19:6737481	GPR108
YAHPSSAF, KYAHPSSAF, YAHPSSAFAPK	2	5:173317785	CPEB4
SPLEEWNQWVM	2	2:230661315	TRIP12
VMFPDTSTL, LVMFPDTSTL	2	6:51890015--6:51889974	PKHD1
AQCPSVPL	2	8:145625525	CPSF1
SEVSRISKVL	2	12:106634007	CKAP4
AADVAQLTH	2	1:66829220	PDE4B
AREASLLL, REASLLLPSW, REASLLLPSWV, EPAAREASL, REASLLLP, REASLLLPS	2	16:2070164	NPW
FQDLQNDAPV, LQNDAPVPY	2	19:48879376	SYNGR4
MYQVQSPM, MYQVQSPML	2	1:147092566	BCL9
YKFSNTPKNVL	2	19:12089902	ZNF763
APSTEWWTMV	2	6:116836954	FAM26E
LPAPHCCQW	2	1:204438071	PIK3C2B
FPGSELREAH	2	19:1065018	ABCA7
KIMCQLLFRQF, CQLLFRQF, IMCQLLFRQF	2	X:41555920	GPR34

LMVWWVHQV, MVWWVHQV, FSYLMVWWV, YLMVWWVHQV, AVFSYLMVWWV	2	9:19528054	SLC24A2
LLPPSAPHHVL	2	10:100186986--10:100186987	HPS1
APGERRRLGF, APGERRRLGFL	2	10:17659131	PTPLA
ARPWSSAPTRM	2	19:5231449	PTPRS
MMGQNGNLYFA, MMGQNGNLYF	2	X:153136382	L1CAM
HPSPRSTWTW	2	19:17838753--19:17838385	MAP1S
FDFKLQDV, FDFKLQDVSSV, FSHKFDFKL	2	2:32475708	NLRC4
TRPSRAREAL	2	9:98211549	PTCH1
EWKVKFPEL	2	15:45003781	B2M
LLGDPFHPL, GLLGDPFHPL	2	19:10116508	COL5A3
HLLLHTEV, VNHTHLLL	2	7:99145140--7:99145142	FAM200A
YGHTSTVHCM	2	4:153249384	FBXW7
TRAGTRGSLV, TRAGTRGSL	2	16:2059622	ZNF598
TASPHSSSF, CTASPHSSSF, CTASPHSSSFL, FLPVPTGRSL, ASPHSSSFLPV, HSSSFLPV, TASPHSSSFL	2	17:79803764	P4HB
SRNYNRRYQI, NYNRRYQISI, YQISICPL, SRNYNRRY, NRRYQISICPL, NYNRRYQI, RRYQISICPL	2	10:121341501--10:121341690	TIAL1
YLVDHKVHF	2	2:112922646	FBLN7
CDLQSSLY	2	6:32811743	PSMB8
VEEASVPA, LEVEEASVPAA, LEVEEASV, EEASVPAA, VEEASVPAA, LEVEEASVPA, EEASVPAAEPA	2	2:239039057	ESPNL
SVPGVSPV, DHSVPGVSPV, HSVPGVSPV	2	19:17838385--19:17838753	MAP1S
GSVEVIQTL	2	19:51217155	SHANK1
QAAVDHTTEL, AAVDHTTEL	2	9:95480987	BICD2
IQIGILIGI, WHIQIGILI	2	12:66622063	IRAK3
EESLPGAM	2	5:137803132	EGR1
VYYEHLEKAAM, YEHLEKAAM, YEHLEKAAMH	2	12:101705568	UTP20
FVSDAVATY, FVSDAVATYY, FVSDAVATYYL, TYFVSDAVATY	2	12:133249847	POLE
FALRPSTV, FALRPSTVPF, STVPFLTRAV	2	15:75942664--15:75942736	SNX33
DRSPQAPLRL	2	X:129190011	BCORL1

HMPRTPALCY, ARPWFPRHM, RHMPRTPAL	2	19:50926264--19:50926165	SPIB
STPPGPGPAV	2	20:21493012	NKX2-2
WSPTHSAQSPV, HSAQSPVPL, AQSPVPLRIHF	2	7:128852004	SMO
INDPRKNPTLW	2	8:28989877	KIF13B
LEIKPYST, LEIKPYSTKKM	2	16:15131990	NTAN1
ATCGCPSSRR, ATCGCPSSR	2	17:70119913--17:70119870	SOX9
FAFNDQFIQIL	2	3:164737505	SI
APAFHTGGPLA, APAFHTGGPL	2	22:28196403	MN1
FSLQREELRAV	2	19:55598724	EPS8L1
KLALCETPAPL	2	21:35183447	ITSN1
FLTDREVQL, LTDREVQL	2	2:11362292	ROCK2
RVVGEVFEVPR, VRSRVVGEVF	2	20:49197838	PTPN1
RAAAGVSV, FAATRAAAGV	2	20:31384651	DNMT3B
LVRQQRLRL	2	2:43452491--2:43452464	ZFP36L2
SRWWSAPSRWL	2	22:50900047--22:50899955	SBF1
QRLLYVYI, YIIETNVF, LYLDKFVQRLL, LYLDKFVQRL	2	18:12699829	CEP76
GRRLGCRV, GRRLGCRVQAL, GREARGRRL, REARGRRL	2	9:140100317	NDOR1
FQILFTMTLI, FRLFIQNLFF, FIQNLFFQI, RLFIQNLFFQI, FQILFTMTL, ILFTMTLI	2	3:98506942	ST3GAL6
WRTAPLLASL	2	1:27100176--1:27100350	ARID1A
LYILPRKVLQM, LYILPRKVL	2	5:112175756	APC
FFHAMHVV, MVFFHAMHV, MVFFHAMHVV	2	4:4285458	LYAR
YPMSSSGL	2	19:39663913	PAK4
YVSATTGAI	2	5:174938494	SFXN1
GPAGRIGGNF, GPRLGPAGRI	2	18:3452223	TGIF1
AWVPAPAGWDL, AWVPAPAGW	2	17:39780697	KRT17
FILGRNRTDVM	2	19:52888438	ZNF880
GTPPWMPFR	2	10:100186987	HPS1
MASGPASSW	2	3:52550119--3:52551362	STAB1
GETGRPTLNQM	2	17:48619249	EPN3
MQHVWRKVDTV, MQHVWRKV	2	3:9426224	THUMPD3
DRSPQAPL	2	X:129190011	BCORL1
ASRVWPSTTTL, SRVWPSTTTL	2	6:13206135	PHACTR1
REAFLEQMMGL, LEQMMGLEGAV	2	9:133948049	LAMC3
AEFRCPGSL, AESPGAEF	2	17:7462459	TNFSF13

MRCSWTSSSL	2	19:16314384	AP1M1
NMQTIWVTGRK, VEERRNMQTI, QTIWVTGRK, RRNMQTIWV, MQTIWVTGRK	2	3:121209199	POLQ
LSYHKGLAWAR, KIGLSYHK, HLAKIGLSYHK	2	6:90387403	MDN1
TELGFTLLKL, TELGFTLLKLL, TELGFTLL	2	8:67752239	SGK3, C8orf44-SGK3
NFIDSKEVYR, KVYQNFIDSK, FIDSKEVYR	2	10:50684305	ERCC6
FWPQRCAQV, FWPQRCAQVL	2	6:46656871	TDRD6
RRWRCSQTTSL	2	5:131042147	FNIP1
KLYCLIKI, IQKLYCLIKI	2	10:128908585	DOCK1
ALQDLAPPAPL, ALQDLAPPA	2	18:72998443	TSHZ1
WPARRGPL	2	3:114058003	ZBTB20
RRTSKWMEML, MRRTSKWMEML, RRTSKWMEM, MRRTSKWM, MRRTSKWMEM	2	17:5036224	USP6
GRRVCLFL	2	3:65346916	MAGI1
SPFRSVGVSAS, MENISPFRSV	2	17:7577065--17:7576881	TP53
LYQESLPTLF, YQESLPTL, LYQESLPTL	2	6:10557410	GCNT2
MQDPLLSL	2	3:168833257	MECOM
NRKIINQIL, NRKIINQILIL, NRKIINQILI	2	16:46836877	C16orf87
KSSNFRRYK, SSNFRRYK, LLKSSNFRRYK	2	21:38302646	HLCS
KYLMKPLI, TWPLKYLM	2	12:52379121	ACVR1B
FTLPFNMVL, FTLPFNMV	2	18:43316498	SLC14A1
MEQGHLQM	2	8:125109516	FER1L6
HLVGIALPEVM	2	1:46120890	GPBP1L1
LEQLLSQGA	2	19:17393530	ANKLE1
FTAQYYRLQF, AQYYRLQF, FTAQYYRL	2	17:29120666	CRLF3
VAPQPQLL	2	2:43452464--2:43452439	ZFP36L2
KENVENRAEM, AEMALLDSL, VENRAEMAL, AEMALLDS, AEMALLDSLE, VENRAEMALL, AEMALLDSLEP	2	3:48612871	COL7A1
VRAAWAASAV, QQVTGIVWAV	2	17:73501069	CASKIN2
KNISLVIGMFI, ISLVIGMFI, VKNISLVI	2	5:5486894	KIAA0947
FGAGETGRPTL	2	17:48619249	EPN3
MFLKKTHLQL	2	16:15131990	NTAN1

RMYGWNFRV, KRMYGWNFRV	2	14:56137474	KTN1
APRSTSRPT, RPTGRPAT, RPTGRPATVA	2	16:2059622	ZNF598
AEIWFLGSS, AEIWFLGSSAS, AEIWFLGSSA, AEIWFLGS	2	9:139945517	ENTPD2
SQEMFSQALL	2	15:91304139	BLM
RIYITNYYY, ITNYYYGNTLV, RIYITNYY, YITNYYYGNTL	2	1:161954657	OLFML2B
LRLRRMHV, LRRMHVCGGSL	2	16:1273490	TPSG1
STWHSVLM, RSTWHSVLM, RSTWHSVL	2	16:50745399	NOD2
SLKNLSWK, QSLKNLSWK, QSLKNLSWKK, RVKKLKRQSLK, SLKNLSWKK	2	7:26245989--7:26245999	CBX3
VFKFCAPRLW, KFCAPRLW	2	1:201983066	ELF3
LENRDPLDSRV, WMSQVLDSL, RVLRGSRAAM, CLWMSQVLDSL	2	3:48612871	COL7A1
KALTTRRSSL, LRSPSPKAL	2	11:618971--11:618999	CDHR5
HRLSLSVPDLL	2	15:94841631	MCTP2
MRATGPPPPHY	2	19:39330958	HNRNPL
FLSPWPSPAI, WPSPAIPL, WPSPAIPLL, SPWPSPAIPL, FLSPWPSPA	2	6:33263965	RGL2
MTLPAPTACSR	2	22:20130522	ZDHHC8
LVIDTMTEV	2	13:46288409	SPERT
EMEDQKHSY, LLEMEDQKHSY, MEDQKHSY	2	1:209948748	TRAF3IP3
IYWTGRKI, IYWTGRKIDPV	2	4:123283194	KIAA1109
SSPPPLLL	2	8:144886852	SCRIB
QPLLPAHHPL, QPLQPPTLQPL, QPPTLQPL, LPAHHPLTV, LPAHHPLTVRL	2	17:70120194--17:70119909	SOX9
CRKSDASSL, CRKSDASSLM	2	11:9172291	DENND5A
YGDIIQQDF, YGDIIQQDFL	2	3:160804109	B3GALNT1
RTMEDAAHM	2	3:52551362--3:52550119	STAB1
TMTVWMAV, GTMTVWMAV	2	12:57595335	LRP1
ALMVVPALL, TAGAPPAL, TATAGAPPAL, ALMVVPALLA, TATAGAPPALM	2	7:100285170	GIGYF1
WVADSNVFTK	2	1:220311332	IARS2
TSWPSALAY, RTSWPSALAYL, RTSWPSAL, RTSWPSALAY, TSWPSALAYL	2	5:140176838	PCDHA2

QMLAVSATYPK	2	1:112303389	DDX20
VASISAVSLEV	2	1:22191454	HSPG2
QEKLDQYSVI	2	6:152679555	SYNE1
RVPSVTLV	2	18:44400988	PIAS2
LSCVVFINK, LSCVVFINKK, PLSCVVFINK, CVVFINKK	2	9:95610657	ZNF484
CFPGNQHPL, FPGNQHPL	2	11:124845049	CCDC15
HQFWWDISK, CRILWGHQF	2	9:134073007	NUP214
TRCSWQARPL	2	4:860923--4:861066	GAK
LPPSPSTSCSM, LPLPPSPST, SPSTSCSM	2	16:2059622	ZNF598
MERCTHCT	2	16:2059622	ZNF598
EASKKAMGPL	2	7:100285170	GIGYF1
IENSSIQELF, IENSSIQEL	2	2:210558484	MAP2
FSKRCDSPLPV, FSKRCDSPL	2	11:4674564	OR51E1
CTWVRGPTAPR	2	X:1407724	CSF2RA
SLHFLCWSL, FLPGSTPSLHF	2	16:84213651	TAF1C
KLDFETMSLY	2	5:140626087	PCDHB15
KLLGQLYK	2	11:33054956	DEPDC7
RSWMWNQLF, SWMWNQLF, NQLFLLEEY, RSWMWNQLFLL, RSWMWNQL, WMWNQLFL, RSWMWNQLFL	2	5:31267760	CDH6
YHHPCQAPV	2	14:105173873--14:105174013	INF2
AEWPGLALAE	2	16:2059622	ZNF598
KENLIGTLL, KENLIGTL, KENLIGTLLAI	2	1:24766677	NIPAL3
SAPHHVLPAPV	2	10:100186986	HPS1
MVPWGMEAL	2	14:104642555--14:104642401	KIF26A
WMVPWGMEAL	2	14:104642401	KIF26A
IRPPQSPPPEL	2	16:677579	RAB40C
DLEFHGVMIFY	2	6:168265256	MLLT4
LPLPRRPRGPW	2	10:124895627	HMX3
HALQILLLV, RVLRATHAL	2	10:112838962--10:112837855	ADRA2A
LLSFWPGGYPA	2	15:45003781	B2M
HRPLCQRF, RFPTLTVSRWM	2	16:14346300	MKL2
GQWMAHMAL	2	1:27100176--1:27100350	ARID1A
ARLFALLLL	2	20:43976991	SDC4
ITFLFDSTL	2	8:124799557	FAM91A1
SLPPPPPSQL	2	7:101839973	CUX1
STFEGAKML	2	6:87967866	ZNF292
LLLSPLDSSPL	2	3:77657038	ROBO2
KMDLDGMLTV, GMLTVIGQGLV	2	17:76046980	TNRC6C
LLLWVENL, MLLLWVENL	2	14:103593950	TNFAIP2

NPHIQRLL, SEYLLGNPHI, NPHIQRLLNTT, YLLGNPHIQRL	2	4:8607830	CPZ
HPQWAPHSAGL, MPPLLHPQW, HPQWAPHSA	2	17:48433967	XYLT2
RQQPGLSA	2	1:204159787	KISS1
HPSPFSGL	2	2:204305618	RAPH1
KLPSCPLATW	2	17:61560426--17:61560412	ACE
FYISSTVCF, FYISSTVCFL	2	3:182603763--3:182602674	ATP11B
YRCEGDTTPTI	2	7:81346553	HGF
NISSRTRVL, SRTRVLMV	2	5:140801569	PCDHGA11
HTSTVCCMHLH	2	4:153249385	FBXW7
VQDRAVPHL	2	2:43452464--2:43452439	ZFP36L2
AESPGAAAL	2	17:7462459	TNFSF13
FRITRGPRAA, RRSAVSCF	2	7:42005573	GLI3
WATSGAATPV, WATSGAATPVI	2	9:16436658	BNC2
TLLPSTTSSL	2	9:5921755	KIAA2026
STMAPGSSAAV, TMAPGSSAAV, LASPSLSTSTM	2	11:618999--11:618971	CDHR5
SRHGTQPL	2	5:141046035	ARAP3
NSCQTTTY, FQNSCQTTTY	2	1:82408841	LPHN2
STFNNVMEQNF	2	8:12957475	DLC1
LATPPPLL	2	2:60688969	BCL11A
SAQQSLTK	2	3:114058003	ZBTB20
YVAEAGPPPA	2	14:74967607	LTBP2
SLMEQIPHLK, SLMEQIPHL	2	13:53049034	CKAP2
SEQADKKVSL	2	8:70667685	SLCO5A1
FTNKENNL	2	9:86617158	RMI1
HYPCPPAL	2	11:64507638	RASGRP2
SEMAVHRRTHS, SEMAVHRRT, SEMAVHRR, SEMAVHRRTH	2	16:30566747	ZNF764
ARMAQAYVL, RMAQAYVL	2	18:21453109--18:21451595	LAMA3
ALNKHSLERY	2	16:12136879	SNX29
KMNFTGGTL, KMNFTGGTLAI	2	6:160485488	IGF2R
REIDVLEW, LEWELNILIF, WELNILIFQL, REIDVLEWEL, WELNILIF, LEWELNIL, LEWELNILI	2	1:233497916	KIAA1804
ELSRCPEPY, LSRCPEPY	2	11:118770651	BCL9L
QSPHHAGHLR	2	16:68026995	DPEP2
LQWPHPDPA	2	14:104642401--14:104642560	KIF26A
LARMGGAASF	2	7:91732038	AKAP9
CEGFKLHF	2	7:115874657	TES
FEMVTAGDMRV, FEFEMVTA	2	1:237729972	RYR2
NPSSWLVSL, FVVQNPSSWLV	2	4:154513664	KIAA0922

STVASSHRFCF, STVASSHRF	2	7:91732038	AKAP9
HPDQTFSSVA, QTFSSVAR, HPDQTFSSV, QTFSSVARSWR	2	13:36167523	NBEA
SRKEELMFFFV, KEELMFFF, SRKEELMFFF, KEELMFFFV, EELMFFFV	2	20:17581679	DSTN
PYPAASAPCW	2	22:20130522--22:20130515	ZDHHC8
HEAVEKEA	2	9:140918171--9:140918181	CACNA1B
STSSHLILL	2	22:31485922	SMTN
FIRPANQYK	2	6:76623878	MYO6
VRMCNHPPRKI	2	X:47083885	CDK16
HLILLWLL	2	22:31485922	SMTN
WKKASWVSSTL, KKASWVSSTL	2	5:13701536	DNAH5
SQGLFLHPHCK, GLFLHPHCK	2	10:85978958	CDHR1
MRRQWSLWVL	2	6:116836954	FAM26E
KLRCLQSLGF	2	9:135947054--9:135947065	CEL
LRHKTSGKICL, KTSGKICLLM	2	2:109380686	RANBP2
FAAELPPETY, FAAELPPETYL	2	6:128388794	PTPRK
HLQPHDAL, RHLQPHDAL	2	7:42005678	GLI3
VAAAHAVQRPV, RAVAAAHAV, SLFCLQRAV, VQLGQHPPV	2	20:21493012	NKX2-2
CPRTTWSA, LPCPRTTWSA	2	10:124895627	HMX3
LAFNVPGGVW	2	14:20846338	TEP1
LEAFPLPRASL	2	19:1430254	DAZAP1
WWASSTLM, STPMWWASSTL, MWWASSTLM, TPMWWASSTLM, TPMWWASSTL	2	11:46702224--11:46702223	ARHGAP1
VLLSRELFR, QLWDVLLSR	2	1:15850613	CASP9
TRSLTLTPSL	2	21:42551433	PLAC4
WMTDVIHML	2	6:152688429	SYNE1
FWDGKIML, FWDGKIMLV, FWDGKIMLVL	2	8:27649528	ESCO2
TAVTGSVSGMF, TAVTGSVSGM	2	12:27132834	TM7SF3
CLFFHWLK	2	1:171086513	FMO3
KLFSELEFGK	2	10:97135757	SORBS1
KAPSLSQLFLF, KAPSLSQLF	2	2:234420362	USP40
FLAQHQII	2	19:56935058	ZNF583
WLFREHCVCV, WLFREHCV	2	15:44811371	CTDSPL2
QEVCMLFKV, QEVCMLFKVSM	2	5:148427439	SH3TC2

RRPRCWVL, RRRPRCWVL, MRRRPRCWVL	2	2:220494118	SLC4A3
CICLPAKR	2	7:20371449	ITGB8
MQMGGGGAMNM, YMDPRERDMQM, MQMGGGGAM	2	1:35652836	SFPQ
YQHLPIRIFV, YQHLPIRIF, AYQHLPIRIF, SETLAYQHLPI, LPIRIFVALF, LAYQHLPIRIF, TLAYQHLPIR, IRIFVALF	2	17:56385076	BZRAP1
RVAASAPL	2	11:76751585--11:76751604	B3GNT6
DEGYGPPPL	2	19:39330959	HNRNPL
QPPPTTSFPFL, FPFLSGLPG	2	16:30736370	SRCAP
AMDSLRPVRL	2	12:121432115	HNF1A
KLVPLVILGLI, LIADSSASV, KLVPLVIL, KLVPLVILGL, GLIADSSASV	2	1:43772910	TIE1
KLITQIDAAI, KLITQIDAA, KLITQIDAAIL	2	14:45642287	FANCM
ASMERPVRCV, TRASMERPV	2	3:52550119	STAB1
FSSKNPETI, YIFSSKNPETI	2	7:121650934	PTPRZ1
LQDQVHFV	2	1:87549881	HS2ST1
YASSIRIHEI, NYASSIRIHEI	2	19:12637432	ZNF564
WPPPPQGAQV	2	19:1004710	GRIN3B
AGFAFSIFK, VAGFAFSIFK, SIFKSDIPF, SVAGFAFSIFK, GFAFSIFK, KSDIPFIF, FSIFKSDIPF, KSDIPFIFTFL, KSDIPFIFTF	2	17:66881414	ABCA8
KEDIMCMY	2	16:2011238	NDUFB10
TPYQSQGFST	2	4:160277271	RAPGEF2
ARLAALGRQV	2	19:7747293	TRAPPC5
KRKKKMCKV	2	10:29760116	SVIL
KMKTSTSL	2	5:57753012	PLK2
RLLPYPFHVLA, RLLPYPFHV, LLPYPFHV	2	17:40937417--17:40939870	WNK4
FVNVYITSFI	2	20:5170471	CDS2
WELASHCTA, WELASHCTAA, WELASHCT	2	2:148680663	ACVR2A
MVRTASETSL	2	7:4153028	SDK1
IRVVSHTSIPL, IRVVSHTSI	2	1:76877876	ST6GALNAC3
YSIKNQTIL, HTAATEGLPNY, YSIKNQTI	2	12:85521717	LRRIQ1
YSRWIFLF	2	5:112175760	APC
VQFMKITM	2	8:113241029	CSMD3
IAAAPTLTPA	2	19:10202224	C19orf66

LRLVCLQSM	2	12:122723193	VPS33A
WPLLRGQSEI, WPLLRGQSEIV, LTAPGVCWPL, LTAPGVCWPLL	2	19:42861727--19:42861055	MEGF8
DPTRVMVPL, DPTRVMVPLPV, VPLPVWMSLL, LPVWMSLLERV, LPVWMSLL, VPLPVWMSL	2	3:3067806	CNTN4
FAPFFVPMSL, SLACSITV	2	4:15067858	CPEB2
RTALGGSQPL, LSATPSRVL, LSATPSRV	2	1:228559450	OBSCN
SFQSHMIM	2	19:12461221	ZNF442
KLLIQILPSL, LLIQILPSL	2	18:21662977	TTC39C
WTPEPDTPL	2	20:18163864	CSRP2BP
TTRLHTAHSTL	2	17:70120345--17:70120314	SOX9
EEKKCSPSCPM	2	10:61828580	ANK3
NEDRRQGVVVL, RRQGVVVL, DRRQGVVVL, RRQGVVVLHVL, FTNEDRRQGVV, RRQGVVVLHV, FTNEDRRQGV	2	20:19496186	SLC24A3
RSLHHHHLPL, SLHHHHLPLPV, SLHHHHLPL	2	11:67812475	TCIRG1
CSWFNGTEL	2	7:1097183	GPR146
TEYKLVVVGAA	2	12:25398284	KRAS
LRPKGFDTL	2	12:132475981	EP400
MIVPSVGHHLW, HLWSSLSF, TYQKFLCRNM	2	12:9885707	CLECL1
RQFPNTSL, KRQFPNTSL, RQFPNTSLI, RQFPNTSLIGL	2	12:21630862	RECQL
HRLGAGSAAV, HRLGAGSAA	2	19:18879553	CRTC1
YWDRVSRTVSL, YYWDRVSRTV, YWDRVSRTV	2	1:200956173	KIF21B
LSFFYSSVL, SLSFFYSSV	2	11:124972065--11:124972035	TMEM218
AWHSHPLTCSL	2	2:219920368--2:219920405	IHH
SRPSSGSFL, FLSPCSPL	2	11:46702223--11:46702224	ARHGAP1
IREKYIEIDAL	2	14:92470812	TRIP11
RLFELSELR	2	19:19040312	HOMER3
KVFQSSSK	2	19:52918941	ZNF528
LPQKKTPSEM, MAVGGLSPL	2	1:207139176	FCAMR
FNNETFRISV, YIRERFNNETF, ERFNNETFRI, FNNETFRISVY, ERFNNETF	2	20:55803340	BMP7
EELQVDQLWDV	2	1:15850613	CASP9
ITSQINLI	2	1:200573037	KIF14

GPSWMEAWNQM, SWMEAWNQM	2	17:7496122	FXR2
SFSGFCPATL, FSGFCPATLTV, FSGFCPATL	2	19:11353972	DOCK6
SLMTNCLF, MTNCLFSPIF	2	2:54093345	PSME4
TTSTTAKK, SSMTTSTTAK, MTTSTTAK, SSSMTTSTTAK, MTTSTTAKK, SSMTTSTTAKK	2	8:22054260--8:22054259	BMP1
TYNKTDVATPF	2	3:125824614	ALDH1L1
LYLYYKDGSCL	2	8:120220776	MAL2
VLWPLRPPPL	2	5:137803132	EGR1
FATLALVI, FATLALVITL	2	14:74060514	ACOT4
AMAATTTTTI	2	1:176525981	PAPPA2
RLQLRLNPR	2	19:17836781	MAP1S
STTSTPCSSV, TTSTPCSSV	2	16:30041821	FAM57B
HLHHHYHHVL, FLQCCHHHHL	2	6:102503432	GRIK2
VERNIQII	2	4:71527815	IGJ
KTQIGLAQY	2	8:121209180	COL14A1
SLTARVIAL	2	3:33414638	FBXL2
TREAKYEQL, TREAKYEQLAL	2	11:2435956	TRPM5
SRSAPPTRGPL	2	X:135956575	RBMX
RPSCAGPSRRA, TPRATRPSCAG, TPRATRPSCA, TPRATRPS, RPSCAGPS, TPRATRPSC, GPSRRAARAST	2	2:43452623--2:43452512	ZFP36L2
SRMRPSWL	2	19:50926165--19:50926264	SPIB
HRHYASPEI	2	9:131370209	SPTAN1
ARGIAIISVL	2	12:4919742	KCNA6
RMDFDEFFITL, RMDFDEFFI, DEFFITLSSM, FFITLSSM	2	12:132394804	ULK1
LLFGLVLLLLR	2	2:43452439--2:43452491	ZFP36L2
LDFETMSL, LDFETMSLYDL, FETMSLYDL, FETMSLYDLDI	2	5:140626087	PCDHB15
SQIYAVDYETL	2	16:1823024	MRPS34
HSSLSLHSL	2	17:72350412	KIF19
NYRVYNMM	2	22:37273855	NCF4
EELLNTLYCEF, KEELLNTLY, EELLNTLY	2	17:27014352	SUPT6H
ARFDGRPSSHL	2	1:158064555	KIRREL
MGLALALALAL, MGLALALAL	2	11:6411931--11:6411935	SMPD1
YVPVAPPHPSL	2	5:154173389	LARP1
TAAHVTDM, TAAHVTDMVEV, ITAAHVTDM	2	X:135956575	RBMX
ASPSYWAAV	2	7:150034378	LRRC61
SRRETHLHTY, RRETHLHTY	2	10:114900984	TCF7L2
SPREEAGQL	2	22:50528569	MOV10L1

KYTDSNNIASL	2	11:17419957	ABCC8
SWLYAAATV, LYAAATVCL, YAAATVCL, SWLYAAATVCL, LYAAATVCLL, YAAATVCLL	2	5:131647886--5:131657935	SLC22A4
KIHILQKHGK, ILQKHGKNVPK, HILQKHGK	2	20:56089711	CTCFL
RAIGLSWVL	2	17:7402719	POLR2A
CEICVKSHH	2	18:34205516	FHOD3
SPFRTRPA, RPAFKKKI, SPFRTRPAF, NISPFRTR, RPAFKKKIV	2	17:7576881--17:7577065	TP53
SANTAPALPMV, MVVVTATRTTL, NTAPALPM, SANTAPALPM, SANTAPAL, VVVTATRTTL	2	15:41146120--15:41146715	SPINT1
LLPEHIVVCSL	2	22:44583707	PARVG
PPRRGRRTSL, DPPRRGRRTSL	2	2:43452491--2:43452464	ZFP36L2
QPCAHGLWL, RVWPSRRQPC, WPSRRQPCAH	2	19:39330959--19:39330958	HNRNPL
AYVVYPDERTTL, VYPDERTTL, VYPDERTTLY, YPDERTTL, YVYPDERTTL, YVYPDERTTLY	2	4:140450287	SETD7
MTNYSIQFL, MTNYSIQF, KTPMTNYSI	2	12:65134422	GNS
LADGATVRL, GPLADGATV, LADGATVRLAL	2	22:17590180	IL17RA
WASGRITY, RLGWASGRITY	2	12:20522723	PDE3A
DASGGLRPDAL	2	10:135012429	KNDC1
SYKTSKRFSSL	2	19:57967221	VN1R1
FIDIKSKFAL	2	8:19805714	LPL
ELYDRGPPL	2	19:49850473	TEAD2
VHVCRDHVF	2	X:53564590	HUWE1
LELSKKKYFPL	2	1:100378027	AGL
LLDPDWVCPPV	2	7:21942715	CDCA7L
LAGVISALQTL, LAGVISAL	2	15:41099899	ZFYVE19
TAVRIVAGPSV	2	15:41099899	ZFYVE19
CPMRKKVPQ, VPQETRRCPRL, SPSCPMRKKV, CPMRKKVPQET	2	10:61828580	ANK3
LQNDAPVPYK	2	19:48879376	SYNGR4
HRPLAWGNINL	2	3:178927980	PIK3CA

CPRLPGVAS, PRLPGVASL, TRRCPRLPGV, CPRLPGVASL, VPQETRRCPRL, GVASLRLQRSL, SLRLQRSL, SLRLQRSLL, LPGVASLRL, CPRLPGVA	2	10:61828580	ANK3
TQAFNIGAL	2	5:21752211	CDH12
HEVQDKLREI	2	X:23751331	ACOT9
HAVANPGLIYL, HAVANPGLIY, AVANPGLIY, LIYLTGPY, VANPGLIYL	2	1:65131775	CACHD1
FALELKVTLPV, FALELKVTL	2	6:36452576	KCTD20
NRALHAAECL	2	9:13247771	MPDZ
WELASVRQAL, RQALCQLQW	2	8:145737816	RECQL4
EHDVALRREGL	2	7:100285170	GIGYF1
STFLTYMMCSY, YMMCSYMIDNV, YMMCSYMI, LTYMMCSYM, LSTFLTYMM, LTYMMCSYMI, STFLTYMM, LTYMMCSY	2	8:87126109	ATP6V0D2
GSMNRRPI, GSMNRRPIL	2	17:7577548	TP53
TRVMVPLPV, RVMVPLPVWM, TRVMVPLPVWM	2	3:3067806	CNTN4
CISMTTISR, ISMTTISR	2	17:48433967	XYLT2
MERLQAETKL	2	1:108145748	VAV3
KTFKFTSEL	2	1:232650179	SIPA1L2
SRHCPRRTRTM	2	20:49197838	PTPN1
FAMELLDGNLY	2	14:79175866	NRXN3
RSFMDLKI, CSVRSFMDLKI, MISETCSVR	2	5:72199545	TNPO1
IRELFILLQV, GRITIRELF, GRITIRELFIL, GRITIREL, IRELFILL, GRITIRELFI	2	15:40921506	CASC5
YQASSIPPTRL	2	16:3293377	MEFV
SRRAPRSPFL, SRRAPRSPF, RRAPRSPF	2	7:100285170--7:100283942	GIGYF1
VPASLFSL, LRQGRGLLTLV, VPASLFSLTL	2	7:99818416--7:99817848	PVRIG
ASRMRSMSATV	2	X:153070087	PDZD4
RRTSASRWWI	2	11:618999--11:618971	CDHR5
LYWADTKL, YWADTKLSF, LYWADTKLSF	2	11:68125269	LRP5
YRQWTHRGRL	2	7:149544824	ZNF862
VERCPWKGI	2	10:29760116	SVIL
TWTRCVPW, SMTAWPPTW	2	17:39780697	KRT17

FMMAFYLKY, KYTEKAWVMEL, FYLKYTEKAWV, YTEKAWVMEL, YTEKAWVM, KYTEKAWVM	2	18:12699829	CEP76
SVVKPSLFTV	2	19:12461741	ZNF442
WRIGGGTPL, WRIGGGTPLSI	2	1:27105931	ARID1A
LRMPESSGHTM, PESSGHTM, TESPPAQAVML	2	10:102762593	LZTS2
GRAGARGL, GRAGARGLPAL	2	10:100186986	HPS1
CLLHPFSAA, FSAACLLRQHL, LLHPFSAACLL, FCLLHPFSAA, HPFSAACLL, FSAACLLR, HPFSAACL	2	11:117222648	CEP164
FQVTWWLRRPV, RSFQVTWWL	2	12:132394804	ULK1
CASSTSRSSHM	2	16:2059622	ZNF598
WSQLAGIPL	2	3:195453018	MUC20
ISSFLNYF, ISSFLNYFV, VTISSFLNYFV, SSFLNYFV, ISSFLNYFVF, FLNYFVFKM, VTISSFLNYF, SSFLNYFVFKM	2	12:109947484	UBE3B
KRFVFIGEAI, FVFIGEAI	2	7:21730393	DNAH11
LQLREASPW	2	22:38477342	SLC16A8
GGYPAYSK, YPAYSKDSGLL, YPAYSKDSGL	2	15:45003781	B2M
FIWKRGSNTL	2	6:37623535	MDGA1
QRHSGKDWYL	2	4:42581868	ATP8A1
HSFENRAPQV	2	21:44323721	NDUFV3
SRNPVCSGANI	2	6:160511040	IGF2R
FMEQLCTY	2	2:183960324	DUSP19
LEKAFIEV	2	2:234750348	HJURP
LQIQTLLYGL	2	15:99679573	TTC23
TATAACPM	2	22:20130515--22:20130522	ZDHHC8
LSFASLRPRSV	2	16:28847350	ATXN2L
RTDGFAEAIHL, EAIHLPQV	2	1:186289548	TPR
LLHQIIHK, FLLHQIIHK	2	19:38230342	ZNF573
LPFRKNKM	2	13:52661488	NEK5
RRVTQALQASL, GRRVTQAL	2	9:137688741--9:137693829	COL5A1
DELNFRRQKLY	2	14:95574728	DICER1
QEKKVTRTI	2	7:136700754	CHRM2
VLPVKTLLEYL, TLLEYLEY, TLLEYLEYEK, KTLLEYLEY, KTLLEYLEYEK	2	1:220356133	RAB3GAP2
HRPQRQEPL	2	11:618971--11:618999	CDHR5

SPARASPARAS, RASPARASPA, SPARASPARA	2	11:117789327--11:117789342--11:117789346--11:117789345--11:117789327	TMPRSS13
IRFLCFTF	2	11:111742146	ALG9
RLISGSGSPSL, YMPPHLRGRLI, MPPHLRGRLI, SPSLWARV, AYMPPHLRGRL, SLWARVATPA, LISGSGSPSL	2	6:33273072	TAPBP
QESVVWLGL, QESVVWLGLKG, QESVVWLG	2	3:48612871	COL7A1
FLLPLVSS, FLLPLVSST, FLLPLVSSTA	2	1:156814053--1:156814343	INSRR
ARASGIEI, ARASGIEIYAV	2	20:43933021	MATN4
RLLFIIDSF, FIIDSFEEL	2	19:56369444	NLRP4
LPGQKYIVNV, IPDLLPGQKY, LPGQKYIV	2	2:216273098	FN1
QTARTGAPY	2	7:100285170	GIGYF1
SRMASTSC	2	2:43452623--2:43452512	ZFP36L2
YQTPYFLTV	2	1:99764662	LPPR4
IRAKTASIF	2	14:27066542	NOVA1
GEQIPGPL, GEQIPGPLV	2	2:43452635--2:43452622	ZFP36L2
ALLLLLLLLV	2	9:139581758	AGPAT2
MTMMTRRTL, MTMMTRRTLL	2	18:45567085	ZBTB7C
KSWIHSFRGVK, KSWIHSFR	2	16:27761525	KIAA0556
NASDAYPSAF	2	1:29138975	OPRD1
RAPRSPFLR	2	7:100285170--7:100283942	GIGYF1
RMVALVTGSV, ALVTGSVVV	2	1:43772910	TIE1
GANLRHLQAKK, NLRHLQAKK, GANLRHLQAK, NLRHLQAK, HLQAKKKFVK, LQAKKKFVK	2	10:71168685	TACR2
APPPPDPGTPA	2	7:6193521	USP42
ISPGPTSPSL	2	1:150530506	ADAMTSL4
ALVPPCPVPV, ALVPPCPV, SLGPHDALV	2	14:24619809--14:24619975	RNF31
LLSPSAVEL, LSPSAVEL	2	3:124739746	HEG1
SEFCFNDNLV, SEFCFNDNL, SEFCFNDNLVS	2	22:45802729	SMC1B
TSLISSLR, MTSLISSLR, LTVSRWMTSL, TSLISSLRVER	2	16:14346300	MKL2
FYVQTYQKKPM	2	6:10984132	ELOVL2
RTPTTRLMAPV	2	17:48433967	XYLT2
AQASPALA	2	11:117789313	TMPRSS13
FSMNSKRI, NRFSMNSKRI	2	16:81904511	PLCG2
QNDFQSLLKM	2	21:30701851	BACH1
KRHPRFPV	2	6:71011709	COL9A1

KAKIILKK	2	3:179306713	MRPL47
AQWAAPTPSLW, AQWAAPTPSL, HDAAAAAAQW	2	11:64534503	SF1
LQYWGAEL, LQYWGAELHL	2	1:161167952	ADAMTS4
ASLLFSIRR, ASLLFSIR	2	7:150698398	NOS3
RLGFLATAWL	2	10:17659131	PTPLA
RALTPGAPAL, AAASARAL, LTPGAPALPL, ARALTPGAPAL, HNDAAASARAL, ALTPGAPAL	2	16:31072705	ZNF668
GTSGVDCNHK	2	11:13375965	ARNTL
LPGPCASL, HPLGDTPQPSL, LPGPCASLL	2	3:51417604	DOCK3
LPKRLPEL	2	2:43452491--2:43452464	ZFP36L2
MAMTVIAGA, KMAMTVIAGA	2	3:77657038	ROBO2
AERAVETI	2	2:227661664	IRS1
VSACCAPR, SVSACCAPR	2	22:20130522	ZDHHC8
IFMCLRLCPHI, LRLCPHILDRL, LRLCPHIL	2	15:59376343	RNF111
SLLGLSLA	2	11:64572093	MEN1
DAYPSAFPSA	2	1:29138975	OPRD1
LLGERVWL	2	14:72117181	SIPA1L1
KSHLIVHQI, SQKSHLIVHQI	2	9:115805204	ZFP37
CASVAIGK	2	1:150530506--1:150529443	ADAMTSL4
SLFLGSHPFRA, SHPFRARIL, FLGSHPFRARI, AHFFRISLF, SHPFRARILCL, FLGSHPFRA	2	3:40529660	ZNF619
APRCPRSAV	2	1:150530506	ADAMTSL4
SVYENKLLNL, SVYENKLL	2	13:53616083	OLFM4
LPQSAAAAA, LPQSAAAA, LPQSAAAAAA	2	2:43452464--2:43452491	ZFP36L2
KMFLESPKL	2	1:92642631	KIAA1107
YEMTLHVKLPV, YEMTLHVKL, YEMTLHVKLP, YEMTLHVK	2	2:182387013	ITGA4
RWLWYCWPTWL	2	1:27106804	ARID1A
DEFNQIDAS, DEFNQIDASVL, QIDASVLSV, DEFNQIDA, DEFNQIDASV	2	12:124333344	DNAH10
EEASLKPKAAV	2	12:114395841	RBM19
LPSLPTMALL	2	14:20846338	TEP1
RLFPLLQSLL, YGSRLFPL, RLFPLLQSL, GRYGSRLFPL	2	1:54605319--1:54605318	CDCP2
TTKKPIIGILM	2	8:63951237	GGH
LEDHITHKTI	2	3:37367985	GOLGA4
VLDINDNPPLF	2	4:134071648	PCDH10

FEVCVCAC	2	17:7577121	TP53
SSRKRIIVI, RIIVINYII, KRIIVINYI, RKRIIVINYII, RIIVINYI, KRIIVINYII	2	10:63958149	RTKN2
CRSTGSLLKM	2	1:223178953	DISP1
WRKVLATPGL	2	14:24532703	LRRC16B
LEADMRRSL	2	19:33167455	RGS9BP
TSDTPKFNLI	2	16:28847356--16:28847350	ATXN2L
GPRTTTATMPT, GPRTTTAT	2	16:2059622	ZNF598
SEHIRLEKEM, LEKEMSKL	2	4:104032041	CENPE
GSYPPARQPSR	2	17:48433967	XYLT2
RSPAAAVRV	2	3:52551362--3:52550119	STAB1
SRRGPPTV	2	5:179193279--5:179193138	MAML1
LAHTQFLR	2	17:80400298	HEXDC
HWSSGWTL	2	1:55081757	FAM151A
YLNVFSLNYLF, NVFSLNYLF	2	14:35560282	PPP2R3C
SRHVCSHGDKL	2	21:43256586	PRDM15
ARQSCCLPGL, SRARQSCCL	2	1:27105931	ARID1A
SLSSWPPA	2	1:1290110	MXRA8
YLQCHHGNY, TTYLQCHHGNY	2	12:57921001	MBD6
TPATLTLAVA, CPKCSTPAT, TPATLTLA	2	19:10202224	C19orf66
GVAHKAILSPK, VLLSFGVAHK, LLSFGVAHK, VAHKAILSPK, IVLLSFGVAHK, LSFGVAHK	2	9:77397705	TRPM6
RSLLACCQL	2	19:51015404	ASPDH
LRWSSTRMSTV, RMSTVSQAPCL, LRWSSTRM	2	1:110169810--1:110168968	AMPD2
LLDSTNMNF, LLDSTNMNFK	2	6:3111145	RIPK1
LPDSSPPQL	2	14:94245571	PRIMA1
TLFASLCNL, FEDTLFASL	2	2:118578755	DDX18
CLLLLWLV, LLLWLVLVRL, VLVRLEPV, LLLLWLVLV	2	3:66550762	LRIG1
SAIAATVGSV	2	7:21942715	CDCA7L
TLNVKDIAPDK	2	8:13356915	DLC1
HTHTSSWRRPL	2	22:50297991	ALG12
FWRSLLACCQL	2	19:51015404	ASPDH
VLAAAGAVF	2	10:71906150	TYSND1
RLVGALSL	2	2:176988158	HOXD9
KGRRVTQAL	2	9:137677855--9:137688741	COL5A1
HYKDPSLCM, HYKDPSLCMF, HYKDPSLCMFF	2	2:46987060	SOCS5
SRTVGQCPAL	2	7:123332485	WASL

KLLNKLHK, KLHKYFHEPNK, KYFHEPNKKF, KYFHEPNKKFF	2	4:11400771	HS3ST1
IAFGIILNR, CIAFGIILNR, RMPISILYNK	2	9:2729545	KCNV2
CPAPRRAPA, APRRAPARCPA, RPGSRPSCPA	2	16:88599701	ZFPM1
RRLTRMPAW, LRTPPRRLTRM, LRTPPRRL, TRMPAWPSTL, RMPAWPSTL	2	9:95738900	FGD3
GAFYHRAQRL, FYHRAQRL, AFYHRAQRL	2	20:49626754	KCNG1
KEIWLSKNPYL	2	1:65278472	RAVER2
FMLGRMPTGM	2	19:38028415	ZNF793
MGAAVMRY, LLRGMGAAVM, FLYRFDLLRGM, YRFDLLRGM	2	22:50988105	KLHDC7B
RLTSTPNV	2	3:12645699	RAF1
RAGTRGSLV	2	16:2059622	ZNF598
LSSVICCLCAI	2	14:76121263	C14orf1
DSAGKTMLL, DSAGKTMLLY, MLLYKLKGHQL	2	13:50204663	ARL11
VRRAPLGTNF, VRRAPLGTNFV, KRVPEPSRRV, RRVRRAPL, SRRVRRAPL	2	X:92928044	NAP1L3
TESTPTASSAM	2	9:139945517	ENTPD2
TRSSNSVYL	2	1:34046429	CSMD2
RRRLGFLATAW	2	10:17659131	PTPLA
LTSTPNVHM	2	3:12645699	RAF1
KNWILVNLF, LFSGTSKAPF, NWILVNLF	2	7:23775341	STK31
FQYPLTISQL, VTWILFQY, GVTWILFQY	2	8:61135236	CA8
SRSTTAPV	2	22:46931196--22:46931227	CELSR1
FTITVRSL	2	6:161574408	AGPAT4
LEIISLDI, IYHPNVDKLEI	2	X:142967459	UBE2NL
NPVLPNEVL	2	19:45867259	ERCC2
KAFQFQTEL, NECGKAFQF	2	19:40521033	ZNF546
APTSCGPT, GPTRAPSSA	2	8:120220776	MAL2
NRGFRALL	2	9:137688741--9:137693829	COL5A1
GLKGEGSRSK	2	19:2226676	DOT1L
YRIRKAHSWPI, TPKWIRYFYRI	2	4:15689376	FAM200B
SRHGLIPLVNL	2	6:107100203	QRSL1
SALESHKI, SALESHKII	2	19:53058013	ZNF808
AERTLACV	2	19:17837425	MAP1S

VYSPQTQWI, QTQWIHFF, YSPQTQWIHFF	2	12:56231653	MMP19
TRNWVIQQKI, RNWVIQQKI	2	7:91726945	AKAP9
YYDSYSVDEHV	2	6:31860203	EHMT2
FHPWTTDMPA	2	2:171572940	SP5
METLCPAS, METLCPASRLA, METLCPASRL	2	17:36830727	C17orf96
SATHRIHEI	2	19:11978669	ZNF439
SMHPVVLV, VPSMHPVV, VPSMHPVVL	2	2:152727085	CACNB4
AQQCLAHTQFL, TQFLRALGSPW, AHTQFLRAL	2	17:80400298--17:80400208	HEXDC
FRLYSRAV, FRLYSRAVL, FRLYSRAVLV, RLYSRAVLV, KSIFRLYSRAV	2	X:53112052--X:53111923	TSPYL2
GANPHCFK	2	14:30100143	PRKD1
IMAVKRIWSTV	2	17:11998898	MAP2K4
ASRPPVTQRLV	2	2:70315174	PCBP1
YEGAATATDI	2	6:166571895	T
IQSYMLFLF, IVKNLLIIQSY, LIIQSYMLF, LIIQSYMLFLF, IIQSYMLF, LLIIQSYMLF, LLIIQSYM	2	1:225211543	DNAH14
SVWQACRK	2	4:37447379	KIAA1239
WPCPLCSPRWL, SPRWLRQA	2	3:183951135	VWA5B2
YLLRLWRI	2	8:23290534	ENTPD4
NTKPNRMSV, NRMSVAPY	2	8:38285864	FGFR1
VLYSFQPPSQL, FQPPSQLFAI, LYSFQPPSQLF, YSFQPPSQL, LYSFQPPSQL, SFQPPSQLF, YSFQPPSQLF	2	10:73326600	CDH23
LPLPPPVWAVL, LPLPPPVWAV, LPPPVWAV, LPPPVWAVL	2	14:104642560--14:104642555	KIF26A
FQAGALQLL, SYFQAGAL, SYFQAGALQLL, YFQAGALQLLL, ESYFQAGAL, YFQAGALQLL, FQAGALQLLL, SYFQAGALQL, FQAGALQL	2	20:44533618	PLTP
KIDDIFNKIKF, KIDDIFNK, KIDDIFNKIK	2	12:122437640	WDR66
SRPWLTTTWL, SRPWLTTTW, RMWWHPASRPW, WWHPASRPWL	2	2:219920405--2:219920368	IHH
TQVIFIAKI, HTQVIFIAK	2	19:42224988	CEACAM5
AFMNQEIMKAF	2	10:62648035	RHOBTB1

VRALAASGTWF, ALAASGTWFIL, ALAASGTWFI, ALPQSPAV	2	1:35370639--1:35370731	DLGAP3
WSWCGTSQTY	2	X:129190011	BCORL1
AYEIWHSDSF	2	2:179611639	TTN
HRASMQPIQI, HRASMQPI	2	11:71715795	NUMA1
FVIPPQTL	2	16:4944500	PPL
KIIGHFLFW	2	7:106513165	PIK3CG
ATASPSPL	2	9:98209617--9:98209593	PTCH1
LLKIPRTLK, SLLKIPRTLK	2	6:89793677	PNRC1
SAVMSANAAV, AVMSANAAV	2	8:22054260--8:22054259	BMP1
NEQKARGAAL	2	7:150698398	NOS3
KMIKWTLL	2	14:92472698	TRIP11
FEIEMNEI, FEIEMNEITNV, IEMNEITNV, IEMNEITNVGI	2	18:28914134	DSG1
RPQLRRWL	2	1:85040070	CTBS
KSNGPGLQY	2	7:107823349	NRCAM
KQVGFISL	2	2:152522864	NEB
CSHFPTPLL	2	16:68835781	CDH1
VTETNNSPLM	2	19:50547943	ZNF473
ARPCLPQAM, ASARPCLPQAM, SARPCLPQAM	2	1:53974841	GLIS1
RRNPTQGGLL	2	20:3870079	PANK2
YIIQTLSRVEF	2	6:118596709	SLC35F1
KRAQWVSVATL, KRAQWVSV	2	9:137688741--9:137693829	COL5A1
ASDGSDTSM, TASDGSDTSM	2	X:75649461	MAGEE1
LPWMVPWGMEA, VPWGMEAL	2	14:104642555--14:104642560	KIF26A
TAYWALAGLI, TAYWALAGL	2	1:116569517	SLC22A15
HPLVATQAL, HPLVATQALL	2	1:228559450	OBSCN
LRHEASRPLRL, LRHEASRPL	2	16:632283	PIGQ
WPLASPPA	2	8:10583646	SOX7
VIQDFIFFV, GVIQDFIFFV, IQDFIFFV	2	5:72199545	TNPO1
LLMRPAGAL	2	9:114393784	DNAJC25, DNAJC25-GNG10
FSDKMGTL	2	1:154313476	ATP8B2
SQIEIEAV	2	8:99115125	HRSP12
LTSTPNVHMV	2	3:12645699	RAF1
YTFASEPNNVF, YTFASEPNNV, FASEPNNV, FASEPNNVFF, FASEPNNVF	2	2:238249185	COL6A3
HRLSQLKPPVL	2	22:50962782	SCO2
KRERVDLSK	2	5:112175649--5:112175552	APC

VTHPPWWAAM, WAAMASACSL	2	16:67325658	KCTD19
WSSTCGGSM	2	16:2059622	ZNF598
TMAKTSAV, VTMAKTSAV	2	19:31768228	TSHZ3
VMMLEVCSRI, LEVCSRIQEF, TVPEVMMLEV, MMLEVCSRI	2	16:71602123	TAT
FTLRWSYNEL, LRWSYNEL, LRWSYNELGLL, LRWSYNELGL	2	8:73848578	KCNB2
IPGAHPASSAW	2	1:154942910	SHC1
AEVKHLHHLL	2	5:112175572--5:112175553	APC
YVMAGLGSPY, ILDEAYVMAGL, YVMAGLGSPYV	2	17:37881000	ERBB2
DELFTMMRSFI, LFTMMRSF, GFLSKDELF	2	15:45396172	DUOX2
FYFIPSQL, FYFIPSQLCEI, YFIPSQLCEI	2	11:59633897	TCN1
LAPPPPSAPL	2	14:75230947	YLPM1
CSSVSSSDGSF	2	3:78666908	ROBO1
IVHNLFVLR	2	4:3182271	HTT
QIQAATLQAK, FQIQAATL, YSMESRTHFQI	2	1:51753908	TTC39A
CRVRHERYFLL, VRHERYFLL	2	14:74004517	ACOT1
ERNRCVML, VMLFRQLHQEL	2	12:121746350	ANAPC5
NYWKRHIEVVM, YWKRHIEVVM, NYWKRHIEV, RHIEVVMREY, KRHIEVVM, KRHIEVVMREY, NYWKRHIEVV, YWKRHIEV	2	7:73021716	MLXIPL
RWRAAGF, ARWWRAAGF	2	2:38301663	CYP1B1
RMPARRESRFW	2	18:34205516	FHOD3
WMWAVEHSLPR, FLWMWAVEHSL	2	10:134158106	LRRC27
KDLAWLYFKIF, LAWLYFKI, KDLAWLYFKI	2	3:186281890	TBCCD1
RPAPRPTA	2	16:88599701	ZFPM1
CRLIGVTEI	2	21:45402199	AGPAT3
KLFDILDHYT	2	4:115544340	UGT8
RPRTARAAGAA, AARAARARV, AARAARARVA, RPRPRTARAAG, RPRTARAA, RPRTARAAGA, RPRPRTARA, RPRPRTARAA	2	16:88599701	ZFPM1
RTPMTTRL	2	22:18300932	MICAL3

MSPSLLDYLTM, MSPSLLDY, EMSPSLLDY	2	9:134183379	PPAPDC3
EAWWCRCPL	2	18:60639886	PHLPP1
IQEVTVNQSI, ILTPLNLQI	2	12:52913636	KRT5
VLFTGILY, SVLFTGILY, VLFTGILYV, ILYVDPVGNNY	2	1:216138672	USH2A
ASALPEALAV	2	17:80274142	CD7
IIDQLFAV	2	5:13928280	DNAH5
IEGQQVLQI, IEGQQVLQIAV	2	12:46230605	ARID2
RLRRLHRRLPR, RLRRLHRR	2	15:59981590	BNIP2
WPRCPSWCTA	2	16:88599701	ZFPM1
SLPRSTWHSV	2	16:50745399	NOD2
WSSSPTTPCPK	2	8:22054259--8:22054260	BMP1
KRTWSRPSL	2	14:59112895	DACT1
RDKLPFLLLI, KRDKLPFL, KRDKLPFLLLI	2	10:63958149	RTKN2
YFQVNITADSL, LRIQPAVKYF	2	1:117556033	CD101
ASACTMPPLL	2	17:48433967	XYLT2
FSCMRVASY	2	14:20846338	TEP1
TRMPGPVPA	2	17:61781804	STRADA
SFWPGGYPAY	2	15:45003781	B2M
AEHQAINTI, YRKAHAEHQAI, AEHQAINT, AEHQAINTIV	2	3:121155115	POLQ
GLAPETLSA	2	17:46629593	HOXB3
RRSSPPRRPL, SSPPRRPLQTL, RRPLQTLL	2	1:36643530--1:36643581	MAP7D1
LDLEPGGASAW, LEPGGASAW	2	19:1004710	GRIN3B
GYCFRMSL, WLRGHETGYCF, YLWLRGHETGY	2	X:39913253	BCOR
ETLNPMYV, MYVPCVKELL, NPMYVPCV, MYVPCVKEL, NPMYVPCVKEL, GETLNPMYV	2	19:10659690	ATG4D
MRLLMLAL, STMRLLMLAL, MRLLMLALLF, MSTMRLLM, MRLLMLALL	2	9:140033961	GRIN1
MQLYMQLLHEI	2	9:129870607	ANGPTL2
LLFPSTAHYL, TAHYLGAYV, FPSTAHYL, LFPSTAHYL, STAHYLGAYV	2	12:8075422	SLC2A3
FLCATEWLTL, FLCATEWLT, FLCATEWL, VMFLCATEWL	2	14:54898881	CNIH
IIFDTSWNPSY	2	X:76814235	ATRX
CPLICQQA, VPLCPLICQQA, HPVPLCPL, HPVPLCPLI	2	1:207139176	FCAMR
APQLTITSLSL, APQLTITSL	2	5:140215022	PCDHA7

GLWPWGAQV	2	20:23066469	CD93
KECQATARKF	2	1:76198353	ACADM
RWISEDLDGF, GFLRDWHSL, RWISEDLDGFL, WFSIVQTRW, SWFSIVQTRWI, SWFSIVQTRW, FLRDWHSL, FSIVQTRWI	2	5:13830840	DNAH5
FRQQNELKKTY	2	X:84526620	ZNF711
RTDCLLAVV, RTDCLLAV	2	5:57753012	PLK2
FAREDRIIRM	2	15:54919114	UNC13C
MQLMAIFKR, MQLMAIFK	2	6:76715225	IMPG1
ALRRAACLAH	2	11:76751542	B3GNT6
GELANIGLTL	2	2:238296564	COL6A3
ESNKLETICY	2	11:82976919	CCDC90B
FLFIQPEC	2	5:112175788--5:112175755	APC
AEDLLGVLAVF	2	1:200378558	ZNF281
WQIYQYNY, KIWQIYQY, QKIWQIYQY, KIWQIYQYNY	2	7:82579967	PCLO
VALPQPQPQSL, LAAVRGVAL, AAVRGVAL	2	12:111800827	FAM109A
KWCRPWLEQM, KWCRPWLEQML, SFSKWCRPW	2	X:56591874	UBQLN2
SRKHHHKL	2	2:171705836	GAD1
AAAPPVRLAR	2	2:43452439--2:43452491	ZFP36L2
RRQPPHHLVPL, RQPPHHLVPL, RRQPPHHL, RRQPPHHLV, RQPPHHLV	2	16:30736371	SRCAP
TRTVLQVTL, TRTVLQVTLSI, RTVLQVTLSIY	2	6:101094534	ASCC3
LRQGRGLL, LRQGRGLLTL, LRQGRGLLTLV	2	7:99818416--7:99817848	PVRIG
CRSSLPSPTL	2	1:35370639--1:35370731	DLGAP3
FEVRVCACT, FEVRVCACTG	2	17:7577106	TP53
FFSKPKRIKNL, KRIKNLSL	2	2:201436992	SGOL2
MERCTHCTG	2	16:2059622	ZNF598
SEGWHWGAAPM	2	7:120971879	WNT16
RRANKDATAEI, ATAEIIDEF	2	11:116656573	ZNF259
ILCQKLMQV, WLLQGIFSTTI	2	16:87866620	SLC7A5
LEGMLLPRAL	2	18:34205516	FHOD3
WRRRGSGTIM, RRRGSGTIM, RRGSGTIM	2	1:35370731--1:35370639	DLGAP3
SELVMRVQGNV, MRVQGNVYHL	2	1:87805290	LMO4
HRPGTPPPLI, HRPGTPPPLIL	2	2:74687410	WBP1
TRMTVQASL	2	2:220494118	SLC4A3

HQWLALLPL	2	16:28913639	ATP2A1
FQMHRMWQSF	2	19:20044269	ZNF93
GVAPRCPR, CPRSAVDI	2	1:150529443--1:150530506	ADAMTSL4
YSFQVAKDMEF	2	4:55958809	KDR
ALAEEPLI	2	18:72775210	ZNF407
LSELTMEL	2	1:39759288	MACF1
LKMMYHIF, LQMIWAFYL, QMIWAFYL	2	1:35972480	KIAA0319L
HQFKSFPEIYF, SFPEIYFL	2	17:42818751	DBF4B
AAPPVRLARV	2	2:43452439--2:43452244	ZFP36L2
SWFNGTELV	2	7:1097183	GPR146
LLSSCSSR	2	17:56448298	RNF43
RLLTRCCSSA	2	6:116783330	FAM26F
MCGSALAFFYR, SALAFFYR, GSALAFFYR, CGSALAFFYR	2	7:20371449	ITGB8
TSQPPQLINLK	2	8:59728228	TOX
ARTWPGIACRV	2	16:2059622	ZNF598
KTYPISEGLTM, TYPISEGLTM, YPISEGLTM	2	2:54093345	PSME4
VVVCVRPENTK	2	11:28119446	KIF18A
WTSWPTATWWA	2	8:145150876	CYC1
RSWTSKGWR, RSWTSKGWRRR	2	7:100285170--7:100283942	GIGYF1
PHSSGLAL	2	22:31485922	SMTN
YSYRGPQDFNY, YRGPQDFNYFV, SYRGPQDFNYF, YRGPQDFNYF	2	14:72171471	SIPA1L1
KVPVRAWLPR	2	2:43452439--2:43452491	ZFP36L2
LPGGKAEAAAV, FPFLSGLPG	2	16:30736370	SRCAP
IAITTGGRI, ITTGGRIVPR, TTGGRIVPR	2	5:10261684	CCT5
VAVKILLTF, FVAVKILLTF, FVAVKILL	2	6:13306704	TBC1D7
WFPTHVQVTVF	2	10:119805617	RAB11FIP2
RERLYQDTSL	2	13:20638677	ZMYM2
APSSAWRF	2	8:120220776	MAL2
TEVPSLTA	2	2:1481231	TPO
TPSEGRLDA, TPSEGRLDAL	2	16:54967096	IRX5
YLMEFGGTPSI	2	10:15107665	OLAH
ITHYHEMGLLY, LLYDYLQLTTL, HEMGLLYDYL	2	2:158622630	ACVR1
RSHTSMYI, LVRSHTSM	2	19:38028415	ZNF793
KTYRWTAL, YRWTALPW, KTYRWTALPW	2	19:50926264--19:50926165	SPIB

SSMNPTSL, RSSMNPTSL	2	7:100855927	PLOD3
LFYAKGTYGL, GQFRVSLFY, SLFYAKGTY	2	2:21235259	APOB
QTDSCTFYI	2	11:3123436	OSBPL5
YAPCCLSL, GLSDSYAPCCL	2	22:50315971	CRELD2
HSLEYSLQIQK, SLEYSLQIQK	2	3:115805269	LSAMP
FQLCLHYRYFI, CLHYRYFIPGV, YRYFIPGVAIA, FQLCLHYRY, YFIPGVAI, YRYFIPGV, YRYFIPGVAI, FQLCLHYRYF, YRYFIPGVA	2	9:120476537	TLR4
MPTLHTQV, MPTLHTQVQRV	2	16:633353--16:633354--16:633354--16:633353	PIGQ
LENIKTVI	2	1:76343855	MSH4
LLLGALGL, LLLGALGLKAL	2	18:43419003	SIGLEC15
FSLVVHQII, FSLVVHQI	2	19:58084067	ZNF416
RMVRGTQVY, LPLPNLRM, MVRGTQVYDGK	2	12:56478854	ERBB3
IEIVCRVF, IEIVCRVFSS	2	14:36217850	RALGAPA1
CQKTTLTLHQI	2	19:37118166	ZNF382
LLPAPSALVLL, SWISVPCPNLL, LLPAPSALV	2	16:30736371	SRCAP
STINSDGSV, STINSDGSVTV	2	X:8538695	KAL1
HATPQTQLPK	2	16:50745399	NOD2
YWEALGAPEAL	2	19:622336	POLRMT
VVVGAVGV	2	12:25398284	KRAS
GRAAARAV	2	2:43452464--2:43452439	ZFP36L2
TWHVAHTFL, TWHVAHTF, STWHVAHTF, TWHVAHTFLLM	2	11:57185284	SLC43A3
FLWPGTSPHRL	2	X:128926360	SASH3
GEGPDTFSCSW	2	22:21982892	YDJC
RLLTRAHVECL, LTRAHVECL	2	4:1389117--4:1389384	CRIPAK
MLYGNDRFEGY, FVMFRKSDRM	2	1:37307503	GRIK3
LILAVFSPV, YLILAVFSPV, ILAVFSPV	2	19:47969003	SLC8A2
EVKHLHHLL, EVKHLHHL	2	5:112175552--5:112175572	APC
RFPQSLYRGTV	2	11:619789	CDHR5
WRFCSGVLSG	2	8:120220776	MAL2
LMNLTVLIYQF, QFSRLQPSKF, MNLTVLIYQF, YQFSRLQPSKF, VLMNLTVLI	2	5:13830840	DNAH5
LRWLKRDSYL, IHMDCLRWL	2	12:133250289	POLE
VDLNTNFKKI	2	7:84727134	SEMA3D

TRASCLGWSM	2	19:7592423--19:7592444	MCOLN1
CENSLPYQVSL	2	9:33796691	PRSS3
ATWWCTGAATR	2	2:60688969	BCL11A
FTVPSNRSREV	2	5:112128184--5:112116559	APC
RWAAASKL	2	19:52715982	PPP2R1A
YTVCRFSI	2	3:62254809	PTPRG
LEERGSPQYLV	2	6:75875478	COL12A1
YCINLAALSF	2	12:65857005	MSRB3
TVIAGAHHCQY, IAGAHHCQY, GAHHCQYKLTY, VIAGAHHCQY, MAMTVIAGAH	2	3:77657038	ROBO2
ATDKWAFFI, ATDKWAFF	2	1:156912567	ARHGEF11
CSSWRSSSPR, SSWRSSSPR	2	6:41555186	FOXP4
WLWWWIWGLL, FRHWWLWWWI, WLWWWIWGL, WIWGLLQW	2	12:53207602	KRT4
AECNVHSVCI, AECNVHSVCIA, AECNVHSV	2	18:7032076	LAMA1
SLHQITHFI	2	4:122756323	BBS7
GLELKTINY	2	6:43305257	ZNF318
FMLILSIAT, TLNFSVATFML, FMLILSIA	2	10:94243045	IDE
KLAMKIGGV, LAMKIGGV, SVRSFMDLKIK, FMDLKIKL	2	5:72199545	TNPO1
CPTTPAPL, LPSQRRNHWM	2	17:7577065--17:7577036	TP53
FLLKGILALAI, FLLKGILAL	2	9:98209593--9:98209617	PTCH1
SRSVQQLQTSF	2	3:137983011	ARMC8
LRIASQVLI	2	16:88494506--16:88494491	ZNF469
RRQQKKEPLII, RRQQKKEPL, RRQQKKEPLI	2	5:31322961	CDH6
SMDASSSKM	2	10:93220255	HECTD2
LENKLKAAYM	2	15:56739117	MNS1
ALDKPCHQAEV	2	5:112175552--5:112175553	APC
TRNPRRSL	2	1:156715104	HDGF
KDHIFSFNLV, FSFNLVNIKDF, KDHIFSFNL	2	7:83764139	SEMA3A
LPMNWSPL	2	15:65678360--15:65678319	IGDCC4
VRPPPGPPCL	2	5:14487781	TRIO
RETPQNHSL	2	18:19144267	ESCO1
TPLLNGLDYTV, TPLLNGLDY, LPSHTPLL, ISSLPSHTPLL, LPSHTPLLNGL	2	1:180243511	LHX4
LEGGQAEVQL, AEVQLRRNA	2	16:3304463	MEFV
RVCLFLKR	2	3:65346916	MAGI1
ALACSLEEK	2	2:36825137	FEZ2

RLTWGRRR	2	7:102715804	ARMC10
KEICIEKL	2	14:92471873	TRIP11
YGNPIEASLY	2	16:20335551	GP2
REILDGLVEI, REILDGLV	2	7:43918139	URGCP
SRQGYDVHSDV	2	17:12028657	MAP2K4
RYKDLVLNETL, YKDLVLNETL	2	10:101455842	ENTPD7
STDDQIIVSYL	2	6:24566874	KIAA0319
HRNAYQTEF	2	7:94539496	PPP1R9A
VTANSWLPF, TANSWLPF, SSMPHGVV	2	17:79803764	P4HB
NRHWHWSSGSL	2	2:79349974	REG1A
RPAPSIWGMLL	2	8:28210809	ZNF395
CAWASTTRSSL	2	19:16314384	AP1M1
VLNPCLPSI, GMFVLNPCL, VLNPCLPSIML, FVLNPCLPSI	2	22:38895455	DDX17
RYFLKRQEM, YFLKRQEM, LRRYFLKRQEM	2	13:95735432	ABCC4
TVSYLKTQK, MTTVSYLKTQK, VSYLKTQK, GNMTTVSYLK, MTTVSYLK, TTVSYLKTQK, NMTTVSYLK	2	3:39159652	TTC21A
FEATEKKIF, FFQKCIYI, TYLFEATEKKI, IFFQKCIYI, FEATEKKIFF	2	1:87025657	CLCA4
EHSGSAGVV, EHSGSAGVVAL, EHSGSAGV, EHSGSAGVVA	2	19:17039884	CPAMD8
SLILSFQRV	2	5:36985083	NIPBL
SPVFLNGNYFI, FLNGNYFI, YFIQGPSGVIL	2	14:61186924	SIX4
VPAQRLQEA	2	8:144996029	PLEC
LSFAYMPPHL, YMPPHLRGRLI, AYMPPHLRGRL, MPPHLRGRLI, FAYMPPHL	2	6:33273072	TAPBP
QPLQRAAAAL, ALAPTDRLQPL	2	17:70120194--17:70119909	SOX9
EDMVVALVLAW	2	17:39742887	KRT14
FGMALGTLF, FGMALGTL, TLFVHEHF, FGMALGTLFV, MALGTLFV, RHFGMALGTLF	2	2:233348755	ECEL1
TLALTVLKSR, QTLALTVLK, TLALTVLK	2	3:52740182	SPCS1
LRNPNKDVCLL	2	4:39929664	PDS5A
WRMKAPAKKTL	2	10:67680207	CTNNA3
PRHDGRLYPL	2	2:38301663	CYP1B1

MEVKVLWTSGA	2	14:70633480	SLC8A3
STGACWLSAR	2	16:50745399	NOD2
MEHDVALRRE	2	7:100285170	GIGYF1
SSAYGGGVPL, AYGGGVPL, SAYGGGVPL, AYGGGVPLL, GETSSAYGGGV, SSAYGGGVPLL, SAYGGGVPLL	2	4:84230034	HPSE
YLKIKHLLL	2	5:112175605--5:112175552	APC
KALADFICNK	2	3:148563303	CPB1
FANWPRPA, CFANWPRPAL, FANWPRPAL	2	17:7579716--17:7579316	TP53
YLNWDAVL	2	11:108098609	ATM
FTIDLVTTCI	2	15:69331226	NOX5
LRILPPPL	2	22:46931227	CELSR1
LYMHSLTHSM, YMHSLTHSM	2	16:20975196	DNAH3
AQWKAQVQSI	2	2:234580888	UGT1A8, UGT1A7, UGT1A3, UGT1A1, UGT1A5, UGT1A10, UGT1A6, UGT1A9, UGT1A4
TVVDASFFL, ISMEQVTVV, ISMEQVTV	2	5:138643806	MATR3
HLLTSSAYRL	2	12:110370961	GIT2
YVTERIIAI, YVTERIIAISF	2	2:218762616	TNS1
AADRAWGL	2	7:128852004	SMO
ITICAVSSL, ITICAVSSLL	2	5:140203464	PCDHA5
IRNHPRTQL	2	19:58880242	ZNF837
SLVVPSAWRV, WRVGGPLGSL	2	1:35370731--1:35370639	DLGAP3
LPMRTLDPQI	2	1:21220089	EIF4G3
NSSCMGGMNQR	2	17:7577538	TP53
KRVDLSKL	2	5:112175676	APC
FRASELLQSGL	2	15:35174815	AQR
ITIVIPRF, TRITIVIPRF	2	7:103230055	RELN
SPAREHPL	2	X:150869296	PRRG3
SSFSYLLL, SSFSYLLLI	2	14:23549379	ACIN1
QQEGKKHILVL	2	2:220417266	OBSL1
GEWELCRAAPG	2	1:200880978	C1orf106
AQAPPEVTL	2	20:23066469	CD93

SYLKTMYSSL, KREPRLSY, KTMYSSLFW, SYLKTMYSSLF, KREPRLSYL, YSSLFWSQF, MYSSLFWSQF, YLKTMYSSLF, KTMYSSLF, YSSLFWSQFPF	2	6:102503432	GRIK2
FVYGGNCF	2	1:100376324	AGL
KPQIIEVAPQV	2	10:13361209	SEPHS1
KTLHHLLK, RSKTLHHLLK, KTLHHLLKQLK, QTMPPSRSKTL, TLHHLLKQLK	2	5:112175606--5:112175605	APC
FTSPKIDSV	2	6:17637996	NUP153
TAMCNSYDVRL, AMCNSYDV, TAMCNSYDV	2	1:21016738	KIF17
FAVLGTMVMK	2	1:150529443--1:150530506	ADAMTSL4
IEPKPFLYI	2	7:93108747	CALCR
DETRQLCDL, DETRQLCDLRL, EEFFDETRQLC	2	3:178916876	PIK3CA
CMSSSLPPL	2	1:156814343--1:156814053	INSRR
FPDTNFPGL, TNFPGLITL	2	1:17668563	PADI4
WALVLETML	2	15:42371752	PLA2G4D
FRSLRALHSL, RALHSLQL	2	15:74467645	ISLR
TRLHGNIL	2	8:24167472	ADAM28
YPSATISWFW	2	11:113102999	NCAM1
SRKDLVLINSL	2	12:132490710	EP400
RMHVCGGSLL	2	16:1273490	TPSG1
SPALKQVSPA, ALKQVSPA	2	2:203420130	BMPR2
FTCMTSRGFSY, MTSRGFSY, GRMPLLFTCM, MTSRGFSYM, LTGRMPLLF, CMTSRGFSY	2	16:81904511	PLCG2
ATPCHLRYL, LRATPCHLRY, LRATPCHLRYL, LRYLMATDTV, YLMATDTV, RATPCHLRYL, LRATPCHL	2	6:80383444	SH3BGRL2
MENISPFRCYL	2	17:7577065--17:7576881	TP53
TMPPLLHPQW	2	17:48433967	XYLT2
YFIPPSSSAPF, FIPPSSSAPF, FIPPSSSAPFL	2	19:13446675	CACNA1A
LSATITLTV, LSATITLTVAV	2	5:140802769	PCDHGA11
APCPAQVL, APCPAQVLLRL	2	19:1004896	GRIN3B
YVDPSGGDCF	2	18:48591918	SMAD4
DRHTKFFEEL	2	5:145980011	PPP2R2B
LSQSSSGYFSF	2	2:111881594	BCL2L11
NQHCKTRNRYI	2	16:70219844	CLEC18C

KLFYSAKNTSK, SAKNTSKLLFK, YSAKNTSK, NTSKLLFKK, YSAKNTSKLL, TSKLLFKK, FYSAKNTSKLL, YSAKNTSKLLF, FYSAKNTSKL, NTSKLLFK, KNTSKLLFK, YSAKNTSKL	2	3:16343361	OXNAD1
SEGIYAIAV, SEGIYAIAVVL	2	11:101344457	TRPC6
YVISVDYKSF, SAPINNYV	2	7:31862779	PDE1C
FSTTVTNPQPM	2	19:10246814	DNMT1
HRTEHTTSSM	2	11:111742146	ALG9
YRTQPDHPSAL	2	1:981860	AGRN
STNSVCLML, STNSVCLM	2	15:50853889	TRPM7
WEPAPAPATY	2	16:84224967	ADAD2
RQSPKRLLLR, LRKDTSVKIKI, ERRQSPKRLLL, RQSPKRLLL, RRQSPKRLLL, RQSPKRLLLRK, LRKDTSVKI	2	3:148872896	HPS3
FVNGGELFSTY, STYKENGPF, TYKENGPFL	2	8:67752239	SGK3, C8orf44-SGK3
SAASYLWPSR, ASYLWPSR	2	14:89878534	FOXN3
WRAKISTM, WRAKISTMSTY	2	12:122018740--12:122018773	KDM2B
SERQMRTRTM, RQMRTRTM, MRTRTMGPPL, TRTMGPPL, ERQMRTRTM	2	18:74778307	MBP
TETTTGRPL, TETTTGRPLSL	2	10:124895627	HMX3
AVLGTMVMK	2	1:150530506--1:150529443	ADAMTSL4
TSIQTGYMPRK, SIQTGYMPRK, IQTGYMPRK	2	4:46930528	GABRA4
RRWSSQTCSL	2	19:5231449	PTPRS
LQDEGSSLW	2	3:155546024	SLC33A1
RECPPEPA	2	17:79899272	MYADML2
VVLTSDSPAL	2	7:23293803	GPNMB
GSMNRRPILTI	2	17:7577548	TP53
IQNAAFLSNI	2	13:20638677	ZMYM2
LYPPEIVTL, LYPPEIVTLL, KRLYPPEIVTL, LYPPEIVTLLI	2	15:62256915	VPS13C
LRDTPAMASSV, TRPEPPPAPVL	2	3:52550119	STAB1
RAENQQLTASL, AENQQLTASL, AENQQLTA, AENQQLTAS	2	22:37906262	CARD10
MEACSNGVLI	2	1:45228107	KIF2C
IVLALKHLHK	2	14:91413793	RPS6KA5
WRTPMTTRL	2	22:18300932	MICAL3
MMATCPCPQGW	2	2:227661664	IRS1

QEVLLSEDL	2	1:118642238	SPAG17
WRQEGPCI	2	5:141046035	ARAP3
YKFWNHIV, YKFWNHIVACV	2	8:36766859	KCNU1
IVSSITASLQF, VSSITASLQF, SSITASLQF	2	2:132237994	TUBA3D
VRKTPHPL	2	22:37900249	CARD10
VLQTGGTTHLK, QTGGTTHLK	2	5:36035970	UGT3A2
MEHDVALRR	2	7:100285170	GIGYF1
SIVDYVEVTV, SSIVDYVEV, IVDYVEVTV, GSDENEESSI, GSDENEESSIV, SIVDYVEV	2	8:123966118	ZHX2
SAILYVHRKI	2	4:436610	ZNF721
RELLLVGRYGG	2	17:18918510	SLC5A10
QPQFCSSIL	2	X:54497147	FGD1
LYPGNTTV, LYPGNTTVTI	2	8:110457666	PKHD1L1
STPSLHFL	2	16:84213651	TAF1C
FLGDWTLPMA, FLGDWTLPM, FLGDWTLPMAA	2	17:4719139	PLD2
LEIRKQRQHL	2	10:61828580	ANK3
YYVCMKELTL, FFCPVYYVCM, VYYVCMKEL, YYVCMKEL, KAFFCPVYYV, AFFCPVYYV, VYYVCMKELTL	2	19:12384448	ZNF44
GEAAFAAA, FAEGGGEAAF, GEAAFAAAAA, AEGGGEAAFAA, GEAAFAAAA, GEAAFAAAAAA, AEGGGEAAF	2	2:176988158	HOXD9
AVGPRQLPK, LAVGPRQLPK, GLAVGPRQLPK	2	2:43452439--2:43452491	ZFP36L2
PTPPHLHLF	2	12:58157486	CYP27B1
HRKSLTLHQII	2	19:57089719	ZNF470
QTQPRMSPL, SQTQPRMSPL	2	20:25457050	NINL
TRKGSWSL, TRKGSWSLM	2	1:156715104	HDGF
TVIKKRDQCF	2	11:124845049	CCDC15
QEKEAEKNY	2	5:112175951	APC
RPRTRLPRA, RAGPRRPPA, VQAAVRPRTRL, LPRAGPRRPPA, AVRPRTRL	2	16:88599701	ZFPM1
TRRGGWRAL	2	6:39272281	KCNK17
TLACVCAL, TLACVCALL, TLACVCALLV	2	19:17837425	MAP1S
LELKGSHHPCI	2	2:48030670	MSH6
ITLSNWWFV	2	17:73227439	NUP85
WHWGAAATTL	2	12:49374348	WNT1
HSPYWAPPV	2	X:70327614	IL2RG

EVAAQARASGI	2	20:43933021	MATN4
RARWSTSF	2	22:31485922	SMTN
ADWLCGAW	2	16:50745399	NOD2
ALHGILYVL	2	4:3234980	HTT
MPLHHGLTQKM, MISETCSVR	2	5:72199545	TNPO1
KVLQMDFLV	2	5:112175761--5:112175752	APC
QLLTSGMELK, LLTSGMELK	2	4:153245446	FBXW7
RASGHTPEAV	2	17:80049304	FASN
GRSPPATGGRM	2	1:204228411	PLEKHA6
MSNLHPLL, SMSNLHPLL, SMSNLHPL	2	6:12124863	HIVEP1
CWYCRRQNGYR, RQNGYRALMDK, WYCRRQNGYR	2	9:5897631	MLANA
VLLRPLHV, VLLRPLHVPRV	2	11:76751585--11:76751604--11:76751585--11:76751542	B3GNT6
RHPKVHLNTM	2	5:112175351--5:112175346	APC
QASRPRPAR, QASRPRPARR	2	19:5244243	PTPRS
FRHWWLWWWIW, WWLWWWIW, GGFRHWWLW, HWWLWWWIW, LWWWIWGLLQW	2	12:53207602	KRT4
RETHLHTY	2	10:114900984	TCF7L2
RERQEELQRQL	2	19:12876964	HOOK2
FQEEPMTGFL	2	16:28847350--16:28847356	ATXN2L
MATDTVEIMTV, YLMATDTV, LRYLMATDTV	2	6:80383444	SH3BGRL2
EVSELSSWGA, EVSELSSWGAR	2	5:140588663	PCDHB12
FRFWVEKL	2	18:12699829	CEP76
LRMVRGTQVY, LRMVRGTQV	2	12:56478854	ERBB3
REVLKKNW, REVLKKNWNSA	2	10:890939	LARP4B
IYITVKLMVCL, IYITVKLM	2	2:197645342	GTF3C3
TAWQLVPL	2	1:27106804	ARID1A
FSDHGMTNIFW, FSDHGMTNI, MTNIFWMDKV, FSDHGMTNIF	2	4:185038131	ENPP6
RRSLARASL, SRRSLARASL	2	6:30864547--6:30863251	DDR1
LQWMSGCDL	2	6:31239108	HLA-B, HLA-C
IEACRVLSH	2	2:96992986	ITPRIPL1
AVQPFTIGDNK	2	18:8113645	PTPRM
WSSTVGNVSPM	2	10:46999178	GPRIN2
RHLWHCTM, GRHLWHCTMF, GRHLWHCTM	2	19:38376035	WDR87
YFQYCQLNLDM	2	8:62212687	CLVS1
LPLLWAGVL	2	19:51728477	CD33

LPLQIKGEGM	2	16:71127814	HYDIN
HLEKAFRSY	2	X:13778510	OFD1
LTSKINLH	2	16:71509257	ZNF19
WPARRGPLTSA	2	3:114058003	ZBTB20
FGSTVASSHRF, ASSHRFCFHQY	2	7:91732038	AKAP9
CWPRRWPRRL	2	16:89598371	SPG7
EVCVCACPGR	2	17:7577121	TP53
YHHPDPREL	2	16:88951498	CBFA2T3
HLLKQLKPSEK, KQLKPSEK	2	5:112175594--5:112175596	APC
FLDDHQNKI	2	15:49048485	CEP152
RQPPPTTSF, RQPPPTTSFPF	2	16:30736370	SRCAP
CRHTGGAGV	2	11:6590002	DNHD1
SLGFKLTSV	2	12:30906661	CAPRIN2
FRVIQVSPFI, IQVSPFIL, FRVIQVSPFIL, IQVSPFILAL	2	3:58303179	RPP14
SAMPFKII, SAMPFKIIY	2	9:8484375	PTPRD
YHEEVLTV	2	X:1428338	CSF2RA
LEFAPSNHYF	2	17:74391933	UBE2O
NSKAVFFSMK, KAVFFSMK	2	1:202407190	PPP1R12B
FFCDVSSDVF, FFCDVSSDVFY	2	19:56321612	NLRP11
QEAPLAQW, QEAPLAQWVV, QEAPLAQWV, QEAPLAQWVVM	2	3:48612871	COL7A1
YAQNVVQI, AQNVVQIQK, YAQNVVQIQKL	2	12:64196001	TMEM5
SRSNSTSWSM	2	19:7592423--19:7592444	MCOLN1
ISMVHTTTIL, MVHTTTILYL, ISMVHTTTI, MTFLKGNISMV, FLKGNISM, ISMVHTTTILY	2	10:126100644	OAT
TERNLRPLSL	2	9:35043650	C9orf131
YYESVQTPGTY	2	1:16254645	SPEN
SPYPSCSSTPA, YPSCSSTPANI	2	2:204305618	RAPH1
ARHKAVEFL	2	5:112175351	APC
LLHFARKVL, FARKVLQM	2	5:112175766	APC
CARPATSASAA, CARPATSASA, AARCARPATSA	2	16:88599701	ZFPM1
IQVPNYSLW, IQVPNYSLWI	2	4:153881906	FHDC1
HTAKVNCLSF, KVNCLSFNPY, LVDAHTAK, HLVDAHTAK	2	X:16870937	RBBP7
CRRQNGYRALM, RRQNGYRALM, CRRQNGYRAL, RRQNGYRAL	2	9:5897631	MLANA

AMVQSHPMSL, MVQSHPMSLSL, MSLSLWPGPSR, VQSHPMSL, SHPMSLSL, VQSHPMSLSL, LAMVQSHPM, MVQSHPMSL, SRLAMVQSHPM, LAMVQSHPMSL	2	19:17039884	CPAMD8
RARGGTTIGEV, SPALRARGGTT, SPRRSPALRA, SPALRARGGT	2	12:122242658	SETD1B
SEKYLKIKHL	2	5:112175553--5:112175594	APC
TMPTGLQSRLF	2	7:97933623	BAIAP2L1
SRLPAISL, TLLSRLPAISL, SRLPAISLCCL	2	1:156715104	HDGF
GASAAGRAL, ASAAGRAL	2	11:380921	B4GALNT4
AESPGAAL	2	17:7462459	TNFSF13
VVMETLDHLV, QEAPLAQWV, QEAPLAQWVV, TLDHLVPRV, METLDHLV, METLDHLVPRV, QEAPLAQWVVM	2	3:48612871	COL7A1
GRALARGRL	2	11:380921	B4GALNT4
TPAAAQLL, MPLSTPAA, MPLSTPAAAQ, STPAAAQLL, MPLSTPAAAQL, MPLSTPAAA	2	7:99817848--7:99818416	PVRIG
ATTSSTSLSF	2	19:46269076	SIX5
RLLWQICR, QICRGNVYLK, WQICRGNVYL, MASFERLLWQI, WQICRGNVYLK	2	7:138444564	ATP6V0A4
KSMLVLHR	2	5:112176027	APC
LRRLHRRL	2	15:59981590	BNIP2
YVQERKNLIY, HTCLTLNY	2	4:84370084	HELQ
LELGPDGHQRL	2	8:144810138	FAM83H
ALLGSSCAPPL	2	18:3452223	TGIF1
YKLVVVGAV	2	12:25398284	KRAS
RRAPRRAWRL, RAPRRAWRL, MRRAPRRAWRL	2	20:3765819	CENPB
RAAAWRTAV, AAAWRTAV, RAAAWRTAVLL, AAAWRTAVL, RAAAWRTAVL, AAAWRTAVLL	2	11:76751585--11:76751604	B3GNT6
LIFKATSPL	2	7:1855851	MAD1L1
LQVDQLWDVLL, LQVDQLWDVL	2	1:15850613	CASP9
LLMWTPCTV, LLMWTPCT, LMWTPCTV, HLLMWTPCTV	2	10:94243045	IDE

STFSLYRGRAL	2	2:220309843	SPEG
HLVRFLLPV, FLLPVLQL, FLLPVLQLCPL	2	17:37627694--17:37627688	CDK12
QARASGIEI	2	20:43933021	MATN4
YATIQTESI, IMYATIQTESI	2	5:134235320	TXNDC15
CMYSPALNKMF	2	17:7579313	TP53
APTLTPAQLPT, LPTATTGESPT, TPAQLPTA, TPAQLPTATT, TPAQLPTAT, APTLTPAQL, LPTATTGES	2	19:10202224	C19orf66
WQSGAMWV, AMWVQPPWEQL	2	16:28913640	ATP2A1
FRVCSQISL	2	19:38103616	ZNF540
AAASSSSSF, SAAASSSSSF	2	1:27875371	AHDC1
LTVPVPESPAF	2	7:139167934	KLRG2
QVIEAMLVAAV, MLVAAVTATV	2	16:1502857	CLCN7
YMKMQDTSKM	2	6:144772609	UTRN
LLSSILSSL, ILLSSILSSL, LLSSILSSLA, ILSSLATPM	2	5:38407192	EGFLAM
RPRRGRRRRA, RPRRGRRRRAA	2	16:88599701	ZFPM1
RTHEITHTGEK	2	19:12637516	ZNF564
RMSRALWTL, MSRALWTLL, WRMSRALWTL, WRMSRALWTLL, RMSRALWTLL	2	16:633125	PIGQ
SSLQKHVK, YTDPSSLQK	2	12:57861838	GLI1
HSMRYFSTSV	2	6:29910558	HLA-A
KEASKKAM	2	7:100285170	GIGYF1
QRVAAGVLYEL	2	3:41277892	CTNNB1
SRSSHLVSHQI	2	19:44981496	ZNF180
ISKDDLIAY, SISKDDLIAY, LSISKDDLIAY, SKDDLIAY	2	2:23916296	KLHL29
WELCRAAPGPA	2	1:200880978	C1orf106
PPMPPPANPQL, MPPPANPQL, MPPPANPQLL	2	9:35043650	C9orf131
GEALQSVHEV, GEALQSVHEVF, HEVFSAPA	2	4:1165130	SPON2
SAPSAQQSL	2	3:114058003	ZBTB20
LIKRRGPYEM, YEMSPSLL, YEMSPSLLD, YEMSPSLLDYL, KLIKRRGPYEM	2	9:134183379	PPAPDC3
KQAMCLQLL, KQAMCLQL	2	6:33171324	SLC39A7
KQFGAQVHQR	2	12:22208151	CMAS
RQIAHGADAV	2	4:22414962	GPR125

FVIKHLSFL	2	1:92769903--1:92772747	RPAP2
RFFRFRGRLVW	2	19:54974318	LENG9
KTHSTSMHM, LSSSKTHSTSM, SSKTHSTSM, SSSKTHSTSM	2	10:94243045	IDE
ADYQAVNDEI, YQAVNDEI	2	1:223949290	CAPN2
YTVELRGATLL	2	17:43480123	ARHGAP27
SEARYKDVYV	2	2:152548665	NEB
HSIQDVQFM	2	8:113241029	CSMD3
VEVSESAPPA	2	X:99662505	PCDH19
YSSAPRWKACL	2	17:46135788	NFE2L1
SSASTRHVTPL, ASTRHVTPLPV, ASTRHVTPL, SASTRHVTPL	2	3:195506746	MUC4
SRTLVVFV, KYVSRTLVV, KYVSRTLVVFV, YVSRTLVVF, KYVSRTLV	2	4:141888889	RNF150
FLSLVHHTY, LQFLSLVHHTY	2	10:50025369	WDFY4
ASMVFQNHVEK, MVFQNHVEK, MVFQNHVEKK, SMVFQNHVEKK, SMVFQNHVEK	2	9:100257990	TDRD7
SRDLAKKKLFL	2	5:98206409	CHD1
RWSRAVLYHI, SARRWSRAV, RRWSRAVL, ARRWSRAVL, ARRWSRAV, SARRWSRAVL	2	3:19384132	KCNH8
YANLPEYF, YANLPEYFKRL	2	11:126126690	FAM118B
TVFGTLASLYL, TTVFGTLASL, TTVFGTLASLY, SPTTVFGTL	2	1:230846185	AGT
RIDNNSTASYL	2	2:165551820	COBLL1
SYHRLLPLW, SYHRLLPLWAA	2	2:43452464--2:43452439	ZFP36L2
HEIIHTGERPY	2	22:24086597	ZNF70
IPPTSRGSSL, PPTSRGSSL	2	17:16285785	UBB
RTVHAFAM, VRTVHAFAM, HAFAMEQR, RTVHAFAMEQR, TVHAFAMEQR	2	7:150733024	ABCB8
SPTEPSTEAL	2	6:32017322	TNXB
YTIKWFEV	2	11:114183101	NNMT
ITVPRCVKMAY	2	8:104312432	FZD6
AAVPPTTAGAL	2	11:64981481	SLC22A20
MQLQAIQI, IEMQLQAIQII, IEMQLQAIQI, MQLQAIQII	2	2:55756108	CCDC104
FVICWLPFL	2	3:88040823	HTR1F
YKQDRDITL, YKQDRDITLL, TLLDLINFFI	2	3:136287655	STAG1

MMGCDLVEV, NMMGCDLV, NMMGCDLVEV	2	1:15901300	AGMAT
CSSHFPYI, FPYIQYQFW, FPYIQYQF	2	3:46414947	CCR5
DRAVPHLSY, SEVQDRAVPH, SEVQDRAVPHL	2	2:43452464	ZFP36L2
VAPPTPPPL, QVAPPTPPPLL, VAPPTPPPLL, QVAPPTPPPL	2	2:204305619	RAPH1
QLLGADSWK, RVQLLGADSWK, LLGADSWK	2	18:3452223	TGIF1
AEAAAVVTA, AEAAAVVTAA, AEAAAVVT	2	7:100285170	GIGYF1
EAAAAAGVLY	2	18:48723146	MEX3C
GLQGSWWA	2	11:77920855	USP35
SRLGGPGRIL	2	6:39272281	KCNK17
YVIEWGLGPPA	2	1:36935323	CSF3R
TLNFSVATF, STLNFSVATF, FSVATFML, FSVATFMLIL, STLNFSVATFM, TLNFSVATFM, FSVATFMLI, VSTLNFSVATF	2	10:94243045	IDE
LREMVSNM	2	13:46288145	SPERT
AESLLLFLLT, AESLLLFL, AESLLLFLLTS, AESLLLFLL	2	3:148863159	HPS3
SELQPKISKSL, SLMQTIRCL	2	13:31735655--13:31732974	HSPH1
SRDVEALSLL, VEALSLLIF, EALSLLIF, VEALSLLI, VEALSLLIFL	2	11:57268319	SLC43A1
YPIPVTMVL, YPIPVTMV	2	4:5016903	CYTL1
HPGLRPGHLHL, RPGHLHLL, HPGLRPGHL, HPGGCADHPGL	2	8:145625525	CPSF1
GLRGLHAL	2	2:43452491--2:43452464	ZFP36L2
LLASWCVPK, LASWCVPK	2	16:50745399	NOD2
NVHFASVVNY, VVNYFIEKENY	2	13:48615145	NUDT15
KMIGGNLKI	2	2:238976762	SCLY
AQNIYVEL	2	3:89259138	EPHA3
NPSQPVPL	2	1:155308000	ASH1L
ILFPCLRL, ILFPCLRLL	2	6:116836954	FAM26E
RETEPCML, RETEPCMLL	2	1:156814053	INSRR
RMQLCTQLAR	2	17:56435161--17:56435252	RNF43
WRAGGVWAV, WAVGSAGCMEY	2	14:20852646	TEP1
MVPMNRMAK	2	4:119951693	SYNPO2
GMLQIYLL, MSGTGMLQIY	2	18:25565698	CDH2
HPWTGVGSAW	2	1:161068553	KLHDC9

GLLTASGSAAA	2	15:59981515	BNIP2
FMDQNQYAL, FMDQNQYALL, FMDQNQYALLL	2	2:116548905	DPP10
FSRSPIRRKI	2	21:34927647	SON
MLSALARPVSA, VSAALRRSF, ALARPVSAA	2	7:75677504	MDH2
SLQPGLGNV	2	11:66055105	YIF1A
RQIYPPSTCSL	2	2:71191573	ATP6V1B1
SSLPLPRHTSL, LPLPRHTSLT, LPLPRHTSL	2	12:25232370	LRMP
TVASSHRF, TVASSHRFCF	2	7:91732038	AKAP9
REMRAGGCC, REMRAGGC	2	10:46999601	GPRIN2
KQAVCFRAW	2	9:117071681	COL27A1
VPRRQCQA, RRQCQASARSF, VPRRQCQASA	2	9:98211549	PTCH1
KQDKCFSTF, KLKQDKCFSTF	2	13:38154705	POSTN
GREQRPEV	2	10:46998995	GPRIN2
AEAKATAETV	2	9:124855330	TTLL11
YRFDLLWGV	2	22:50988099	KLHDC7B
SELPPGRAW	2	12:110019240	MVK
FQIRGAAPPYI, AAPPYIYAV, GAAPPYIYAV	2	20:47271846	PREX1
SRPQLRRWL, SRPQLRRWLLV, SRPQLRRWLL	2	1:85040070	CTBS
KLDENNTPM	2	5:151051214	SPARC
FLYYKSIGPL	2	1:79392756	ELTD1
CQYLHEAY	2	13:32813960	FRY
YANDAFPEEYM	2	14:63671738	RHOJ
SRCDRVHIF	2	19:38958286	RYR1
RSSPPRRPL	2	1:36643581--1:36643530	MAP7D1
VPNYSLWI, YSLWIEAMVL, YSLWIEAM	2	4:153881906	FHDC1
KLFPLLFKV	2	8:145745194	LRRC14
RRSGWLGRL, CRRSGWLGRL, RRSGWLGRLHV	2	16:50745399	NOD2
YIFCLHYGTQL	2	9:114695180	UGCG
SSMPSFQQAF, LRADASSM	2	7:47344502	TNS3
SELKMVTSTGL	2	1:100889837	CDC14A
ATHQWPGSCPR	2	16:50745399	NOD2
KLHSLHPTSV	2	14:55817461	FBXO34
QRFPTLTV, RPLCQRFPTL	2	16:14346300	MKL2

RMMMTMPIAM, MMMTMPIAMCL, IAMCLNLNF, MMTMPIAM, RMMMTMPI, TRMMMTMPIAM, MMMTMPIAM, TRMMMTMPI, MTMPIAMCL, KTRMMMTM, MTMPIAMCLNL	2	X:39913253	BCOR
SQSFFGLF	2	19:48018113	NAPA
TYKPQDVVHIL	2	7:122341990	RNF148
AQWVSVATL	2	9:137693829--9:137677855	COL5A1
LEEHDGHAVSI	2	2:145162528	ZEB2
QNDYFIRQQY, IQNDYFIRQQY	2	13:60565310	DIAPH3
AVLIFLPGYDK, VLIFLPGYDK, LIFLPGYDK	2	5:112889319	YTHDC2
VSFEASMALL, VSFEASMAL	2	20:37464776	PPP1R16B
LLSRLPAI, GRSRTTLLSRL, SRTTLLSRL, GRSRTTLL, TLLSRLPAI	2	1:156715104	HDGF
FQRGLPWRRL, LPWRRLDAL	2	15:101427829	ALDH1A3
SQSRSPVPK, RSSQSRSPVPK, SQSRSPVPKK, SSQSRSPVPKK, SSQSRSPVPK, QSRSPVPK	2	18:34205516	FHOD3
RLAAKGWRIPL	2	15:84639305	ADAMTSL3
LPLVPRPL	2	15:93588289	RGMA
VPACWVTCL	2	12:57921401	MBD6
IIYSIPPA, AFRDRNTRIL	2	11:58962827	DTX4
RPGGWNRM, NRMGEPRGF	2	1:36935323	CSF3R
GTNVVFTTK, SSGTNVVFTTK	2	5:38510602	LIFR
ESALQNKM	2	20:36940320	BPI
KTPPTNAL	2	5:137803132	EGR1
CSMQDPLLSL	2	3:168833257	MECOM
LRDGCMLIKL, LRDGCMLI, LRDGCMLIKLL	2	14:65271701	SPTB
DPSGGDHF	2	18:48591919	SMAD4
RRTMRPLPGRI	2	18:658064	C18orf56
RELALFLTPD, RELALFLTPDP	2	20:49411698	BCAS4
DEAVSTEVCLM, TEVCLMITDAA	2	5:71482444	MAP1B
FTDDPITEF	2	8:121290381	COL14A1
WSHHTLPI, HVWSHHTLPI, APRHVWSHHTL	2	19:990281	WDR18

IQYALDQGMNV, ALDQGMNV, GMNVTQVFV, IQYALDQGM, ALDQGMNVTQV	2	19:12918306	RNASEH2A
QESSFIWPSFM, FIWPSFMVMTM, QESSFIWPSF, SFMVMTML, FIWPSFMVM, FMVMTMLRNI, FIWPSFMV	2	7:21942715	CDCA7L
ITFEGCQPPTV	2	11:76256898	C11orf30
WAFTDTFL, WAFTDTFLNL, LQWAFTDTF, FTDTFLNLTL	2	11:76751166	B3GNT6
LAAGLKSSIIM	2	6:36452576	KCTD20
SASQFQSDKY	2	13:23904858	SACS
MTSSRCWTHCL	2	15:75942664--15:75942736	SNX33
AESNLFLA	2	4:151829982	LRBA
TFSSVPLVASI	2	1:22191454	HSPG2
VSLTSFLF	2	5:72199545	TNPO1
CSRPLCSRPL	2	7:128587374--7:128587381	IRF5
ATYPKFLANAL	2	1:112303389	DDX20
VPPPHLCLPL, MPPQVPPPHL, VPPPHLCL	2	1:204438071	PIK3C2B
KQICIPRSL	2	3:170013719	PRKCI
LYVENQFCEF, YVENQFCEF	2	3:195798938	TFRC
HRALQLLHEV, HRALQLLHEVL, HEVLHTMPI, HEVLHTMPIA	2	18:48604787	SMAD4
YTYQDPRGI, YTYQDPRGIY	2	19:36317520	NPHS1
MAASVWLLTSV, MAASVWLL	2	3:52543930	STAB1
YIDPSPDYCM	2	3:55504335	WNT5A
FSHHPSGSTK	2	18:34205516	FHOD3
WFDQHQIIHFL	2	7:99084495	ZNF789
LLDDSITIYI, LLDDSITI	2	3:113138965	WDR52
GPAATSSL, WAGPAATSSL	2	19:17836781	MAP1S
YLDDRNIFR, EYLDDRNIFR, YLDDRNIF	2	17:7578217	TP53
SRHTKFGYGVL, SRHTKFGY, SRHTKFGYGV	2	6:121562685	C6orf170
ILSTKASNTI	2	6:116836954	FAM26E
RTVASSPM, RTVASSPMEPL, AAPRTVASSPM, KMWCPAAPRTV, VASSPMEPL	2	1:53974841	GLIS1
TMIQDTQWV, ILTMIQDTQWV, KSTQNGILTM, MIQDTQWV, STQNGILTM	2	5:138643806	MATR3

IYKWVPVM, LRIYKWVPVM	2	12:122468668	BCL7A
GQISTLVL, FVGQISTLV	2	4:15067858	CPEB2
ILWNVSSLI, ILWNVSSL	2	5:112170822	APC
WMAPKVIKSSL, TYAWMAPKV, GTYAWMAPKV	2	1:233482322	KIAA1804
WRWWTWGGL	2	3:196674360	PIGZ
QMNDARHGGW	2	3:178952085	PIK3CA
QETAKFFL, QETAKFFLSL	2	19:6183180	ACSBG2
CRPWLEQML, CRPWLEQM	2	X:56591874	UBQLN2
HTAPSLGL, SPWGSHTAPSL, SHTAPSLGL, WGSHTAPSL	2	19:17039884	CPAMD8
STISQNHTPTV	2	8:145150876	CYC1
LLWQLSIL, LSFCFSTI, KLLWQLSI, CLWKLLWQL, CLWKLLWQLSI	2	17:19729452	ULK2
SPWVRPRRRLL, WVRPRRRLLDL, WVRPRRRL, SPWVRPRRRL	2	22:38477342	SLC16A8
GQSSSLIHHQI	2	8:146067469	ZNF7
RTATWVRNLTL, ATWVRNLTL, RRRTATWVRNL, RRTATWVRNL, LRTPPRRLTRM, LRTPPRRL	2	9:95738900	FGD3
EASQNLSTPPL	2	1:233397867	PCNXL2
NEVKDLLRCL	2	7:92760715	SAMD9L
ISIYLTYQKF, TYQKFLCRNM, SIYLTYQKF, FAISIYLTY, KFAISIYLTY	2	12:9885707	CLECL1
QVDQLWDVLL	2	1:15850613	CASP9
RRAEQRRHL, RRHLQHRDL	2	17:70119807--17:70119780	SOX9
RARARAPAA, RARARAPA	2	16:88599701	ZFPM1
APGLLWMGLAL	2	11:6411935	SMPD1
APPLHRRAA	2	18:3452223	TGIF1
RVSPRPLSPL	2	13:111368164	ING1
FFFSFFYFLWI, FFYFLWILWL, FFFLFFFSF, YWFFFLFF, TYWFFFLF, FYFLWILWL, FFFSFFYFL, YFLWILWL, FFFSFFYF, YWFFFLFFFSF, FYFLWILWLI	2	6:153019117	MYCT1
SQNNLYRYV	2	19:12433451	ZNF563
QRVTVIRRGM	2	1:156715104	HDGF
TVALTIIHR, GTVALTIIHR	2	2:40656129	SLC8A1
ILTHIIECL	2	5:57753012	PLK2

RDAVASAW	2	1:228504670	OBSCN
VRVPSVTL, VRVPSVTLV	2	18:44400988	PIAS2
ARWESPWPV	2	19:17039884	CPAMD8
SSMFLRFYL, RFYLDASVQRK, LRFYLDASV, SMFLRFYL	2	10:98133400	TLL2
HEQVYIQS, HEQVYIQST	2	3:132051046	ACPP
YTLRVQAL, AQYTLRVQAL	2	18:59166573	CDH20
GIQSHSLK, CLNGIQSHSLK	2	1:235969998	LYST
LLWPGTALILL, LLWPGTALI, LLWPGTALIL, LLWPGTAL, GLLWPGTALI	2	8:144945516	EPPK1
LHQNRFSTF	2	12:51497497	TFCP2
PPVNRGFRAL	2	9:137688741--9:137677855	COL5A1
HPVLTPRAG, TPRAGSRTT, SPTCLGHPVL, TPRAGSRTTC, HPVLTPRA, TPRAGSRT	2	19:10202224	C19orf66
SNYKLFLLF, KLFLLFLAYSL	2	8:17063200	ZDHHC2
FQFYLTGVTQM, YLTGVTQMAYL, YLTGVTQM, FYLTGVTQMAY, FQFYLTGV	2	17:56693650	TEX14
AAAVVTAAF, AAVVTAAFTK, AVVTAAFTK, AAVVTAAF, AAAVVTAAFTK, VTAAFTKEASK, AAFTKEASK, AAFTKEASKK, VVTAAFTK	2	7:100285170	GIGYF1
KLCSCHPV, RPFSAPDACKL	2	12:96077487	NTN4
FMFEGWEIKLV, FMFEGWEIKL, FMFEGWEI	2	2:84864358	DNAH6
LESLVFPG	2	3:48612871	COL7A1
LAGPGLGGY, HLAGPGLGGY	2	9:139752899	MAMDC4
TTYDRHFVI	2	17:36936775	PIP4K2B
FMKITMAKQLL, ITMAKQLLKI	2	8:113241029	CSMD3
LVAARTLPV, TLVAARTLPV	2	12:132394804	ULK1
LEASINPARM, SRSLSLPL, LEASINPARMV, LRSRSLSLPL	2	17:43482356	ARHGAP27
RVCACPGRDWR	2	17:7577094	TP53
HRRCHKFVTF	2	19:54387447	PRKCG
SAAWRSCIAL, IALWCASSV, AAWRSCIAL	2	1:27106804	ARID1A
RFLHVFFF, RFLHVFFFL, RLKRFLHVFFF, RFLHVFFFLHF, VFFFLHFL, KRFLHVFFF	2	2:230661315	TRIP12
YNPYGPQTL, LYNPYGPQTL	2	3:37150132	LRRFIP2
EELDALKKPL	2	9:106885501	SMC2

STPATLTLAV, STPATLTL	2	19:10202224	C19orf66
FLYTDLFLSL	2	14:88792776	KCNK10
CANSWSTWLSL, CANSWSTWL, NSWSTWLSL	2	19:17836781	MAP1S
CLHGASCLDGV	2	9:126128253	CRB2
IAFTTATKM	2	13:37569724	ALG5
KEITVRGGMFV, KEITVRGGMF, KEITVRGGM	2	22:38895455	DDX17
YRPWRGTT	2	8:67341501	RRS1
LSNPLCASPSY	2	7:150034378	LRRC61
YLDRCFARNRV, FARNRVCI, FARNRVCIS, FARNRVCISF, FARNRVCISFG	2	3:194353875	TMEM44
LLANDPTYL, TLLANDPTYL	2	1:11184573	MTOR
EEWALLDLAQI	2	19:9413107	ZNF699
LQPPALSSVF	2	17:7906519	GUCY2D
EFYKLIRFLCF, CFTFCDVF, RFLCFTFCDVF, CFTFCDVFWLL, CFTFCDVFW	2	11:111742146	ALG9
RPNQQQT	2	19:58879976	ZNF837
RPHSCLADVL, RPHSCLADV, RPHSCLAD, MFRRPHSCL, RPHSCLADVLL	2	5:112175350--5:112175351	APC
ERVGRHVL, ERVGRHVLL, ERVGRHVLLI	2	22:24225987	SLC2A11
WQNSIWHNL, WQNSIWHNLSL	2	7:4796634	FOXK1
TMPPSRSKTL	2	5:112175605	APC
SPRRGSSPACS, SPRRGSSP, SPRRGSSPAC	2	1:17265560	CROCC
MALCQNAREM	2	3:145796919	PLOD2
GSYGCRLGF, KTYQGSYGCRL, KTYQGSYGCR	2	17:7579361	TP53
CASVAIGKLAL	2	1:150530506	ADAMTSL4
FFEEGMWLQY, FEEGMWLQY	2	7:147914416	CNTNAP2
FLLITITL, FLLITITLGT	2	17:34313637	CCL14
HLSYHRLLPL	2	2:43452491--2:43452464	ZFP36L2
EPPKGVDPGAL	2	12:52215102	FIGNL2
REDSRRHGRNW	2	19:47768128	CCDC9
VPACSHVPI	2	4:1388944	CRIPAK
LRRKVPAQRL, RRKVPAQRL	2	8:144996029	PLEC
EEFRDQWGV	2	11:380921	B4GALNT4
STRFLTVPV	2	7:139167934	KLRG2
SVRSFMDLK, FMDLKIKL, CSVRSFMDLK, KMISETCSV, MISETCSV, SVRSFMDLKIK	2	5:72199545	TNPO1

ILFDGPMDTL	2	17:7690256	DNAH2
FARNNATKLI, ARNNATKL, FARNNATKL	2	10:7605141	ITIH5
RPRPRDPL, LGRPRPRPPL	2	16:88599701	ZFPM1
PPSRSKTL	2	5:112175605--5:112175606	APC
RVRFFRFR	2	19:54974318	LENG9
ITWQHSTGQLY	2	20:53260081	DOK5
NDDLFMMPQI, FMMPQIVNV	2	15:42058229	MGA
KEIPLLQLSL	2	12:77449827--12:77449858	E2F7
ILPEFHILTV	2	3:151046142	P2RY13
KEFVCHWGA	2	12:57860075	GLI1
SLFLGSHPF, ISLFLGSHPF, GSHPFRARIL	2	3:40529660	ZNF619
MISETCSVRSF, FMDLKIKLAM	2	5:72199545	TNPO1
AEFKKETFDVM, KETFDVMLEPL, KETFDVML, FDVMLEPL	2	6:6174830	F13A1
WRMWWHPA, WWHPASRPWL, SQSMGHWWW, RMWWHPASRPW	2	2:219920405--2:219920368	IHH
FVDLWALVLET	2	15:42371752	PLA2G4D
KPTIRLTV	2	3:151046142	P2RY13
RTMTTTKM, MTRRTMTTTKM, RTMTTTKMMMM	2	18:45567085	ZBTB7C
YQSSIHSKYKI	2	1:227843477	ZNF678
MATKMATL	2	6:11185508	NEDD9
FIICGMLYV	2	9:138011798	OLFM1
RWATWLSLAL, RWATWLSL	2	10:124895627	HMX3
SPMTPMTPM	2	22:38483155	BAIAP2L2
MAALVRPSRF	2	17:685640	RNMTL1
KIYPPSGSPK	2	11:128842602	ARHGAP32
LQPSKFPLF, YQFSRLQPSKF, SRLQPSKF, RLQPSKFPLF, SRLQPSKFPL, QFSRLQPSKF, SRLQPSKFPLF	2	5:13830840	DNAH5
AQRWSCHTL, QRWSCHTL	2	1:110169810--1:110168968	AMPD2
GAREAGRFAVL	2	1:150530506	ADAMTSL4
KRAANIAEALY, KRAANIAEAL	2	5:158140131	EBF1
GRLRGPACL	2	2:43452342--2:43452464	ZFP36L2
ALMTFLLA, FLLASWCVPKV, ALMTFLLAS, FLLASWCV, RQHRSALMTFL, LMTFLLASWCV, RQHRSALM, LLASWCVPKV	2	16:50745399	NOD2

LPEALAVISFL, LPEALAVISF, LPEALAVI	2	17:80274142	CD7
SRKSKLVVEV, SRKSKLVV	2	11:119181151	MCAM
MIVQCTVEV, GMIVQCTVEV, GMIVQCTV, MIVQCTVEVEV	2	5:140768431	PCDHGB4
LLAPGSFSTCI	2	10:101544462	ABCC2
RLFLPGSTPSL	2	16:84213651	TAF1C
SEIGATPPA, SEIGATPPAA	2	10:13696452	FRMD4A
FQMWISISL	2	6:161469829	MAP3K4
VSSTFRSYLV, LASHTVSSTF, ASHTVSSTF, RSYLVTISIKL, STFRSYLV, HTVSSTFRSYL, STFRSYLVTI	2	10:127483521	UROS
MENFCRAIGLR, MENFCRAI, MENFCRAIG, MENFCRAIGL	2	3:127831743	RUVBL1
FFENLDSRQKF	2	14:96761299	ATG2B
ILDTAGHEEY	2	12:25380275	KRAS
YEIRICRPCEI, YEIRICRPC	2	9:5968511	KIAA2026
TRLHTAHSTL	2	17:70120314--17:70120194	SOX9
VEEQSITIVW, EEQSITIVW, EEQSITIVWGL, EQSITIVW	2	1:228466896	OBSCN
IQSKDTTI	2	1:151552161	TUFT1
ALDRRFYL, IALDRRFYLM, IALDRRFYL	2	1:93169000	EVI5
FTHNHFQFFCL, LRICQSIVL, FQFFCLRI	2	13:67800833	PCDH9
RANCLCYLFL, RANCLCYL	2	9:36169902	CCIN
KRVCTVEL	2	1:186269343	PRG4
KQIPDGFLPI	2	1:78338701	FAM73A
SKLQMIWAF, QMIWAFYL, LQMIWAFYL	2	1:35972480	KIAA0319L
HTRLHAPPSTM, HAPPSTML	2	8:145109542	OPLAH
LPLPPVPFL, LPLPPVPF, HPLPLPPVPF, HPLPLPPV, HPLPLPPVPFL	2	7:128852004	SMO
SQNSGLINHQI	2	18:32917323	ZNF24
KTSTSLGM	2	5:57753012	PLK2
LPRIKSQT, LPRIKSQTIA, LPRIKSQTI, LPRIKSQTIAC	2	1:156561651	APOA1BP
ILFQRLYGPQA	2	5:169805848	KCNMB1
SFPPPRLPAL, SRTSFPPPRL	2	6:31762843--6:31762844	VARS
LAAVVPPM	2	16:84213434	TAF1C
LRSASSTF	2	17:42153276	G6PC3
RSWHLPSHPM, WHLPSHPM, HPMQQTPTTA	2	1:27876869	AHDC1
RQSANLYVHRI	2	4:436526--4:437198	ZNF721

TRMSWWSY, TRPTRMSWWSY, RRAPTTRPTRM	2	19:6213976	MLLT1
WLPVSRRRPL	2	17:63533939	AXIN2
RRQSSPLEV, HRRQSSPL, HRRQSSPLEV	2	2:220494118	SLC4A3
FKFINWSSLL, FKFINWSSL	2	3:151545601	AADAC
TTDCLQML, LAITTDCLQML	2	3:41267325	CTNNB1
APRSTPRA, TPRATRPSCAG, TPRATRPSCA, TPRATRPS, APRSTPRAT, TPRATRPSC	2	2:43452512--2:43452623	ZFP36L2
HELSQPTLQPA, HELSQPTL	2	1:154932027	PYGO2
ILYHRWIV, YLGRTLLVTCI	2	10:890939	LARP4B
RPVQRSQRRA, RPVQRSQRRAP	2	17:73518328	TSEN54
HWGHTKLAF	2	5:179251037	SQSTM1
SRLFSAEKTPL, RLFSAEKTPL, GLQSRLFSA	2	7:97933623	BAIAP2L1
WARAHLAV	2	14:104642560--14:104642555	KIF26A
STKTRQRV	2	12:25378562	KRAS
LEVIATHPT	2	2:37041397	VIT
WRWTRATA	2	19:17039884	CPAMD8
LELPAVPEPPA	2	10:105233172--10:105233174	CALHM3
KTLESAGLF, KTLESAGLFPW	2	3:41860985	ULK4
SSHPWTLSL, SSHPWTLSLTL	2	10:73511467	C10orf54
MFTYVFHDEF, FTYVFHDEF	2	7:132192810	PLXNA4
EVVVHKKRGLF	2	2:148683686	ACVR2A
SPRSWGPRL, VPSPRSWGPRL, GPRLGPAGRI	2	18:3452223	TGIF1
FPRPRPFL, FPRPRPFLA, FPRPRPFLAS	2	19:1231142	C19orf26
RSCVVAVATW	2	19:49611319	SNRNP70
RPPVTQRL, RPPVTQRLV, RPPVTQRLVV	2	2:70315174	PCBP1
FATEVLQMDF, FATEVLQMDFL, FATEVLQM	2	5:112175772	APC
QQFDPLWKRW, KRWNVECLQPL, KRWNVECL	2	9:96860136	PTPDC1
VAMRAQRASRM	2	19:871222	MED16
LPEFHILTV	2	3:151046142	P2RY13
SPGPTSPSL	2	1:150530506	ADAMTSL4
TWGTLRAPSSF	2	1:228559450	OBSCN
SRLLEEKVCAL	2	20:25434140	NINL
WAWVPQRPSSL, WVPQRPSSLL	2	19:10433397--19:10433828	RAVER1
APSCHLSA, APSCHLSAS	2	16:2059622	ZNF598

WLWYCWPTWL, WYCWPTWL, WLWYCWPT	2	1:27106804	ARID1A
GRVGKSALTI	2	1:115258745	NRAS
RSAPPTRGPL	2	X:135956575	RBMX
KSTSVSAI, STSVSAIM, KSTSVSAIM	2	13:102375233	FGF14
GVFTCSWTGPR, FTCSWTGPR	2	7:20371449	ITGB8
LWFREAEI	2	9:101900288	TGFBR1
YYYTNTPRC, YYYYTNTPRC, YYYTNTPRCG	2	19:54745497	LILRA6, LILRB3
CPTKSQVPPL	2	1:65157116	CACHD1
SRTSCWPM, RRASRTSCWPM	2	19:5231449	PTPRS
RRASVPCRPMI	2	1:204228411	PLEKHA6
SLLSVFLL, CSLLSVFLLV, FLLVRPHI	2	4:156716530	GUCY1B3
TFICRSTL, CRKTFICRSTL, CRSTLSQHLRI, CRSTLSQHL	2	19:36832181	ZFP14
KQLLLFLV	2	5:57753012	PLK2
SVGKHSVFPV, SVFPVLFV	2	19:12155709	ZNF878
GRTGRIHVLSF, GRTGRIHVL	2	X:31224764	DMD
QEARGPVPAL	2	17:70117710--17:70117715	SOX9
MANMYQWIRV, FPFDLIIM, FPFDLIIMANM	2	14:96761299	ATG2B
ILFSSVVY	2	1:111216188	KCNA3
KTHPCTMLL	2	7:91936914	ANKIB1
FSKLQVLCL	2	1:203311557	FMOD
AQRASRMRRWW	2	19:871222	MED16
YIAMQGPL	2	2:220161201	PTPRN
IILETVATAGV	2	12:13061271	GPRC5A
KLMHTKIDK, LMHTKIDK	2	10:91497569	KIF20B
FKFNLTPKWI, TPKWIRYFYRI	2	4:15689376	FAM200B
CECYAMVYGY, YRLCECYAMVY, YRLCECYAMV, CECYAMVY, YRLCECYAM	2	12:15035144	MGP
NPIEASLYGL, NPIEASLY	2	16:20335551	GP2
KFDSFRQSL	2	5:112862360	YTHDC2
KAISTRDPLSK, AISTRDPLSK, STRDPLSK	2	3:178936082	PIK3CA
IPPTGSPF, WTIPPTGSPF	2	7:100855927	PLOD3
DQLPPSLRWLF	2	20:60887581	LAMA5
LETVHESNCIF, HESNCIFAF	2	16:23113703	USP31
KKMMELEW	2	18:12699829	CEP76

VYQLRHFSIYF, YQLRHFSIYF, YQLRHFSIY, QLRHFSIY, IYFLSSLL, YQLRHFSIYFL, GVYQLRHFSIY, LRHFSIYFL, VYQLRHFSIY, LRHFSIYF	2	13:45010694	TSC22D1
VAAVCCVQY	2	4:153247289	FBXW7
VEAVDHGWPPM, VEAVDHGW	2	22:46931077	CELSR1
GETATAGAPPA	2	7:100285170	GIGYF1
FFRRLVSTL, FFRRLVSTLNF, LVSTLNFSV, TLNFSVATFML, RLVSTLNFSV	2	10:94243045	IDE
CYDKDRNYY	2	11:1261118	MUC5B
GSYDGHHWM, SYDGHHWMEI, GSYDGHHWMEI, SYDGHHWM	2	22:50943232	LMF2
RRHLWGPF	2	17:70120314--17:70120345	SOX9
FLWRAAGPRPM	2	19:36122298	RBM42
RTSAATKAL	2	18:45567085	ZBTB7C
FIFSLTSPSI	2	8:77767750	ZFHX4
QRRSHPLGKQL, RRSHPLGKQL	2	19:7592423--19:7592444	MCOLN1
STYDLSPIEWL	2	1:211654526	RD3
IQMLTPGSY	2	14:23549379	ACIN1
RRPLPHRPL, HRPLQPPQPL, SRCPSRRPL, SRRPLPHRPL	2	1:203144870	MYBPH
HEFGTGRAV, RAVPRITV, LSHPVYCGHEF	2	12:123351891	VPS37B
RRVQRLQW	2	19:36530332--19:36530343	THAP8
IPRHKTWV, IPRHKTWVV	2	1:166135296	FAM78B
SRPSWPSCPI	2	11:67176565	TBC1D10C
LGRSFLPL	2	16:2059622	ZNF598
APTSPLNPA, APTSPLNPAT, SPLNPATRPA	2	22:46449891	C22orf26
KMYTTSMAMPI, MAMPILPL	2	1:27100176--1:27100350	ARID1A
HRPPELQLLL	2	17:70120194--17:70120314	SOX9
RQELLDIINEL, QELLDIINEL, RQELLDII, TERQELLDII	2	5:122685653	CEP120
ATADLLLALA	2	19:17000632	F2RL3
GADGFPAL	2	2:36825137	FEZ2
RSWNTMQW	2	3:77657038	ROBO2
KLSNAKIAY	2	17:42882279	GJC1
RTFKFMKGHK	2	19:11943750	ZNF440
VTDNQIYW	2	12:12317235	LRP6
MALNTTKTQSK, AMALNTTK	2	2:158115364	GALNT5
CLSDEDLFK	2	9:339015	DOCK8
SESSLSSG	2	17:7798765	CHD3

STVCCMHLHEK, TVCCMHLHEK	2	4:153249385	FBXW7
IMFASIPDFK, CIMFASIPDFK	2	5:7802364	ADCY2
QDIPDGLFW, WMLTCTTLSV, WMLTCTTL	2	1:151206914	PIP5K1A
NRCQRPSRTRL, CQRPSRTRL	2	7:94897945	PPP1R9A
VPIMAHPPL	2	1:55081757	FAM151A
HLYLQDLDFIF, ILVPFFHLY	2	8:113241029	CSMD3
RLSPTHPL, ARLSPTHPL	2	10:126714966	CTBP2
MTACSILAR	2	22:20130522	ZDHHC8
LLTSSSREWKV	2	15:45003781	B2M
AALLCGMREM	2	10:46999601	GPRIN2
RPPWHGLM, CPVHRPPW	2	7:128852004	SMO
HPCCHPCLAL	2	19:10433828--19:10433397	RAVER1
SRQPASGPHL, RSRQPASGPHL	2	2:219920368--2:219920405	IHH
FGFDVPPPTPI	2	17:46629736	HOXB3
AENARSCIQAY	2	5:64905248	TRIM23
TEKGTFRL, TEKGTFRLRW	2	6:32811743	PSMB8
RSLNFGIMPDR, SLNFGIMPDR	2	11:75590944	UVRAG
AWRRFPLL, LWRAWRRFPL, LWRAWRRFPLL	2	22:18300932	MICAL3
VLQCITTI	2	15:33765647	RYR3
QPHDALGHGV	2	7:42005678	GLI3
FGSKVCSPV	2	20:23966824	GGTLC1
AARARVAPW, AARAARARVA, AARAARARV	2	16:88599701	ZFPM1
SQNKRAMRRL	2	6:31778966	HSPA1L
RPAPRPPRRPA, APRPPRRPA	2	16:88599701	ZFPM1
TYPAPYLSLV	2	15:58467216	AQP9
VRCCCWLSL, LASPSLSTSTM, WRPWAGCWV, WLSLASPSL	2	11:618971--11:618999	CDHR5
SLITFICKHLF, ITFICKHLFY, FICKHLFY	2	12:13720042	GRIN2B
DEFVKIIFSVI, VKIIFSVI	2	1:212193518	INTS7
AVMDKRRNL	2	1:211534503	TRAF5
YSDTGNHASM, IYSDTGNHASM	2	X:55044078	ALAS2
FLISDDLRSV	2	22:29837569--22:29837567	RFPL1
WVLGTFAL	2	1:82447508	LPHN2
MLNNRLVGL, MLNNRLVGLSV	2	16:57713122	GPR97
PRMSWCSSL, RMSWCSSL	2	6:33263965	RGL2
APRHARTA, APRHARTATST, RPATVAAA, APRHARTAT, APRHARTATS	2	16:2059622	ZNF598
FTTVLHKQINK	2	6:64421713	PHF3

FTSVPPGFK, TFFTSVPPGFK	2	20:30789952	PLAGL2
GLLQLWKQK, LLQLWKQK	2	1:201437832	PHLDA3
SPFIYFFL, APREQKKISPF	2	5:140857742	PCDHGC3
KLSERNLKCFL	2	6:116836954	FAM26E
APSPSAPSTL, SPSAPSTL	2	8:70744643	SLCO5A1
ARIRVAKTGSL, IRVAKTGSL, SLNAYLHSKR, SLNAYLHSK	2	1:112323370	KCND3
VTATRTTL, VVVTATRTTL, VVTATRTTL, MVVVTATRTTL	2	15:41146715--15:41146120	SPINT1
HSNHLCLLL	2	17:37627694--17:37627688	CDK12
LLSLWTLF	2	3:168833257	MECOM
MNQRPILTI	2	17:7577538	TP53
LADVLLSVHLI, LADVLLSV, LADVLLSVH	2	5:112175424--5:112175351	APC
LFEAWLLLLL, LFEAWLLL, MMIGSQHLLF, LFEAWLLLL, KMMIGSQHLLF, MIGSQHLLF	2	3:77657038	ROBO2
EEFSQVMTLDV, MTLDVFYEL, CEEFSQVMTL, TLDVFYELSK, EEFSQVMTL	2	14:93730195	BTBD7
IRPGRHPPL	2	7:97933623	BAIAP2L1
LPSPPVRST, LPSPPVRSTVC, SPPVRSTVCAM, LPSPPVRSTV, PPVRSTVCAM, SPPVRSTV, APLACLHL	2	12:121432115--12:121432118	HNF1A
MMDPHENI, MMDPHENIL	2	5:160753376	GABRB2
SSSEENVCGSY	2	10:25888009	GPR158
LEAHFEAL, LEAHFEALSL, HQFPLEAHF	2	1:149895562	SF3B4
FFICGPSV	2	8:70514036	SULF1
GQYVTFLRY, FLRYFSDKQK, YFSDKQKISLL, YFSDKQKISL, VTFLRYFSDK, YVTFLRYFSDK, FLRYFSDK	2	9:101980562	ALG2
SYPEFQSSSV, TRRSYPEF, RSYPEFQSSSV	2	5:145883505	TCERG1
AEHSMTPSF, AEHSMTPS, FMAEHSMTPSF	2	13:31735655--13:31732974	HSPH1
FSMMCKEDL	2	19:56329312	NLRP11
NTMMNNNDLV, TMMNNNDLV	2	9:100353693	TMOD1
LRFNLPFRCYL, FNLPFRCYL	2	4:114823493	ARSJ

CPASPHSPGA, CPASPHSPGAS, SPHSPGASPMS, HPAARSSSCC, SPHSPGASPM, CPASPHSPG, SPHSPGAS, SPHSPGASP, SPGASPMSL	2	17:25910016	KSR1
LRLLLLLLPLL	2	19:41858921	TGFB1
LENNQFVV	2	1:154304153	ATP8B2
IRQKPVNLL, RQKPVNLLK	2	16:81969848	PLCG2
YINSGNIPNYL, NIPNYLFDF, NYLFDFFEHL, GNIPNYLFDFF, INSGNIPNYLF, GNIPNYLF, NIPNYLFDFF, GNIPNYLFDF	2	2:32475156	NLRC4
FAQAAFWNHVL, TFAQAAFW	2	12:53647137	MFSD5
AAHGPGEASAM	2	7:5427720	TNRC18
RLVATPAL	2	22:20130522--22:20130515	ZDHHC8
FSKWCRPWL	2	X:56591874	UBQLN2
REASLRWWRVV, REASLRWWRV, DERTREASLRW, ASLRWWRVVFY	2	9:32633583	TAF1L
VSTSGRSTFCV	2	16:2059622	ZNF598
MSMGGPLL	2	5:13830840	DNAH5
SRPHSARLTA, SRPHSARL	2	12:122018740--12:122018773	KDM2B
APPAWSAL, RPALQAPPA, RPALQAPP, RPALQAPPAW	2	9:137677855--9:137688741	COL5A1
GFWLPPPMF, RFCSGVLSGFW, RFCSGVLSGF	2	8:120220776	MAL2
SLASTCWWL	2	2:219920405--2:219920368	IHH
SPMKSFLSEL	2	17:59857686	BRIP1
KTDEVVTLTPH, DEVVTLTPHI	2	5:82817499	VCAN
KVWWRLCKTCY	2	3:38616871	SCN5A
KETWVNFH, SEKKETWVNF, GESEKKETW	2	18:74778307	MBP
MSAFDPLKK	2	3:100451455	TFG
GPTRHHHPA, GPTRHHHPAH	2	X:54209387	FAM120C
RTMQLVVL, RTMQLVVLV	2	10:43604533	RET
VTAFWRSLL	2	19:51015404	ASPDH
IPLTRWKPL, MVMEQAIPL, TRWKPLQTVR, TRWKPLQT	2	6:151673000	AKAP12
LPAAAPPVRL, LPAAAPPV	2	2:43452491--2:43452464	ZFP36L2
ARAPHLQVA, SRCPSARAPHL, ARAPHLQV	2	11:618999--11:618971	CDHR5
ILWTFKFI	2	11:36596949	RAG1
MMVRHTQSSPV	2	10:24813454	KIAA1217

WMRLHFPI, LEYWMRLHF, YWMRLHFPI, LEYWMRLHFPI	2	14:21793439	RPGRIP1
WAFFIICTSKL	2	1:156912567	ARHGEF11
NLLSFSRR, YSGNLLSFSRR, YSGNLLSFSR	2	2:96078466	FAHD2A
CTALWVAVF	2	15:65678360--15:65678319	IGDCC4
FRVLIPQIARV, FVFRVLIPQI, VLIPQIARV, FRVLIPQI	2	9:92006198	SEMA4D
ILFAVRVPISA, ILFAVRVPI	2	12:123351891	VPS37B
LPDHCVHQPV	2	1:207139176	FCAMR
RQLGSAAAEQW	2	10:135000159	KNDC1
SVRQALCQL, LASVRQAL	2	8:145737816	RECQL4
TTNYDFSLYM	2	5:160753376	GABRB2
GTDTLLIQDNV	2	13:20399175	ZMYM5
VRQALCQL	2	8:145737816	RECQL4
WRLLQAQAA	2	1:6614391	NOL9
LLHLHLHEPR, RSAPHVHPHR, RSAPHVHPHRR, RSAPHVHPH, HLHLHEPR	2	17:70119909--17:70120345	SOX9
MNHQRTNSI	2	3:123044275	ADCY5
LEEAGWTLL, LEEAGWTL, SLEEAGWTLL, EEAGWTLL	2	15:41797016--15:41797050	LTK
TRVRVMAI	2	17:7578454	TP53
MHNDFNVM	2	12:130839451	PIWIL1
REAGRFAVL, REAGRFAV	2	1:150530506	ADAMTSL4
YVICQFILI, RVIPICALYV	2	5:138643806	MATR3
ARRRRSSA	2	10:27389197	ANKRD26
TYMGNSSTKL	2	8:116617112	TRPS1
VLLAHIMSRL, LLAHIMSRL	2	X:1584668	P2RY8
LPDPPTASAL	2	17:80274162--17:80274159	CD7
KENCQGWTV	2	12:110449927	ANKRD13A
RPSTLPTPPSC, LPTPPSCL, RPSTLPTPPS, TPPSCLARVSV, LPTPPSCLARV	2	20:3215484--20:3218646	SLC4A11
GRCTCVRSTAL, TALKIWLVL, VRSTALKIWL	2	6:30863251--6:30864547	DDR1
SRSPAAAVRV, SRSPAAAV	2	3:52550119	STAB1
SQIKCVEI, IELSQIKCVEI, SQIKCVEIV	2	5:156635937	ITK
NRSLPGTTRM	2	5:154173390	LARP1
IAANLSLLL	2	16:5075633	NAGPA
CQACSLPAWQL, SLPAWQLSL, CQACSLPAW, WQLSLHTW	2	2:219920368--2:219920405	IHH

MPQMQLQALAM, VTFPPGTAM, TAMPQMQL, MPQMQLQAL	2	17:40939870--17:40937417	WNK4
PPMFLYLYY, LPPPMFLYLYY	2	8:120220776	MAL2
PVYPTRILPR	2	19:10202224	C19orf66
RSHPRPLPA	2	11:118773098	BCL9L
AEGATTADV	2	19:49116359	FAM83E
SSWSSSGR	2	19:49850473	TEAD2
SPFRCYLTY	2	17:7577065--17:7577036	TP53
AEAAPASPL, AEAEAAPASPL	2	16:88599701	ZFPM1
LLLALSFASL, VAAARGLLLAL, GLLLALSFASL, GLLLALSFA, LLLALSFA, LLALSFASL	2	16:28847356--16:28847350	ATXN2L
KTWGFHNNL, KTWGFHNNLL	2	19:37134730	ZNF461
CYNVALEYL, YHFCYNVAL, YHFCYNVALEY	2	1:29652132	PTPRU
FTHCTLATDVM	2	X:10181921	CLCN4
RPGLLGATSSA	2	10:71332204	NEUROG3
KPPRTPPHPAA, HPAARSSSC, HPAARSSSCC, HPAARSSS	2	17:25910016	KSR1
LPRAGTGT, YPTRILPRAGT, LPRAGTGTRIA	2	19:10202224	C19orf66
RAFMQGQLGVV	2	8:145738768	RECQL4
YANYSYASSY, SYPAKAYANY, AYANYSYASSY, KAYANYSY	2	7:96653656	DLX5
GEAALTCG, GEAALTCGG	2	7:100285170	GIGYF1
MTNTGCPL	2	16:30041821	FAM57B
YSKESNYLY, YSKESNYL	2	8:30704275	TEX15
KYSYPSSLSV	2	3:169802469	GPR160
GLLCPALHLV, LLCPALHLV	2	X:56591874	UBQLN2
HMWRAIQTAF	2	17:67247996	ABCA5
MASPGRLL, GRLLCVLGLLL, GRLLCVLGL	2	8:63951312	GGH
SFQTEVSYL, FQTEVSYL, FQTEVSYLEI	2	20:16493483	KIF16B
CRFLGGLL	2	1:49052793	AGBL4
LTIRCCCCRHR	2	21:41151173	IGSF5
FLTRAVPM, STVPFLTRAV, FALRPSTVPF	2	15:75942664--15:75942736	SNX33
LFPGCTPPAYL	2	19:17837512	MAP1S
GMNQRPIL	2	17:7577538	TP53
FYQLAKTCPV, YQLAKTCPV, YQLAKTCPVQL	2	17:7578526	TP53
AIWFQTYL, LAAIWFQTYL	2	2:9098719	MBOAT2
SMACHLQPL	2	19:46832495--19:46832587	HIF3A

LLFDNTTVVY, LLFDNTTVV, LLFDNTTV, LLFDNTTVVYI	2	1:67724321	IL23R
HAAPPLPTAL, AAPPLPTAL	2	17:63533939	AXIN2
CTIDGLLMLLY, CTIDGLLML, TIDGLLMLLY, CTIDGLLM	2	14:45369824	C14orf28
VTVPCGHTF	2	6:30131546	TRIM15
STVTAAPSGQY, TAAPSGQY, TAAPSGQYF, VTAAPSGQYF, VTAAPSGQY, ISAPQPSSSTV	2	6:160485488	IGF2R
KTHSRFHTF, TFAERTECVEF	2	2:98750294	VWA3B
KADQLAQMSI	2	1:220247379	BPNT1
SAACSPSSPF	2	19:871135	MED16
KKMMIGSQHLL	2	3:77657038	ROBO2
VPLVASISAV, VPLVASISA	2	1:22191454	HSPG2
SSCAPPLHRR, CAPPLHRR	2	18:3452223	TGIF1
SLMNVMSV, LERSLMNVM, FTLERSLMNV, FTLERSLMNVM, REFTLERSL, LERSLMNV, FTLERSLM, REFTLERSLM, LERSLMNVMSV	2	3:44636243	ZNF660
SSSSGDALV	2	15:93198679	FAM174B
HLAVRTSM	2	1:204228411	PLEKHA6
RRSTAVRIV, SRRSTAVRIV, SRRSTAVRI	2	15:41099899	ZFYVE19
GLAPPHCV	2	20:57766219	ZNF831
AEREIDML, REIDMLEREL	2	1:233497907	KIAA1804
RTSAMPSAMGV, TSAMPSAMGV	2	1:204228411	PLEKHA6
SRFEDWLKY, KYSERTAAF	2	6:152476106	SYNE1
KARFSTASL, LAKELTHPL, FSTASLAKEL	2	16:68835781	CDH1
YEKKRQFL	2	1:182545478	RNASEL
KPTRAATVSV, TRAATVSV, KPTRAATV	2	17:7579508--17:7579390	TP53
YLAHTHTM, VVYLAHTHTM	2	9:2824764	KIAA0020
FRQLSGDI, RRLWCFRQL, MRRLWCFRQL, WMRRLWCF, RLFWMRRLWCF	2	7:2641075--7:2638193	IQCE
SQLHPWMLL, SQLHPWML	2	12:53703424	AAAS
ATCSQPSSSR	2	16:50745399	NOD2
HPSLHVAA, HPSLHVAALSL, HPSLHVAAL	2	7:99818416--7:99817848	PVRIG
CPAYCSQQGPW	2	1:62253579	INADL

YYILPRKVL, VLPDADTYYI, YYILPRKV, VLPDADTYYIL, YYILPRKVLQM	2	5:112175752	APC
GLLSIADFR, GTGLLSIADFR, LLSIADFR, SIADFRTVLR	2	22:43926712	EFCAB6
LPAGAAAQVA, LPAGAAAQV	2	2:43452491--2:43452464	ZFP36L2
VTAGVGLTATL	2	1:3410404	MEGF6
IRSVATDKWAF	2	1:156912567	ARHGEF11
RRRSQQSWMRL, RRSQQSWMRL, RRSQQSWM, MRLGAGRKSLL, MRLGAGRKSL, RRRSQQSWM	2	17:43482356	ARHGAP27
LSSELLQF, SSELLQFQV	2	18:29041236	DSG3
APFRVNHA	2	5:112175350--5:112175346	APC
GDYEIFGL, YEIFGLPGWAV	2	5:167990927	PANK3
AQFGTVITL	2	5:140222014	PCDHA8
SLTMNPLENVY, YSDDLMET, YSDDLMETLYI, YSDDLMETL, YSDDLMETLY, LTMNPLENVY, TMNPLENVY	2	3:135721560	PPP2R3A
LELIQTINNI	2	8:68116980	ARFGEF1
KRLFWMRRL, RLFWMRRLWCF, WMRRLWCF, MRRLWCFRQL, RRLWCFRQL	2	7:2638193--7:2641075	IQCE
SARPGPWRTSA	2	1:204228411	PLEKHA6
YPVQRLPFST	2	2:242674803	D2HGDH
SEEEEEVPL	2	6:109767002	MICAL1
ATCATTRAPR, TSATCATTR	2	2:60688969	BCL11A
AQNPSLRMWL, MWLGTHSPM	2	16:88494506--16:88494491	ZNF469
TRAHERPHPI, RAHERPHPIHI, RAHERPHPI	2	15:59376343	RNF111
EADMRRSLEL, DMRRSLEL	2	19:33167455	RGS9BP
QYSDIGILYI, YSDIGILYI, YSDIGILY, YSDIGILYIRL	2	11:121000473	TECTA
SPNGKYVYIA, NISPNGKYVY, GINISPNGK	2	7:94937330	PON1
LVVVGAVGVGK	2	12:25398284	KRAS
VTFKPSSEEYL	2	5:82837443	VCAN
AGIINSLLTI	2	11:103029471	DYNC2H1
KQISRSSQSL	2	21:38512962	TTC3
KMKPHPIAIL, KMKPHPIAI	2	2:179638834	TTN
AREAAAAAGVL	2	18:48723146	MEX3C
YRKSGTHQM, IYRKSGTHQM	2	11:58958609	DTX4
FARAKKWVKEL, ARAKKWVKEL	2	3:20017562	RAB5A

SSTPCTVL, LTAPGVCWPL, LTAPGVCWPLL	2	19:42861055--19:42861727	MEGF8
SQAPCLGTW, CLGTWAWV, SQAPCLGTWAW, RMSTVSQAPCL	2	1:110168968--1:110169810	AMPD2
CFSDNALPY, RVCFSDNALPY	2	15:40259920	EIF2AK4
SSWSGCVVQM	2	17:7402719	POLR2A
VPIPNTRPV	2	16:88494491--16:88494506	ZNF469
GRSYDTYTPPI	2	11:31812317	PAX6
KSHHRVVFSH	2	18:34205516	FHOD3
QPPLSLAI	2	1:155041387	EFNA4
APSALGAL, AAHRAAPSAL, AHRAAPSAL, APSALGALT	2	6:18122506	NHLRC1
ALLSEGLDEL, DELPAACL, GLDELPAA, GLDELPAACL	2	20:37555116	FAM83D
FLIIWQNTM	2	11:104878041	CASP5
VENLYPNDII	2	14:103593950	TNFAIP2
GEKNQLTRDW	2	2:231931680	PSMD1
LPAEKEVTELY, LPAEKEVTEL	2	3:132338375	ACAD11
LPRCGCSASS, LPRCGCSAS	2	16:2059622	ZNF598
IHIPRSLSV	2	3:170013720	PRKCI
ERALFRSL, ERALFRSLNL	2	4:87671632	PTPN13
IIYLIPRYVR, YLIPRYVRDL, FIIYLIPRYV	2	21:10916378	TPTE
RWKPLQTVRLM	2	6:151673000	AKAP12
VRNSFEVRV	2	17:7577141	TP53
KSYNFNTF	2	9:99581565	ZNF782
SSCLQIHQR	2	19:44778797--19:44778796	ZNF233
EQALPLWTV	2	1:108700183	SLC25A24
LEVEEWSDM	2	11:115099985	CADM1
FGEEPPAPPV	2	X:128926360	SASH3
WSHHRRML	2	19:16038110	CYP4F11
ALFPKGSQSVV, ALFPKGSQSV	2	4:48597671	FRYL
LPAFDAIDM	2	4:8603117	CPZ
AMILWTLVSL	2	1:46120890	GPBP1L1
LISTNPFNFPF	2	17:10258071	MYH13
LRPRSVVI, SFASLRPRSV	2	16:28847356--16:28847350	ATXN2L
RRSRLELELQI, SRLELELQI	2	11:830670	EFCAB4A
VQLLGADSW	2	18:3452223	TGIF1
ISKFCTGK	2	2:163130424	IFIH1
KFLNSTNSWW, KFLNSTNSW	2	2:27248517	MAPRE3
RTDVMNVV	2	19:52888438	ZNF880
SEGNTPLTCM	2	7:100681721	MUC17
DPSLCMFFEPL, DPSLCMFF	2	2:46987060	SOCS5

HCMDILFTF, RLHCMDILFTF, CMDILFTF	2	3:38888499	SCN11A
SRKKMVHRVV, HRVVCILV, SRKKMVHRV, HRVVCILVWL, HRVVCILVWLL	2	2:237489590	CXCR7
TFTPSASPL, FTPSASPL	2	3:52550119--3:52551362	STAB1
ITMAKQLL	2	8:113241029	CSMD3
FLTPDPGA	2	20:49411698	BCAS4
CPASRLAVPA, CPASRLAV	2	17:36830727	C17orf96
FRDRNTRI, FRDRNTRIL, AFRDRNTRIL	2	11:58962827	DTX4
SRSPQPSL, LRPHPHPL	2	19:4816465	TICAM1
VTMTSCLY, YLDPPTCRAIV, VTMTSCLYL	2	10:81072446	ZMIZ1
RRETPTATM, RETPTATM, ARRETPTATM	2	1:16462199	EPHA2
TLSPVSAF, FAHQGAQEPPV	2	1:3410404	MEGF6
TRFGKKNITI	2	5:56219767	MIER3
RRAPPPACHWL	2	17:56448298	RNF43
ALGALTCHHTF	2	6:18122506	NHLRC1
RRGGHLPV, CRRGGHLPV	2	12:6657973	IFFO1
MQHQRWRGSKW	2	2:189454468	GULP1
LTQERSPM, LTQERSPMGAM	2	19:38028415	ZNF793
SENPFKDKKI	2	1:68171151	GNG12
QTINNIVFF	2	8:68116980	ARFGEF1
EAMFLWRAAV	2	19:36122298	RBM42
TAAARRWWLF, TAAARRWWLFV	2	11:837543--11:837536	CD151
SRLPRIKSQTI	2	1:156561651	APOA1BP
LEGNQIEI, IEIIDQLFAV, NQIEIIDQL, LEGNQIEII	2	5:13928280	DNAH5
YQNPVQSNSF	2	13:61103095	TDRD3
LSAAAVPV	2	20:21493012	NKX2-2
LARAIGYAA	2	10:1094906	IDI1
HAFAGLKIY	2	3:3886823	LRRN1
LSSLCVLLHF, LSSLCVLL, FILPEFHI	2	3:151046142	P2RY13
ILLSRYSL, SRYSLVFFL, LLSRYSLVFFL	2	10:101544462	ABCC2
AVRTSMLTL, AVRTSMLTLL	2	1:204228411	PLEKHA6
ASPGRRPLK	2	7:150698398	NOS3
TRKFTWAL, SAETRKFTWAL	2	13:99738554	DOCK9
LPWHIQIGIL, APLPWHIQI, APLPWHIQIGI	2	12:66622063	IRAK3
RPRVAAAA	2	2:43452491--2:43452464	ZFP36L2
LRSPADPTDL	2	8:22428658	SORBS3

IMANMYQWI, IMANMYQWIRV	2	14:96761299	ATG2B
IRGSTLCF, IRGSTLCFL	2	11:14515315	COPB1
MPAPKSCCTGF, FAFTSPIGL, APKSCCTGFAF	2	19:17955112--19:17954643	JAK3
ALLGASAPCGA	2	1:150530506	ADAMTSL4
LENIQSIF	2	X:138697171	MCF2
KRFEGINIDF, KRFEGINI	2	17:11597209	DNAH9
HWKSQTLQF	2	4:81967605	BMP3
LVSKARPAL, RPALQAPPAW, RPALQAPP, RLVSKARPAL, RPALQAPPA	2	9:137688741--9:137677855	COL5A1
KNLKWLLQL	2	22:21988833	CCDC116
QPLSATPSRVL	2	1:228559450	OBSCN
HRKELCSSV	2	2:100210199	AFF3
IPRRHTRST	2	12:4479724	FGF23
KRSAWRKTTWL	2	16:57113024	NLRC5
RQHRSALMTF	2	16:50745399	NOD2
AAAPTQQL, RAAAPTQQL	2	1:228559450	OBSCN
LESWRRTW	2	10:102762593	LZTS2
TRPEPPPAPV, TRPEPPPAPVL	2	3:52550119	STAB1
YLRFGVQVL, ILYLRFGVQVL	2	6:159049469	TMEM181
AEAPADCGPAQ	2	8:145747920	LRRC24
DRIGYFPPAL	2	17:73501069	CASKIN2
QLLNQIQR	2	6:109294665	ARMC2
FVIIVFAYV, FVIIVFAY	2	2:167162315	SCN9A
SSAARAGM	2	7:100285170	GIGYF1
FASVVNYFI	2	13:48615145	NUDT15
IQLMTTRNFW, SAKNSNIQLM, QLMTTRNFWPM, MTTRNFWPM, LMTTRNFWPM	2	8:92352747	SLC26A7
SLLERVLFYR, MSLLERVLFYR, RVLFYRAR	2	3:3067806	CNTN4
LLTSKLFSYK, TSKLFSYK, LTSKLFSYK	2	7:34989478	DPY19L1
TRWLPQAAKL	2	1:27088681--1:27088668	ARID1A
RTAAWQLCCR, RTAAWQLCCRR	2	16:50745399	NOD2
FVLNPCLPSIM	2	22:38895455	DDX17
SRASGTARTWL, RASGTARTWL	2	17:80049354	FASN
AMMCASTTSW, AMMCASTTSWR, MMCASTTSWR	2	8:22054260--8:22054259	BMP1
HRSHLKQHQRI	2	19:57840047	ZNF543

APSVRGDAV	2	20:62321691	RTEL1
CNIRFSRHETY, RHETYTVHKRY	2	16:88599701	ZFPM1
RMMRRQWSLWV, RQWSLWVL	2	6:116836954	FAM26E
HPKVHLNTMF, MFRRPHSCL	2	5:112175351--5:112175350	APC
ASSVLPCHSLL	2	2:73302665--2:73302655	RAB11FIP5
VVMDRICSFPL, VVMDRICSF, VMDRICSFPL, VMDRICSF	2	6:90397868	MDN1
LFPSTTTKLL, FSNCSGLF, SGLFPSTTTK, GLFPSTTTK, GLFPSTTTKL, CSGLFPSTTTK	2	8:100520130	VPS13B
TVFGGFLKYTV	2	18:7036080	LAMA1
SRCSTPTTSL, LRSRSLSL, LRSRSLSLPL	2	17:43482356	ARHGAP27
SLLTCSWL	2	11:66055105	YIF1A
RPSTRFLTVP, RPSTRFLT, SPRPSTRFLT	2	7:139167934	KLRG2
RLFHPWTTDM	2	2:171572940	SP5
WTVAPTAL, HQVPCPWCPW	2	17:41610700	ETV4
RPLPARDKHAL, LPARDKHAL	2	6:70423652	LMBRD1
MPPSSPSMASL, MASLCWSTV	2	19:17836781	MAP1S
HEIIHTGGKPY, YASHIVQHEII, YASHIVQHEI	2	19:38230678	ZNF573
YILGSVDK	2	12:124317763	DNAH10
SERDYNMDF, RESERDYNM, RDYNMDFLTL, RDYNMDFL, SERDYNMDFL, RESERDYNMDF	2	1:100889837	CDC14A
HLVSQSIYH, KLHLVSQSIY, KLHLVSQSIYH	2	3:193332592	OPA1
FTSLSSASLPR, TSLSSASLPR, LSSASLPR	2	16:50745399	NOD2
RSRRENVGLY	2	14:59113430	DACT1
SYIHYVFHL, YVFHLPNTY	2	1:63021531	DOCK7
RATEVPSLTAL, FLAGDGRATEV	2	2:1481231	TPO
MRGPPPLMPPL	2	1:149895562	SF3B4
QRHWSSKINL, QRHWSSKI	2	6:158850873	TULP4
RRVQRLTL, CRRVQRLTL	2	11:64597506	CDC42BPG
MMLPVRARTAR, RSALPQGRAPL, MMLPVRAR	2	18:34205516	FHOD3
KDVWNFKAW, FESLCSKKDVW	2	17:38711971	CCR7
FQMMEGKAFL, QMMEGKAFL	2	11:124845049	CCDC15
RWKPLQTVRL, RWKPLQTV	2	6:151673000	AKAP12

FTMARASVNEW, TARPLSLTLSF, FTMARASV	2	1:155221333--1:155221578	FAM189B
ATQASMTL	2	22:20130522--22:20130515	ZDHHC8
AEVLSDHIAFL, AEVLSDHIAF, DEAEVLSDHI, VLSDHIAFL, LSDHIAFL	2	2:215845310	ABCA12
GPKSLRPPV, SPSGPKSL, RPPVWAPPT	2	19:39330959--19:39330958	HNRNPL
RKLEAFFAF, FAFSSLLWPS	2	12:51392999	SLC11A2
HAWSRLRGL	2	19:49376582	PPP1R15A
SSAARRSNFR, SAARRSNFR, ASSAARRSNFR	2	2:60688969	BCL11A
KLFSELEF, NEPWYKLF	2	10:97135757	SORBS1
YQMILQQDF	2	16:20673143	ACSM1
CRNLWLTSM	2	1:110168968--1:110169810	AMPD2
SLHNRTWYV, LQSLHNRTW	2	3:57561285	ARF4
GETQNTFL, LEEPGETQNTF	2	20:5903379	CHGB
FRTESPPAQAV, TESPPAQAVM, TESPPAQAV, TESPPAQAVML	2	10:102762593	LZTS2
VLGGKAAIPEV	2	1:62672556	L1TD1
SEPLATPRHQY, LATPRHQYY	2	X:47072566	UBA1
HMDCLRWL	2	12:133250289	POLE
SRNKSELL	2	12:58025103	B4GALNT1
HPQWGSGV, HPQWGSGVPRA	2	20:62326110	RTEL1
RTLNGSQM	2	19:18497024	GDF15
SVDPFPQVKL, LLASVDPFPQV, SVDPFPQV	2	14:30068249	PRKD1
YLGRTLLV, YLGRTLLVTCI, NWNSAYLGRTL	2	10:890939	LARP4B
HECSLWGSWL	2	19:44150698	PLAUR
AQNILSYLNL	2	14:104026187	BAG5
RATPLSSL, SRRATPLSSL, RRATPLSSL	2	12:124848228	NCOR2
VAMWISSVF, RVAMWISSV, VAMWISSV, RVAMWISSVF, AMWISSVFEA, AMWISSVF	2	6:36452576	KCTD20
YASAVSSL	2	6:42957352	PPP2R5D
ITYSHSPPR	2	12:20522723	PDE3A
FLWTKDGNPF	2	3:369855	CHL1
STASPCSLTSL, TASPCSLTSLR	2	8:22054260--8:22054259	BMP1
LAAPGGRARL, RARLGQGRQTM	2	20:21493012	NKX2-2
ASASTSTPSR, STSTPSRQLHR	2	16:50745399	NOD2
RLPELRQPQRL	2	2:43452439--2:43452244	ZFP36L2
FIIFLRISAYM	2	6:51889974--6:51890015	PKHD1

HEHAQREVQRL	2	1:17266536	CROCC
FPALACSLE, FPALACSLEEK, FPALACSLEE	2	2:36825137	FEZ2
APTSCGPTRA	2	8:120220776	MAL2
RPPELQLLL	2	17:70119909--17:70120194	SOX9
GEWQPIQITM	2	2:68544315	CNRIP1
YNVDALRYFV, GLYNVDALR, LYNVDALRYF, YNVDALRYF	2	8:28385376	FZD3
AMTPPVLTTAK, TTAKTSVK, LTTAKTSVK, MTPPVLTTAK, VLTTAKTSVK	2	3:151163622	IGSF10
ILLGGSTSSWA	2	17:16330835	TRPV2
RRAHERPHPI, RAHERPHPI, RAHERPHPIHI, SRRAHERPHPI	2	15:59376343	RNF111
KRIPQIRI	2	13:73347916	DIS3
VEYVQLVT, AQVEYVQLV, VEYVQLVTEL	2	6:105198346	HACE1
CLHLPSPPV	2	12:121432115--12:121432118	HNF1A
HQTSVLSF, FTTANTVPTAI	2	9:109689491	ZNF462
LRHKRDKDYL, RVYDLRHKRDK	2	8:77617034	ZFHX4
CPASCSGCYSW, SLLRFCPA, WTIPPTGSPF, CPASCSGCY	2	7:100855927	PLOD3
YSAPVPPNAL, FYSAPVPPNAL	2	20:33328934	NCOA6
RTSYLENK, TSYLENKLSCK	2	1:247588527	NLRP3
ISVTPLPLQPY, ISVTPLPL	2	1:62253580	INADL
SPADPTDL	2	8:22428658	SORBS3
CRVSTWQGTL, STWQGTLAL	2	19:7592444--19:7592423	MCOLN1
YPPDNLRVM	2	19:50462324	SIGLEC11
IACRVTPM	2	16:2059622	ZNF598
FSLYLIHEI	2	19:12059564	ZNF700
LECRTRHPNM, CRTRHPNMEL, CRTRHPNM, RTRHPNMEL, TRHPNMEL	2	16:10729660	TEKT5
SLYQYFENF	2	14:78045431	SPTLC2
FAFSSLLW, WPSHLDMSML, FSSLLWPSHL, WPSHLDMSM	2	12:51392999	SLC11A2
GRLPALSLL, GRHAPGGRL, GRLPALSL	2	17:46629736	HOXB3
YTQSEMEFL, YTQSEMEF, YTQSEMEFLL	2	1:237947200	RYR2
EEGGCASGTW, GEEGGCASGTW	2	X:153042691	PLXNB3
RSFQIHER	2	19:12574841--19:12574925	ZNF709
AATLRCPAV	2	3:51417604	DOCK3
RRIQSRSPFPL, FPLYSGRLL	2	11:46702223--11:46702224	ARHGAP1

MQNHTAEKPY	2	19:37441368	ZNF568
ATASAAPRPPR, ATASAAPR, RAATASAAPR	2	17:70119870--17:70119913	SOX9
SRWMTSLI	2	16:14346300	MKL2
KLWNLLMPM	2	4:141461344	ELMOD2
WRQLWLFLLLL, WRQLWLFLL, WLFLLLLLPGA, FLLLLLPGA, WRQLWLFL, WLFLLLLL, RQLWLFLL, WRQLWLFLLL	2	1:179051300	TOR3A
KVDPVSLRKV	2	5:176026129--5:176026138	GPRIN1
VEAMPLCLL, WEVEAMPLCL, VEAMPLCL, WEVEAMPLCLL, VEAMPLCLLPA	2	2:120005381	STEAP3
YSVANLAKYL	2	5:140515167	PCDHB5
FKHPHVANPQL	2	13:113854748	PCID2
ILFSTMWF	2	8:142221695	SLC45A4
PLDSSPLQLLL	2	3:77657038	ROBO2
FADMERHHILF	2	3:25668727	TOP2B
HRHGLLPAEL	2	1:27088681--1:27088668	ARID1A
TPACTASC	2	11:76751542--11:76751604	B3GNT6
VSSSTVSSVM, SSSTVSSVM, SSTVSSVM	2	3:183508631	YEATS2
TAAHVTDMV, VEVETVTQAA, ITAAHVTDMV	2	X:135956571--X:135956575	RBMX
LYIQNALNL	2	12:122018773--12:122018740	KDM2B
CRRPWPPRL, RRPWPPRL, RRPWPPRLTPL	2	12:132394804	ULK1
HERGALPRRAL	2	17:70117710--17:70117715	SOX9
YVPGPTNETL	2	16:77353780	ADAMTS18
LPSKPFANSSL	2	22:28389367	TTC28
IQCFILMYF	2	7:33014804	FKBP9
VLISPLMCV, WLVLISPL, WLVLISPLMCV, TALKIWLVL	2	6:30864547--6:30863251	DDR1
RPSRRGPEA, AEAEAAPASPL	2	16:88599701	ZFPM1
ATWLSLAL	2	10:124895627	HMX3
RQLSSKSLW	2	14:75230947	YLPM1
LRLELAKM	2	1:39332608	MYCBP
TVDCKINCLL, TVDCKINCLLL	2	3:151046142	P2RY13
VPCSMQDPL	2	3:168833257	MECOM
RNNSAFLK, RNNSAFLKK, IRNNSAFL, NIRNNSAFLK	2	18:56202169	ALPK2
RRGPGGQLRL, RRGPGGQLRLM, GRRGPGGQLRL, LMLPSSARPW, RRGPGGQL	2	9:17135271	CNTLN

LARPVSAA	2	7:75677504	MDH2
HPPHRHPGGCA, HPGGCADHPGL, RPDHPPHRHPG	2	8:145625525	CPSF1
KDNIFGTVI	2	5:38510602	LIFR
SLWIEAMVLK, WIEAMVLKK, SLWIEAMVLKK, NYSLWIEAM, YSLWIEAMVLK	2	4:153881906	FHDC1
KLRQNQNY, LRQNQNYMNIL	2	2:234078674	INPP5D
GEDIFWTCL, GEDIFWTCLKM	2	16:11141159	CLEC16A
REAAAAAGV	2	18:48723146	MEX3C
TPPPPPLL	2	1:109792735	CELSR2
FRATPLQGM, FRATPLQGML	2	2:238289624	COL6A3
QACLTFRGSW	2	10:64564892	ADO
DVTPTTSSLV	2	19:13882968	MRI1
LSFWQQLTF	2	3:151046142	P2RY13
LMNLATADLL	2	19:17000632	F2RL3
TRTLCLFPRPV, FQMHTRTLCL, CLFPRPVVAF, FQMHTRTLCLF	2	11:47304002	MADD
KSYKMNGREY	2	2:202514862	MPP4
MRWSSQTCSL, RRMTRPNM	2	19:5231449	PTPRS
SATVTAFWRSL	2	19:51015404	ASPDH
ELRALREM	2	13:46288145	SPERT
TMLHGQVMDQK	2	15:42371752	PLA2G4D
QQNELKKTY	2	X:84526620	ZNF711
TRTFIAARTFV, VTRTFIAAR, TRTFIAARTF	2	13:94482727	GPC6
SQTHLNTL	2	15:72143639	MYO9A
MANRNHLKL, KMANRNHL	2	5:33576343	ADAMTS12
RQTSFCTLTW	2	22:50297991	ALG12
QEQQNVLKVL, QEQQNVLKV	2	18:73000406	TSHZ1
REVITVYQLFV, REVITVYQL, REVITVYQLF	2	16:83251091	CDH13
FRLFIQNL, FIQNLFFQI, FRLFIQNLFF, FRLFIQNLF, RRTTFRLFI, RLFIQNLFFQI	2	3:98506942	ST3GAL6
HTEMIKKEER	2	15:52244057	LEO1
TDFGLVREW	2	1:233482260	KIAA1804
SATEEGNTQVL	2	9:124855211	TTLL11
SPSVHNVKET, SPSVHNVKETA	2	3:195518119	MUC4
FHRIQNQL, NQLQLFRNFFL, FHRIQNQLQL	2	6:117622233	ROS1
SRLLSRTAM	2	10:114900984	TCF7L2
LTSPAVCL	2	6:83819938	DOPEY1
CPMTACSIL, CPMTACSI	2	22:20130515--22:20130522	ZDHHC8

YFREAYFWL, TRNILDYF, LDYFREAYFWL	2	3:31666425	STT3B
FFYRHPSY	2	1:205589099	ELK4
ARCPRVGCL	2	16:28847356--16:28847350	ATXN2L
LEKAVQSFW, LEKAVQSFWY	2	6:107361391	C6orf203
FQNWKWITVYL, FQNWKWITV	2	7:103205756	RELN
FFFGFWLV	2	19:56249721	NLRP9
SPASACTMPPL, QPSPASACTM, MPPLLHPQW, SPASACTM	2	17:48433967	XYLT2
SQLPASSPTK	2	18:34205516	FHOD3
YQQWNITSPEV, QQWNITSPEV	2	7:4056840	SDK1
SPLDSSPL	2	3:77657038	ROBO2
YWKRHIEL, NYWKRHIEL	2	7:73021716	MLXIPL
GLGDGPAQPEI	2	19:55815035	BRSK1
GEWGAFNDIMW	2	14:102463483	DYNC1H1
HSNDGGNILYY	2	1:65332611	JAK1
SQNTRVPRLPK	2	6:32918419	HLA-DMA
YSAAPGAPPL	2	10:102587324	PAX2
TRKSPALSAL	2	3:52550119	STAB1
HPQRKRRGGPP, PPSPPLAL, GPPSPPLAL	2	17:56435161	RNF43
LEKNVPLRVKI, FLEKNVPL	2	1:156931470	ARHGEF11
KVWSRTAYL, SSAKVWSRTAY	2	12:64819643	XPOT
YLDPPTCRAI, YLDPPTCRAIV, SYLDPPTCRAI	2	10:81072446	ZMIZ1
REIQTAVHLLL, REIQTAVHLL, REIQTAVHL	2	6:27783120	HIST1H2BM
LLVSSPPSGV, WLLVSSPPSGV	2	1:85040070	CTBS
YSTQYGLKL	2	2:159537101	PKP4
GRPLRPPI	2	8:54792097	RGS20
KRDQCFQM	2	11:124845049	CCDC15
MDFSLVFSPSF	2	4:113540617	C4orf21
WPCLWNSLLRF, SLLRFCPA, WPCLWNSLL, WPCLWNSL	2	7:100855927	PLOD3
WRVEQRKIL, WRVEQRKILL, WRVEQRKI, RIQDFVWR	2	5:14336771	TRIO
YKNFLRCL, YKNFLRCLVI, LRSAEAYKNFL, AYKNFLRCLV, AYKNFLRCL, AYKNFLRCLVI	2	15:75694278	SIN3A
FTRQSTGTIAY, QSTGTIAY	2	4:187158051--4:187158019	KLKB1
LEYFMKQINDA, LEYFMKQI	2	3:178952074	PIK3CA
MEANMSIPSM	2	14:105408302	AHNAK2
LEDSSGNLLV	2	17:7577141	TP53
HQADNLLM, SRHQADNLLM, SRHQADNLL, SRHQADNL	2	22:40356095	GRAP2
SEAPGAQYPP	2	19:12996740	KLF1
HICSRRFK	2	18:74154167	ZNF516
SLKEKVNLQK	2	5:13920716	DNAH5
LPQGRRDL, LPQGRRDLRA	2	8:145625525	CPSF1
SEREREAAL	2	17:70117715--17:70117710	SOX9
VYGGNVVMLV	2	17:65119099	HELZ
MLVMHNMFSHR, SYMLVMHNM, VMHNMFSH, MLVMHNMFSH, VMHNMFSHR, LVMHNMFSHR, SYMLVMHNMF	2	12:57992945	PIP4K2C
FRTARKGEKF	2	11:58962827	DTX4
YAVSLAAL, SASYAVSL, SASYAVSLAAL	2	19:47549454	TMEM160
LQSSLNPAASV	2	14:76368544	TTLL5
NSFKYLLDQLK	2	12:112479863	NAA25
NQIVIRGLNTI	2	3:41277291	CTNNB1
FTSLSSASL	2	16:50745399	NOD2
QRFNPAMFNI, FRGQRFNPAM	2	7:44425654	NUDCD3
FVIIVFAYL, FVIIVFAY	2	2:167162315	SCN9A
SARTGRSV, SARTGRSVSA	2	22:20130515--22:20130522	ZDHHC8
MRGSELPL	2	18:56887507	GRP
GPRMQLCT, SPPLALGPRM, PPLALGPRMQL, GPRMQLCTQLA	2	17:56435161	RNF43
LLDSLEPL, VENRAEMALL, AEMALLDSLEP, AEMALLDSLE, AEMALLDS, VENRAEMAL, AEMALLDSL	2	3:48612871	COL7A1
TRSCSISL	2	1:155308000	ASH1L
STLNFSAHLLM	2	10:94243045	IDE
IQNYHLRGIGM	2	1:85020800	CTBS
ALHPLWWA, ALHPLWWATA	2	1:206650063	IKBKE
DEDIVTILQA, DEDIVTILQAT, DEDIVTIL	2	20:1445016	NSFL1C

Table S5: Accession numbers of the microarray profiles of colorectal cancer

Dataset	Platform	#Sample	Publication (PMID)
TCGA	RNAseqV2	538	22810696
GSE12945	HG-U133A	62	19399471
GSE41258	HG-U133A	187	19359472
GSE14333	HG-U133_Plus_2	290	19996206
GSE17538	HG-U133_Plus_2	240	22115830
GSE29623	HG-U133_Plus_2	130	22362069
GSE33113	HG-U133_Plus_2	90	22496204
GSE39582	HG-U133_Plus_2	566	23700391
GSE24549	HuEx-1_0-st	83	21619627
GSE24550	HuEx-1_0-st	77	22991413
GSE30378	HuEx-1_0-st	95	22991413
GSE28722	Rosetta custom human 23K array	125	21251323

Table S6: List of features used for the regression model.

Parameter	Description
HLA-A	Antigen presenting machinery
HLA-B	Antigen presenting machinery
HLA-C	Antigen presenting machinery
HLA-E	Antigen presenting machinery
HLA-G	Antigen presenting machinery
HLA-F	Antigen presenting machinery
Neo-antigen frequency	Antigen presenting machinery
B2M	Antigen presenting machinery
TAP1/2	Antigen presenting machinery
Heterogeneity	Tumor heterogeneity
Treg	Immunosuppressive TILs
MDSC	Immunosuppressive TILs
ATAD2	Cancer germline antigens
CASC5	Cancer germline antigens
CEP55	Cancer germline antigens
CASC5	Cancer germline antigens
CSAG1	Cancer germline antigens
CSAG2	Cancer germline antigens
CTAGE5	Cancer germline antigens
CTCFL	Cancer germline antigens
DCAF12	Cancer germline antigens
GPATCH2	Cancer germline antigens
KDM5B	Cancer germline antigens
KIAA0100	Cancer germline antigens
LEMD1	Cancer germline antigens
MAGEA12	Cancer germline antigens
MAGEA2	Cancer germline antigens
MAGEA3	Cancer germline antigens
MAGEA6	Cancer germline antigens
IGF2BP3	Cancer germline antigens
MPP1	Cancer germline antigens
NUF2	Cancer germline antigens
ODF2	Cancer germline antigens
OIP5	Cancer germline antigens
PBK	Cancer germline antigens
PIWIL1	Cancer germline antigens
POTEE	Cancer germline antigens
RQCD1	Cancer germline antigens
SPA17	Cancer germline antigens
SPAG1	Cancer germline antigens
SPAG4	Cancer germline antigens
SPAG9	Cancer germline antigens
TTK	Cancer germline antigens
ZNF165	Cancer germline antigens
SEMG1	Cancer germline antigens
IDO	co-inhibitor
PDL1	ligand

B7H2	ligand
CD28	co-stimulator
ICOS	co-stimulator
LAG3	co-inhibitor
BTLA	co-inhibitor
CD160	co-inhibitor
B7DC	ligand
HVEM	ligand
CD137L	ligand
OX40	co-stimulator
OX40L	ligand
CD70	co-stimulator
GITRL	ligand
GITR	co-stimulator
BAFF	ligand
BAFFR	co-stimulator
APRIL	ligand
CD40L	co-stimulator
CD40	ligand
TACI	co-stimulator
AIMP1	Cytokine
BMP10	Cytokine
BMP13	Cytokine
BMP2	Cytokine
BMP4	Cytokine
BMP5	Cytokine
BMP6	Cytokine
BMP7	Cytokine
CSF2	Cytokine
CSF3	Cytokine
CTF1	Cytokine
EDA2R	Cytokine
IFNA1	Cytokine
IFNA13	Cytokine
IFNA14	Cytokine
IFNA16	Cytokine
IFNA17	Cytokine
IFNA2	Cytokine
IFNA21	Cytokine
IFNA4	Cytokine
IFNA5	Cytokine
IFNA6	Cytokine
IFNA7	Cytokine
IFNA8	Cytokine
IFNAR1	Cytokine
IFNAR2	Cytokine
IFNG	Cytokine
IFNGR1	Cytokine
IFNGR2	Cytokine
IFNK	Cytokine

IL10	Cytokine
IL10RB	Cytokine
IL11	Cytokine
IL12B	Cytokine
IL12RB1	Cytokine
IL12RB2	Cytokine
IL13	Cytokine
IL15RA	Cytokine
IL16	Cytokine
IL17B	Cytokine
IL17RA	Cytokine
IL17RB	Cytokine
IL18	Cytokine
IL19	Cytokine
IL1A	Cytokine
IL1B	Cytokine
IL1R1	Cytokine
IL2	Cytokine
IL20	Cytokine
IL21	Cytokine
IL21R	Cytokine
IL22	Cytokine
IL23	Cytokine
IL23R	Cytokine
IL24	Cytokine
IL25	Cytokine
IL27	Cytokine
IL28RA	Cytokine
IL29	Cytokine
IL2RA	Cytokine
IL3	Cytokine
IL31RA	Cytokine
IL32	Cytokine
IL33	Cytokine
IL36RN	Cytokine
IL4	Cytokine
IL4R	Cytokine
IL5	Cytokine
IL6	Cytokine
IL7	Cytokine
IL8	Cytokine
IL9	Cytokine
PF4	Cytokine
TGFA	Cytokine
TGFB1	Cytokine
TGFB2	Cytokine
TGFB3	Cytokine
TGFBR1	Cytokine
TGFBR2	Cytokine
TGFBR3	Cytokine

TNF	Cytokine
TNFRSF10A	Cytokine
TNFRSF10B	Cytokine
TNFRSF10C	Cytokine
TNFRSF10D	Cytokine
TNFRSF11B	Cytokine
TNFRSF12A	Cytokine
TNFRSF13B	Cytokine
TNFRSF19	Cytokine
TNFRSF1B	Cytokine
TNFRSF21	Cytokine
TNFRSF25	Cytokine
TNFRSF3	Cytokine
TNFRSF4	Cytokine
TNFRSF6	Cytokine
TNFSF10	Cytokine
TNFSF11	Cytokine
TNFSF12	Cytokine
TSLP	Cytokine
CCL11	Chemokine
CCL15	Chemokine
CCL16	Chemokine
CCL17	Chemokine
CCL18	Chemokine
CCL2	Chemokine
CCL20	Chemokine
CCL22	Chemokine
CCL23	Chemokine
CCL25	Chemokine
CCL27	Chemokine
CCL28	Chemokine
CCL3	Chemokine
CCL4L2	Chemokine
CCL5	Chemokine
CCL7	Chemokine
CCL8	Chemokine
CCR1	Chemokine
CCR10	Chemokine
CCR3	Chemokine
CCR4	Chemokine
CCR6	Chemokine
CCR8	Chemokine
CCR9	Chemokine
CCRL1	Chemokine
CCRL2	Chemokine
CKLF	Chemokine
CX3CL1	Chemokine
CX3CR1	Chemokine
CXCL1	Chemokine
CXCL10	Chemokine

CXCL11	Chemokine
CXCL12	Chemokine
CXCL13	Chemokine
CXCL14	Chemokine
CXCL16	Chemokine
CXCL17	Chemokine
CXCL2	Chemokine
CXCL3	Chemokine
CXCL5	Chemokine
CXCL6	Chemokine
CXCL9	Chemokine
CXCR1	Chemokine
CXCR2	Chemokine
CXCR2P1	Chemokine
CXCR3	Chemokine
CXCR4	Chemokine
CXCR5	Chemokine
CXCR6	Chemokine
CXCR7	Chemokine
DARC	Chemokine
XCL1	Chemokine
XCL2	Chemokine
XCR1	Chemokine
CXCL7	Chemokine
